# Uplift and denudation in the continental area of China linked to climatic effects: evidence from apatite and zircon fission track data

**DOI:** 10.1038/s41598-018-27801-7

**Published:** 2018-06-22

**Authors:** Nansheng Qiu, Shuai Liu

**Affiliations:** 1 State Key Laboratory of Petroleum Resources and Prospecting, China University of Petroleum, Beijing, 102249 China; 2 College of Geosciences, China University of Petroleum, Beijing, 102249 China

## Abstract

Approximately 2284 fission track data were collected to draw a fission track thermotectonic image of the continental area of China. The result exhibits features such that apatite fission track ages increase from the southwestern to eastern and northern continental areas of China. Thermal paths also reveal the different uplift/denudation processes and times between different tectonic units. At the same time, tectonic uplift of the continent has been among the causes of climate change in the continent since the Cenozoic. The uplift of the Qinghai-Tibet Plateau since the Oligocene is the main cause of the formation of the Asian monsoon and inland droughts, and rapid uplift of the Tibet Plateau after the Pliocene has changed the atmospheric circulation. The main period of climate aridity in Central Asia was caused by the rapid uplift of the Tianshan Mountains since the Miocene, and rapid uplift during the Late Miocene to Pliocene intensified the process of aridity. This study provides the first thermotectonic image of uplift and denudation in the continental area of China and provides a new dating of the formation of the Asian monsoon and climate aridity in Central Asia.

## Introduction

Fission track of minerals can reveal crustal tectonic movement. Apatite fission track (AFT) may reveal thermal events of ~120 °C^1^, and zircon fission track (ZFT) can reveal higher thermal events of 210~240 °C^2,3^. Regional fission track age (and track length) patterns are the result of cooling in the near-surface environment due to the interaction of surface processes and underlying tectonics^4,5^. It has also become clear that low-temperature thermochronology is providing a window into upper crustal processes that are often not discernable by other geochronological methods^6^. The increasing amounts of fission track data over the past 30 years in China have enabled us to build a fission track thermotectonic image of the continental area of China. The overall aim of this paper is to determine the time of tectonic uplift and its influence on climate changes at macroscopic scale. The paper will produce a regional coverage of apatite and zircon fission track data across an entire continent in a form that can be combined and compared with other continental-scale data sets, for example, those of heat flow and digital topography. This approach can provide new interpretations of the thermal and tectonic evolution of the continental area of China. The increasingly large data set resulting from this study can be used to image the evolution of the upper part of the continental crust and to reconstruct the denudation history of the land surface. In addition, some climatic effects, such as the aridity in central Asia and the Asian monsoon, can also be referred to the uplift of plateaus and mountains.

## Geological Setting

**Figure 1:**
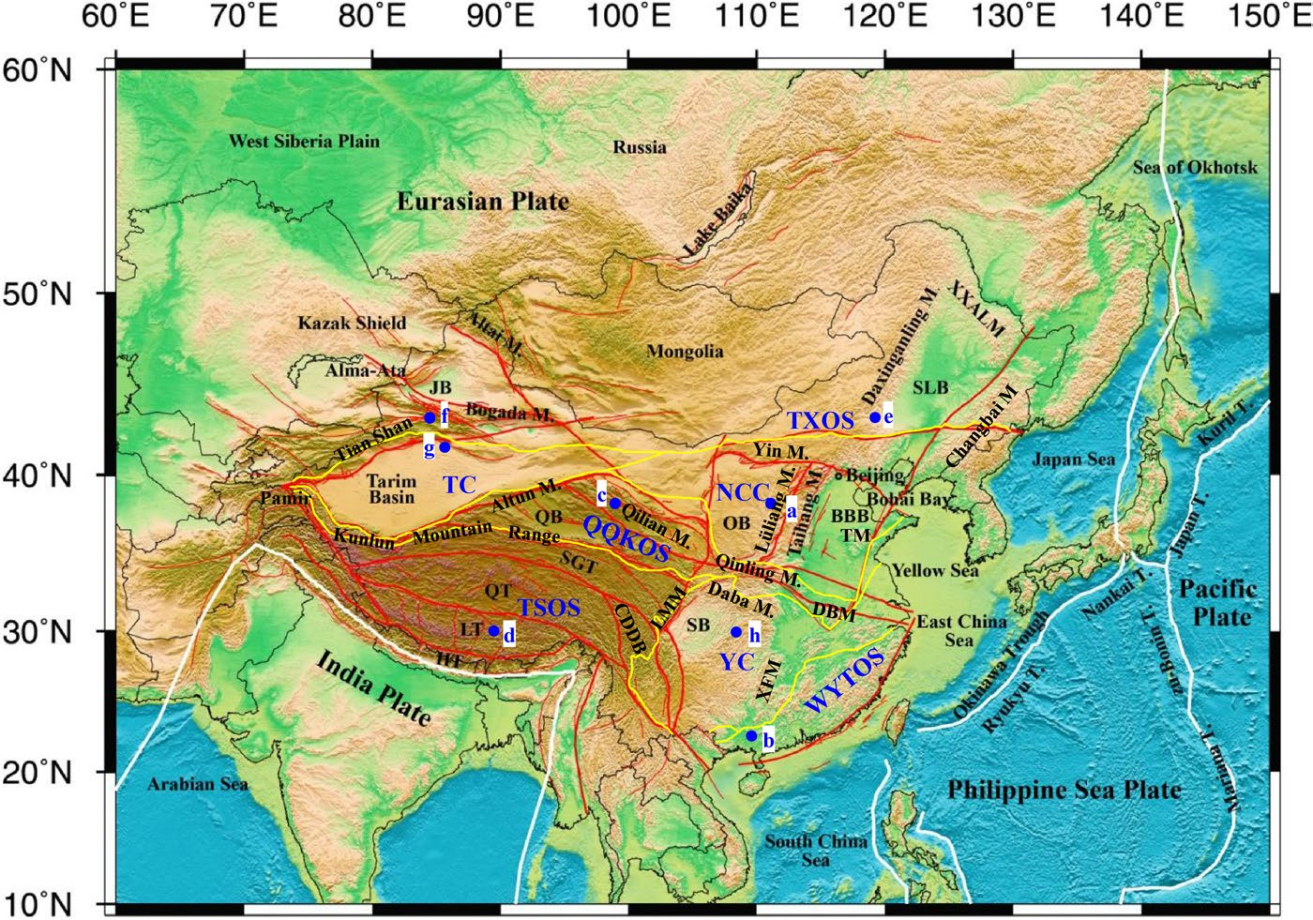
Topography and tectonic background of China and its surroundings. Color shows the surface topography. White curved lines show the plate boundaries. Red lines show the large fault zones and/or tectonic block boundaries in continental China. The yellow lines are tectonic unit boundaries from Pan *et al*.^28^. NCC = North China Craton, QQKOS = Qinling-Qilian- Kunlun Orogenic System, TC = Tarim Craton, TSOS = Tibet-Sanjiang Orogenic System, TXOS = Tianshan-XingMeng Orogenic System, WYTOS = Wuyi-Yunkai-Taiwan Orogenic System, YC = Yangtze Craton. Blue solid circles show the locations of typical apatite samples which were used for thermal paths modeling in each tectonic unit. JB = Junggar Basin, QB = Qaidam Basin, SB = Sichuan Basin, OB = Ordos Basin, BBB = Bohai Bay Basin, SLB = Songliao Basin, SGT = Songpan-Ganzi Terrane, CDDB = Chuandian Diamond Block, QT = Qiangtang Terrane, LT = Lhasa Terrane, HT = Himalaya Terrane, XXAXM = Xiaoxinganling Mountain, TM = Tai Mountain, DBM = Dabie Mountain, LMM = Longmen Mountain, XFM = Xuefeng Mountain, M. = Mountain, T. = Trough.

The continental area of China is comprised of the southeastern part of the Eurasian Plate and north rim of the Indian Plate, and is bordered by the Pacific Plate to the east **(**Fig. 1**)**. It is located in one of the most complex tectonic domains in the world, where the Paleo-Asian Ocean, Tethyan and Western Pacific domains met in a triangular framework, carrying a mosaic of ancient cratonic blocks and orogenic belts built through different tectonic regimes^7,8^. The fundamental tectonic framework of the continental area of China has been in place since the Early Mesozoic^9^. However, different tectonic evolutions have been experienced in the eastern, middle and western parts of the continental area of China **(**Fig. 1**)**.

There is a general uplift and extensional basin dominated landscape pattern in eastern China (Fig. 1). The tectonic deformation system has undergone a major transformation since the Late Jurassic. The tectonic movement evolved from the strong intracontinental compressional orogenesis and crustal thickening before the Late Jurassic to the strong intracontinental rifting and lithospheric thinning after the early Cretaceous due to the Pacific plate subducting under the Asian continent^10–12^. Although some arguments remain regarding the timing of the strong extensional deformation and rifting in eastern China, some progress has been recently made by the study of the destruction of the North China Craton. In addition, an increasing number of documents now support the early Cretaceous as the main period of strong extensional deformation in eastern China^13–15^ and a wide range of rift basins and extensional basin mountain coupling systems have been formed.

Central China was mainly controlled by the middle Tethys-paleo-Pacific Ocean geodynamic domain from the Triassic to early Jurassic of the Indosinian movement. During the period of the Triassic, the Sichuan, Ordos and other large sedimentary basins formed on the margin of the ancient Asian continent due to the tectonic processes of subduction of the Tethyan oceanic crust or sub-oceanic crust under Eurasia. At the middle to late period of the Early Jurassic, central China entered a relatively stable depression stage^16^. It was uplifted and eroded and entered a stage of structural transformation at the Late Jurassic and Cretaceous due to the influence of the paleo-Pacific geodynamic domain^17^. Since the Cenozoic, the tectonic stress field in central China is situated in the NW-NE dextral extensional tectonic setting, and entire basins have been uplifted, including the Sichuan and Ordos basins^18^.

The present geomorphic pattern is characterized by the mosaic distribution of orogenic belts and basins in western China (Fig. 1). The thrust imbricated structures and thrust strike slip structures were formed since the Late Paleozoic due to the collision between the northern margin of the Tarim ancient land and the Kazakhstan and Siberia old land accretionary margins. The old sutures and faults were revived due to the northward subduction of the Yarlung Zangbo River oceanic lithosphere and the collision and consolidation between Lhasa Block and Qiangtang Block at the end of Jurassic, which resulted in the rapid uplift of orogens. Since the Paleocene, the collision between the India and Eurasian plates and the northward subduction and compression of the India plate caused large-scale crustal compression shortening, lithospheric thickening and uplift in western China^19–24^. The Qinghai-Tibet Plateau was characterized by rapid uplift during 2~25 Ma^25–27^.

Although the arguments regarding the major tectonic units in China remains controversial, in this paper, we adopted the divisions of seven tectonic units of Pan *et al*.^28^ (Fig. 1), which are the North China Craton (NCC), Yangtze Craton (YC), Tarim Craton (TC), Tianshan-XingMeng Orogenic System (TXOS), Qinling-Qilian-Kunlun Orogenic System (QQKOS), Tibetan-Sanjiang Orogenic System (TSOS) and Wuyi-Yunkai-Taiwan Orogen System (WYTOS).

### Cratonic blocks

The North China Craton (NCC) has an Archean to Paleoproterozoic crystalline basement and is overlain by Mesoproterozoic to Cenozoic sedimentary strata^8^. The NCC is comprised of the Eastern Block, Western Block and the Central Orogenic Belt (also known as the Trans-North China Orogen)^8,13^. The eastern NCC experienced two periods of destruction during the Early Cretaceous and Paleogene based on evidence from the structural geology, mantle xenoliths, magmatic petrology, magmatic geochemistry, geophysics and geothermics^29–34^. The Yangtze Craton was a carbonate platform at the early Triassic. Due to the Indosinian movement, the Craton continued to collide with the North China plate to the north, the Qiangtang plate to the west and the Indo-China plate to the southwest since the middle Triassic, which resulted in the closure of the oceanic basins’ periphery^35,36^. From late Triassic to late Cretaceous, the continental basin developed within the Yangtze Craton^37^. However, most areas of the Yangtze region were in a state of uplift in the late Cretaceous to Cenozoic^38^, and geological, geophysical and geochemical data reveal that the eastern part of the Yangtze Craton was destroyed in the Mesozoic^39,40^. The Tarim Craton entered the foreland basin stage in the Triassic. It developed an intra-continental depression from the Jurassic to the Paleogene and the recombined foreland basin from the Neogene to the Quaternary^41,42^.

### Orogenic Systems

The Tianshan–XingMeng Orogenic System (TXOS) is a large accretionary and collision orogen that was finally sutured during the Mesozoic to Cenozoic and is comprised of several island arcs, oceanic islands, seamounts, and other accretionary complexes within the Paleo-Asian Ocean^43,44^. The Qinling-Qilian-Kunlun Orogenic System (QQKOS) stretches across the continental area of China in an east-west direction. Several tectono-magmatic thermal events have laid the main structural framework for the evolutionary process of the QQKOS, in which the Triassic magmatic thermal event developed all over the QQKOS, but the Jurassic-Cretaceous magmatic thermal event only occurred in the eastern Qinling Mountain^45,46^. The Tibet-Sanjiang Orogenic System (TSOS) is the largest active orogenic belt on the globe and was produced during the onset of the India-Asia continental collision at approximately 70 Ma^47^. The Himalayan-Tibetan orogen was built upon a complex tectonic collage that was created by the sequential accretion, from north to south, of several micro-continents, flysch complexes, and island arcs onto the southern margin of Eurasia since the early Paleozoic^47,48^. The Wuyi-Yunkai-Taiwan Orogenic System (WYTOS) in southeastern China trends NE-SW and consists of the Wuyi-Yunkai orogen and the Taiwan orogen. This area is characterized by intensive and widespread Mesozoic magmatism associated with subduction of the paleo-Pacific Plate^49^. Since the Late Cretaceous, numerous NE- or NNE- trending basins formed during an extensional tectonic setting and were filled by the Late Cretaceous–Paleogene^50^.

## Fission Track Data

**Figure 2:**
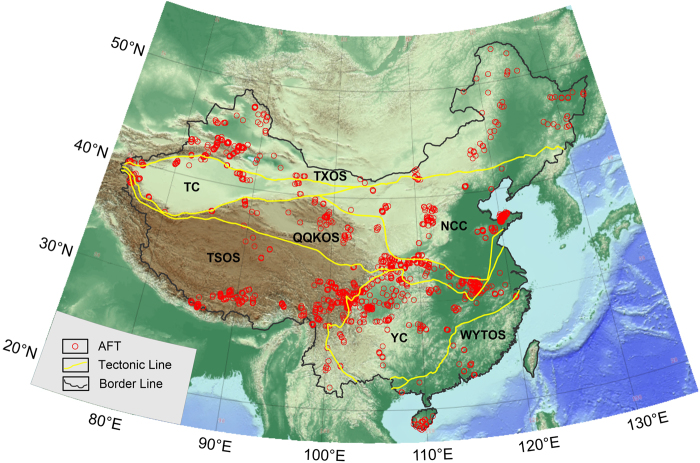
The locations of apatite fission track data of the continental area of China.

**Figure 3:**
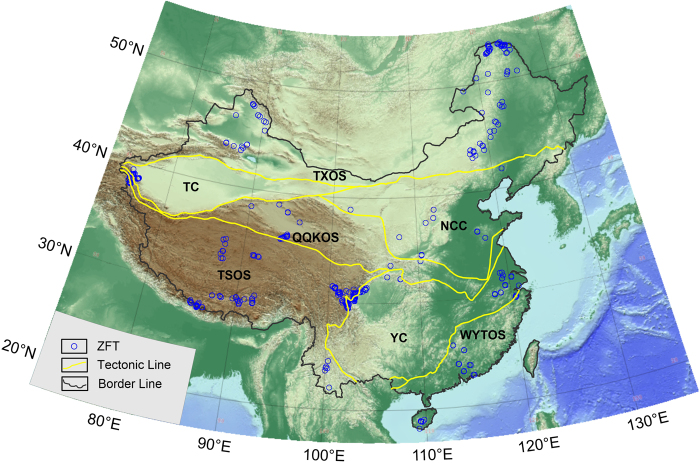
The locations of zircon fission track data of the continental area of China.

In this study, a total of 2284 published fission track data throughout mainland China were collected, of which 1847 are apatite fission track data and 437 are zircon fission track ages (see Supplementary materials 1 and 2). In addition, the 1847 apatite samples have 1294 fission track lengths and 1847 fission track ages. All the related published documents are listed in the Supplementary material 3. The samples were collected from exposed basement terranes throughout mainland China, and they are mostly granitic rocks, diorite, gabbro, andesite, tuff, rhyolite, gneisses and phyllite. In some terranes, the samples included various metamorphic and sedimentary lithologies (such as sandstone and conglomerate) where granitic lithologies were unavailable, but overall, some 80% of the studied samples are rocks of granitic composition with a relatively limited compositional range. The samples range from Precambrian to Cenozoic in age. Figures 2 and 3 show the locations of the apatite and zircon samples on a topographic map of mainland China, respectively, which shows that almost all the samples are from fold orogenic systems, such as the Qinghai-Tibetan Plateau, Tianshan, Longmenshan, Qinling, Lüliangshan and Daxinganling. Only a few samples are from sedimentary basins (e.g., the Sichuan and Ordos basins). In fact, numerous fission track data are from sedimentary basins throughout the continental area of China. The samples obtained from drilled wells in sedimentary basins may have experienced complicated burial and uplift histories, and their thermal paths may also be complicated. However, outcrop samples from exposed basement terranes or basin margins only experienced historical cooling processes; their thermal paths can reflect the uplift history and the FT ages can date the uplifting.

These fission track data were tested in different laboratories; most of the data were tested using the conventional external detector method (EDM) and a small amount of the data were tested using laser ablation inductive coupled plasma mass spectrometry (LA-ICP-MS). All seven tectonic units have different numbers of fission track samples. The apatite samples’ distribution is even in the TXOS **(**Fig. 2**)**, but the zircon samples’ locations were concentrated in Daxinganling and both sides of the Junggar basin in the eastern and western TXOS respectively **(**Fig. 3**)**. The apatite and zircon FT data in the NCC concentrate mostly in the Lüliang, Taihang, Taishan and Yanshan Mountains. The apatite FT data are relatively abundant for the Yangtze Craton (YC), but the ZFT data were obtained only in the eastern and western ends of the YC. However, there are only limited FT data from the WYTOS, most which were obtained from the middle of the WYTOS and Hainan Island. Most of the ZFT data for the TSOS were obtained in the Hengduan Mountains of the eastern TSOS and Himalaya and Lhasa blocks of the southern TSOS. The QQKOS also has relatively abundant AFT data, the distributions of which are from the Dabie, Qinling, Qilian, Kunlun and Altyn Mountains. However, a small amount of ZFT data is restricted to the Kunlun and Qilian Mountains. Regarding the Tarim Block, the AFT data are mainly distributed on the periphery of the Tarim Basin, and a small amount of ZFT data were derived only from the Eastern Pamir within the continental area of China.

## Regional Patterns of Fission Track Ages and Lengths

### Method of mapping

Interpolation and imaging of the FT data were performed with Surfer software (Version 13.0) using a Kriging gridding algorithm, in which the Block Kriging Type, None Drift Type and Linear Variogram Model (Slope = 1, Anisotropy ratio = 1.0) were adopted. To complete the interpolation for one node, 4 sectors were divided to search for available data around it and each sector at most provided 16 data samples for calculation. According to the coordinate dataset of FT samples in this paper, the Search Ellipse radius and angle were set to 2.5 and 0°. In the process of interpolation, if a minimum number of available data in all 4 sectors of one node were less than 4, that node was blanked, and if more than 2 sectors were empty, the node was also blanked. This specified Kriging algorithm excluded regions where there were no data over a large distance and made the interpolation more data-based.

### AFT ages and length

**Figure 4:**
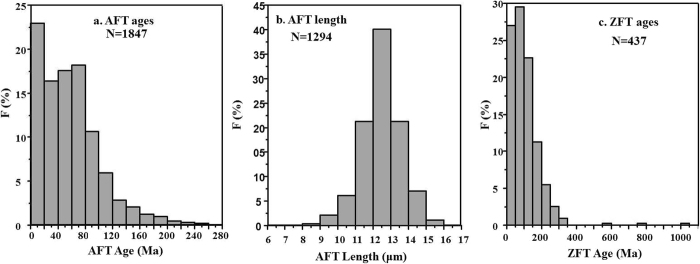
Frequency histogram of fission track parameters of the continental China. N = Data Number.

All of the 1847 AFT ages ranged between 0.20–275.40 Ma. The proportion of 0~20 Ma ages accounted for 23%, and approximately 75% of the AFTs are younger than 80 Ma; The frequency of samples older than 80 Ma is exponentially decreasing, and only 2 apatite samples are older than 250 Ma **(**Fig. 4a**)**. All of the 1294 AFT lengths are distributed within 6.51–16.15 μm. The length frequency histogram is approximately normally distributed, more than 40% of the lengths range over 12.0–13.0 μm, and approximately 83% of the lengths range over 11.0–14.0 μm **(**Fig. 4b**)**.

**Figure 5:**
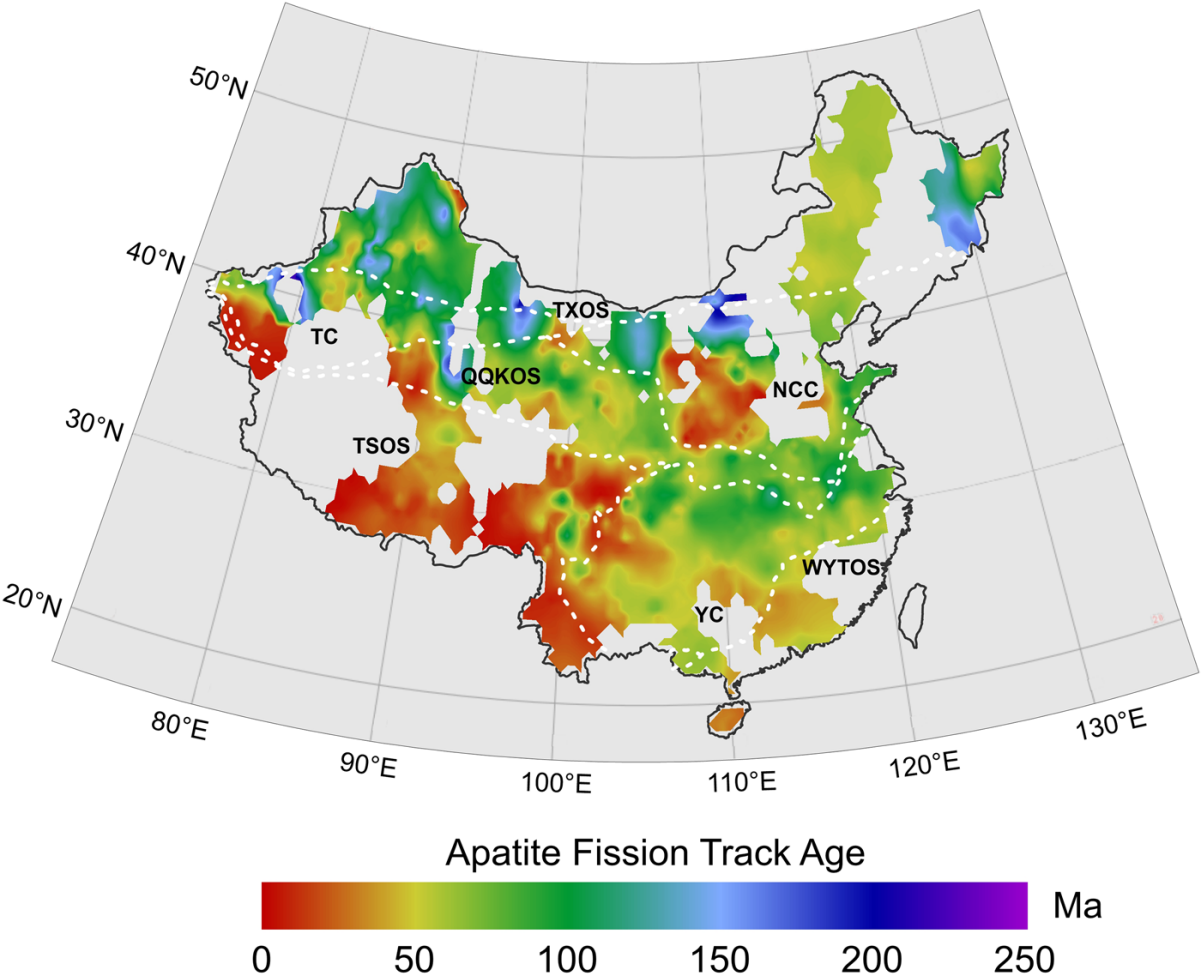
Interpolated image of apatite fission track age in continental area of China. The dashed white lines show tectonic unites (see Fig. 1. for details).

**Figure 6:**
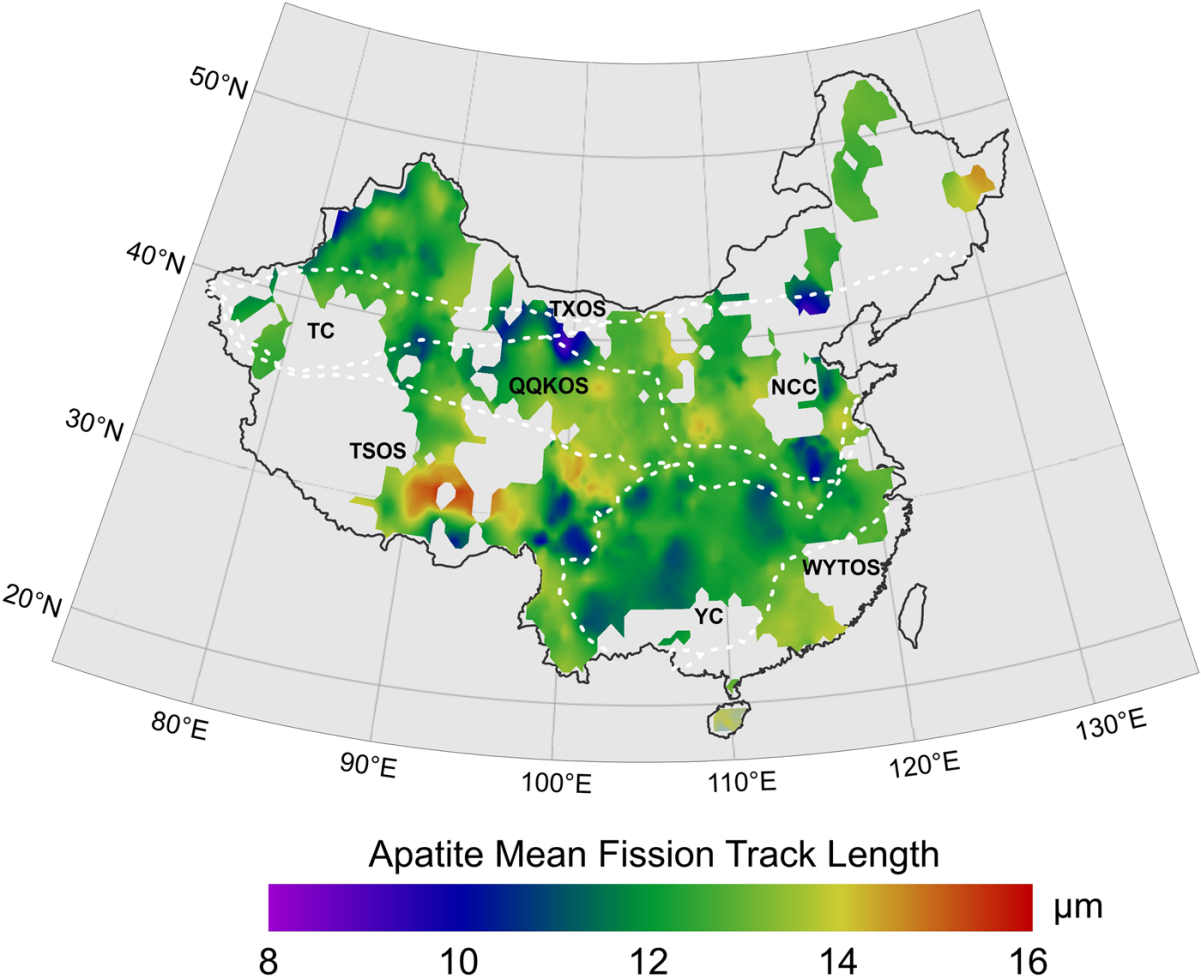
Interpolated image of apatite mean fission track length in continental area of China. The dashed white lines show tectonic unites (see Fig. 1. for details).

The interpolated images of central fission track ages and mean track lengths for apatite in the continental area of China are shown in Figs 5 and 6 respectively. Generally, the AFT ages range from approximately 0.20 Ma to just under 250 Ma and show a number of clear trends across the continent (Fig. 5). First, there is a tendency for the youngest fission track ages to be concentrated on and around the southwestern part of the continent and mountains within the NCC. For the most part, the youngest ages along this area range between 0.20 and 50 Ma. Many of the mean track lengths for apatites from these same areas are very long and often exceed 14 μm (Fig. 6), indicating that the apparent ages are actually dating the time of episodes of rapid cooling. However, the older AFT ages are mainly distributed in the northern continent, especially in western South Tianshan, the junction of the Altun and Kunlun Mountains, the Beishan tectonic belt and the Changbai Mountains, with an AFT age of ~250 Ma (Fig. 5). The AFT age and length distribution characteristics of each tectonic unit are discussed below.

#### Tibet-Sanjiang orogenic system (TSOS)

The TSOS shows the characteristics of having young AFT ages (mostly less than 30 Ma) and longer AFT lengths (>14 μm), revealing the cooling process of the rapid denudation and uplift of the Tibetan Plateau for a short time. The AFT ages decreases from the Qiangtang block to the Lhasa block, and Himalaya block (e.g., from 30 Ma to 0.5 Ma) and are closely related to the tectonic evolution of those blocks, which underwent gradual collaging under the effects of the collision and compression of the India plate in the Cenozoic. To the eastern part of the Hengduan Mountains and Songpan Ganzi Plateau, the strike slip faults and folds cause considerable variability in AFT ages of ~100 Ma and lengths of ~10 μm.

#### Yangtze craton (YC)

The western and southern regions of the Upper Yangtze show young AFT ages less than 50 Ma. The AFT ages in the northern YC range between 50–100 Ma. A new apatite fission track age of 27.9 to 61.5 Ma was obtained in the Xuefeng Mt. Range of the central YC^51^ and the result from the study by Yang *et al*.^52^ is consistent with the above ages. However, the mean track lengths in the region are of generally intermediate values (10–12 μm) that are indicative of more prolonged cooling histories.

#### Wuyi-Yunkai-Taiwan Orogenic System (WYTOS)

The AFT ages range between 25 to 50 Ma and the lengths range between 13–14 μm in this system, which indicate some prolonged cooling histories. Recently, apatite (U-Th-Sm)/He ages of 43–36 Ma have been obtained, which temporally coincides with continental rifting in the SE South China Block^53^.

#### North china craton (NCC)

The NCC developed a more than 3.8 Ga old continental crust and two large sedimentary basins, the Bohai Bay Basin in the eastern part and the Ordos Basin in the western part. The NCC should have an older AFT age, but there are also very young ages from the Taihang, Lüliang and Taishan Mountains (<30 Ma) and intermediate AFT lengths of 12–14 μm. The AFT ages reveal that these mountains provided a source area for the formation of the Cenozoic basin in Bohai Bay.

#### Qinling-qilian-kunlun orogenic system (QQKOS)

The AFT ages mostly range between 50~100 Ma from the eastern Dabie Mountains to the western Qilian Mountains. The youngest AFT ages (<30 Ma) are from the Altun Mountains of the western QQKOS, and the remaining youngest AFT age is from the central part of the QQKOS (i.e., the eastern part of the Qilian Mountains). At the same time, the lowest AFT mean length value (~10 μm) is from the eastern and western parts of the QQKOS, and the highest mean fission track length value (~14 μm) is from the central part of the QQKOS (Fig. 6). The late Cenozoic exhumation since 15 Ma has been recently revealed from apatite fission track data in the South Qinling Orogenic belt and may be attributed to the combined effect of the eastward growth of the Tibetan Plateau uplift and the Asian monsoon^54^. A younger exhumation at 65–40 Ma in the Dabie-Sulu orogenic belt was recorded by apatite fission track and apatite (U-Th)/He ages^55^. The characteristics of the AFT data may imply the different uplifts and denudations of different parts of the QQKOS.

#### Tianshan-xingmeng orogenic system (TXOS)

The AFT age characteristics in this area are generally older, with a range of 50~250 Ma, and show three significant high age distribution areas, which are the Internal Mongolian Plateau (~150–250 Ma)^56^, Beishan tectonic zone (~150–200 Ma) and Changbai Mountains (~150 Ma). The apatite samples from the Internal Mongolian Plateau and Beishan tectonic zone show intermediate AFT lengths of 12–14 μm, which shows that they have undergone more prolonged cooling histories. However, all of the AFT ages for the western TXOS are older than those of the eastern TXOS, ranging between 50~150 Ma for the Junggar basin and its periphery, and ~50 Ma for the Da Hinggan Mountains. Their AFT lengths all are between 12–14 μm, indicating that the beginning of uplift and erosion in the west was earlier than that in the east.

#### Tarim craton (TC)

The AFT ages along the Tianshan Mountains, on the northern margin of the Tarim basin, range between 25 Ma and 250 Ma and reveal the differential uplift and denudation of different segments of the Tianshan Mountains. Dumitru *et al*. revealed that the Tianshan Mountains experienced three cooling periods during uplift, exhumation and deformation by studying a series of apatite samples along the Dushanzi-Kuqa Highway, which are at the end of the Late Paleozoic, Late Mesozoic and Late Cenozoic^57^. Gillespie *et al*. also revealed the middle Triassic and late Cretaceous cooling in easternmost Tianshan using the apatite fission track and apatite (U-Th-Sm)/He data^58^. AFT ages at the end of the Late Paleozoic cooling periods formed the oldest age distribution. However, the AFT age of the Pamirs Plateau and Kunlun Mountains on the southern margin of the Tarim Basin is very young (<10 Ma). The mean track lengths in the region are of generally intermediate values (12–14 μm) indicative of more prolonged cooling histories.

### ZFT Ages

**Figure 7:**
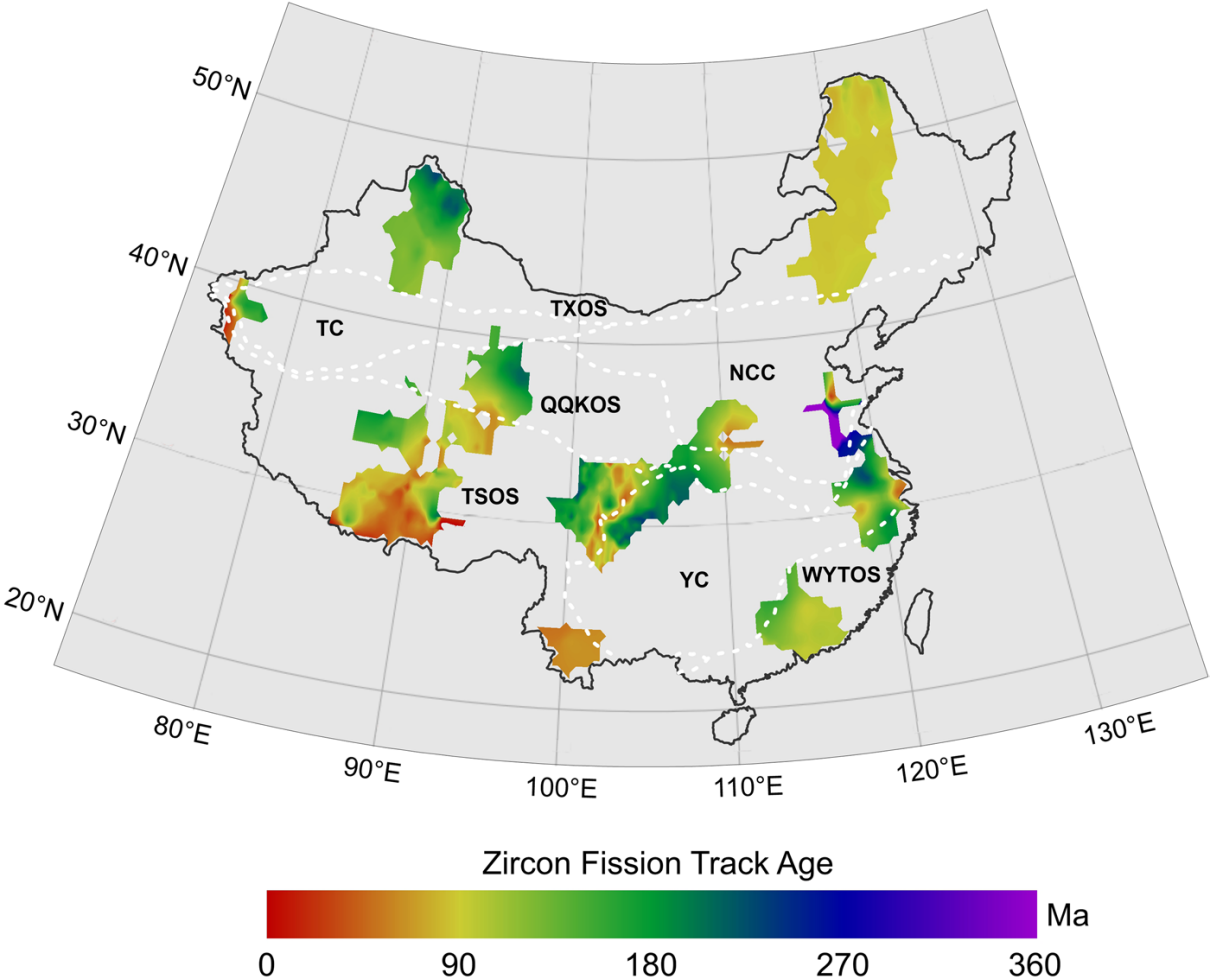
Interpolated image of zircon fission track age in continental area of China. The dashed white lines show tectonic unites (see Fig. 1. for details).

All 437 ZFT ages range between 0.70–1012 Ma. The proportion of 0~150 Ma ages accounted for 79%, and approximately 99% of the ZFTs are younger than 350 Ma (Fig. 4c); only three zircon samples are older than 350 Ma (i.e., 567 Ma, 797 Ma and 1012 Ma, and they were not included in the Kriging interpolation). The interpolated image of zircon fission track ages in the continental area of China was obtained based on the above data by applying the same Kriging interpolation method used for the AFT data (Fig. 7).

The youngest ZFT ages are distributed in the southern margin of the TSOS (<90 Ma), which indicates the late uplift orogeny. The ZFT data for the Yangtze Craton include ages that are less than 180 Ma, and all of are located on the leading edge of the Longmen Mountains in Western YC and the Lower Yangtze. All the ZFT ages from the WYTOS range between 90 to 180 Ma. Only two regions, the Taishan and northern Qinlian Mountains, have ZFT data in the NCC. The oldest ZFT age in the continental area of China, 1012 Ma, was obtained from the Taishan Mountains. Only a small number of ZFT age data that range from 50–180 Ma were obtained from the QQKOS, and are mainly distributed on the margin of the Qaidam basin. The ZFT samples from TXOS are concentrated in the Altai and Tianshan Mountains (~180 Ma) and Da Hinggan Range of the eastern TXOS (~90 Ma). Only a small amounts of ZFT data from the Tarim Block, which are concentrated in the Pamir plateau, have relatively young ages of less than 180 Ma, which correspond to the young AFT ages for this area.

## Discussion

### The effect of data on the thermotectonic images

In our study, the fission track data were obtained from different rocks and were tested in different laboratories. Numerous factors will affect the quality of fission track data and then the thermotectonic maps, including the chemical composition of apatite and the kinds of rock. It is well known that variations in chemical composition^59,60^ and other mineralogical properties^61^ can influence the fission track annealing properties of apatite. Usually, apatites with high Cl compositions are known to have greater resistance to thermal annealing than fluorapatites, and therefore require higher temperatures to produce a given degree of annealing. Apatites with high Cl compositions are typically from mafic and ultramafic igneous rocks rather than typical granitic rocks. In this study, most of the published data do not include the chemical compositions of apatites. Although it is impossible to know the chemical composition of all these samples, caution is warranted if implicating the differences in fission-track ages and lengths to the overall denudation variations. A discussion on the grouping of rock types may help to reduce the uncertainty in the future.

Numerous fission track data have been obtained from sedimentary basins throughout the continental area of China, but those fission track data from the core samples were excluded, and we mainly focused on the field outcrop samples. The core samples from drilled wells of sedimentary basins may experience complicated burial and uplift histories, and their thermal paths may also be complicated. However, outcrop samples from exposed basement terranes or basin margins only experienced cooling history processes; their thermal paths can reflect the uplift history and the FT ages can date the uplifting. That is why our study ignored regions of sedimentary basin cover. Although many sedimentary rocks from outcrops, such as sandstones, were used in our study, those samples can also reflect the tectonic uplift process. Therefore, AFT and ZFT data from sedimentary rocks do not affect the conclusions.

### Differential tectonic uplift of the continental area of China

Macroscopically, compared with other stable inland areas within the plate, the AFT ages for the continental area of China are generally young and range between 0.20~275.4 Ma. Gleadow *et al*. counted approximately 2750 AFT ages from the continent of Australia, most of which ranged between 50 and 450 Ma^6^. Even for the East Phanerozoic continental margin rift activity, the young AFT ages are between 25 to 250 Ma. This is obviously related to the plate tectonics of the two continents. The entire continent of Australia is located in the Australian plate, which is relatively stable and where there are smaller disturbances. However, the continental area of China is jointly affected by the Pacific plate and the India plate, and intracontinental tectonic deformation and fold orogeny are very common (Fig. 1). At this stage, there are numerous documents on AFT/ZFT studies, but all those studies focused on a limited research area. For this paper, the published FT data were compiled to draw broad-scale images of AFT and ZFT ages. These thermotectonic images have the advantage of allowing analyses of tectonic uplift and differences at a macroscopic scale. The AFT ages in the continental area of China can reflect important external surface processes and internal dynamic actions. The Meso-Cenozoic magmatic activity over the continent is the best proof of the internal dynamic actions.

**Figure 8:**
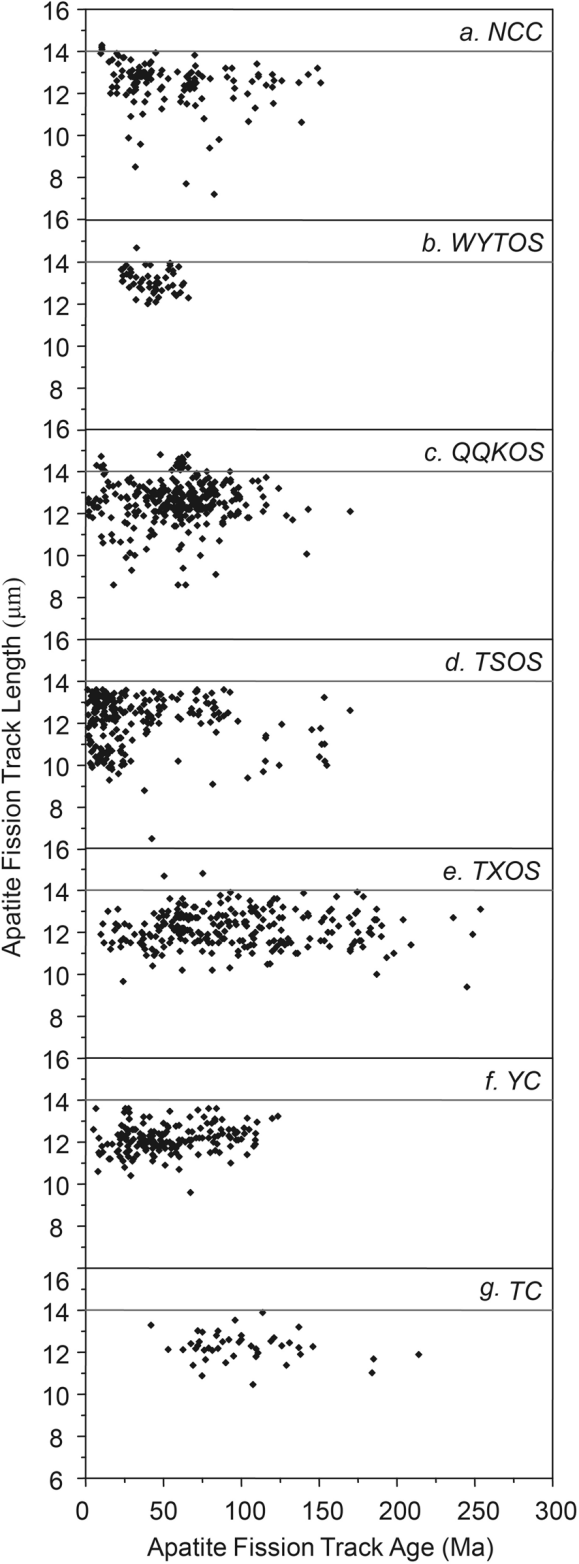
The relationship between mean apatite track length and fission track ages in each tectonic unit. A reference line is also drawn on all of the plots at the critical mean length of 14 μm, usually taken to be the minimum length characteristic of a rapid cooling event.

**Figure 9:**
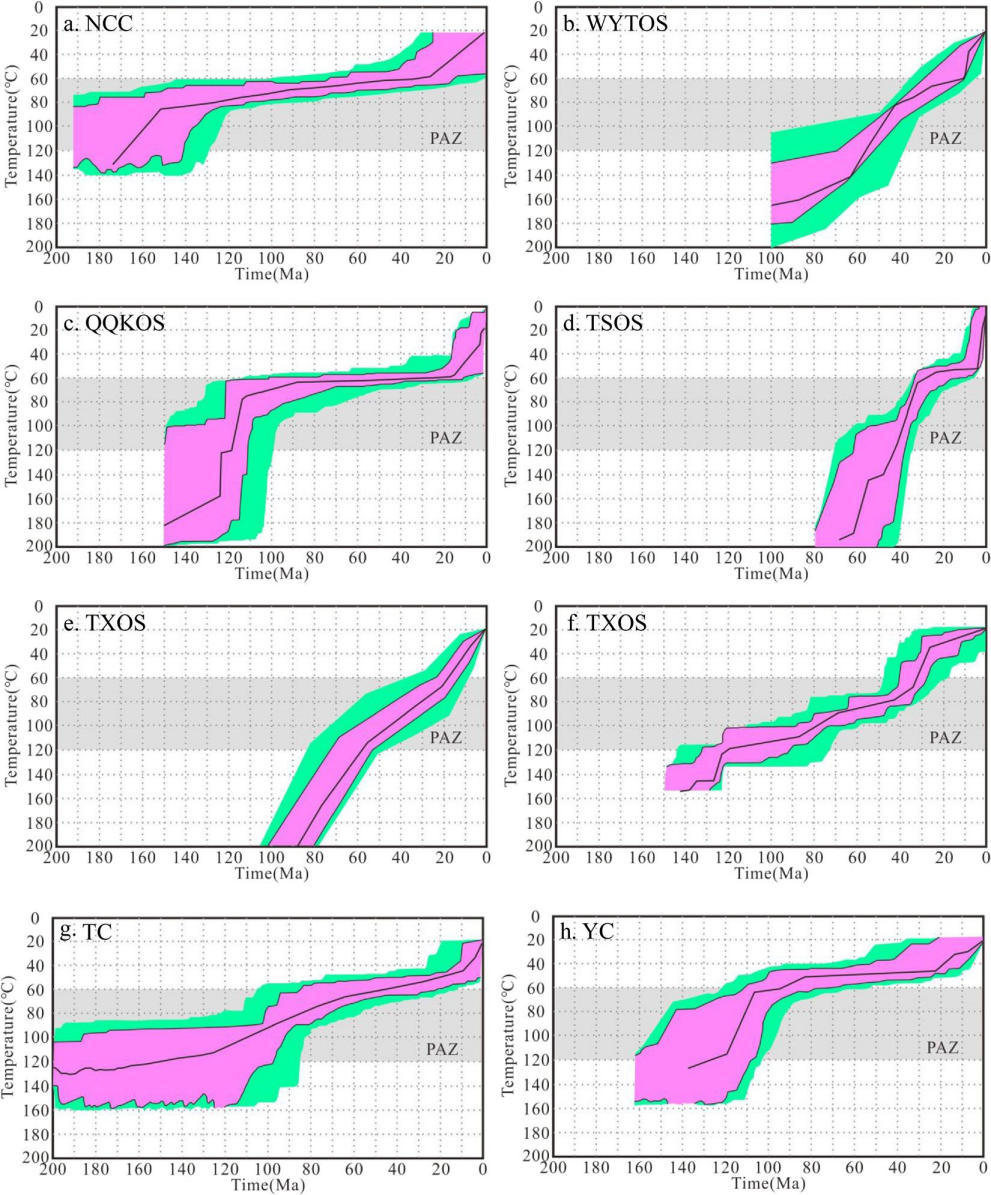
Thermal paths modeling results by fission track data of typical apatite samples in each tectonic unit. (**a**) NCC^89^; (**b**) WYTOS^90^; (**c**) QQKOS^91^; (**d**) TSOS^92^; (**e**) TXOS^93^; (**f**) TXOS^94^; (**g**) TC^95^; (**h**) YC^96^.

The relationship between track length and fission track age can yield insights into the underlying cooling trends responsible for variations within a regional fission track data set. Gleadow *et al*. divided two typical models of mean track length against fission track age^6^, e.g., boomerang and flat patterns. The boomerang trend is produced when a group of older fission track ages are simultaneously affected to differing degrees by a later rapid cooling event. Both older and younger age groups are characterized by relatively long mean track lengths and unimodal length distributions. The younger group typically has mean lengths >14 μm and the apparent age closely approximates the actual age of the cooling event. However, the flat pattern shows very minimal change in mean track length with age (typically 12.5–13.5 μm), which indicates that samples have experienced relatively slow, prolonged cooling over various time ranges. Usually, the critical mean length of 14 μm is taken as the minimum length characteristic of a rapid cooling event^62,63^. Figure 8 shows the relationships between mean apatite track length and fission track ages for each tectonic unit. Figure 9 shows the thermal paths modeling results obtained using HeFTy software and fission track data of typical apatite samples (for their locations, see Fig. 1) in each tectonic unit, which can reveal the tectonic uplift and the denudation process and denudation amount of the last stage of the tectonic unit, although the stress mechanism and uplift rate of different positions within the same tectonic unit may vary greatly. In this study, it was difficult to select a “typical” sample to show the tectonic evolution within a tectonic domain because of the wide range of mean track lengths in each tectonic unit. At the same time, the continental area of China has experienced a complicated tectonic history, and climate has introduced significant and different effects on denudation. In fact, we compared many thermal modeling results from different locations with tectonic evolution data within a tectonic unit and then selected a “good” thermal path model to show the tectonic uplift process. The sample of the “good’ thermal path modeling result is the typical sample. The detailed cooling analyses included in this study have been published in a number of regional studies (for the references, see Supplement 3).

According to the above division method, the “boomerang” type relationship is found in the NCC, WYTOS and QQKOS (the TSOS may also belong to this type) (Fig. 8a–d), among which the NCC has the typical boomerang type relationship, and the track length tended to increase with the decrease in AFT age. However, there seems to be no apparent boomerang type in the WYTOS, QQKOS and TSOS. This may result from a complicated denudation process within the tectonic unit or the numerous different published data. The AFT length for the NCC is larger than 14 μm at an age of ~10 Ma (Fig. 8a), and the thermal path of typical samples also shows that it slowly cooled before ~30 Ma, and had a tendency of rapid cooling since ~30 Ma (Fig. 9a). The AFT length for the WYTOS is larger than 14 μm at an age of ~30 Ma (Fig. 8b), and the thermal path shows that it has experienced relatively rapid continuous cooling since the Late Mesozoic (Fig. 9b). Some of the AFT lengths for the QQKOS exceed 14 μm for an age of ~75 Ma (Fig. 8c), and the thermal path also shows that it slowly cooled between 100~15 Ma, and had a tendency of rapid cooling since ~15 Ma (Fig. 9c). The thermal path in the TSOS shows three cooling stages during the Cenozoic, rapid cooling in the Paleocene and Eocene, slow cooling in the Oligocene and Miocene, and rapid cooling since the Pliocene (Fig. 9d), but its AFT length and age relationship is not that of a typical “boomerang” or “flat” type.

TXOS, YC and TC have the typical “flat” type relationship, and they all showed that there is not an obvious change in AFT length with decreasing of AFT age (Fig. 8e–g). The thermal paths in these tectonic units all show a continuous cooling process generally. The TXOS shows the continuous cooling process but with different cooling rates between the eastern and western parts (Fig. 9e,f). The Tarim Block and Yangtze Craton also experienced the continuous cooling processes since the Late Mesozoic; however, the Tarim Block shows rapid cooling after the Late Miocene (Fig. 9g), and the Yangtze Craton shows a rapid cooling process after the Miocene (Fig. 9h).

### Tectonic uplift and climatic effects

Tectonic uplift has been considered to be among the causes of continental climate change since the Cenozoic. For example, the tectonic uplift in Central Asia during the Miocene was the dominant factor of middle Miocene climatic aridity in Central Asia^64^. The collision between the India and Eurasian plates in the Cenozoic resulted in the structural deformation of the deep lithosphere and the uplift of the Qinghai Tibet Plateau. That tectonic activity has affected a series of geological processes, including the evolution of the Asian monsoon climate, atmospheric circulation in the northern hemisphere and even on a global scale, denudation and weathering of the shallow surfaces of plateaus, geomorphic differentiation, water system regulation and succession of animals and plants^65^. Climate model studies tend cite the uplift of the Tibetan Plateau as the main reason for the formation of the Asian monsoon and inland droughts^66–68^. Sun *et al*. pointed out that the present climate pattern in China^69^, in which eastern China is a monsoon region and the northwest inland basin is a westerly climate control region, had formed at least in the late Oligocene before 24 Ma. However, the climate in the continental area of China was governed by the main planetary wind system under the control of the zonal circulation before the uplift of the plateau, and the uplift of the plateau undoubtedly changed the atmospheric circulation.

Harrison *et al*. suggested that the entirety part of the Tibetan Plateau had experienced an accelerated uplift of 1000~2000 m by approximately 8 Ma, and its height had reached or exceeded the present height^70^. However, recent research on the plateaus’ uplift history and height has put forward new views. Spicer *et al*. considered that the plateau reached its present height before 15 Ma and further believed that this height has remained unchanged in the past 15 Ma^71^, and Rowley and Currie’s study suggested that the plateau surface reached a height exceeding 4000 m before 35 Ma^72^. At the same time, there are differences in the uplift times of different blocks on the Qinghai Tibet Plateau. The earliest uplift on the southwestern margin of the plateau began approximately at 50~55 Ma^73^, the tectonic deformation on the northern margin of the plateau occurred at 5~10 Ma^74^, the rapid uplift of the northeastern margin of the plateau occurred at 3.6 Ma^75^ or at 4.5 Ma^76^. From this paper, the broad-scale images of AFT and ZFT ages provide the differential tectonic uplift at a macroscopic scale and can provide new dates for the climate effects. The AFT and ZFT ages from the Qinghai Tibet Plateau decreased from north to south (e.g., the AFT ages decreased from ~50 Ma to ~0.5 Ma) **(**Figs 5 and 6). This fission track ages may reveal the timing of the uplift of the Qinghai Tibet Plateau in the Cenozoic, and, in particular, the AFT thermal paths reveals the rapid uplift since the Pliocene (Fig. 9d). Therefore, it can be inferred that the main period of the Asian monsoon and inland aridity caused by the uplift of the Tibetan Plateau began at the Pliocene, and the rapid uplift of the Tibet Plateau has changed the atmospheric circulation.

At the same time, the uplift of the Tianshan Mountains in western China has also had an impact on the climate of Central Asia. Oxygen isotope and spore pollen in Central Asian foreland basins and dust records from the Loess Plateau and the North Pacific all indicate that climate aridity in the middle Miocene (16~12 Ma) in Central Asia was significantly enhanced^77–82^. The enhanced climate aridity at 16 Ma has been attributed to the driving force of tectonic uplift in Central Asia^64^. In fact, widespread crustal shortening deformation and orogeny occurred in Central Asia during the middle Miocene. The rapid uplift of Tianshan at the Miocene has been verified by AFT ages^57,83–85^. In addition, we also revealed the Late Miocene to Pliocene rapid uplift of the southern Tianshan Mountains by the apatite (U-Th)/He ages^86^. Sun *et al*. revealed that the period of 6.5 Ma to the Pleistocene was an important period of crustal shortening and orogenic revival in the southern Tianshan Mountains via a study on the growth stratigraphy of the Kuqa foreland basin^87^. Recently, Kassner *et al*. suggested that accelerated exhumation and cooling occurred over the last ~10 Ma based on the apatite fission track and (U-Th)/He thermochronologic ages along the Ghissar-Alai Range of the southwestern Tianshan (southwestern Kyrgyzstan, northern Tajikistan) (in Fig. 2 of Kassner *et al*.^88^). Geomorphologic parameters, including incision, river steepness and concavity, confirm the youth of the southwestern Tianshan’s mountain building. The AFT ages in the eastern Pamir Plateau and Kunlun Mountain are very young and less than 10 Ma (Fig. 5 and Supplementary material 1), revealing that the Pamir Plateau began to rapidly uplift at the Late Miocene, and the thermal path of apatite samples also reveals the rapid uplift stage since the Late Miocene (Fig. 9g). Therefore, it is concluded that the main period of climate aridity in Central Asia was caused by the uplift of the Tianshan Mountains since the Miocene, and the rapid uplift during the Late Miocene to Pliocene intensified the process of aridity.

## Conclusions

This study has produced the first broad-scale coverage of the low-temperature thermochronology and a thermotectonic image of the entire continental area of China based on 2284 samples of fission track data. The study focused on the exposed basement regions and ignored regions of sedimentary basin cover. Interpolated images of fission track ages and mean track lengths have the advantage of enabling analyses of differential tectonic uplift and the climate effects on a macroscopic scale and can reveal complex patterns with significant regional variations.

Generally, the apparent AFT ages range from approximately 0.20 Ma to just under 250 Ma and show an increase trend from southwestern to eastern and northern continental area of China. The youngest AFT ages are concentrated at and around the southwestern continent and mountains within the NCC with the AFT ages between 0.20 to 50 Ma and very long AFT lengths (>14 μm), indicating the rapid cooling events. The older AFT ages are mainly distributed at the northern continent, especially in the western South Tianshan, junction of Altun and Kunlun Mountains, Beishan tectonic belt and Changbai Mountians, with the AFT age of ~250 Ma.

The continent-wide perspective of the low-temperature thermal evolution of the crust has major implications for studies of the long-term denudation history of many regions. Cooling patterns over the continental area of China revised using AFT data also reveal the different uplift/denudation processes and times between different tectonic units. The TXOS and WYTOS show patterns of prolonged slow cooling with different cooling rates, whereas other regions of the continent show discrete episodes of rapid cooling and the last rapid uplift/cooling episode primarily occurred during the Miocene and Quaternary. Significant areas of unusually young apatite ages (<10 Ma) are found in Pamir, the Qinghai-Tibetan Plateau and the Kunlun Mountains.

Tectonic uplift of the continent has been among the causes of climate change in the continent since the Cenozoic. This study provides new dating of the formation of the Asian monsoon and climate aridity in Central Asia. The uplift of the Qinghai-Tibet Plateau since the Miocene is the main cause of the formation of the Asian monsoon and inland drought, and the rapid uplift of the Tibet Plateau, which began at the Pliocene, has changed the atmospheric circulation. The main period of climate aridity in Central Asia was caused by the uplift of the Tianshan Mountains since the Oligocene, and rapid uplift during the Late Miocene to Pliocene intensified the process of aridity.

## Electronic supplementary material

Dataset 1

Dataset 2

Dataset 3
