# Supplementary material for: Uplift and denudation in the continental area of China linked to climatic effects: evidence from apatite and zircon fission track data
Source: Sci Rep. 2018 Jun 22;8:9546. doi: 10.1038/s41598-018-27801-7 (PMC6015080; doi:10.1038/s41598-018-27801-7)
Supplement: Supplementary file 1 — Dataset 1 [file 41598_2018_27801_MOESM1_ESM.pdf]

Uplift and denudation in continental area of China linked to climatic effects: evidence from apatite and zircon fission track data

Nansheng Qiu[1,2,*], Shuai Liu[1,2]

1. State Key Laboratory of Petroleum Resources and Prospecting, China University of Petroleum, Beijing, 102249, China
2. College of Geosciences, China University of Petroleum, Beijing 102249, China

Supplement 1. AFT Dataset

Apatite Fission Track Dafa of Continental China

Sample	Mineral	Latitude	Longtitude	Altitude(m)	Strata	Lithology	Mean Track Length (μ	1σ	Fission Track Age (Ma)	1σ	Reference
BX-1	Apatite	30°52'39"	102°38'19"	3519	Triassic	sandstone	11.60	0.90	25.70	1.90	Tan et al., 2014
BX-2	Apatite	30°52'20"	102°40'54"	4056	Triassic	sandstone	—	—	27.00	3.00	Tan et al., 2014
BX-3	Apatite	30°50'25"	102°42'15"	3394	Triassic	sandstone	10.90	1.70	23.50	2.00	Tan et al., 2014
BX-6	Apatite	30°48'44"	102°43'45"	2485	Triassic	sandstone	—	—	10.70	1.30	Tan et al., 2014
BX-10	Apatite	30°38'11"	102°50'29"	2203	Proterozoic	granite	—	—	1.90	1.40	Tan et al., 2014
BX-11	Apatite	30°36'11"	102°52'04"	2268	Proterozoic	granite	—	—	2.90	0.80	Tan et al., 2014
BX-13	Apatite	30°31'44"	102°54'47"	1416	Proterozoic	granite	—	—	3.80	0.80	Tan et al., 2014
BX-14	Apatite	30°26'52"	102°51'31"	1254	Proterozoic	granite	12.60	0.00	5.00	0.40	Tan et al., 2014
BX-15	Apatite	30°20'29"	102°47'32"	976	Proterozoic	granite	—	—	2.70	0.40	Tan et al., 2014
GD-1	Apatite	31°03'41"	103°24'20"	1219	Proterozoic	granite	—	—	6.30	0.70	Tan et al., 2014
GD-9	Apatite	30°54'41"	102°54'46"	4355	Triassic	sandstone	—	—	8.10	1.10	Tan et al., 2014
GD-12	Apatite	30°57'50"	102°39'02"	2728	Triassic	sandstone	—	—	11.60	1.70	Tan et al., 2014
Gong-78	Apatite	30°47'17"	102°43'45"	2513	Triassic	sandstone	—	—	16.80	1.00	Tan et al., 2014
Gong-83	Apatite	30°18'51"	102°47'08"	956	Proterozoic	granodiorite	—	—	4.40	0.40	Tan et al., 2014
Gong-90	Apatite	30°33'26"	102°59'49"	1513	Proterozoic	granite	—	—	3.90	0.40	Tan et al., 2014
Gong-92	Apatite	30°30'43"	102°54'54"	1366	Proterozoic	granite	—	—	4.00	0.50	Tan et al., 2014
Gong-94	Apatite	30°27'41"	102°52'47"	1464	Proterozoic	granite	—	—	5.00	0.50	Tan et al., 2014
Gong-95	Apatite	30°24'33"	102°50'10"	1066	Proterozoic	granite	—	—	5.30	0.50	Tan et al., 2014
JY-1	Apatite	31°51'21"	104°44'15"	584	Triassic	sandstone	9.60	1.70	67.30	4.60	Tan et al., 2014
JY-2	Apatite	31°51'53"	104°43'56"	595	Triassic	sandstone	11.60	0.30	83.30	9.10	Tan et al., 2014
Kc0511	Apatite	30°27'52"	102°52'38"	1222	Proterozoic	granite	—	—	5.40	0.70	Tan et al., 2014
WCH-1	Apatite	31°19'38"	103°23'55"	1782	Proterozoic	granite	—	—	2.10	0.20	Tan et al., 2014
PW-12	Apatite	32°23'09"	104°42'49"	1121	Silurian	sandstone	13.89	0.30	24.40	1.90	Tan et al., 2014
WCH-6	Apatite	31°03'07"	103°28'48"	884	Palaeozoic	sandstone	—	—	8.40	1.2	Tan et al., 2015
WCH-7	Apatite	31°03'40"	103°41'35"	726	Jurassic-Cretaceous	sandstone	—	—	69.10	8.2	Tan et al., 2015
JH-3	Apatite	31°21'36"	104°01'19"	888	Jurassic-Cretaceous	sandstone	—	—	7.10	0.9	Tan et al., 2015

JH-4	Apatite	31°21'22"	104°01'16"	874	Jurassic-Cretaceous	sandstone	—	—	8.00	0.9	Tan et al., 2015
JH-5	Apatite	31°24'36"	104°01'19"	1090	Jurassic-Cretaceous	sandstone	—	—	6.40	0.6	Tan et al., 2015
LK-4	Apatite	31°28'12"	104°09'14"	659	Jurassic-Cretaceous	sandstone	—	—	8.10	0.8	Tan et al., 2015
FT1	Apatite	31°03'31"	103°29'01"	957	Proterozoic	altered plagiogranite	—	—	1.20	0.3	Yang et al., 2010
FT2	Apatite	31°04'06"	103°22'34"	1322	Proterozoic	altered amphibolite	—	—	6.80	2.9	Yang et al., 2010
FT3	Apatite	31°04'21"	103°19'19"	1463	Proterozoic	altered mylonite	—	—	1.30	0.4	Yang et al., 2010
FT4	Apatite	31°06'51"	103°28'52"	970	Permian	altered diorite	—	—	0.70	0.2	Yang et al., 2010
FT6	Apatite	31°17'25"	103°27'58"	1196	Proterozoic	altered granodiorite	—	—	1.20	0.4	Yang et al., 2010
FT9	Apatite	31°57'52"	102°38'20"	3251	Triassic	diorite	—	—	0.90	0.2	Yang et al., 2010
FT10	Apatite	31°55'31"	102°39'06"	3419	Triassic	altered sandstone	—	—	2.90	1.1	Yang et al., 2010
FT11	Apatite	31°51'03"	102°40'15"	4022	Triassic	altered sandstone	—	—	2.40	0.5	Yang et al., 2010
FT12	Apatite	31°49'10"	102°41'12"	3577	Triassic	sandstone	—	—	0.50	0.2	Yang et al., 2010
FT13	Apatite	31°45'03"	102°49'39"	3517	Jurassic	granite	—	—	0.50	0.1	Yang et al., 2010
FT14	Apatite	31°37'12"	102°49'41"	2603	Triassic	altered sandstone	—	—	0.80	0.2	Yang et al., 2010
FT15	Apatite	31°24'15"	103°06'03"	2012	Triassic	slate	—	—	3.80	1.0	Yang et al., 2010
GDB17	Apatite	36°15′36.1″	101°33'07.3"	2888	Ordovician	granite	12.55	1.15	35.80	2.50	Wang et al., 2016
GDB18	Apatite	36°21′35.9″	101°25'52.5"	3852	Ordovician	granite	12.74	1.35	47.30	3.60	Wang et al., 2016
GDB19	Apatite	36°27′54.5″	101°27'19.3"	2966	Ordovician	granite	13.37	1.31	48.30	2.50	Wang et al., 2016
XN5	Apatite	36°21′47.0″	101°30'12.0"	3293	Ordovician	granite	12.53	1.62	39.50	4.90	Wang et al., 2016
XN6	Apatite	36°21'40.0"	101°26'47.0"	3800	Ordovician	granite	12.82	1.45	49.80	2.50	Wang et al., 2016
HW4	Apatite	36°31'29.4"	101°54'31.6"	2437	Ordovician	granite	13.48	1.10	92.70	5.70	Wang et al., 2016
XN1	Apatite	36°31'39.5"	101°54'23.0"	2233	Ordovician	granite	13.23	1.34	153.30	7.80	Wang et al., 2016
QW2	Apatite	35°58'0.6"	101°18'26.7"	2478	Triassic	granite	13.25	1.41	55.40	3.80	Wang et al., 2016
LHG3	Apatite	35°43'19.6"	101°20'51.9″	2990	Triassic	granite	13.62	1.26	58.10	3.00	Wang et al., 2016
GD20	Apatite	36°07′25.5″	101°35'18.2"	2222	Triassic	sandstone	13.44	1.23	81.00	5.50	Wang et al., 2016
W2	Apatite	36°59'57.4"	98°35'30.7"	3360	—	diorite	12.99	0.22	18.60	2.10	Lu et al., 2012
W3	Apatite	36°59'38.7"	98°35'31.5"	3590	—	diorite	14	0.12	19.90	1.60	Lu et al., 2012
W4	Apatite	36°59'15"	98°35'41"	3760	—	diorite	13.68	0.12	17.90	1.70	Lu et al., 2012
W5	Apatite	36°59'4.7"	98°36'2.9"	3950	—	diorite	13.65	0.14	22.20	2.20	Lu et al., 2012
APH-63	Apatite	36°21'33.78"	101°26'54.42"	3811	Ordovician	quartz diorite	13.5	0.13	70.60	7.20	Zhang et al., 2015

APH-64	Apatite	36°21'53.94'	101°26'37.62'	3778	Ordovician	quartz diorite	13.14	0.14	58.80	5.50	Zhang et al., 2015
APH-65	Apatite	36°22'2.88"	101°27'6.12"	3657	Ordovician	quartz diorite	13.16	0.12	45.90	4.50	Zhang et al., 2015
APH-66	Apatite	36°22'4.08"	101°28'6.72"	3503	Middle Proterozoic	sandstone	12.49	0.14	31.90	3.60	Zhang et al., 2015
APH-68	Apatite	36°22'32.58'	101°31'38.1"	3104	Middle Proterozoic	sandstone	13.13	0.23	16.30	3.20	Zhang et al., 2015
APH-69	Apatite	36°16'5.46"	101°57'3.78"	3121	Carboniferous	volcanics	13.6	0.15	88.80	9.60	Zhang et al., 2015
APH-70	Apatite	36°16'16.92'	101°57'53.52'	3209	Carboniferous	volcanics	13.27	0.14	77.20	8.10	Zhang et al., 2015
APH-71	Apatite	36°16'36.18'	101°58'15.48'	3245	Carboniferous	volcanics	12.56	0.25	12.10	2.70	Zhang et al., 2015
APH-62	Apatite	37°00'9.7"	102°07'28.8"	3071	Silurian	sandstone	14.07	0.15	38.40	6.10	Zhang et al., 2015
APH-63	Apatite	37°00'13.4"	102°09'26.6"	3277	Silurian	sandstone	14.67	0.1	47.30	3.10	Zhang et al., 2015
APH-64	Apatite	37°00'30.6"	102°10'33.2"	3474	Silurian	sandstone	14.24	0.13	52.90	4.70	Zhang et al., 2015
APH-65	Apatite	37°13'08.1"	101°47'10.2"	3775	Silurian	sandstone	14.59	0.14	32.90	2.20	Zhang et al., 2015
APH-66	Apatite	37°12'46.1"	101°47' 5.9"	3586	Silurian	sandstone	14.34	0.15	49.00	3.40	Zhang et al., 2015
APH-67	Apatite	37°11'10.5"	101°46'42.0"	3285	Silurian	sandstone	14.38	0.12	36.80	2.60	Zhang et al., 2015
APH-68	Apatite	37°09'46.0"	101°47'07.4"	3047	Middle Proterozoic	gneiss	14.38	0.09	60.00	4.10	Zhang et al., 2015
SG02	Apatite	31°04'14.65'	101°24'39.19'	3384	—	monzogranite	—	—	26.10	2.90	Jolivet et al., 2015
SG03	Apatite	31°04'33.56'	101°24'13.34'	3348	—	porphyritic granite	—	—	16.30	3.50	Jolivet et al., 2015
SG04	Apatite	31°04'32.68'	101°25'25.60'	3348	—	porphyritic granite	13.6	0.2	10.90	1.70	Jolivet et al., 2015
SG05	Apatite	31°04'23.71'	101°26' 01.05'	3350	—	diorite	—	—	11.40	1.50	Jolivet et al., 2015
SG06	Apatite	31°04' 16.97'	101°26' 16.81'	3300	—	leucocratic granite	—	—	12.80	2.60	Jolivet et al., 2015
SG07	Apatite	31°04' 35.90'	101°28' 25.10'	3147	—	mylonitic gneiss	13.4	0.2	8.10	0.80	Jolivet et al., 2015
SG08	Apatite	31°05'43.15'	101°31'21.10'	2867	—	gneiss mylonitic	13.8	0.2	5.70	0.80	Jolivet et al., 2015
SG09	Apatite	31°04'26.76'	101°32'21.34'	2762	—	gneiss	14	0.1	5.70	0.70	Jolivet et al., 2015
SG10	Apatite	31°02'36.86'	101°34'30.24'	2657	—	leucocratic dyke	—	—	4.50	0.60	Jolivet et al., 2015
SG12	Apatite	30° 59'17.21'	101°41'20.44'	2395	—	gneiss	—	—	5.20	1.00	Jolivet et al., 2015
SG15	Apatite	30°47'11.30'	101°44'38.55'	2211	Ordovician	paragneiss	—	—	26.00	9.80	Jolivet et al., 2015
SG17	Apatite	30°51'09.00'	101°49'25.00'	1982	—	mylonitic gneiss	—	—	8.60	2.00	Jolivet et al., 2015
SG18	Apatite	30°48'53.62'	101°56' 26.22'	1860	Silurian	paragneiss	13.3	0.2	32.70	11.80	Jolivet et al., 2015
SG19	Apatite	30°47'23.70'	101°56'48.87'	1853	—	granite	14	0.2	4.70	0.70	Jolivet et al., 2015
SG20	Apatite	30° 44'15.58'	102°01'31.05'	1800	—	granite	11.7	0.2	145.30	41.20	Jolivet et al., 2015
456	Apatite	29°06'00"	99°04'12"	2610	—	muscovite-rich coarse	12.84	0.25	9.90	1.10	Reid et al., 2005

457	Apatite	29°23'24"	99°03'36"	2615	Triassic-Jurassic	granite	12.66	0.35	5.90	0.80	Reid et al., 2005
460	Apatite	29°22'12"	99°03'36"	2630	Triassic-Jurassic	granodiorite	13.23	0.19	8.10	1.20	Reid et al., 2005
YA13	Apatite	28°26'24"	98°56'24"	3320	Triassic-Jurassic	granite	12.35	0.21	42.00	4.00	Reid et al., 2005
YA02	Apatite	29°36'00"	100°19'12"	4160	Triassic-Jurassic	granodiorite	12.35	0.2	88.70	7.30	Reid et al., 2005
YA03	Apatite	29°31'48"	100°16'48"	4490	Triassic-Jurassic	granodiorite	12.4	0.22	72.30	7.00	Reid et al., 2005
YA04	Apatite	29°31'48"	100°16'48"	4640	Triassic-Jurassic	granodiorite	12.5	0.23	85.50	10.90	Reid et al., 2005
YA05	Apatite	29°27'36"	100°11'24"	4680	Triassic-Jurassic	granodiorite	13	0.19	82.30	16.00	Reid et al., 2005
YA32	Apatite	30°15'36"	99°26'24"	3900	Cretaceous	granite	10.69	0.29	16.80	1.80	Reid et al., 2005
HF1	Apatite	33°24'53.2"	102°33'36.7"	3578	Triassic	sandstone	13.10	1.80	50.00	4.00	Deng et al., 2013b
HF2	Apatite	33°25'35.6"	102°34'48.8"	3510	Triassic	sandstone	12.70	1.90	68.00	5.00	Deng et al., 2013b
HF3	Apatite	33°27'34.0"	102°39'00.5"	3480	Triassic	sandstone	12.50	1.80	67.00	5.00	Deng et al., 2013b
HF4	Apatite	33°28'42.3"	102°39'36.6"	3506	Triassic	sandstone	12.20	1.80	61.00	4.00	Deng et al., 2013b
HF5	Apatite	33°29'25.9"	102°40'11.9"	3482	Triassic	sandstone	13.40	1.50	61.00	5.00	Deng et al., 2013b
NGL1	Apatite	29°15'12"	91°48'30"	3700	—	granodiorite	10.20	3.20	29.00	2.10	Yuan et al., 2002b
NGL2	Apatite	29°15'12"	91°48'24"	3600	—	altered andesite	11.20	2.90	29.90	2.20	Yuan et al., 2002b
NGL3	Apatite	29°15'12"	91°48'24"	3690	—	volcanic breccia	—	—	12.90	2.20	Yuan et al., 2002b
LDG1	Apatite	29°22'00"	88°24'12"	4180	—	ore	11.80	2.60	11.40	1.10	Yuan et al., 2002b
LJ1-1	Apatite	29°41'42"	91°44'54"	4850	—	skarn	12.40	2.30	18.80	1.10	Yuan et al., 2002b
LJ2-1	Apatite	29°41'42"	91°45'00"	4710	—	altered sandstone	13.20	2.20	16.10	0.90	Yuan et al., 2002b
LJ1-2	Apatite	29°41'42"	91°44'54"	4850	—	altered sandstone	11.30	2.70	22.00	4.30	Yuan et al., 2002b
LLR1	Apatite	29°48'08"	89°22'52"	4360	Eocene	volcanic breccia	—	—	46.00	12.00	Yuan et al., 2007
LLR2	Apatite	29°46'40"	89°23'04"	4320	Eocene	sandstone	13.20	2.20	5.90	0.70	Yuan et al., 2007
LLR3	Apatite	29°44'23"	89°23'05"	4300	Palaeocene	andesite	13.00	2.20	5.20	0.60	Yuan et al., 2007
LLR4	Apatite	29°44'40"	89°14'33"	4200	Eocene	granite	—	—	8.70	1.00	Yuan et al., 2007
LLR6	Apatite	29°41'05"	89°06'49"	4050	Palaeocene	andesite	12.10	2.40	19.00	3.00	Yuan et al., 2007
LY2	Apatite	29°41'18"	90°53'18"	3720	—	diorite	—	—	21.40	2.10	Yuan et al., 2001
LY9	Apatite	30°02'54"	90°35'36"	4030	—	granite	—	—	9.80	0.60	Yuan et al., 2001
LY10	Apatite	30°05'12"	90°33'06"	4250	—	granite	—	—	6.90	0.60	Yuan et al., 2001
LY14	Apatite	30°29'12"	91°09'18"	4320	—	slate	—	—	20.20	1.30	Yuan et al., 2001
LY17-1	Apatite	30°05'42"	90°32'42"	4340	—	granite	—	—	8.80	0.60	Yuan et al., 2001

LY19	Apatite	30°05'24"	90°28'30"	4250	—	granite	—	—	3.00	0.60	Yuan et al., 2001
LY15	Apatite	30°22'48"	90°55'18"	4400	—	granite	—	—	14.60	1.00	Yuan et al., 2001
LY13	Apatite	30°31'06"	91°14'42"	4390	—	slate	—	—	47.60	3.40	Yuan et al., 2001
NGL1	Apatite	29°15'12"	91°48'30"	3770	Eocene	diorite	—	—	29.00	2.10	Yuan et al., 2001
LL13	Apatite	29°19'18"	90°41'12"	3600	Upper Cretaceous	diorite	—	—	37.20	2.10	Yuan et al., 2001
NGL2	Apatite	29°19'18"	91°48'24"	3600	pper Jurassic-Lower Cretaceou	andesite	—	—	29.90	2.20	Yuan et al., 2001
LL23	Apatite	29°21'00"	90°40'48"	3600	Upper Cretaceous	diorite	—	—	21.10	1.20	Yuan et al., 2001
LLR17	Apatite	29°29'48"	89°04'48"	3940	—	diorite	—	—	16.40	0.80	Yuan et al., 2001
LLR18	Apatite	29°29'48"	89°04'30"	4250	—	granite	—	—	15.30	0.80	Yuan et al., 2001
LL14	Apatite	29°36'00"	90°59'30"	3670	pper Jurassic-Lower Cretaceou	sandstone	—	—	18.30	1.10	Yuan et al., 2001
K-7	Apatite	29°38'3.1"	102°10'4.2"	1160	—	sandstone	13.72	0.24	1.00	0.40	Tan et al., 2010
K-8	Apatite	29°13'4.0"	102°13'4.0"	1412	—	granite	13.75	0.24	1.80	0.50	Tan et al., 2010
K-9	Apatite	30°03'35.6"	102°09'43.9"	1374	—	granite	13.65	0.24	0.60	0.20	Tan et al., 2010
K-11	Apatite	30°03'25.9"	101°58'33.1"	2473	—	granite	14.08	0.33	1.60	0.50	Tan et al., 2010
K-12	Apatite	29°34'1.6"	101°58'37.6"	3190	—	granite	13.64	0.25	0.40	0.20	Tan et al., 2010
K-13	Apatite	29°34'5.9"	101°58'52.6"	3216	—	granite	13.98	0.29	1.10	0.40	Tan et al., 2010
K-14	Apatite	29°34'12.9"	101°59'11.0"	3173	—	sandstone	13.86	0.27	2.70	0.70	Tan et al., 2010
K-15	Apatite	29°34'37.0"	102°00'34.0"	2903	—	granite	14.05	0.32	1.00	0.30	Tan et al., 2010
K-16	Apatite	29°34'49.6"	102°00'31.1"	2919	—	granite	14.17	0.28	0.70	0.20	Tan et al., 2010
K-17	Apatite	29°35'04.6"	102°01'43.2"	2662	—	sandstone	15.09	0.25	0.60	0.20	Tan et al., 2010
K-18	Apatite	29°36'20.5"	102°04'59.8"	1951	—	sandstone	14.60	0.27	0.20	0.10	Tan et al., 2010
K-19	Apatite	29°36'38.6"	102°06'20.2"	1807	—	sandstone	15.19	0.26	1.20	0.50	Tan et al., 2010
LL1-2	Apatite	28°59'06"	90°24'00"	4510	Upper Triassic	slate	—	—	17.20	1.60	Yuan et al., 2002a
LL5-2	Apatite	29°05'30"	90°23'36"	4470	Upper Triassic	slate	—	—	15.30	1.90	Yuan et al., 2002a
LL10	Apatite	29°11'42"	90°37'00"	4660	Upper Triassic	sandstone	—	—	13.60	1.00	Yuan et al., 2002a
LL12	Apatite	29°14'06"	90°27'54"	4060	Upper Triassic	sandstone	—	—	17.00	1.70	Yuan et al., 2002a
AD0566t1	Apatite	27°47'53"	99°49'27"	3480	Upper Triassic	sandstone	12.70	1.80	19.00	2.00	Zou et al., 2014
AD05058t1	Apatite	27°47'1.1"	100°9'34"	2167	Lower Triassic	sandstone	12.20	2.70	15.00	2.00	Zou et al., 2014
ZD0571t1	Apatite	27°46'26"	100°8'18"	2200	Lower Triassic	sandstone	12.20	1.90	43.00	5.00	Zou et al., 2014
ZD05640t3	Apatite	27°49'5.7"	100°13'53"	2120	Lower Permian	sandstone	12.10	2.20	37.00	3.00	Zou et al., 2014

PM001-18t1	Apatite	27°48'13"	99°59'21"	3660	Upper Triassic	sandstone	12.00	2.00	42.00	4.00	Zou et al., 2014
CY-3	Apatite	28°34'56"	97°06'33"	1630	—	diorite	13.10	0.30	3.44	0.52	Lei et al., 2008c
CY-4	Apatite	28°35'29"	97°09'47"	1780	—	diorite	13.50	0.60	4.13	0.69	Lei et al., 2008c
CY-5	Apatite	28°36'34"	97°17'42"	2019	—	diorite	13.10	0.30	5.09	0.82	Lei et al., 2008c
CY-6	Apatite	28°37'58"	97°25'18"	2241	—	diorite	13.30	0.90	3.61	0.58	Lei et al., 2008c
CY-7	Apatite	28°45'34"	97°28'12"	2453	—	granitegneiss	12.10	0.40	3.68	0.50	Lei et al., 2008c
CY-9	Apatite	29°00'30"	97°23'36"	2842	—	granitegneiss	—	—	4.40	1.14	Lei et al., 2008c
CY-10	Apatite	29°19'33"	97°08'02"	3877	—	granite	12.90	0.20	11.00	0.80	Lei et al., 2008c
CY-11	Apatite	29°29'06"	97°03'12"	4500	—	granite	14.40	0.10	12.20	0.70	Lei et al., 2008c
CY-12	Apatite	29°19'21"	97°02'03"	4768	—	granite	13.90	0.20	16.00	0.80	Lei et al., 2008c
2003T128	Apatite	30°12'18"	99°56'41"	4750	—	granite	10.20	2.30	23.90	2.10	Lai et al., 2006
2003T129	Apatite	30°12'31"	99°57'06"	4607	—	granite	10.00	2.00	23.20	2.30	Lai et al., 2006
2003T130	Apatite	30°12'29"	99°57'03"	4564	—	granite	9.60	2.10	21.00	2.00	Lai et al., 2006
2003T131	Apatite	30°12'29"	99°59'02"	4542	—	granite	9.90	2.30	16.50	1.60	Lai et al., 2006
2003T132	Apatite	30°12'28"	99°56'59"	4493	—	granite	9.30	1.90	15.20	1.50	Lai et al., 2006
2003T133	Apatite	30°12'23"	99°56'56"	4411	—	granite	9.90	2.40	15.50	1.70	Lai et al., 2006
2003T134	Apatite	30°12'16"	99°56'53"	4314	—	granite	10.50	2.20	14.00	1.40	Lai et al., 2006
2003T135	Apatite	30°12'11"	99°56'46"	4227	—	granite	10.70	2.10	10.50	1.10	Lai et al., 2006
2003T140	Apatite	31°54'53"	98°57'42"	5891	—	granite	10.50	2.60	25.20	2.10	Lai et al., 2006
2003T139	Apatite	31°56'11"	98°56'16"	4756	—	granite	10.60	2.10	22.90	2.00	Lai et al., 2006
2003T138	Apatite	31°56'40"	98°55'57"	4690	—	granite	9.80	2.50	16.90	1.60	Lai et al., 2006
2003T137	Apatite	31°55'32"	99°56'14"	45601	—	granite	10.10	2.60	16.80	2.00	Lai et al., 2006
2003T136	Apatite	31°56'25"	98°55'27"	4410	—	granite	10.80	2.80	15.00	1.40	Lai et al., 2006
2003T179	Apatite	31°10'38"	99°47'08"	4280	—	granite	10.00	2.60	124.30	8.60	Lai et al., 2006
2003T175	Apatite	31°12'27"	99°45'31"	4402	—	granite	11.00	2.20	151.70	10.40	Lai et al., 2006
2003T176	Apatite	31°13'19"	99°50'44"	4477	—	granite	10.40	2.40	150.20	10.20	Lai et al., 2006
2003T177	Apatite	31°15'50"	99°52'19"	4567	—	granite	10.20	2.50	153.70	10.20	Lai et al., 2006
2003T1141	Apatite	29°38'36"	100°21'06"	3845	—	granite	9.10	2.50	81.60	5.80	Lai et al., 2006
2003T1142	Apatite	29°37'55"	100°20'41"	3966	—	granite	9.40	2.30	104.10	7.10	Lai et al., 2006
2003T1143	Apatite	29°36'56"	100°19'57"	4122	—	granite	10.20	2.50	115.40	7.60	Lai et al., 2006

2003T1144	Apatite	29°32'35"	100°16'13"	4477	—	granite	11.30	2.70	115.60	4.00	Lai et al., 2006
2003T1145	Apatite	29°31'56"	100°16'59"	4488	—	granite	9.70	2.30	114.10	7.50	Lai et al., 2006
2003T1150	Apatite	29°15'20"	100°05'11"	4272	—	granite	10.00	2.30	154.90	10.60	Lai et al., 2006
2003T26	Apatite	30°32'35"	101°34'43"	3928	—	granite	10.40	2.10	6.40	0.90	Lai et al., 2006
2003T227	Apatite	30°04'28"	101°48'16"	4300	—	granite	10.10	2.70	11.80	1.40	Lai et al., 2006
2003T229	Apatite	30°02'08"	101°50'38"	3717	—	granite	10.80	2.50	9.80	1.10	Lai et al., 2006
2003T230	Apatite	29°59'40"	101°53'05"	3252	—	granite	10.50	2.70	10.60	1.20	Lai et al., 2006
2003T231	Apatite	29°59'11"	101°55'32"	2994	—	granite	10.20	2.80	8.10	0.90	Lai et al., 2006
2003T232	Apatite	29°59'28"	101°56'16"	2999	—	granite	10.40	2.60	8.50	1.30	Lai et al., 2006
2003T207	Apatite	28°33'46"	101°46'40"	1490	—	granite	10.60	2.40	12.80	1.30	Lai et al., 2006
2003T208	Apatite	28°22'06"	101°44'59"	1489	—	granite	9.90	2.40	14.90	1.60	Lai et al., 2006
2003T212	Apatite	28°38'35"	101°38'37"	1998	—	granite	10.30	1.80	13.20	1.80	Lai et al., 2006
2003T213	Apatite	28°52'28"	101°34'09"	2596	—	granite	10.70	2.30	16.00	2.00	Lai et al., 2006
BJ11	Apatite	36°26'44"	89°30'59"	5000	—	—	—	—	0.50	0.20	Jolivet et al., 2003
11HYJ06-1	Apatite	41°48'48"	96°09'13"	—	Upper Permian	sandstone	11.10	0.69	170.00	20.00	Tian et al., 2016
11HYJ06-2	Apatite	41°48'49"	96°08'47"	—	Upper Permian	sandstone	12.60	0.67	204.00	16.00	Tian et al., 2016
11HYJ06-3	Apatite	41°48'46"	96°08' 21"	—	Upper Permian	sandstone	12.00	0.52	175.00	12.00	Tian et al., 2016
11HYJ06-4	Apatite	41°48' 48"	96°07'3"	—	Upper Permian	sandstone	12.60	0.70	187.00	19.00	Tian et al., 2016
11HYJ06-5	Apatite	41°48' 44"	96°07'22"	—	Upper Permian	sandstone	12.20	0.56	183.00	12.00	Tian et al., 2016
11HYJ06-6	Apatite	41°48'42"	96°07'01"	—	Upper Permian	sandstone	12.60	0.45	186.00	10.00	Tian et al., 2016
T3	Apatite	41°48'06"	95°59'10"	—	Upper Permian	sandstone	9.40	0.69	245.00	28.00	Tian et al., 2016
T4	Apatite	41°48'21"	95°59'37"	—	Upper Permian	sandstone	10.50	0.96	118.50	8.20	Tian et al., 2016
T5	Apatite	41°48'17"	95°58'55"	—	Upper Permian	sandstone	11.56	0.60	173.00	13.00	Tian et al., 2016
T6	Apatite	41°48'20"	95°59'04"	—	Upper Permian	sandstone	11.80	0.57	190.00	14.00	Tian et al., 2016
T7	Apatite	41°48'09"	95°59'12"	—	Upper Permian	sandstone	10.00	0.55	187.00	13.00	Tian et al., 2016
T8	Apatite	41°48' 29"	95° 58'48"	—	Upper Permian	sandstone	11.60	0.71	177.00	11.00	Tian et al., 2016
T10	Apatite	41°48'19"	95°58'43"	—	Upper Permian	sandstone	11.00	0.59	198.00	13.00	Tian et al., 2016
BH01	Apatite	41°47'17"	95°07'53"	1803	Upper Palaeozoic-Mesozoic	granite/mylonite	—	—	118.40	9.30	Gillespie et al., 2017
BH02	Apatite	41°06'30"	95°30'55"	1809	Silurian	diorite/pink granite	—	—	132.00	11.00	Gillespie et al., 2017
BH03	Apatite	40°59'17"	95°02'20"	1737	Ordovician-Silurian	granodiorite	—	—	109.30	4.30	Gillespie et al., 2017

BH04	Apatite	40°59'10"	95°02'20"	1740	Ordovician-Silurian	augen gneiss	—	—	125.00	10.00	Gillespie et al., 2017
BH05	Apatite	40°07'07"	95°43'17"	1498	Upper Palaeozoic	granodiorite vein	—	—	156.00	11.00	Gillespie et al., 2017
BH06	Apatite	40°07'05"	95°43'19"	1538	Upper Palaeozoic	pinkish granite vein	—	—	141.40	9.90	Gillespie et al., 2017
BH07	Apatite	40°06'22"	95°51'54"	1616	Upper Palaeozoic	granulite	—	—	167.50	9.80	Gillespie et al., 2017
BH08	Apatite	41°06'11"	95°24'42"	1799	Silurian	granite-granodiorite	—	—	108.10	8.00	Gillespie et al., 2017
BH09	Apatite	41°04'08"	95°16'21"	1802	Lower Palaeozoic	coarse diorite	—	—	139.80	8.70	Gillespie et al., 2017
BH10	Apatite	41°16'54"	95°21'34"	1522	Mesozoic	granite	—	—	150.00	13.00	Gillespie et al., 2017
BH12	Apatite	41°47'45"	95°08'21"	1808	Upper Palaeozoic-Mesozoic	granite	—	—	114.40	7.30	Gillespie et al., 2017
BH13	Apatite	41°48'27"	95°08'51"	1832	Upper Palaeozoic-Mesozoic	granite	—	—	141.00	13.00	Gillespie et al., 2017
BH14	Apatite	41°48'48"	95°07'05"	1833	Upper Palaeozoic-Mesozoic	granite	—	—	140.00	10.00	Gillespie et al., 2017
BH15	Apatite	41°49'01"	94°58'01"	1590	Upper Palaeozoic	diorite	—	—	102.90	2.30	Gillespie et al., 2017
BH16	Apatite	41°49'01"	94°58'01"	1590	Upper Palaeozoic	granodiorite	—	—	111.00	13.00	Gillespie et al., 2017
257	Apatite	47°42'00"	88°28'00"	821	—	instrusive/metamorphic rock	12.70	2.00	68.30	4.40	Zhao et al., 2013
258	Apatite	47°42'00"	88°26'00"	781	—	instrusive/metamorphic rock	13.60	2.00	54.10	6.40	Zhao et al., 2013
259	Apatite	47°40'00"	88°26'00"	738	—	instrusive/metamorphic rock	13.60	1.60	62.20	4.60	Zhao et al., 2013
260	Apatite	47°39'00"	88°26'00"	817	—	instrusive/metamorphic rock	12.90	2.00	61.40	3.10	Zhao et al., 2013
261	Apatite	47°38'00"	88°25'00"	839	—	instrusive/metamorphic rock	12.70	1.60	70.60	4.50	Zhao et al., 2013
262	Apatite	47°36'00"	88°25'00"	778	—	instrusive/metamorphic rock	12.50	2.50	55.60	4.70	Zhao et al., 2013
263	Apatite	47°35'00"	88°23'00"	703	—	instrusive/metamorphic rock	13.70	1.50	98.10	5.00	Zhao et al., 2013
291	Apatite	47°42'00"	88°30'00"	950	—	instrusive/metamorphic rock	12.00	2.50	62.90	8.20	Zhao et al., 2013
293	Apatite	47°40'00"	88°30'00"	835	—	instrusive/metamorphic rock	12.70	2.10	71.00	3.50	Zhao et al., 2013
299	Apatite	47°34'00"	88°26'00"	775	—	instrusive/metamorphic rock	13.40	2.00	86.70	3.20	Zhao et al., 2013
300	Apatite	47°36'00"	88°27'00"	853	—	instrusive/metamorphic rock	13.20	1.60	69.40	2.70	Zhao et al., 2013
303	Apatite	47°40'00"	88°23'00"	762	—	instrusive/metamorphic rock	13.00	1.70	106.30	5.30	Zhao et al., 2013
304	Apatite	47°38'00"	88°28'00"	919	—	instrusive/metamorphic rock	12.80	2.00	87.90	4.00	Zhao et al., 2013
306	Apatite	47°39'00"	88°29'00"	963	—	instrusive/metamorphic rock	12.30	1.80	85.60	3.40	Zhao et al., 2013
QH-1	Apatite	46°36'10"	90°15'39"	1345	Ordovician	instrusive/metamorphic rock	12.40	0.70	19.30	17.00	Xu et al., 2015
QH-9	Apatite	46°53'20"	90°18'58"	1499	Ordovician	granite	13.10	1.40	20.70	2.00	Xu et al., 2015
QH-11	Apatite	46°56'38"	90°16'23"	1555	Ordovician	granite	11.60	1.20	22.70	2.20	Xu et al., 2015
QH-15	Apatite	46°35'00"	90°14'42"	1268	Ordovician	granite	12.30	1.70	21.00	2.40	Xu et al., 2015

WQG-14	Apatite	47°11'26"	89°47'41"	1230	Ordovician	granitegeniss	12.10	1.70	18.70	1.60	Xu et al., 2015
HL07-01	Apatite	39°16'26"	106°39'39"	1338	Archaeozoic	gneiss	14.03	0.32	12.30	1.70	Liu JH et al., 2010
HL07-02	Apatite	39°16'28"	106°39'41"	1338	Archaeozoic	gneiss	13.87	0.43	11.80	1.40	Liu JH et al., 2010
HL07-03a	Apatite	39°16'29"	106°39'42"	1329	Archaeozoic	gneiss	13.98	0.21	12.70	1.70	Liu JH et al., 2010
HL07-05	Apatite	39°05'45"	106°25'19"	1171	Carboniferous	sandstone	14.72	0.55	10.00	1.40	Liu JH et al., 2010
HL07-06	Apatite	39°07'02"	106°24'07"	1248	Carboniferous	sandstone	14.3	0.45	11.40	0.90	Liu JH et al., 2010
HL07-08	Apatite	39°10'25"	106°22'37"	1334	Carboniferous	sandstone	11.3	0.82	30.90	3.50	Liu JH et al., 2010
HL07-09	Apatite	39°11'39"	106°21'02"	1381	Carboniferous	sandstone	12.8	1.06	26.20	1.70	Liu JH et al., 2010
HL07-11	Apatite	39°17'21"	106°18'56"	1519	Permian	sandstone	12.08	0.37	31.40	5.00	Liu JH et al., 2010
HL07-13	Apatite	39°18'06"	106°18'55"	1524	Archaeozoic	migmatite	13.82	1.84	71.90	9.60	Liu JH et al., 2010
HL07-14	Apatite	39°19'23"	106°18'54"	1458	Archaeozoic	migmatite	13.47	0.56	89.00	7.00	Liu JH et al., 2010
HL07-16	Apatite	38°47'09"	105°53'19"	2930	Permian	sandstone	13.55	3	107.20	5.80	Liu JH et al., 2010
HL07-17	Apatite	38°47'05"	105°53'14"	2794	Permian	sandstone	—	—	112.50	29.30	Liu JH et al., 2010
HL07-18	Apatite	38°46'55"	105°53'56"	2570	Permian	sandstone	13.35	2.11	88.10	6.60	Liu JH et al., 2010
HL07-20	Apatite	38°46'26"	105°55'04"	2087	Permian	sandstone	12.73	1.24	77.50	4.60	Liu JH et al., 2010
HL07-21	Apatite	38°45'31"	105°56'13"	1820	Permian	sandstone	13.49	2.04	73.50	4.00	Liu JH et al., 2010
HL07-23	Apatite	38°42'57"	105°59'07"	1439	Triassic	sandstone	11.43	1.34	71.70	3.60	Liu JH et al., 2010
HL07-24	Apatite	38°51'57"	105°53'21"	2244	Triassic	sandstone	13.55	1.9	110.80	6.80	Liu JH et al., 2010
HL07-25	Apatite	38°51'11"	105°51'06"	2095	Permian	sandstone	13.72	0.66	116.00	6.10	Liu JH et al., 2010
NJ3T	Apatite	32°37'12"	106°49'30"	1650	Proterozoic	diorite	13.14	0.12	103.80	5.10	Tian et al., 2012
NJ2T	Apatite	32°36'36"	106°49'48"	1588	Proterozoic	diorite	13.23	0.14	123.50	6.00	Tian et al., 2012
NJ1T	Apatite	32°36'58"	106°49'41"	1554	Proterozoic	diorite	13.13	0.1	119.80	5.90	Tian et al., 2012
NJ5T	Apatite	32°35'42"	106°50'28"	1268	Proterozoic	diorite	11.4	0.25	103.70	4.20	Tian et al., 2012
NJ6T	Apatite	32°35'28"	106°50'28"	1286	Pre-Cambrian	—	12.95	0.12	110.10	5.30	Tian et al., 2012
NJ8T	Apatite	32°34'26"	106°50'42"	1259	Pre-Cambrian	siltstone	—	—	93.90	4.10	Tian et al., 2012
NJ12T	Apatite	32°28'26"	106°52'44"	636	Lower Ordovician	sandstone	12.25	0.17	82.90	7.80	Tian et al., 2012
NJ14T	Apatite	32°27'18"	106°53'20"	575	Silurian	sandstone	—	—	60.80	5.70	Tian et al., 2012
NJ15T	Apatite	32°25'30"	106°51'50"	506	Upper Triassic	sandstone	12.66	0.2	73.80	3.70	Tian et al., 2012
NJ17T	Apatite	32°22'19"	106°51'11"	557	Jurassic	sandstone	12.49	0.16	68.40	3.40	Tian et al., 2012
DBS417	Apatite	34°06'16.5"	109°34'09.2"	1301	Cretaceous	granite	12.9	2.20	32.00	3.00	Chen et al., 2015

DBS418	Apatite	34°06'57.6"	109°33'35.6"	1242	Cretaceous	granite	13.4	2.00	21.00	2.00	Chen et al., 2015
DBS419	Apatite	34°07'27.0"	109°33'07.1"	1196	Cretaceous	granite	13.4	1.60	31.00	2.00	Chen et al., 2015
DBS420	Apatite	34°08'12.8"	109°32'44.4"	1133	Cretaceous	granite	13.60	1.90	27.00	2.00	Chen et al., 2015
DBS421	Apatite	34°08'32.3"	109°32'31.0"	1080	Cretaceous	granite	13.70	1.50	29.00	3.00	Chen et al., 2015
DBS422	Apatite	34°08'59.0"	109°32'10.5"	990	Cretaceous	granite	13.00	1.90	34.00	3.00	Chen et al., 2015
DBS484	Apatite	34°14'50.0"	106°56'07.8"	1521	Triassic	granite	13.10	1.90	53.00	4.00	Chen et al., 2015
DBS485	Apatite	34°15'28.4"	106°57'42.8"	1114	Triassic	granite	13.00	1.70	59.00	4.00	Chen et al., 2015
DBS487	Apatite	34°15'43.1"	106°58'14.0"	1066	Triassic	granite	13.10	1.90	48.00	3.00	Chen et al., 2015
DBS488	Apatite	34°16'07.6"	106°59'28.8"	939	Triassic	granite	13.00	2.00	68.00	5.00	Chen et al., 2015
DBS490	Apatite	34°16'25.9"	107°01'12.8"	832	Triassic	granite	12.20	1.80	77.00	5.00	Chen et al., 2015
DBS491	Apatite	34°16'37.0"	107°02'22.9"	767	Triassic	granite	12.40	1.80	50.00	4.00	Chen et al., 2015
DBS492	Apatite	34°17'56.6"	107°04'15.9"	686	Triassic	granite	12.30	1.70	57.00	4.00	Chen et al., 2015
DBS433	Apatite	33°47'33.3"	108°51'15.7"	1571	Triassic	granite	12.70	2.10	27.00	3.00	Chen et al., 2015
DBS430	Apatite	33°48'24.1"	108°56'29.0"	1177	Triassic	granite	13.50	1.70	41.00	3.00	Chen et al., 2015
DBS428	Apatite	33°48'20.8"	108°58' 07.7"	1083	Triassic	granite	13.10	1.70	44.00	3.00	Chen et al., 2015
DBS465	Apatite	34°01'43.8"	105°57'52.1"	1524	Triassic	granite	12.50	1.50	71.00	4.00	Chen et al., 2015
DBS466	Apatite	34°01'35.1"	105°57'54.1"	1455	Triassic	granite	12.40	1.80	74.00	4.00	Chen et al., 2015
DBS467	Apatite	34°01'15.6"	105°58'17.4"	1382	Triassic	granite	11.80	1.80	89.00	6.00	Chen et al., 2015
DBS468	Apatite	34°00'23.6"	105°59'13.4"	1308	Triassic	granite	12.20	1.60	67.00	4.00	Chen et al., 2015
DBS469	Apatite	33°58'34.2"	105°59'50.2"	1248	Triassic	granite	12.60	1.60	82.00	5.00	Chen et al., 2015
DBS470	Apatite	33°56'58.0"	106°00'15.1"	1210	Triassic	granite	12.20	1.60	57.00	4.00	Chen et al., 2015
DBS483	Apatite	33°41'36.2"	106°48'05.0"	1613	Triassic	granite	13.70	1.90	79.00	6.00	Chen et al., 2015
DBS481	Apatite	33°41'28.3"	106°48'35.0"	1484	Triassic	granite	12.90	1.70	66.00	4.00	Chen et al., 2015
DBS479	Apatite	33°41'15.6"	106°49'15.5"	1378	Triassic	granite	13.00	1.70	81.00	6.00	Chen et al., 2015
DBS478	Apatite	33°41'03.4"	106°49'40.9"	1287	Triassic	granite	12.30	1.90	90.00	6.00	Chen et al., 2015
DBS476	Apatite	33°41'31.8"	106°51'13.2"	1196	Triassic	granite	12.70	1.90	67.00	4.00	Chen et al., 2015
DBS474	Apatite	33°37'19.1"	106°54'50.8"	970	Triassic	granite	12.80	1.60	99.00	7.00	Chen et al., 2015
DBS472	Apatite	33°34'50.6"	106°57'11.2"	867	Triassic	granite	13.00	1.70	67.00	4.00	Chen et al., 2015
DBS455	Apatite	33°43'14.7"	107°57'57.3"	1688	Triassic	granite	12.80	2.30	48.00	4.00	Chen et al., 2015
DBS454	Apatite	33°42'52.4"	107°57'49.1"	1579	Triassic	granite	12.70	1.80	59.00	4.00	Chen et al., 2015

DBS452	Apatite	33°42'02.9"	107°56'57.8"	1437	Triassic	granite	12.40	1.70	68.00	4.00	Chen et al., 2015
DBS451	Apatite	33°41'32.0"	107°56'57.5"	1358	Triassic	granite	12.60	2.10	53.00	3.00	Chen et al., 2015
DBS449	Apatite	33°39'41.9"	107°58'06.4"	1224	Triassic	granite	12.30	2.00	65.00	4.00	Chen et al., 2015
DBS446	Apatite	33°37'04.4"	107°58'22.6"	1074	Triassic	granite	13.30	1.80	81.00	5.00	Chen et al., 2015
DBS444	Apatite	33°35'19.3"	107°58'48.4"	963	Triassic	granite	13.00	1.90	78.00	4.00	Chen et al., 2015
DBS441	Apatite	33°31'47.5"	107°59'15.3"	851	Triassic	granite	12.50	2.00	60.00	4.00	Chen et al., 2015
DBS323	Apatite	33°21'24.1"	108°26'47.6"	1248	Triassic	granite	12.80	1.80	40.00	3.00	Chen et al., 2015
DBS322	Apatite	33°20'51.9"	108°26'14.4"	1193	Triassic	granite	12.40	1.60	40.00	4.00	Chen et al., 2015
DBS320	Apatite	33°20'07.0"	108°26'02.8"	1123	Triassic	granite	13.40	1.70	80.00	6.00	Chen et al., 2015
DBS319	Apatite	33°19'17.3"	108°25'49.5"	1074	Triassic	granite	12.40	2.00	80.00	5.00	Chen et al., 2015
DBS316	Apatite	33°18'46.8"	108°24'18.0"	999	Triassic	granite	12.30	1.70	51.00	3.00	Chen et al., 2015
DBS314	Apatite	33°21'25.8"	108°19'21.7"	831	Triassic	granite	12.80	1.60	63.00	5.00	Chen et al., 2015
DBS426	Apatite	33°45'38.0"	109°03'30.5"	918	Triassic	granite	12.60	1.90	47.00	3.00	Chen et al., 2015
DBS324	Apatite	33°40'04.7"	108°38'55.2"	811	Triassic	granite	12.60	2.00	39.00	2.00	Chen et al., 2015
TB07-01	Apatite	33°59'47"	107°48'19"	3480	iddle Triassic-Lower Cretaceo	granite	13.58	0.2	48.30	2.30	Liu et al., 2013
TB07-02	Apatite	34°00'00"	107°48'27"	3288	iddle Triassic-Lower Cretaceo	granite	13.03	0.13	47.90	2.10	Liu et al., 2013
TB07-03	Apatite	34°00'22"	107°48'37"	3075	iddle Triassic-Lower Cretaceo	granite	12.77	0.6	42.20	2.20	Liu et al., 2013
TB07-04	Apatite	34°00'48"	107°48'34"	2801	iddle Triassic-Lower Cretaceo	granite	12.76	0.22	38.60	2.20	Liu et al., 2013
TB07-05	Apatite	34°01'07"	107°48'31"	2690	iddle Triassic-Lower Cretaceo	granite	11.89	0.37	37.20	2.30	Liu et al., 2013
TB07-06	Apatite	34°01'00"	107°48'15"	2500	iddle Triassic-Lower Cretaceo	granite	13.28	0.18	34.60	2.10	Liu et al., 2013
TB07-07	Apatite	34°01'00"	107°47'57"	2309	iddle Triassic-Lower Cretaceo	granite	12.83	0.29	32.10	1.80	Liu et al., 2013
TB07-08	Apatite	34°01'33"	107°47'16"	2105	iddle Triassic-Lower Cretaceo	granite	12.39	0.31	32.00	1.90	Liu et al., 2013
TB07-09	Apatite	34°02'33"	107°47'40"	1832	iddle Triassic-Lower Cretaceo	granite	12.35	0.24	28.10	1.60	Liu et al., 2013
TB07-10	Apatite	34°03'11"	107°47'03"	1622	iddle Triassic-Lower Cretaceo	granite	12.29	0.27	26.60	1.30	Liu et al., 2013
TB07-11	Apatite	34°03'42"	107°47'01"	1512	iddle Triassic-Lower Cretaceo	granite	10.73	0.6	28.70	1.60	Liu et al., 2013
TB07-12	Apatite	34°03'49"	107°46'16"	1470	iddle Triassic-Lower Cretaceo	granite	11.58	0.31	25.10	1.30	Liu et al., 2013
TB07-13	Apatite	34°03'55"	107°45'16"	1284	iddle Triassic-Lower Cretaceo	granite	12.76	0.43	22.70	1.20	Liu et al., 2013
TB07-14	Apatite	34°04'32"	107°44'47"	1160	iddle Triassic-Lower Cretaceo	granite	12.93	0.11	19.00	1.20	Liu et al., 2013
TB07-15	Apatite	34°05'30"	107°44'56"	1080	iddle Triassic-Lower Cretaceo	granite	14.21	0.17	9.60	0.80	Liu et al., 2013
TB07-16	Apatite	34°06'28"	107°44'38"	990	iddle Triassic-Lower Cretaceo	granite	14.29	0.14	7.00	0.90	Liu et al., 2013

HS07-01	Apatite	34°28'49"	110°04'52"	2037	iddle Triassic-Lower Cretaceo	granite	12.89	0.26	47.90	3.80	Liu et al., 2013
HS07-02	Apatite	34°29'00"	110°04'54"	1850	iddle Triassic-Lower Cretaceo	granite	12.67	0.18	42.00	3.40	Liu et al., 2013
HS07-03	Apatite	34°29'18"	110°04'55"	1655	iddle Triassic-Lower Cretaceo	granite	13.25	0.11	34.50	4.20	Liu et al., 2013
HS07-04	Apatite	34°29'27"	110°04'47"	1480	iddle Triassic-Lower Cretaceo	granite	12.55	0.15	33.10	3.60	Liu et al., 2013
HS07-05	Apatite	34°29'27"	110°04'43"	1320	iddle Triassic-Lower Cretaceo	granite	12.49	0.63	31.50	2.80	Liu et al., 2013
HS07-06	Apatite	34°29'38"	110°04'31"	1050	iddle Triassic-Lower Cretaceo	granite	12.13	0.71	28.30	2.50	Liu et al., 2013
HS07-07	Apatite	34°30'30"	110°04'35"	830	iddle Triassic-Lower Cretaceo	granite	13.08	0.57	23.50	2.80	Liu et al., 2013
HS07-08	Apatite	34°31'20"	110°04'41"	470	iddle Triassic-Lower Cretaceo	granite	14.29	0.19	10.40	1.50	Liu et al., 2013
HS08-01	Apatite	34°29'45"	110°05'32"	820	iddle Triassic-Lower Cretaceo	granite	13.4	0.21	20.90	2.10	Liu et al., 2013
HS08-02	Apatite	34°30'16"	110°05'48"	710	iddle Triassic-Lower Cretaceo	granite	13.6	0.11	17.10	1.60	Liu et al., 2013
HS08-03	Apatite	34°30'44"	110°05'54"	600	iddle Triassic-Lower Cretaceo	granite	14.1	0.12	10.50	1.20	Liu et al., 2013
HS08-04	Apatite	34°31'09"	110°05'58"	500	iddle Triassic-Lower Cretaceo	granite	14.2	0.16	10.30	1.20	Liu et al., 2013
HS08-05	Apatite	34°31'36"	110°05'56"	445	iddle Triassic-Lower Cretaceo	granite	13.9	0.21	9.70	1.30	Liu et al., 2013
QL07-01	Apatite	34°01'58"	108°48'32"	630	iddle Triassic-Lower Cretaceo	granite	14.16	0.25	10.20	0.90	Liu et al., 2013
QL07-02	Apatite	33°55'03"	108°51'00"	1093	iddle Triassic-Lower Cretaceo	granodiorite	12.95	0.13	33.10	2.40	Liu et al., 2013
QL07-03	Apatite	33°48'04"	108°47'00"	1796	iddle Triassic-Lower Cretaceo	gneiss	13.27	0.17	37.00	2.20	Liu et al., 2013
QL07-04	Apatite	33°42'08"	108°40'33"	1063	iddle Triassic-Lower Cretaceo	granite	13.11	0.31	39.40	2.20	Liu et al., 2013
QL07-05	Apatite	33°27'25"	108°28'51"	1960	iddle Triassic-Lower Cretaceo	granite	12.77	0.24	67.70	3.50	Liu et al., 2013
QL07-06	Apatite	33°24'16"	108°22'39"	1108	iddle Triassic-Lower Cretaceo	granodiorite	12.07	0.16	60.60	3.50	Liu et al., 2013
QL07-07	Apatite	33°20'07"	108°18'41"	790	iddle Triassic-Lower Cretaceo	granite	12.69	0.36	61.40	4.10	Liu et al., 2013
QL07-08	Apatite	32°57'51"	107°38'14"	464	iddle Triassic-Lower Cretaceo	gneiss	12.97	0.11	61.70	4.00	Liu et al., 2013
QL07-09	Apatite	33°02'09"	107°28'53"	573	iddle Triassic-Lower Cretaceo	granodiorite	13.61	0.9	93.10	5.70	Liu et al., 2013
QL07-10	Apatite	33°06'01"	107°24'15"	562	iddle Triassic-Lower Cretaceo	granodiorite	13	0.2	95.50	5.40	Liu et al., 2013
QL07-11	Apatite	33°17'02"	106°55'51"	562	iddle Triassic-Lower Cretaceo	granite	11.88	0.33	61.60	2.90	Liu et al., 2013
QL07-12	Apatite	33°23'33"	106°59'33"	656	iddle Triassic-Lower Cretaceo	gneiss	12.22	0.19	56.90	3.70	Liu et al., 2013
QL07-13	Apatite	33°40'24"	107°00'52"	878	iddle Triassic-Lower Cretaceo	gneiss	13.33	0.16	49.70	3.00	Liu et al., 2013
QL07-14	Apatite	33°48'41"	107°05'19"	1017	iddle Triassic-Lower Cretaceo	gneiss	13.04	0.8	60.70	2.90	Liu et al., 2013
QL07-15	Apatite	34°01'00"	107°12'42"	1458	iddle Triassic-Lower Cretaceo	gneiss	13.66	0.19	55.90	3.60	Liu et al., 2013
QL07-16	Apatite	34°09'42"	107°18'40"	1857	iddle Triassic-Lower Cretaceo	granite	13.25	0.27	61.00	3.10	Liu et al., 2013
QL07-17	Apatite	34°12'18"	107°18'04"	1259	iddle Triassic-Lower Cretaceo	granite	12.1	0.14	57.60	3.00	Liu et al., 2013

QL07-18	Apatite	34°17'26"	107°19'00"	726	iddle Triassic-Lower Cretaceo	granite	12.99	0.31	42.50	2.50	Liu et al., 2013
FT01	Apatite	34°27'42.0"	110°26'18.3"	1843	Cretaceous	granite	12.90	2.00	39.00	3.00	Yu et al., 2013
FT02	Apatite	34°27'50.8"	110°26'09.7"	1685	Cretaceous	granite	12.80	1.90	36.00	3.00	Yu et al., 2013
FT03	Apatite	34°27'58.4"	110°26'08.3"	1470	Cretaceous	granite	13.00	2.00	37.00	4.00	Yu et al.， 2013
FT04	Apatite	34°28'11.4"	110°26'10.2"	1285	Cretaceous	granite	12.90	2.10	41.00	3.00	Yu et al., 2014
FT05	Apatite	34°28'37.7"	110°25'58.0"	1087	Cretaceous	granite	13.00	1.60	42.00	3.00	Yu et al.， 2014
FT06	Apatite	34°29'29.9"	110°25'47.4"	886	Cretaceous	granite	12.80	2.10	38.00	3.00	Yu et al., 2015
FT07	Apatite	34°30'11.4"	110°25'51.2"	686	Cretaceous	granite	12.70	1.80	32.00	2.00	Yu et al.， 2015
FT08	Apatite	34°30'16.2"	110°25'43.9"	632	Cretaceous	granite	13.00	1.60	38.00	3.00	Yu et al., 2016
B019-1	Apatite	37°31'00"	98°27'00"	3780	Devonian	granite	12.80	1.90	114.00	9.00	Qi et al., 2016
B315-1	Apatite	37°45'00"	99°45'00"	4025	Ordovician	tuff	13.50	1.90	93.00	10.00	Qi et al., 2016
B325-3	Apatite	37°38'00"	99°57'00"	3619	Ordovician	tuff	13.20	1.20	112.00	21.00	Qi et al., 2016
B353-1	Apatite	38°08'00"	99°03'00"	4371	Ordovician	granite	12.10	2.00	114.00	8.00	Qi et al., 2016
B354-2	Apatite	38°08'00"	99°03'00"	4351	Ordovician	granite	11.90	2.30	93.00	7.00	Qi et al., 2016
B355-1	Apatite	38°08'00"	99°03'00"	4371	Ordovician	granite	11.80	2.20	104.00	8.00	Qi et al., 2016
B405-1	Apatite	37°22'00"	99°16'00"	3498	Silurian	dacite	12.50	1.90	89.00	9.00	Qi et al., 2016
B412-1	Apatite	37°23'00"	99°14'00"	3495	Silurian	dacite	11.50	2.10	88.00	11.00	Qi et al., 2016
N445-2	Apatite	37°49'00"	98°30'00"	3935	Silurian	tuff	12.40	1.90	103.00	13.00	Qi et al., 2016
B447-1	Apatite	37°49'00"	98°30'00"	3918	Silurian	rhyolite	12.20	2.20	74.00	10.00	Qi et al., 2016
B491-1	Apatite	37°37'00"	99°31'00"	3918	Triassic	granite	13.20	1.80	124.00	11.00	Qi et al., 2016
B614-1	Apatite	38°03'00"	98°59'00"	4242	Ordovician	dacite	12.10	2.00	98.00	8.00	Qi et al., 2016
B671-2	Apatite	38°02'00"	99°12'00"	4164	Triassic	sandstone	12.80	2.30	70.00	8.00	Qi et al., 2016
B673-2	Apatite	38°02'00"	99°12'00"	4168	Triassic	sandstone	12.30	2.30	77.00	9.00	Qi et al., 2016
B1095-2	Apatite	38°09'00"	99°01'00"	4274	Ordovician	diorite	12.00	1.90	98.00	8.00	Qi et al., 2016
B030-1	Apatite	38°03'00"	99°13'00"	4049	Triassic	sandstone	13.00	1.80	50.00	3.00	Qi et al., 2016
B030-2	Apatite	38°03'00"	99°13'00"	4049	Jurassic	sandstone	12.80	2.00	69.00	4.00	Qi et al., 2016
B1217-3	Apatite	38°05'00"	99°12'00"	4042	Triassic	sandstone	12.60	2.20	68.00	4.00	Qi et al., 2016
B385-1	Apatite	38°06'00"	99°11'00"	4155	Triassic	sandstone	13.20	1.60	59.00	4.00	Qi et al., 2016
B386-4	Apatite	38°06'00"	99°11'00"	4084	Triassic	sandstone	12.20	2.20	52.00	3.00	Qi et al., 2016
B387-1	Apatite	38°06'00"	99°11'00"	4084	Triassic	sandstone	12.90	2.00	78.00	5.00	Qi et al., 2016

B039-3	Apatite	38°04'00"	99°14'00"	4019	Jurassic	sandstone	12.30	2.20	78.00	5.00	Qi et al., 2016
B077-1	Apatite	38°04'00"	99°16'00"	4013	Triassic	sandstone	13.40	1.90	66.00	5.00	Qi et al., 2016
B043-2	Apatite	38°04'00"	99°14'00"	3985	Triassic	sandstone	12.00	2.10	93.00	6.00	Qi et al., 2016
B044-1	Apatite	38°05'00"	99°15'00"	3985	Triassic	sandstone	12.60	2.00	94.00	7.00	Qi et al., 2016
B048-1	Apatite	38°05'00"	99°17'00"	3954	Triassic	sandstone	13.10	1.70	79.00	6.00	Qi et al., 2016
B074-1	Apatite	38°05'00"	99°14'00"	4079	Jurassic	sandstone	11.90	2.30	72.00	7.00	Qi et al., 2016
B645-4	Apatite	38°11'00"	99°10'00"	4427	Triassic	granite	11.90	1.50	96.00	9.00	Qi et al., 2016
B362-1	Apatite	38°17'00"	99°17'00"	3939	Ordovician	granodiorite	11.90	2.40	32.00	4.00	Qi et al., 2016
B363-1	Apatite	38°23'00"	99°28'00"	3939	Ordovician	diorite	12.60	2.00	13.00	0.00	Qi et al., 2016
B384-1	Apatite	38°18'00"	99°15'00"	4155	Ordovician	granite	12.30	2.00	49.00	5.00	Qi et al., 2016
B054-1	Apatite	38°36'00"	99°29'00"	4018	Ordovician	granite	10.30	1.80	60.00	5.00	Qi et al., 2016
05FT-04	Apatite	39°18'25"	98°41'39"	2840	Upper Devonian	granite	12.00	0.70	77.60	6.70	Wan et al., 2010
05FT-06	Apatite	39°19'04"	98°41'53"	2750	Upper Devonian	granite	11.50	0.60	88.30	8.00	Wan et al., 2010
05FT-07	Apatite	39°19'28"	98°42'08"	2636	Upper Devonian	granite	10.00	0.20	73.70	6.50	Wan et al., 2010
05FT-08	Apatite	39°19'50"	98°42'41"	2518	Upper Devonian	granite	9.40	0.40	62.60	6.00	Wan et al., 2010
05FT-09	Apatite	39°20'29"	98°43'23"	2403	Upper Devonian	granite	8.60	0.50	59.30	5.50	Wan et al., 2010
05FT-10	Apatite	39°21'36"	98°42'53"	2260	Upper Devonian	granite	9.30	0.30	29.50	3.00	Wan et al., 2010
05FT-11	Apatite	39°22'21"	98°43'35"	2080	Upper Devonian	granite	9.90	0.90	25.90	2.70	Wan et al., 2010
05FT-12	Apatite	39°23'17"	98°44'27"	1970	Upper Devonian	granite	8.60	0.70	17.90	1.70	Wan et al., 2010
1	Apatite	36°15'29"	117°06'05"	1524	Upper Archaeozoic	diorite	13.6	1.80	29.00	2.00	Li et al., 2006
2	Apatite	36°15'19"	117°06'04"	1460	Upper Archaeozoic	diorite	13	2.00	37.00	2.00	Li et al., 2006
3	Apatite	36°15'19"	117°05'53"	1448	Upper Archaeozoic	diorite	11.6	3.00	38.00	3.00	Li et al., 2006
4	Apatite	36°15'17"	117°06'00"	1370	Upper Archaeozoic	diorite	12.8	1.90	35.00	1.00	Li et al., 2006
5	Apatite	36°15'12"	117°06'01"	1290	Upper Archaeozoic	diorite	12.5	2.50	33.00	2.00	Li et al., 2006
6	Apatite	36°14'59"	117°06'11"	1050	Upper Archaeozoic	diorite	12.3	1.90	20.00	2.00	Li et al., 2006
7	Apatite	36°14'51"	117°06'19"	960	Upper Archaeozoic	diorite	12	2.30	16.00	2.00	Li et al., 2006
8	Apatite	36°14'40"	117°06'27"	940	Upper Archaeozoic	diorite	12.9	2.10	33.00	4.00	Li et al., 2006
9	Apatite	36°14'29"	117°06'32"	900	Upper Archaeozoic	diorite	12.2	2.50	48.00	3.00	Li et al., 2006
10	Apatite	36°14'08"	117°06'31"	755	Upper Archaeozoic	diorite	12.6	2.30	20.00	2.00	Li et al., 2006
11	Apatite	36°13'58"	117°06'37"	680	Upper Archaeozoic	diorite	10.9	3.00	29.00	4.00	Li et al., 2006

12	Apatite	36°13'38"	117°06'50"	560	Upper Archaeozoic	diorite	11.6	3.00	31.00	3.00	Li et al., 2006
13	Apatite	36°13'14"	117°06'59"	440	Upper Archaeozoic	diorite	12.5	2.50	39.00	4.00	Li et al., 2006
14	Apatite	36°12'49"	117°07'11"	350	Upper Archaeozoic	diorite	12	2.10	20.00	3.00	Li et al., 2006
WK16	Apatite	38°27'12"	75°59'55"	2336	Permian	granite	—	—	9.20	1.10	Cao et al., 2013
WK17	Apatite	38°28'27"	75°59'12"	2270	Carboniferous	sandstone	—	—	6.00	0.60	Cao et al., 2013
WK20	Apatite	38°33'06"	76°03'45"	2074	Jurassic	sandstone	—	—	7.00	0.90	Cao et al., 2013
WK22	Apatite	38°33'56"	76°05'35"	2009	Jurassic	sandstone	—	—	10.30	1.20	Cao et al., 2013
WK23	Apatite	38°34'24"	76°06'55"	1956	Jurassic	sandstone	—	—	8.90	1.60	Cao et al., 2013
WK24	Apatite	38°35'46"	76°08'10"	1897	Jurassic	sandstone	—	—	10.00	1.20	Cao et al., 2013
WK77	Apatite	38°46'27"	75°14'19"	2603	Devonian	sandstone	—	—	3.30	1.10	Cao et al., 2013
WK78	Apatite	38°47'03"	75°18'13"	2394	Devonian	sandstone	—	—	2.70	0.50	Cao et al., 2013
WK82	Apatite	38°53'32"	75°29'39"	1973	Carboniferous	diorite	—	—	62.00	10.00	Cao et al., 2013
WK108	Apatite	38°43'49"	75°03'15"	3567	Triassic	gneiss	—	—	2.10	0.80	Cao et al., 2013
WK100	Apatite	37°53'00"	75°23'38"	3011	Ordovician	schist	—	—	7.20	1.00	Cao et al., 2013
WK102	Apatite	37°57'25"	75°16'14"	3520	Triassic	schist	—	—	5.50	0.50	Cao et al., 2013
WK103	Apatite	38°00'55"	75°14'43"	3061	Ordovician	gneiss	—	—	8.00	1.10	Cao et al., 2013
WK105	Apatite	37°54'50"	75°12'04"	3278	Ordovician	schist	—	—	7.50	1.00	Cao et al., 2013
WK68	Apatite	38°06'30"	74°59'06"	3544	Neogene	granitoid	—	—	10.60	1.20	Cao et al., 2013
WK69	Apatite	38°07'38"	74°58'44"	3595	Neogene	granitoid	—	—	10.80	1.20	Cao et al., 2013
WK73	Apatite	38°36'33"	74°58'59"	3308	Jurassic	granitoid	—	—	8.80	0.80	Cao et al., 2013
UC01	Apatite	39°54'56"	74°24'46"	2578	Middle Jurassic	sandstone	—	—	18.50	5.20	Yang et al., 2014
UC02	Apatite	39°54'45"	74°24'24"	2572	Lower Cretaceous	sandstone	—	—	16.60	2.80	Yang et al., 2014
UC04	Apatite	39°51'59"	74°21'32"	2486	Eocene	sandstone	—	—	87.40	42.30	Yang et al., 2014
UC05	Apatite	39°51'35"	74°20'38"	2508	Palaeocene	sandstone	—	—	69.30	3.50	Yang et al., 2014
UC07	Apatite	39°48'32"	74°19'00"	2525	Eocene	sandstone	—	—	72.10	4.30	Yang et al., 2014
UC08	Apatite	39°46'21"	74°10'22"	2603	Pleistocene	sandstone	—	—	72.40	4.20	Yang et al., 2014
KA02	Apatite	39°48'14"	74°50'41"	2820	Lower Cretaceous	sandstone	—	—	76.50	3.70	Yang et al., 2014
ZKS01	Apatite	39°46'47"	74°57'24"	2538	Lower Cretaceous	sandstone	—	—	95.00	4.10	Yang et al., 2014
Mi-1	Apatite	39°51'02"	74°32'49"	2825	Eocene-Oligocene	sandstone	—	—	48.30	2.90	Yang et al., 2014
Mi-2	Apatite	39°50'46"	74°32'25"	2846	Eocene-Oligocene	sandstone	—	—	40.60	5.30	Yang et al., 2014

Mi-3	Apatite	39°50'44"	74°33'46"	2806	Palaeocene	sandstone	—	—	39.90	2.60	Yang et al., 2014
Mi-4	Apatite	39°50'35"	74°33'28"	2804	Palaeocene	sandstone	—	—	47.10	7.80	Yang et al., 2014
Mi-5	Apatite	39°50'23"	74°32'44"	2803	Palaeocene	sandstone	—	—	42.60	3.20	Yang et al., 2014
Mi-6	Apatite	39°50'08"	74°32'50"	2772	Palaeocene	sandstone	—	—	37.20	2.40	Yang et al., 2014
Mi-7	Apatite	39°49'58"	74°33'18"	2765	Palaeocene	sandstone	—	—	45.60	3.00	Yang et al., 2014
Mi-8	Apatite	39°49'37"	74°33'08"	2735	Palaeocene	sandstone	—	—	53.50	5.30	Yang et al., 2014
TK-09	Apatite	40°05'57.18'	75°47'07.29"	2508	Carboniferous	quartz sandstone	10.20	2.30	81.30	4.70	Jia et al., 2015
TK-10	Apatite	40°04'52.10'	75°47'31.47"	2471	Carboniferous	quartz sandstone	10.80	1.70	193.40	8.20	Jia et al., 2015
TK-12	Apatite	40°03'27.80'	75°47'36.57"	2406	Carboniferous	quartz sandstone	—	—	28.80	2.50	Jia et al., 2015
TK-13	Apatite	40°02'38.40'	75°47'06.00"	2374	Carboniferous	quartz sandstone	—	—	25.70	3.00	Jia et al., 2015
SWTS-01	Apatite	39°54'07.66'	75°56'21.69"	1921	Palaeocene	sandstone	—	—	111.10	12.60	Jia et al., 2015
SWTS-02	Apatite	39°54'06.37'	75°56'22.07"	1906	Palaeocene	sandstone	—	—	112.10	17.90	Jia et al., 2015
SWTS-04	Apatite	39°50'54.34'	75°56'37.77"	1771	Cretaceous	sandstone	—	—	205.10	9.20	Jia et al., 2015
WY-02	Apatite	39°50'14.46'	76°04'13.97"	1598	Carboniferous	sandstone	—	—	68.10	8.50	Jia et al., 2015
WY-03	Apatite	39°55'19.22'	76°04'29.67"	1720	Carboniferous	sandstone	—	—	115.70	11.90	Jia et al., 2015
KKTM-04	Apatite	39°49'55.62'	76°01'40.53"	1587	—	sandstone	—	—	96.20	10.80	Jia et al., 2015
KKTM-05	Apatite	39°49'48.72'	76°03'37.81"	1581	—	sandstone	—	—	109.20	11.40	Jia et al., 2015
KKTM-08	Apatite	39°50'36.63'	75°56'48.16"	1785	—	sandstone	—	—	30.10	3.10	Jia et al., 2015
BY-03-12	Apatite	43°04'37"	84°01'42"	2638	Lower-Middle Jurassic	sandstone	12.00	1.90	72.50	4.10	Guo et al., 2006
DK43-1	Apatite	43°29'39"	84°26'13"	3145	Lower-Middle Jurassic	sandstone	—	—	37.30	40.20	Guo et al., 2006
DK39-1	Apatite	43°30'51"	84°26'47"	2903	Carboniferous	sandstone	11.60	0.80	73.40	11.60	Guo et al., 2006
DK35-1	Apatite	43°44'36"	84°25'18"	3236	Palaeozoic	granite	13.00	1.90	14.30	1.40	Guo et al., 2006
DK33-1	Apatite	43°46'40"	84°27'18"	2766	Palaeozoic	granite	11.30	1.60	50.20	6.80	Guo et al., 2006
DK25-1	Apatite	44°06'17"	84°48'29"	1733	Carboniferous	sandstone	13.10	1.40	186.90	19.10	Guo et al., 2006
DK8-2	Apatite	44°07'30"	84°23'50"	1274	Palaeozoic	granite	11.40	1.30	209.00	14.20	Guo et al., 2006
DK7-1	Apatite	44°06'40"	84°23'50"	1303	Palaeozoic	granite	12.70	1.50	236.20	21.60	Guo et al., 2006
DK6-2	Apatite	44°05'53"	84°23'37"	1408	Palaeozoic	granite	11.60	1.40	175.90	12.80	Guo et al., 2006
DK9-1	Apatite	44°08'02"	84°24'18"	1223	Upper Triassic	sandstone	11.60	1.40	153.40	11.90	Guo et al., 2006
DK19-1	Apatite	44°02'29"	84°57'04"	1751	Lower Jurassic	sandstone	11.30	1.90	178.10	14.00	Guo et al., 2006
DK18-1	Apatite	44°01'34"	84°58'00"	1634	Lower Jurassic	sandstone	11.70	1.50	144.00	8.80	Guo et al., 2006

DK10-1	Apatite	44°09'03"	84°24'32"	1206	Lower Jurassic	sandstone	12.90	1.00	173.40	13.50	Guo et al., 2006
WS-01-06	Apatite	44°09'33"	84°21'05"	1560	Lower Jurassic	sandstone	12.40	1.50	87.20	6.50	Guo et al., 2006
WS-02-17	Apatite	44°10'26"	84°20'03"	1420	Lower Jurassic	sandstone	11.90	1.80	78.40	7.60	Guo et al., 2006
WS-02-22	Apatite	44°10'26"	84°20'03"	1340	Middle Jurassic	sandstone	12.50	1.80	103.10	7.60	Guo et al., 2006
DK20-2	Apatite	44°03'36"	84°54'42"	1865	Lower Cretaceous	sandstone	13.00	1.20	174.60	15.60	Guo et al., 2006
DK20-3	Apatite	44°03'36"	84°54'42"	1865	Paleogene	sandstone	13.00	1.70	156.50	11.40	Guo et al., 2006
DK22-2	Apatite	44°18'59"	84°46'55"	728	Palaeocene	sandstone	12.70	1.50	139.80	10.60	Guo et al., 2006
DK22-3	Apatite	44°18'59"	84°46'55"	728	Palaeocene	sandstone	13.10	1.60	114.10	7.70	Guo et al., 2006
WK18-2	Apatite	43°05'27"	86°49'54"	4031	Palaeozoic	granite	13.30	1.30	118.30	11.80	Guo et al., 2006
WK19-1	Apatite	43°06'13"	86°50'13"	3822	Palaeozoic	granite	12.90	1.90	142.70	12.10	Guo et al., 2006
WK28-1	Apatite	43°17'15"	87°11'02"	2018	Lower Jurassic	sandstone	13.20	1.50	126.70	12.50	Guo et al., 2006
HX-06-03	Apatite	46°15'30.8"	87°09'07.5"	2198	Middle Jurassic	sandstone	12.00	1.90	83.70	6.90	Guo et al., 2006
HX-06-07	Apatite	43°15'30.8"	87°09'08.5"	2170	Middle Jurassic	sandstone	11.90	2.00	68.40	4.60	Guo et al., 2006
HX-10-04	Apatite	43°19'57.2"	87°07'51.9"	2193	Middle Jurassic	sandstone	11.90	1.70	74.10	4.50	Guo et al., 2006
HX-11-04	Apatite	43°19'57.3"	87°07'52.7"	2211	Middle Jurassic	sandstone	12.40	1.70	37.80	2.70	Guo et al., 2006
HX-13-06	Apatite	43°19'33.9"	87°08'34.3"	2127	Middle Jurassic	sandstone	11.90	2.00	36.90	3.00	Guo et al., 2006
HJ-02-01	Apatite	43°39'16"	87°12'54"	1213	Lower Jurassic	sandstone	11.10	1.70	60.60	6.10	Guo et al., 2006
WK35-1	Apatite	43°41'00"	87°10'24"	1114	Middle Jurassic	sandstone	13.10	1.50	253.70	16.50	Guo et al., 2006
WK37-1	Apatite	43°44'06"	87°12'58"	1039	Middle Jurassic	sandstone	12.70	1.50	153.30	10.40	Guo et al., 2006
WK38-1	Apatite	43°36'37"	87°15'28"	962	Upper Jurassic	sandstone	11.50	2.70	130.00	11.80	Guo et al., 2006
WK39-1	Apatite	43°47'19"	87°16'01"	923	Upper Jurassic	sandstone	11.60	0.60	151.00	14.30	Guo et al., 2006
WK40-1	Apatite	43°47'53"	87°16'40"	932	Lower Cretaceous	sandstone	11.50	2.00	125.40	13.20	Guo et al., 2006
WK43-1	Apatite	43°49'56"	87°18'10"	925	Oligocene	sandstone	13.40	1.40	118.60	10.60	Guo et al., 2006
WK43-2	Apatite	43°49'56"	87°18'10"	925	Palaeocene	sandstone	12.70	1.80	169.40	12.30	Guo et al., 2006
QKT6	Apatite	46°09'51"	89°32'09"	1008	Palaeozoic	granite	11.90	1.90	183.80	23.10	Guo et al., 2006
QKT13	Apatite	46°18'00"	89°10'45"	830	Palaeozoic	granite	13.70	1.30	178.40	11.00	Guo et al., 2006
QKT15	Apatite	46°18'24"	89°09'53"	808	Palaeozoic	granite	—	—	152.40	15.30	Guo et al., 2006
YBS-1	Apatite	45°37'18"	90°21'42"	1244	Palaeozoic	granite	13.20	1.80	70.40	7.40	Guo et al., 2006
XHS-1	Apatite	45°26'52"	90°26'13"	1244	Palaeozoic	granite	12.30	1.60	92.40	9.20	Guo et al., 2006
MEG3	Apatite	45°31'12"	83°51'15"	1511	Palaeozoic	granite	12.30	2.70	162.50	16.10	Guo et al., 2006

MEG10	Apatite	45°30'42"	83°53'21"	1470	Palaeozoic	granite	12.70	1.80	124.30	10.70	Guo et al., 2006
TLC1	Apatite	45°44'06"	83°32'12"	1537	Palaeozoic	granite	11.40	2.20	171.40	20.10	Guo et al., 2006
KLMY1	Apatite	45°39"44	84°44'06"	738	Palaeozoic	granite	13.70	2.00	161.20	12.00	Guo et al., 2006
KLMY6	Apatite	45°40'09"	84°42'58"	721	Palaeozoic	granite	13.10	1.90	157.90	14.80	Guo et al., 2006
KLMY7	Apatite	45°40'29"	84°43'09"	777	Palaeozoic	granite	12.60	2.10	158.60	11.80	Guo et al., 2006
KKTH1	Apatite	47°02'30"	89°46'06"	2000	Palaeozoic	granite	12.80	2.10	64.60	8.40	Guo et al., 2006
KKTH5	Apatite	47°02'33"	89°02'55"	1170	Palaeozoic	granite	13.50	1.30	105.60	6.20	Guo et al., 2006
M1	Apatite	42°58'30"	85°49'20"	—	Lower Cretaceous	sandstone	—	—	186.90	10.00	Hendrix at al.,1994
M2	Apatite	43°56'40"	85°51'50"	—	Jurassic	sandstone	—	—	97.60	9.90	Hendrix at al.,1994
M3	Apatite	43°55'10"	85°52'40"	—	Jurassic	sandstone	—	—	32.50	5.30	Hendrix at al.,1994
M4	Apatite	43°51'50"	85°49'40"	—	Jurassic	sandstone	—	—	38.10	4.00	Hendrix at al.,1994
M5	Apatite	43°51'20"	85°49'00"	—	Triassic	sandstone	—	—	29.70	5.70	Hendrix at al.,1994
MN1	Apatite	43°51'25"	85°48'48"	—	Upper Triassic	sandstone	11.60	2.10	26.00	2.00	Du et al., 2007
MN2	Apatite	43°53'00"	85°50'22"	—	Lower Jurassic	sandstone	11.80	2.40	45.00	6.00	Du et al., 2007
MN3	Apatite	43°53'59"	85°50'58"	—	Lower Jurassic	sandstone	11.20	2.10	38.00	4.00	Du et al., 2007
MN4	Apatite	43°54'33"	85°53'00"	—	Middle Jurassic	sandstone	11.50	2.50	17.00	2.00	Du et al., 2007
MN5	Apatite	43°55'28"	85°52'04"	—	Middle Jurassic	sandstone	11.40	1.90	108.00	8.00	Du et al., 2007
MN6	Apatite	43°57'17"	85°46'20"	—	Lower Cretaceous	sandstone	10.90	1.90	98.00	8.00	Du et al., 2007
MN7	Apatite	43°57'24"	85°46'21"	—	Lower Cretaceous	sandstone	11.10	1.80	75.00	7.00	Du et al., 2007
MN8	Apatite	43°57'40"	85°46'08"	—	Lower Cretaceous	sandstone	11.00	2.10	107.00	9.00	Du et al., 2007
MN9	Apatite	43°57'51"	85°46'29"	—	Lower Cretaceous	sandstone	12.20	1.90	113.00	8.00	Du et al., 2007
MN10	Apatite	43°58'53"	85°45'05"	—	Palaeocene	sandstone	11.60	2.00	64.00	5.00	Du et al., 2007
TTH1	Apatite	43°39'39"	87°12'56"	—	Upper Triassic	sandstone	12.00	2.20	109.00	11.00	Du et al., 2007
TTH2	Apatite	43°40'05"	87°13'09"	—	Lower Jurassic	sandstone	11.60	2.10	121.00	10.00	Du et al., 2007
TTH3	Apatite	43°40'11"	87°13'04"	—	Lower Jurassic	sandstone	11.60	2.30	99.00	10.00	Du et al., 2007
TTH4	Apatite	43°40'54"	87°12'34"	—	Middle Jurassic	sandstone	11.40	2.10	128.00	11.00	Du et al., 2007
TTH5	Apatite	43°41'43"	87°09'25"	—	Middle Jurassic	sandstone	12.30	2.30	46.00	3.00	Du et al., 2007
TTH6	Apatite	43°46'44"	87°15'37"	—	Upper Jurassic	sandstone	11.90	1.90	85.00	6.00	Du et al., 2007
TTH7	Apatite	43°47'31"	87°16'15"	—	Upper Jurassic	sandstone	11.60	1.50	101.00	8.00	Du et al., 2007
KC1	Apatite	41°48'52"	82°52'17"	—	Pliocene-Pleistocene	sandstone	12.10	1.80	58.00	4.00	Du et al., 2007

KC2	Apatite	41°54'27"	83°19'02"	—	Palaeocene	sandstone	12.80	1.60	50.00	26.00	Du et al., 2007
KC3	Apatite	42°07'07"	83°02'17"	—	Palaeocene	sandstone	11.70	1.50	55.00	4.00	Du et al., 2007
KC4	Apatite	42°05'51"	83°02'12"	—	Palaeocene-Eocene	sandstone	11.50	1.40	118.00	10.00	Du et al., 2007
KC5	Apatite	42°06'09"	83°08'24"	—	Lower Cretaceous	sandstone	11.00	1.40	81.00	7.00	Du et al., 2007
KC6	Apatite	42°06'37"	83°08'35"	—	Lower Cretaceous	sandstone	11.40	1.50	106.00	7.00	Du et al., 2007
KC7	Apatite	42°08'52"	83°06'19"	—	Middle Jurassic	sandstone	10.90	2.10	39.00	4.00	Du et al., 2007
KC8	Apatite	42°09'30"	83°06'38"	—	Lower Jurassic	sandstone	12.30	1.80	28.00	3.00	Du et al., 2007
KC9	Apatite	43°06'04"	83°15'23"	—	Upper Triassic	sandstone	11.90	2.10	41.00	4.00	Du et al., 2007
KC10	Apatite	42°16'04"	83°15'23"	—	Upper Triassic	sandstone	11.80	1.90	42.00	4.00	Du et al., 2007
HXG1	Apatite	43°02'06"	86°46'44"	—	—	granite	12.40	1.80	82.00	4.00	Du et al., 2007
HXG2	Apatite	43°01'58"	86°46'08"	—	—	granite	12.70	2.00	84.00	5.00	Du et al., 2007
HXG3	Apatite	42°59'37"	86°42'33"	—	—	granite	13.00	1.80	75.00	4.00	Du et al., 2007
HXG4	Apatite	42°56'18"	86°40'52"	—	—	granite	13.00	2.00	76.00	4.00	Du et al., 2007
BLT5	Apatite	42°45'01"	86°18'26"	—	—	granite	11.60	2.20	49.00	3.00	Du et al., 2007
BLT6	Apatite	42°45'21"	86°18'26"	—	—	granite	—	—	22.00	3.00	Du et al., 2007
BLT7	Apatite	42°44'02"	86°18'21"	—	—	granite	11.60	2.40	49.00	3.00	Du et al., 2007
BLT8	Apatite	42°42'59"	86°17'41"	—	—	granite	12.00	1.90	60.00	4.00	Du et al., 2007
BLT9	Apatite	42°41'45"	86°16'23"	—	—	granite	12.10	2.50	57.00	8.00	Du et al., 2007
BLT10	Apatite	42°39'56"	86°16'47"	—	—	granite	12.30	2.20	59.00	4.00	Du et al., 2007
BLT11	Apatite	42°38'57"	86°16'24"	—	—	granite	12.20	2.00	59.00	3.00	Du et al., 2007
BLT12	Apatite	42°39'37"	86°18'42"	—	—	granite	11.90	2.20	74.00	4.00	Du et al., 2007
BLT13	Apatite	42°39'57"	86°19'40"	—	—	granite	12.20	2.00	63.00	4.00	Du et al., 2007
BLT14	Apatite	42°41'35"	86°22'03"	—	—	granite	12.80	1.90	67.00	4.00	Du et al., 2007
KCH15	Apatite	42°17'41"	83°16'13"	—	—	granite	11.90	1.70	61.00	4.00	Du et al., 2007
KCH16	Apatite	42°17'43"	83°16'11"	—	—	granite	11.50	2.10	54.00	3.00	Du et al., 2007
KCH17	Apatite	43°17'59"	83°15'42"	—	—	granite	12.00	2.30	36.00	2.00	Du et al., 2007
KCH18	Apatite	42°17'23"	83°16'07"	—	—	granite	11.50	2.10	35.00	2.00	Du et al., 2007
KCH19	Apatite	42°16'58"	83°16'22"	—	—	granite	11.40	2.30	37.00	3.00	Du et al., 2007
KCH20	Apatite	42°17'22"	83°16'34"	—	—	granite	10.90	2.40	49.00	3.00	Du et al., 2007
KCH22	Apatite	42°17'51"	83°19'30"	—	—	granite	10.40	2.30	43.00	4.00	Du et al., 2007

BY23	Apatite	42°59'55"	84°07'53"	—	—	granite	12.80	2.00	111.00	8.00	Du et al., 2007
BY24	Apatite	43°00'10"	84°07'38"	—	—	granite	11.30	2.40	104.00	7.00	Du et al., 2007
BY25	Apatite	43°01'45"	84°07'20"	—	—	granite	11.50	2.50	94.00	5.00	Du et al., 2007
BY26	Apatite	43°00'49"	84°08'43"	—	—	granite	11.30	2.40	102.00	7.00	Du et al., 2007
BY27	Apatite	43°01'16"	84°10'30"	—	—	granite	11.50	2.50	70.00	4.00	Du et al., 2007
BY28	Apatite	43°01'14"	84°10'34"	—	—	granite	12.70	1.90	72.00	4.00	Du et al., 2007
BY29	Apatite	43°01'13"	84°10'39"	—	—	granite	11.70	2.40	89.00	5.00	Du et al., 2007
BY30	Apatite	43°01'12"	84°10'41"	—	—	granite	11.70	2.00	130.00	7.00	Du et al., 2007
BY31	Apatite	43°05'04"	84°10'06"	—	—	granite	11.10	2.80	98.00	7.00	Du et al., 2007
QM44	Apatite	43°41'17"	84°24'12"	—	—	granite	11.70	2.40	77.00	5.00	Du et al., 2007
QM45	Apatite	43°41'28"	84°25'14"	—	—	granite	11.60	2.20	72.00	5.00	Du et al., 2007
QM47	Apatite	43°43'03"	84°26'28"	—	—	granite	10.20	2.40	62.00	8.00	Du et al., 2007
QM48	Apatite	43°43'36"	84°25'44"	—	—	granite	12.40	2.50	25.00	2.00	Du et al., 2007
QM49	Apatite	43°44'35"	84°25'10"	—	—	granite	12.40	2.30	11.00	1.00	Du et al., 2007
QM50	Apatite	43°45'27"	84°26'33"	—	—	granite	11.20	2.40	16.00	1.00	Du et al., 2007
1	Apatite	43°51'18.1"	85°38'25.6"	2175	Middle Carboniferous	tuff	11.70	2.20	37.10	6.50	Shen et al., 2008
2	Apatite	43°51'40.1"	85°38'41.0"	2014	Lower Permian	andesite	11.90	2.00	9.80	1.30	Shen et al., 2008
3	Apatite	43°54'2.6"	85°39'47.5"	1548	Lower Permian	andesite	12.10	1.80	65.50	4.80	Shen et al., 2008
4	Apatite	43°42'31.9"	86°39'52.0"	1977	Jurassic	sandstone	11.40	1.70	36.20	3.70	Shen et al., 2008
5	Apatite	43°26'51.5"	87°40'14.5"	1425	Middle Carboniferous	tuff	—	—	68.20	10.60	Shen et al., 2008
BLT1	Apatite	42°41'7.3"	86°19'55"	2480	Upper Palaeozoic	granite	—	—	63.10	6.40	Lü et al., 2013
BLT2	Apatite	42°41'5.8"	86°20'1.2"	2386	Upper Palaeozoic	granite	12.51	1.33	92.20	8.20	Lü et al., 2013
BLT3	Apatite	42°41'4.8"	86°20'7.3"	2251	Upper Palaeozoic	granite	—	—	65.30	9.80	Lü et al., 2013
BLT4	Apatite	42°41'5.8"	86°20'14.9"	2147	Upper Palaeozoic	granite	13.37	1.23	59.90	4.30	Lü et al., 2013
BLT6	Apatite	42°40'58.8"	86°20'42.7"	1956	Upper Palaeozoic	granite	—	—	43.20	3.70	Lü et al., 2013
BLT8	Apatite	42°40'47.1"	86°21'12.2"	1796	Upper Palaeozoic	granite	—	—	63.90	5.60	Lü et al., 2013
SC-01-06	Apatite	43°54'0.5"	85°39'50"	1553	Middle-Upper Triassic	sandstone	11.60	1.80	125.30	9.10	Zhang et al., 2007
SC-03-04	Apatite	43°54'11"	85°39'57"	1503	Lower Jurassic	sandstone	11.50	2.00	85.40	5.70	Zhang et al., 2007
SC-03-10	Apatite	43°54'16"	85°39'39"	1428	Lower Jurassic	sandstone	11.70	1.70	87.80	5.90	Zhang et al., 2007
SC-04-07	Apatite	43°54'56"	85°39'32"	1428	Lower Jurassic	sandstone	11.50	1.50	81.30	4.70	Zhang et al., 2007

SC-06-03	Apatite	43°55'49"	85°44'01"	1350	Middle Jurassic	sandstone	10.90	1.90	44.00	5.40	Zhang et al., 2007
MN-09-04	Apatite	43°54'32"	85°51'35"	1120	Middle Jurassic	sandstone	11.50	2.20	11.80	1.10	Zhang et al., 2007
MN-10-03	Apatite	43°54'33"	85°52'21"	1115	Middle Jurassic	sandstone	11.30	1.50	22.50	3.20	Zhang et al., 2007
DB01	Apatite	32°16'00"	107°55'19"	—	Middle Jurassic	feldspar-rich sandstone	12.41	0.19	109.20	6.40	Xu et al., 2010
DB02	Apatite	32°16'26"	107°56'18"	—	Lower Jurassic	quartz-feldspar sandstone	12.67	0.19	101.80	6.00	Xu et al., 2010
DB03	Apatite	36°16'28"	107°56'31"	—	Upper Triassic	quartz-feldspar sandstone	13.08	0.20	87.60	5.60	Xu et al., 2010
DB-1	Apatite	32°16'58"	108°11'08"	476	Sinian	sandstone	11.40	1.60	33.00	4.00	Shen et al., 2007
DB-2	Apatite	32°17'15"	108°10'03"	485	Lower Cambrian	sandstone	12.00	1.90	40.00	5.00	Shen et al., 2007
DB-3	Apatite	32°16'58"	108°03'26"	1200	Middle Cambrian	sandstone	12.20	1.50	73.00	8.00	Shen et al., 2007
DB-4	Apatite	32°20'20"	108°00'12"	954	Sinian	sandstone	12.70	1.60	53.00	5.00	Shen et al., 2007
DB-5	Apatite	32°18'30"	108°59'11"	739	Upper Triassic	sandstone	11.80	1.70	47.00	5.00	Shen et al., 2007
DB-6	Apatite	32°17'01"	107°57'11"	692	Middle Jurassic	sandstone	12.00	1.90	44.00	3.00	Shen et al., 2007
DB-7	Apatite	32°16'10"	107°55'37"	716	Middle Jurassic	sandstone	12.10	1.50	48.00	7.00	Shen et al., 2007
DB-8	Apatite	32°15'50"	107°53'42"	649	Middle Jurassic	sandstone	12.10	1.60	43.00	6.00	Shen et al., 2007
DB-9	Apatite	32°13'32"	107°47'09"	556	Middle Jurassic	sandstone	11.90	1.70	49.00	4.00	Shen et al., 2007
DB-10	Apatite	32°11'55"	107°43'00"	540	Sinian	sandstone	—	—	69.00	13.00	Shen et al., 2007
DB-11	Apatite	32°12'32"	107°43'39"	475	Sinian	sandstone	10.80	3.10	25.00	3.00	Shen et al., 2007
DB-12	Apatite	32°02'35"	108°01'22"	626	Lower Jurassic	sandstone	11.30	2.00	44.00	7.00	Shen et al., 2007
DB-13	Apatite	31°54'54"	108°02'56"	720	Upper Triassic	sandstone	11.50	2.00	58.00	7.00	Shen et al., 2007
DB-14	Apatite	31°54'54"	108°37'11"	870	Sinian	sandstone	12.90	1.50	78.00	7.00	Shen et al., 2007
DB-15	Apatite	31°47'23"	108°35'26"	694	Upper Permian	sandstone	12.10	1.70	26.00	3.00	Shen et al., 2007
DB-16	Apatite	31°46'51"	108°32'56"	663	Lower Silurian	sandstone	12.00	1.90	49.00	5.00	Shen et al., 2007
DB-17	Apatite	31°45'51"	108°32'22"	658	Middle Silurian	sandstone	—	—	41.00	21.00	Shen et al., 2007
DB-18	Apatite	31°37'59"	108°35'42"	1130	Upper Ordovician	sandstone	12.80	1.50	60.00	7.00	Shen et al., 2007
DB-0A	Apatite	31°11'00"	116°35'42"	541	Jurassic	sandstone	11.1	1.05	66.00	8.00	Hu et al., 2006a
DB-0B	Apatite	31°13'06"	116°26'06"	512	Jurassic	sandstone	12.3	0.26	71.00	6.00	Hu et al., 2006a
DB-1B	Apatite	31°24'36"	115°56'30"	453	Palaeozoic	schist	12	0.21	60.00	5.00	Hu et al., 2006a
TB-17	Apatite	31°43'12"	115°28'36"	213	—	gneiss	12.6	0.23	71.00	4.00	Hu et al., 2006a
TB-21	Apatite	31°21'00"	116°16'06"	412	—	schist	13.2	0.22	82.00	7.00	Hu et al., 2006a
TB-11	Apatite	32°31'30"	113°40'30"	205	Palaeozoic	granodiorite	13	0.33	70.00	4.00	Hu et al., 2006a

TB-12	Apatite	32°36'18"	113°37'24"	223	Palaeozoic	granodiorite	12.2	0.15	75.00	4.00	Hu et al., 2006a
TB-13	Apatite	32°31'42"	113°20'42"	253	Palaeozoic	schist	—	—	74.00	27.00	Hu et al., 2006a
DB-2	Apatite	31°21'06"	116°10'42"	220	—	retro-eclogite	12.4	0.2	60.00	4.00	Hu et al., 2006a
DB-3	Apatite	31°21'06"	116°10'42"	220	—	gneiss	—	—	24.00	11.00	Hu et al., 2006a
DB-4	Apatite	31°20'24"	116°09'07"	448	—	gneiss	12	0.35	50.00	4.00	Hu et al., 2006a
DB-5	Apatite	31°20'18"	116°08'26"	464	—	gneiss	12.6	0.17	60.00	4.00	Hu et al., 2006a
DB-6	Apatite	31°16'30"	116°02'41"	615	Cretaceous	granite	11	0.54	44.00	5.00	Hu et al., 2006a
DB-7	Apatite	31°11'12"	116°00'17"	340	—	migmatite	11.4	0.24	57.00	3.00	Hu et al., 2006a
DB-8	Apatite	31°10'12"	116°00'08"	350	—	migmatite	12.5	0.24	52.00	6.00	Hu et al., 2006a
DB-9	Apatite	31°09'07"	116°01'46"	510	Cretaceous	granite	11.9	0.28	51.00	4.00	Hu et al., 2006a
DB-12	Apatite	31°02'20"	116°08'10"	940	Cretaceous	granite	13	0.21	57.00	5.00	Hu et al., 2006a
TB-9	Apatite	31°14'30"	116°02'00"	1103	—	gneiss	12.7	0.22	54.00	3.00	Hu et al., 2006a
TB-14	Apatite	32°15'12"	113°22'36"	612	—	gneiss	12.7	0.21	55.00	3.00	Hu et al., 2006a
TB-15	Apatite	32°15'36"	113°18'24"	252	—	mylonite	12.4	0.2	53.00	4.00	Hu et al., 2006a
TB-20	Apatite	31°24'36"	115°56'30"	437	—	gneiss	13.1	0.17	58.00	3.00	Hu et al., 2006a
TB-2	Apatite	31°15'18"	114°24'42"	349	—	mylonite	—	—	77.00	23.00	Hu et al., 2006a
TB-18	Apatite	30°39'06"	115°02'42"	209	—	mylonite	12.8	0.17	57.00	3.00	Hu et al., 2006a
DB-14	Apatite	30°44'12"	116°17'00"	200	—	eclogite	12.3	0.23	58.00	4.00	Hu et al., 2006a
DB-15	Apatite	30°44'12"	116°17'00"	200	—	eclogite	13.6	1.54	77.00	7.00	Hu et al., 2006a
DB-16	Apatite	30°44'12"	116°17'00"	200	—	gneiss	14.8	0.59	48.00	4.00	Hu et al., 2006a
DB-17	Apatite	30°42'42"	116°15'18"	320	—	quartzite	12.5	0.3	62.00	4.00	Hu et al., 2006a
DB-19	Apatite	30°41'42"	116°16'48"	500	—	granitic gneiss	—	—	62.00	8.00	Hu et al., 2006a
DB-20	Apatite	30°43'18"	116°27'12"	780	Cretaceous	granite	14.4	0.4	63.00	7.00	Hu et al., 2006a
DB-21	Apatite	30°40'24"	116°29'24"	250	—	gneiss	12.6	0.16	44.00	2.00	Hu et al., 2006a
DB-22	Apatite	30°40'24"	116°29'24"	250	—	gneiss	12.3	0.26	55.00	3.00	Hu et al., 2006a
DB-23	Apatite	30°38'06"	116°24'48"	350	—	eclogite	11.8	0.27	45.00	3.00	Hu et al., 2006a
DB-24	Apatite	30°38'00"	116°24'48"	350	—	quartzite	11.9	0.22	51.00	3.00	Hu et al., 2006a
DB-25	Apatite	30°30'54"	116°24'48"	240	—	gneiss	12.1	0.33	45.00	3.00	Hu et al., 2006a
DB-26	Apatite	30°30'54"	116°24'48"	240	—	gneiss	11.6	1.39	44.00	3.00	Hu et al., 2006a
DB-27	Apatite	30°28'54"	116°18'42"	200	Cretaceous	granodiorite	11.6	0.21	50.00	4.00	Hu et al., 2006a

DB-28	Apatite	30°28'18"	116°14'54"	240	—	eclogite	—	—	67.00	21.00	Hu et al., 2006a
DB-29	Apatite	30°28'18"	116°14'48"	240	—	gneiss	12.5	0.16	45.00	2.00	Hu et al., 2006a
DB-32	Apatite	30°27'12"	116°12'12"	260	—	gneiss	12.4	0.2	53.00	3.00	Hu et al., 2006a
DB-36	Apatite	30°34'42"	116°16'24"	390	—	gneiss	12.2	0.32	65.00	4.00	Hu et al., 2006a
DB-37	Apatite	31°01'49"	116°27'42"	1243	Cretaceous	granite	11.9	0.86	64.00	7.00	Hu et al., 2006a
DB-39	Apatite	30°24'12"	116°03'48"	105	—	gneiss	—	—	66.00	7.00	Hu et al., 2006a
DB-40	Apatite	30°42'36"	116°28'42"	891	—	mylonite	12.3	0.3	55.00	4.00	Hu et al., 2006a
DB-41	Apatite	30°42'36"	116°28'42"	892	—	mylonite	12.3	0.47	74.00	7.00	Hu et al., 2006a
TB-4	Apatite	31°33'30"	114°45'12"	315	Cretaceous	granodiorite	13.1	0.66	60.00	10.00	Hu et al., 2006a
TB-5	Apatite	31°35'48"	114°48'00"	318	Cretaceous	granite	11.7	1.1	61.00	5.00	Hu et al., 2006a
TB-7	Apatite	31°42'36"	114°53'54"	295	—	gneiss	—	—	67.00	16.00	Hu et al., 2006a
TB-8	Apatite	31°43'18"	114°46'18"	292	Cretaceous	granite	12.1	0.23	47.00	3.00	Hu et al., 2006a
TB-19	Apatite	31°43'18"	114°46'18"	292	Cretaceous	granite	12.9	0.22	45.00	3.00	Hu et al., 2006a
TB-24	Apatite	31°36'42"	114°38'54"	904	—	eclogite	—	—	73.00	21.00	Hu et al., 2006a
TB-25	Apatite	31°44'42"	114°38'12"	392	—	schist	12.8	0.33	54.00	4.00	Hu et al., 2006a
DB-39	Apatite	30°24'12"	116°03'48"	105	—	gneiss	—	—	66.00	7.00	Hu et al., 2006a
SU-1	Apatite	31°22'42"	113°21'48"	605	—	schist	10.07	1.26	142.00	36.00	Hu et al., 2006a
SU-2	Apatite	31°22'36"	113°21'54"	605	—	schist	11.7	0.71	133.00	20.00	Hu et al., 2006a
DS 012[a]	Apatite	30°27'30"	116°18'12"	—	—	gneiss	—	—	49.30	4.10	Grimmer et al., 2002
DS 025	Apatite	31°18'18"	115°56'54"	—	—	gneiss	—	—	79.70	9.70	Grimmer et al., 2002
DS 043	Apatite	31°22'24"	116°11'30"	—	—	schist	—	—	90.20	6.50	Grimmer et al., 2002
DS 058	Apatite	31°14'20"	116°20'23"	—	—	gabbro	—	—	85.80	3.10	Grimmer et al., 2002
DS 072	Apatite	31°07'29"	116°31'31"	—	—	gneiss	—	—	69.60	3.40	Grimmer et al., 2002
DS 076	Apatite	30°47'00"	116°05'00"	—	—	granite	—	—	77.30	3.10	Grimmer et al., 2002
DS 095	Apatite	30°42'28"	116°23'16"	—	—	gneiss	—	—	66.30	2.90	Grimmer et al., 2002
DS 099	Apatite	30°40'00"	116°27'00"	—	—	gneiss	—	—	55.40	2.30	Grimmer et al., 2002
DS 103	Apatite	30°40'00"	116°27'00"	—	—	gneiss	—	—	70.80	2.60	Grimmer et al., 2002
DS 107	Apatite	30°32'29"	116°17'01"	—	—	gneiss	—	—	58.90	1.90	Grimmer et al., 2002
DS 123	Apatite	30°24'32"	116°07'12"	—	—	gneiss	—	—	62.10	13.20	Grimmer et al., 2002
DS 270	Apatite	29°59'55"	114°50'15"	—	—	granite	—	—	88.70	3.10	Grimmer et al., 2002

DS 277	Apatite	29°30'49"	116°02'22"	—	—	granite	—	—	59.00	2.60	Grimmer et al., 2002
DS 287	Apatite	30°37'22"	116°51'43"	—	—	diorite	—	—	115.60	3.30	Grimmer et al., 2002
DS 516	Apatite	31°43'08"	116°30'35"	—	Lower Cretaceous	sandstone	—	—	105.40	6.10	Grimmer et al., 2002
DS 543	Apatite	31°27'44"	116°10'30"	—	—	quartzite	—	—	68.20	2.40	Grimmer et al., 2002
DS 545	Apatite	31°28'59"	116°12'10"	—	—	gneiss	—	—	60.60	4.70	Grimmer et al., 2002
DS 554a	Apatite	30°50'32"	116°17'36"	—	—	granite	—	—	60.80	5.20	Grimmer et al., 2002
DS 555	Apatite	30°48'59"	116°15'29"	—	—	granite	—	—	61.70	2.30	Grimmer et al., 2002
DS 559	Apatite	30°40'02"	116°29'02"	—	—	gneiss	—	—	49.50	3.20	Grimmer et al., 2002
DS 561	Apatite	30°27'48"	116°14'50"	—	—	gneiss	—	—	48.50	3.50	Grimmer et al., 2002
DS 565a	Apatite	30°27'14"	116°16'29"	—	—	gneiss	—	—	48.10	1.80	Grimmer et al., 2002
DS 567f	Apatite	30°33'14"	116°49'48"	—	—	granite	—	—	72.80	3.90	Grimmer et al., 2002
DS 580	Apatite	30°49'42"	117°38'42"	—	—	gneiss	—	—	91.50	6.20	Grimmer et al., 2002
J 32	Apatite	31°19'24"	117°11'54"	—	—	subvolcanic	—	—	117.80	3.70	Grimmer et al., 2002
J 46	Apatite	30°55'12"	117°54'30"	—	Middle Triassic	sandstone	—	—	103.00	3.60	Grimmer et al., 2002
J 57	Apatite	30°49'48"	117°16'24"	—	Lower-Middle Jurassic	sandstone	—	—	109.50	5.90	Grimmer et al., 2002
J 59	Apatite	30°49'36"	117°16'18"	—	Middle-Upper Jurassic	sandstone	—	—	96.40	4.20	Grimmer et al., 2002
J 66	Apatite	30°39'54"	116°59'36"	—	Middle Triassic	sandstone	—	—	158.20	14.30	Grimmer et al., 2002
J 78	Apatite	30°24'18"	116°36'30"	—	Middle Jurassic	sandstone	—	—	94.40	6.30	Grimmer et al., 2002
DS 91-28a	Apatite	30°45'30"	115°44'24"	—	—	gneiss	—	—	80.20	7.50	Grimmer et al., 2002
DS 91-34a	Apatite	30°48'24"	115°49'00"	—	—	gneiss	—	—	68.40	6.60	Grimmer et al., 2002
DS 91-64a	Apatite	30°45'42"	115°46'42"	—	—	gneiss	—	—	70.70	9.00	Grimmer et al., 2002
DS 98-17	Apatite	31°10'18"	116°32'18"	—	—	gneiss	—	—	59.00	1.30	Grimmer et al., 2002
DS 98-33	Apatite	30°47'52"	116°15'13"	—	—	gneiss	—	—	50.80	3.00	Grimmer et al., 2002
DS 98-35	Apatite	30°46'50"	116°13'41"	—	—	gneiss	—	—	46.20	2.80	Grimmer et al., 2002
Y 113	Apatite	30°27'01"	115°47'13"	—	—	gneiss	—	—	40.90	1.50	Grimmer et al., 2002
Y 118	Apatite	30°18'19"	115°09'24"	—	—	granite	—	—	67.60	2.70	Grimmer et al., 2002
Y 126	Apatite	30°18'14"	114°57'08"	—	Middle Jurassic	sandstone	—	—	66.30	1.80	Grimmer et al., 2002
Y 158	Apatite	30°14'34"	115°00'59"	—	Middle Triassic	sandstone	—	—	66.70	2.60	Grimmer et al., 2002
DS98-17	Apatite	31°10'18"	116°32'17"	—	—	eclogite	—	—	62.60	2.00	Wu et al., 2002
DS072	Apatite	31°07'48"	116°31'52"	—	—	diorite	—	—	69.60	3.40	Wu et al., 2002

DS095	Apatite	30°42'47"	116°23'26"	—	—	granite	—	—	66.90	2.90	Wu et al., 2002
DS103	Apatite	30°40'00"	116°27'00"	—	—	gneiss	—	—	64.70	3.00	Wu et al., 2002
DS107	Apatite	30°32'48"	116°17'01"	—	—	quartzite	—	—	61.30	2.40	Wu et al., 2002
DS076	Apatite	30°47'00"	116°05'00"	—	—	granite	—	—	77.30	3.10	Wu et al., 2002
DS98-35	Apatite	30°46'50"	116°13'41"	—	—	eclogite	—	—	59.40	3.40	Wu et al., 2002
DS98-33	Apatite	30°47'52"	116°15'13"	—	—	eclogite	—	—	59.80	3.50	Wu et al., 2002
DB9164	Apatite	30°45'42"	115°46'42"	—	—	gneiss	—	—	71.30	9.30	Wu et al., 2002
DB9134	Apatite	30°48'24"	115°49'00"	—	—	gneiss	—	—	68.10	6.90	Wu et al., 2002
DB9128	Apatite	30°45'30"	115°44'24"	—	—	gneiss	—	—	79.70	7.80	Wu et al., 2002
DS158	Apatite	30°45'25"	115°44'57"	—	—	gneiss	—	—	81.00	5.20	Wu et al., 2002
DS123-2	Apatite	30°24'53"	116°07'20"	—	—	granite-gneiss	—	—	105.60	9.80	Wu et al., 2002
DS025	Apatite	30°18'03"	115°56'09"	—	—	gneiss	—	—	79.70	9.70	Wu et al., 2002
DS98-08	Apatite	31°22'08"	116°22'10"	—	—	schist	—	—	67.70	4.90	Wu et al., 2002
CN026	Apatite	31°28'59"	116°12'01"	—	—	gneiss	—	—	60.60	4.70	Wu et al., 2002
CN025	Apatite	31°17'44"	116°10'30"	—	—	quartzite	—	—	68.20	2.40	Wu et al., 2002
CN043	Apatite	31°22'40"	116°11'50"	—	—	schist	—	—	90.20	6.50	Wu et al., 2002
CN058	Apatite	31°14'34"	116°20'39"	—	—	gabbro	—	—	86.50	3.00	Wu et al., 2002
DB2	Apatite	31°21'01"	114°18'41"	110	—	granite	13.40	0.30	74.90	2.80	Xu et al., 2004
DB5	Apatite	31°28'41"	114°44'32"	80	rchaeozoic-Lower Proterozoi	gneiss	11.50	0.50	77.70	3.20	Xu et al., 2004
DB6	Apatite	31°33'43"	114°49'20"	380	—	granite	13.70	0.40	68.80	3.60	Xu et al., 2004
DB7	Apatite	31°45'09"	115°13'42"	120	—	granite	10.50	0.96	61.60	2.80	Xu et al., 2004
DB8	Apatite	31°48'13"	115°14'40"	135	Middle Carboniferous	altered sandstone	12.90	0.40	83.30	4.90	Xu et al., 2004
DB12	Apatite	31°51'18"	115°46'19"	70	Middle Jurassic	sandstone	11.50	1.70	62.00	14.80	Xu et al., 2004
DB13	Apatite	31°48'42"	115°59'16"	50	Upper Cretaceous	andesite	8.60	1.40	64.20	4.60	Xu et al., 2004
DB14	Apatite	31°44'44"	115°55'16"	60	Palaeocene	sandstone	10.70	0.60	85.90	6.40	Xu et al., 2004
DB16	Apatite	31°35'33"	115°40'53"	150	—	diorite	14.00	0.20	92.70	6.00	Xu et al., 2004
DB17	Apatite	31°32'18"	115°39'46"	140	—	gneiss	14.00	0.50	77.80	11.40	Xu et al., 2004
DB18	Apatite	31°27'22"	115°41'13"	250	Upper Jurassic	triff	9.10	0.60	83.60	5.20	Xu et al., 2004
DB19	Apatite	31°26'12"	115°36'28"	270	rchaeozoic-Lower Proterozoi	leptite	12.70	0.40	96.40	3.80	Xu et al., 2004
DB20	Apatite	31°19'38"	115°30'39"	290	—	granite	13.50	0.20	58.30	4.80	Xu et al., 2004

DB24	Apatite	30°24'44"	115°30'47"	45	—	granite	13.10	0.40	51.20	5.70	Xu et al., 2004
DB25	Apatite	30°42'21"	115°57'15"	210	—	diorite	13.90	0.20	71.40	8.00	Xu et al., 2004
DB26	Apatite	30°49'38"	116°23'52"	340	—	diorite	13.90	0.20	71.30	6.10	Xu et al., 2004
DB27	Apatite	31°04'03"	116°29'08"	440	—	granite	12.60	0.45	56.60	6.20	Xu et al., 2004
DB31	Apatite	31°23'19"	116°28'11"	135	Lower Cretaceous	sandstone	13.60	0.30	90.40	2.90	Xu et al., 2004
DB34	Apatite	31°08'56"	116°01'56"	390	—	granite	—	—	41.90	13.40	Xu et al., 2004
DB40	Apatite	31°06'21"	115°46'15"	1729	Lower Cretaceous	granite	—	—	69.52	3.13	Zhou et al., 2003
DB41	Apatite	31°06'29"	115°46'44"	1450	Lower Cretaceous	granite	—	—	73.23	4.25	Zhou et al., 2003
DB42	Apatite	31°06'42"	115°47'14"	1070	Lower Cretaceous	granite	—	—	63.93	4.45	Zhou et al., 2003
DB43	Apatite	31°05'48"	115°47'19"	860	Lower Cretaceous	granite	—	—	49.54	4.88	Zhou et al., 2003
DB44	Apatite	31°05'09"	115°48'32"	620	Archaeozoic-Lower Proterozoi	migmatite	—	—	48.96	4.37	Zhou et al., 2003
DB45	Apatite	31°04'24"	115°49'24"	340	Archaeozoic-Lower Proterozoi	migmatite	—	—	43.57	4.22	Zhou et al., 2003
DH1	Apatite	43°12'29"	117°40'23"	1030	Jurassic-Lower Cretaceous	granite	12.40	1.50	51.00	3.00	Li XM et al., 2011
DH4	Apatite	43°36'33"	117°25'42"	1540	Upper Jurassic	moyite	—	—	51.00	3.00	Li XM et al., 2011
DH5	Apatite	43°57'15"	117°31'19"	1750	Jurassic-Lower Cretaceous	monzogranite	12.70	1.50	47.00	3.00	Li XM et al., 2011
DH6	Apatite	43°58'20"	117°33'12"	1728	Jurassic-Lower Cretaceous	granite	—	—	52.00	5.00	Li XM et al., 2011
DH8	Apatite	43°51'41"	117°55'58"	1090	Jurassic-Lower Cretaceous	granodiorite	—	—	50.00	6.00	Li XM et al., 2011
DH9	Apatite	43°46'50"	117°53'23"	1110	Jurassic-Lower Cretaceous	diorite	—	—	47.00	4.00	Li XM et al., 2011
DH10	Apatite	43°44'47"	117°55'07"	1035	Jurassic-Lower Cretaceous	granite porphyry	—	—	48.00	4.00	Li XM et al., 2011
DH11	Apatite	43°25'04"	118°05'59"	960	Jurassic-Lower Cretaceous	granite	13.00	1.40	49.00	3.00	Li XM et al., 2011
DH12	Apatite	43°49'22"	119°18'31"	659	Jurassic-Lower Cretaceous	granite	12.30	2.30	55.00	3.00	Li XM et al., 2011
DH13	Apatite	44°15'00"	119°09'26"	633	Jurassic-Lower Cretaceous	monzonite	—	—	56.00	5.00	Li XM et al., 2011
DH14	Apatite	44°28'33"	118°53'22"	1050	Upper Triassic-Upper Jurassic	diorite	—	—	66.00	4.00	Li XM et al., 2011
DH15	Apatite	44°12'57"	119°15'32"	619	Jurassic-Lower Cretaceous	granite	—	—	57.00	3.00	Li XM et al., 2011
DH17	Apatite	44°56'20"	120°24'31"	635	Jurassic-Lower Cretaceous	granodiorite	—	—	64.00	6.00	Li XM et al., 2011
DH19	Apatite	45°26'58"	120°28'41"	836	Upper Jurassic-Lower Cretaceous	riebeckite granite	—	—	49.00	4.00	Li XM et al., 2011
DH20	Apatite	45°58'09"	121°40'39"	448	Triassic-Jurassic	diorite	—	—	49.00	3.00	Li XM et al., 2011
DH22A	Apatite	45°27'47"	121°01'13"	478	Upper Permian	quartz diorite	12.70	1.80	61.00	3.00	Li XM et al., 2011
DH22B	Apatite	45°27'47"	121°01'13"	478	Upper Permian	granodiorite	12.70	1.50	63.00	5.00	Li XM et al., 2011
DH23	Apatite	41°50'35"	121°09'41"	268	Jurassic-Lower Cretaceous	diorite	—	—	57.00	5.00	Li XM et al., 2011

DH24	Apatite	46°13'33"	121°28'56"	389	Triassic	granite	—	—	51.00	5.00	Li XM et al., 2011
DH25	Apatite	46°18'12"	121°17'43"	435	Jurassic-Lower Cretaceous	granite	13.00	2.00	53.00	2.00	Li XM et al., 2011
DH26	Apatite	47°18'56"	119°45'47"	867	Upper Jurassic	granite	—	—	52.20	4.10	Li XM et al., 2011
DH28	Apatite	49°30'18"	117°37'53"	652	Jurassic-Lower Cretaceous	quartz monzonite	12.50	1.50	50.00	4.00	Li XM et al., 2011
DH30	Apatite	50°32'14"	119°43'16"	730	Upper Permian	monzogranite	13.30	2.20	63.00	3.00	Li XM et al., 2011
DH31	Apatite	50°46'16"	121°29'35"	723	Jurassic-Lower Cretaceous	granite porphyry	—	—	60.00	4.00	Li XM et al., 2011
DH33	Apatite	49°05'03"	121°01'33"	698	Triassic	monzogranite porphyry	12.70	2.00	60.00	3.00	Li XM et al., 2011
DH35	Apatite	48°00'11"	122°46'19"	354	Lower Cretaceous	hornblende monzogranite	—	—	67.00	4.00	Li XM et al., 2011
DH36	Apatite	47°33'24"	122°51'05"	353	Lower Cretaceous	alkaline granite	—	—	55.00	7.00	Li XM et al., 2011
DH37	Apatite	47°33'18"	122°35'49"	349	Triassic	porphyritic granite	12.70	1.60	58.00	4.00	Li XM et al., 2011
DH38	Apatite	47°45'09"	122°21'39"	414	Jurassic-Lower Cretaceous	granodiorite	12.30	1.90	49.00	3.00	Li XM et al., 2011
DH40	Apatite	50°25'19"	124°07'11"	403	Triassic	moyite	12.70	1.80	58.00	4.00	Li XM et al., 2011
DH41	Apatite	50°33'47"	125°41'26"	463	Lower Cretaceous	biotite monzogranite	—	—	57.00	4.00	Li XM et al., 2011
DH42	Apatite	50°35'25"	124°16'26"	459	Upper Jurassic-Upper Cretaceous	plagiogranite	12.80	2.00	59.00	3.00	Li XM et al., 2011
DH43	Apatite	50°42'36"	124°18'32"	426	Permian	monzogranite	13.10	1.90	59.00	4.00	Li XM et al., 2011
DH45	Apatite	52°19'42"	124°41'03"	378	Carboniferous	monzogranite	12.90	1.70	59.00	3.00	Li XM et al., 2011
DH46	Apatite	52°59'10"	122°28'43"	507	Triassic	diorite porphyry	13.30	2.10	64.00	3.00	Li XM et al., 2011
NML-1	Apatite	29°35'15"	94°56'34"	3393	Carboniferous	gneiss	14.00	0.50	1.58	0.28	Lei et al., 2008b
NML-3	Apatite	29°35'08"	94°57'34"	3660	Carboniferous	gneiss	14.40	0.30	0.97	0.11	Lei et al., 2008b
NML-4	Apatite	29°34'49"	94°57'25"	3740	Carboniferous	gneiss	14.00	0.70	1.27	0.26	Lei et al., 2008b
NML-5	Apatite	29°34'23"	94°57'32"	3958	Carboniferous	gneiss	15.00	0.20	0.64	0.09	Lei et al., 2008b
NML-6	Apatite	29°33'14"	94°58'08"	4373	Carboniferous	gneiss	15.20	0.40	1.10	0.24	Lei et al., 2008b
NML-7	Apatite	29°32'39"	94°58'23"	4295	Carboniferous	gneiss	14.30	0.30	1.22	0.26	Lei et al., 2008b
NML-8-1	Apatite	29°32'38"	94°57'37"	4291	Carboniferous	gneiss	15.20	0.20	0.65	0.08	Lei et al., 2008b
NML-8-5	Apatite	29°32'38"	94°59'37"	4291	Carboniferous	gneiss	14.50	0.40	0.67	0.10	Lei et al., 2008b
NML-9	Apatite	29°32'54"	94°57'25"	4295	Carboniferous	gneiss	14.10	0.30	0.74	0.13	Lei et al., 2008b
NML-11	Apatite	29°33'00"	94°56'47"	4537	Carboniferous	gneiss	15.00	0.70	1.17	0.20	Lei et al., 2008b
N-02	Apatite	29°33'20.8"	94°55'55"	3908	—	gneiss	14.62	0.34	1.37	—	Kang et al., 2016
N-03	Apatite	29°33'21.3"	94°55'55.7"	3990	—	gneiss	15.16	0.18	1.23	—	Kang et al., 2016
N-04	Apatite	29°33'24"	94°55'56"	4000	—	gneiss	15.01	0.18	1.19	—	Kang et al., 2016

N-05	Apatite	29°33'24.9"	94°55'57.4"	4048	—	gneiss	14.85	0.25	0.71	—	Kang et al., 2016
N-06	Apatite	29°33'19.9"	94°55'22.9"	3491	—	gneiss	14.93	0.19	2.07	—	Kang et al., 2016
N-07	Apatite	29°33'18.3"	94°54'40.7"	3201	—	gneiss	14.51	0.44	0.74	—	Kang et al., 2016
N-08	Apatite	29°33'20.8"	94°54'38.5"	3161	—	gneiss	15.14	0.25	2.05	—	Kang et al., 2016
N-10	Apatite	29°34'02"	94°54'26.8"	3022	—	gneiss	15.87	0.87	1.44	—	Kang et al., 2016
N1	Apatite	28°22'01.77'	86°00'58.64"	4353	Upper Proterozoic	gneiss	—	—	15.34	1.44	Zheng et al., 2014
N2	Apatite	28°20'07.86'	86°03'10.34"	4248	Upper Proterozoic	gneiss	—	—	10.30	0.93	Zheng et al., 2014
N3	Apatite	28°18'35.28'	86°02'07.41"	4152	Upper Proterozoic	gneiss	—	—	8.24	0.80	Zheng et al., 2014
N4	Apatite	28°13'02.62'	86°00'01.13"	3930	Upper Proterozoic	gneiss	—	—	9.49	0.98	Zheng et al., 2014
N5	Apatite	28°11'41.00'	85°59'25.06"	3845	Upper Proterozoic	gneiss	—	—	6.85	0.71	Zheng et al., 2014
N6	Apatite	28°08'30.46'	85°58'41.94"	3818	Upper Proterozoic	gneiss	—	—	2.88	0.25	Zheng et al., 2014
N7	Apatite	28°07'48.61'	85°58'41.64"	3714	Upper Proterozoic	gneiss	—	—	2.91	0.28	Zheng et al., 2014
N8	Apatite	28°04'54.22'	86°00'05.08"	3328	Upper Proterozoic	gneiss	—	—	2.89	0.35	Zheng et al., 2014
N9	Apatite	28°03'56.27'	86°00'08.79"	3223	Upper Proterozoic	gneiss	—	—	2.94	0.34	Zheng et al., 2014
N10	Apatite	28°02'15.26'	85°59'22.23"	3025	Upper Proterozoic	gneiss	—	—	2.63	0.34	Zheng et al., 2014
N11	Apatite	28°02'30.39'	85°59'18.28"	2919	Upper Proterozoic	gneiss	—	—	2.18	0.25	Zheng et al., 2014
N12	Apatite	28°01'19.34'	85°59'11.11"	2724	Upper Proterozoic	gneiss	—	—	1.78	0.22	Zheng et al., 2014
N13	Apatite	27°59'48.86'	85°58'50.92"	2518	Upper Proterozoic	gneiss	—	—	1.66	0.19	Zheng et al., 2014
N14	Apatite	27°58'58.63'	85°59'07.68"	2241	Upper Proterozoic	gneiss	—	—	1.52	0.24	Zheng et al., 2014
N15	Apatite	28°58'31.88'	85°59'34.14"	2136	Upper Proterozoic	gneiss	—	—	1.14	0.14	Zheng et al., 2014
ZAY06	Apatite	28°07'20"	86°36'08"	5793	—	granite	—	—	13.50	2.50	Gao et al., 2014
ZAY07	Apatite	28°06'07"	86°37'16"	6022	—	monzonite	—	—	13.40	2.00	Gao et al., 2014
ZAY10	Apatite	28°06'19"	86°37'31"	6328	—	granite	—	—	12.40	1.70	Gao et al., 2014
ZAY11	Apatite	28°06'24"	86°37'31"	6435	—	pegmatite	—	—	14.30	1.60	Gao et al., 2014
T4	Apatite	28°17'22"	86°01'27"	4122	Middle-Upper Proterozoic	granite	—	—	8.20	0.70	Wang et al., 2010a
T6	Apatite	28°11'31"	85°59'06"	3848	Middle-Upper Proterozoic	graniteic gneiss	—	—	3.80	0.70	Wang et al., 2010a
T8	Apatite	28°08'15"	85°58'19"	3770	Middle-Upper Proterozoic	biotitic plagiogneiss	—	—	2.50	0.30	Wang et al., 2010a
T9	Apatite	28°06'02"	85°59'40"	3447	Middle-Upper Proterozoic	granitic mylonite	—	—	1.60	0.40	Wang et al., 2010a
T12	Apatite	28°01'28"	85°59'05"	2660	Middle-Upper Proterozoic	biotitic plagiogneiss	—	—	1.50	0.30	Wang et al., 2010a
T13	Apatite	27°59'04"	85°58'55"	2450	Middle-Upper Proterozoic	biotitic plagiogneiss	—	—	0.90	0.40	Wang et al., 2010a

F54	Apatite	38°05'31"	111°29'20"	1584	Archaeozoic	granitegneiss	11.30	0.20	108.90	4.70	Ren et al., 2015
F57	Apatite	37°59'56"	111°15'47"	1487	Proterozoic	quartzite	11.52	0.16	120.60	7.40	Ren et al., 2015
F61	Apatite	38°09'00"	111°18'29"	1446	Archaeozoic	gneiss	11.77	0.15	94.90	3.50	Ren et al., 2015
F62	Apatite	37°45'50"	111°30'11"	1542	Archaeozoic	granite	—	—	102.70	5.10	Ren et al., 2015
F63	Apatite	37°45'54"	111°30'11"	1545	Archaeozoic	granite	—	—	113.90	6.70	Ren et al., 2015
F64	Apatite	37°41'31"	111°45'22"	1198	Archaeozoic	granite	11.26	1.53	48.90	2.10	Ren et al., 2015
F65	Apatite	37°39'11"	111°44'10"	1153	Proterozoic	dolerite	11.42	1.60	70.50	3.00	Ren et al., 2015
F67	Apatite	37°48'00"	111°29'17"	1660	Archaeozoic	granodiorite	—	—	82.10	5.30	Ren et al., 2015
F68	Apatite	37°47'13"	111°25'19"	1965	Archaeozoic	granite	10.62	1.60	138.70	6.60	Ren et al., 2015
F69	Apatite	37°43'19"	111°20'49"	1259	Archaeozoic	granite	11.75	1.91	74.60	3.80	Ren et al., 2015
F70	Apatite	37°25'34"	111°52'52"	881	Permian	sandstone	11.72	1.84	50.50	3.10	Ren et al., 2015
F71	Apatite	37°42'40"	111°54'25"	1339	Permian	sandstone	10.66	1.61	104.60	6.60	Ren et al., 2015
F73	Apatite	37°24'29"	110°47'35"	749	Permian	sandstone	11.97	1.74	39.90	1.90	Ren et al., 2015
F74	Apatite	37°33'00"	111°58'34"	790	Permian	sandstone	13.08	1.48	42.30	1.90	Ren et al., 2015
F80	Apatite	37°53'53"	111°13'59"	1250	Archaeozoic	granodiorite	11.84	1.71	61.50	2.40	Ren et al., 2015
X8	Apatite	43°42'58"	87°48'07"	1343	Middle Carboniferous	tuffaceous sandstone	13.00	1.30	119.90	26.20	Tang et al., 2015
X9	Apatite	43°42'29"	87°47'34"	1326	Carboniferous	diorite porphyry	11.50	1.30	131.90	21.90	Tang et al., 2015
X10	Apatite	43°42'29"	87°47'34"	1326	Upper Carboniferous	sandstone	11.00	2.80	136.20	28.60	Tang et al., 2015
X11	Apatite	43°41'38"	87°47'16"	1269	Middle Carboniferous	sandstone	11.10	3.10	119.90	43.10	Tang et al., 2015
T27	Apatite	43°41'37"	87°57'15"	1277	Middle Carboniferous	sandstone	11.20	2.30	121.30	25.90	Tang et al., 2015
T28	Apatite	43°41'23"	87°46'24"	1283	Upper Carboniferous	sandstone	11.10	2.40	125.40	14.80	Tang et al., 2015
X14	Apatite	43°39'17"	87°41'26"	1124	Upper Carboniferous	sandstone	12.30	1.90	103.40	10.20	Tang et al., 2015
X15	Apatite	43°39'06"	87°39'06"	1096	Upper Carboniferous	sandstone	13.20	1.50	109.20	11.30	Tang et al., 2015
X16	Apatite	43°39'18"	87°38'11"	1076	Lower Permian	sandstone	11.90	2.30	85.60	10.10	Tang et al., 2015
X17	Apatite	43°41'12"	87°35'28"	1017	Lower Permian	sandstone	12.40	1.60	102.20	11.90	Tang et al., 2015
BG1	Apatite	43°46'6.3"	87°49'51.3"	1223	Middle Carboniferous	tuff	11.90	2.40	31.40	5.70	Shen et al., 2006
BG2	Apatite	43°48'4.8"	87°43'11.3"	938	Upper Triassic	sandstone	11.70	1.90	24.20	4.40	Shen et al., 2006
BG3	Apatite	43°41'38.6"	87°55'7.9"	1666	Middle Carboniferous	tuff	12.90	2.10	43.90	8.20	Shen et al., 2006
BG4	Apatite	44°03'17.5"	88°19'49.0"	1087	Upper Permian	tuff-sandstone	11.20	1.90	42.60	5.60	Shen et al., 2006
BG5	Apatite	44°04'11.7"	88°20'58.7"	998	Upper Permian	tuff-sandstone	11.50	1.90	28.80	2.80	Shen et al., 2006

Sx24	Apatite	27°18'35"	112°35'55"	114	Cretaceous	mylonitic albitite	12.20	0.50	79.00	4.00	Li JH et al., 2016
Sx25-1	Apatite	27°16'44"	112°35'58"	98	Proterozoic	biotite gneiss	12.90	0.20	81.00	5.00	Li JH et al., 2016
Sx25-2	Apatite	27°16'45"	112°35'57"	103	Proterozoic	biotite schist	—	—	81.00	5.00	Li JH et al., 2016
Sx25-3	Apatite	27°16'47"	112°35'56"	98	Proterozoic	biotite schist	—	—	84.00	5.00	Li JH et al., 2016
Sx26	Apatite	27°16'28"	112°36'50"	218	Jurassic	monzogranite	13.10	0.10	83.00	5.00	Li JH et al., 2016
Sx28	Apatite	27°15'44"	112°39'26"	485	Jurassic	monzogranite	13.00	0.20	83.00	5.00	Li JH et al., 2016
Sx29	Apatite	27°15'38"	112°40'16"	622	Jurassic	biotite monzogranite	13.60	0.10	84.00	4.00	Li JH et al., 2016
Sx30	Apatite	27°15'18"	112°41'50"	605	Triassic	granodiorite	136.00	0.10	79.00	5.00	Li JH et al., 2016
Sx31	Apatite	27°13'58"	112°43'17"	608	Triassic	biotite monzogranite	—	—	83.00	5.00	Li JH et al., 2016
HS08	Apatite	30°11'27.5"	118°48'52.8"	1196	Jurassic-Lower Cretaceous	granite	11.90	2.10	55.00	5.00	Zheng et al., 2009
HS09	Apatite	30°11'30.2"	118°48'59.8"	1146	Jurassic-Lower Cretaceous	granite	12.10	1.80	68.00	7.00	Zheng et al., 2009
HS10	Apatite	30°11'57.9"	118°49'18.6"	936	Jurassic-Lower Cretaceous	granite	12.10	2.10	60.00	5.00	Zheng et al., 2009
HS11	Apatite	30°12'14.6"	118°48'46.8"	733	Jurassic-Lower Cretaceous	granite	10.90	2.30	51.00	5.00	Zheng et al., 2009
HS12	Apatite	30°13'12.6"	118°48'11.9"	415	Jurassic-Lower Cretaceous	granite	12.00	2.00	54.00	4.00	Zheng et al., 2009
XS-5	Apatite	40°5'6.2"	119°26'44.9"	807	Lower Cretaceous	granite	—	—	76.80	14.20	Li et al., 2009
XS-6	Apatite	40°4'55.6"	119°26'45.8"	650	Lower Cretaceous	granite	—	—	65.30	10.30	Li et al., 2009
XS-8	Apatite	40°4'23.6"	119°27'56.3"	406	Lower Cretaceous	granite	—	—	58.80	8.40	Li et al., 2009
Hb05	Apatite	29°11'38"	113°56'56"	159	Jurassic-Cretaceous	granite	12.10	0.30	40.80	3.20	Shi HC et al., 2013
Hb08	Apatite	29°19'48"	113°38'24"	322	Jurassic-Cretaceous	granite	—	—	65.40	5.50	Shi HC et al., 2013
Sy03	Apatite	29°06'07"	114°03'03"	339	Jurassic-Cretaceous	granite	12.00	0.20	43.00	3.20	Shi HC et al., 2013
Sy06	Apatite	29°03'50"	113°45'36"	189	Jurassic-Cretaceous	granite	11.20	0.20	30.70	2.20	Shi HC et al., 2013
Sy07	Apatite	28°59'42"	113°44'41"	166	Jurassic-Cretaceous	granite	—	—	48.10	4.00	Shi HC et al., 2013
Sy12	Apatite	29°13'25"	113°31'40"	298	Jurassic-Cretaceous	granite	11.30	0.30	27.40	1.80	Shi HC et al., 2013
Sy13	Apatite	29°14'22"	113°31'09"	442	Jurassic-Cretaceous	granite	11.30	0.20	60.10	3.60	Shi HC et al., 2013
Sy14	Apatite	29°18'45"	113°40'09"	400	Jurassic-Cretaceous	granite	—	—	71.20	7.10	Shi HC et al., 2013
NC-03	Apatite	41°40'32"	110°27'09"	1382	Upper Palaeozoic	granodiorite	12.00	0.10	182.20	1.90	Li K et al., 2016
NC-04	Apatite	41°40'32"	110°45'25"	1382	Upper Palaeozoic	granodiorite	1.2.2	0.10	218.40	13.90	Li K et al., 2016
NC-05	Apatite	41°41'58"	110°25'10"	1375	Upper Palaeozoic	granite	11.90	0.10	248.70	21.90	Li K et al., 2016
NC-06	Apatite	41°42'43"	110°26'58"	1371	Upper Palaeozoic	granodiorite	12.40	0.10	171.50	10.30	Li K et al., 2016
NC-07	Apatite	41°44'26"	110°25'20"	1339	Upper Palaeozoic	granite	11.30	0.20	142.80	10.30	Li K et al., 2016

NC-08	Apatite	41°45'49"	110°32'27"	1419	Upper Palaeozoic	granodiorite	—	—	146.70	25.00	Li K et al., 2016
BT-64	Apatite	41°54'54"	110°07'20"	1426	Upper Palaeozoic	granodiorite	12.50	0.10	151.50	13.10	Li K et al., 2016
BT-65	Apatite	41°59'19"	110°06'57"	1361	Lower Palaeozoic	granodiorite	13.10	1.00	134.80	20.60	Li K et al., 2016
BT-66	Apatite	41°59'52"	110°06'45"	1351	Middle Carboniferous	sandstone	12.70	0.10	126.50	9.50	Li K et al., 2016
BT-67	Apatite	42°07'03"	110°25'58"	1242	Lower Palaeozoic	granodiorite	12.10	0.20	125.60	9.00	Li K et al., 2016
MD-80	Apatite	42°32'50"	110°05'47"	1260	Lower Permian	sandstone	12.00	0.50	155.70	15.10	Li K et al., 2016
MD-81	Apatite	42°31'19"	110°06'55"	1218	Lower Permian	sandstone	12.60	0.20	185.50	14.50	Li K et al., 2016
MD-85	Apatite	42°39'25"	110°21'43"	1007	Lower Permian	sandstone	11.20	0.30	170.10	11.80	Li K et al., 2016
08LHS-02	Apatite	41°50'33.2"	103°10'43.4"	—	Upper Permian	clasolite	12.80	1.70	90.00	6.00	Han et al., 2014
08LHS-09	Apatite	41°50'33.2"	103°10'43.4"	—	Upper Permian	clasolite	13.20	1.70	106.00	7.00	Han et al., 2014
08LHS-03	Apatite	41°28'48.2"	103°45'51.8"	—	Permian	clasolite	12.90	1.80	110.00	7.00	Han et al., 2014
08LHS-05	Apatite	41°28'48.2"	103°45'51.8"	—	Permian	clasolite	12.90	1.70	96.00	6.00	Han et al., 2014
08LHS-06	Apatite	41°28'48.2"	103°45'51.8"	—	Permian	clasolite	12.50	1.70	96.00	6.00	Han et al., 2014
08LHS-07	Apatite	41°28'48.2"	103°45'51.8"	—	Permian	clasolite	12.60	1.90	114.00	8.00	Han et al., 2014
ZX1	Apatite	45°31'17.2"	83°50'56.0"	1510	Carboniferous	granite	12.10	2.20	67.00	6.00	Li W et al., 2010
D480/1	Apatite	45°43'55.5"	83°32'37.8"	1496	Carboniferous	granite	11.70	2.20	78.00	7.00	Li W et al., 2010
D480/2	Apatite	45°42'54.4"	83°33'33.3"	1563	Carboniferous	granite	11.50	1.30	79.00	7.00	Li W et al., 2010
D480/3	Apatite	45°35'14.7"	83°39'03.6"	1805	Carboniferous	granite	12.50	2.50	91.00	6.00	Li W et al., 2010
D480/4	Apatite	45°30'46.4"	83°56'23.1"	1398	Carboniferous	granite	13.60	1.90	121.00	7.00	Li W et al., 2010
DZX2	Apatite	45°41'53.9"	84°55'29.6"	617	Carboniferous	granite	13.60	2.10	123.00	9.00	Li W et al., 2010
D468/1	Apatite	45°42'40.0"	84°50'42.0"	732	Carboniferous	granite	12.40	1.60	121.00	6.00	Li W et al., 2010
D310/1	Apatite	46°34'46.6"	86°10'42.3"	1272	Upper Palaeozoic	granite	11.00	2.80	135.00	9.00	Li W et al., 2010
D311/1	Apatite	46°34'45.1"	86°10'43.4"	1250	Upper Palaeozoic	granite	11.70	2.50	108.00	7.00	Li W et al., 2010
D312/1	Apatite	46°34'44.4"	86°10'46.5"	1233	Upper Palaeozoic	granite	13.00	2.40	107.00	8.00	Li W et al., 2010
D313/1	Apatite	46°34'42.7"	86°10'48.5"	1200	Upper Palaeozoic	granite	12.50	2.50	102.00	7.00	Li W et al., 2010
D314/1	Apatite	46°34'31.0"	86°10'50.2"	1165	Upper Palaeozoic	granite	12.30	2.10	118.00	9.00	Li W et al., 2010
D407/1	Apatite	45°38'06.9"	90°22'07.4"	1580	Permian	granite	12.60	2.20	66.00	5.00	Li W et al., 2010
D407/2	Apatite	45°38'09.1"	90°22'07.3"	1538	Permian	granite	12.80	2.70	52.00	4.00	Li W et al., 2010
D407/3	Apatite	45°38'15.1"	90°22'05.9"	1470	Permian	granite	11.70	2.40	61.00	5.00	Li W et al., 2010
D407/4	Apatite	45°38'21.3"	90°22'05.4"	1408	Permian	granite	11.90	2.60	60.00	4.00	Li W et al., 2010

D407/5	Apatite	45°38'23.4"	90°22'02.5"	1328	Permian	granite	12.20	2.60	62.00	4.00	Li W et al., 2010
SH002	Apatite	30°10'24"	106°41'15"	260	Upper Triassic	sandstone	12.10	2.00	38.00	4.00	Deng et al., 2013a
SH003	Apatite	30°10'24"	106°41'15"	295	Upper Triassic	sandstone	12.30	2.10	42.00	3.00	Deng et al., 2013a
SH048	Apatite	30°46'02"	107°08'32"	463	Lower Jurassic	sandstone	11.10	2.00	24.00	2.00	Deng et al., 2013a
SH060	Apatite	30°46'26"	107°02'51"	579	Upper Triassic	sandstone	11.80	1.90	48.00	3.00	Deng et al., 2013a
SH098	Apatite	30°12'14"	106°51'09"	298	Middle Jurassic	sandstone	12.50	2.10	66.00	5.00	Deng et al., 2013a
SH100	Apatite	29°47'51"	106°28'33"	272	Upper Triassic	sandstone	12.90	2.20	50.00	3.00	Deng et al., 2013a
SH120	Apatite	29°56'43"	106°19'50"	209	Middle Jurassic	sandstone	12.40	2.30	51.00	5.00	Deng et al., 2013a
SN-01	Apatite	30°47'42"	106°20'59"	332	Middle Jurassic	sandstone	12.10	2.10	98.00	8.00	Deng et al., 2013a
SLG-01	Apatite	31°23'58"	106°58'04"	581	Upper Jurassic	sandstone	11.90	1.90	109.00	9.00	Deng et al., 2013a
SG-097	Apatite	31°12'31"	107°20'41"	553	Lower Jurassic	sandstone	12.00	1.90	82.00	7.00	Deng et al., 2013a
SG-098	Apatite	31°11'35"	107°23'05"	592	Upper Triassic	sandstone	11.50	1.80	80.00	6.00	Deng et al., 2013a
GN-09	Apatite	30°59'49"	105°52'31"	349	Upper Jurassic	sandstone	12.30	1.90	92.00	8.00	Deng et al., 2013a
GN-7	Apatite	31°25'52"	106°01'14"	421	Upper Jurassic	sandstone	12.10	1.60	109.00	10.00	Deng et al., 2013a
GN-06	Apatite	32°27'49"	105°50'32"	491	Middle Jurassic	sandstone	12.30	1.90	85.00	7.00	Deng et al., 2013a
GN-02	Apatite	32°29'57"	105°51'17"	517	Upper Triassic	sandstone	12.50	1.60	80.00	6.00	Deng et al., 2013a
WS02	Apatite	29°30'11"	104°11'12"	477	Middle Jurassic	sandstone	11.90	2.50	38.00	3.00	Liu et al., 2008
WS10	Apatite	29°24'31"	104°19'42"	342	Middle Jurassic	sandstone	11.50	2.60	26.00	3.00	Liu et al., 2008
WS13	Apatite	29°29'32"	104°26'48"	472	Lower Jurassic	sandstone	12.00	2.50	29.00	3.00	Liu et al., 2008
WS15	Apatite	29°32'37"	104°26'49"	595	Upper Triassic	sandstone	13.10	1.90	28.00	2.00	Liu et al., 2008
WS25	Apatite	29°35'02"	104°22'56"	642	Upper Triassic	sandstone	12.60	2.40	27.00	2.00	Liu et al., 2008
WS27	Apatite	29°33'43"	104°19'05"	491	Upper Triassic	sandstone	12.70	1.90	25.00	2.00	Liu et al., 2008
WS30	Apatite	29°36'13"	104°11'50"	437	Middle Jurassic	sandstone	12.90	1.80	37.00	3.00	Liu et al., 2008
WS33	Apatite	29°38'34"	104°04'44"	383	Middle Jurassic	sandstone	12.10	2.10	38.00	4.00	Liu et al., 2008
WS35	Apatite	29°40'26"	104°10'19"	321	Middle Jurassic	sandstone	11.90	2.40	22.00	2.00	Liu et al., 2008
WS36	Apatite	29°39'55"	104°14'47"	452	Lower Jurassic	sandstone	11.70	2.40	51.00	5.00	Liu et al., 2008
WS37	Apatite	29°41'58"	104°18'27"	495	Lower Jurassic	sandstone	11.80	2.10	33.00	4.00	Liu et al., 2008
WS40	Apatite	29°40'14"	104°21'14"	471	Upper Triassic	sandstone	12.40	2.10	47.00	4.00	Liu et al., 2008
WS41	Apatite	29°38'45"	104°24'47"	549	Upper Triassic	sandstone	12.80	1.90	23.00	2.00	Liu et al., 2008
WS43	Apatite	29°39'54"	104°29'26"	592	Upper Triassic	sandstone	13.40	1.50	28.00	2.00	Liu et al., 2008

WS46	Apatite	29°29'35"	104°42'22"	360	Middle Jurassic	sandstone	12.30	2.10	43.00	3.00	Liu et al., 2008
WS47	Apatite	29°26'45"	104°44'24"	369	Middle Jurassic	sandstone	12.20	2.30	35.00	3.00	Liu et al., 2008
WS49	Apatite	29°32'40"	104°36'25"	349	Middle Jurassic	sandstone	12.40	2.40	23.00	2.00	Liu et al., 2008
WS55	Apatite	29°43'49"	104°28'35"	634	Upper Triassic	sandstone	12.40	2.30	27.00	3.00	Liu et al., 2008
WS60	Apatite	29°48'37"	104°42'29"	378	Middle Jurassic	sandstone	12.60	2.50	31.00	3.00	Liu et al., 2008
WS65	Apatite	29°36'59"	104°44'47"	439	Middle Jurassic	sandstone	13.10	2.30	28.00	2.00	Liu et al., 2008
WS67	Apatite	29°35'02"	104°39'21"	397	Lower Jurassic	sandstone	12.20	2.60	26.00	2.00	Liu et al., 2008
WS70	Apatite	29°40'53"	104°37'15"	436	Upper Triassic	sandstone	13.20	1.70	41.00	5.00	Liu et al., 2008
ACQ1	Apatite	28°28'39"	106°10'32"	—	Cretaceous	sandstone	11.90	2.80	38.00	3.00	Deng et al., 2009
ACQ4	Apatite	28°22'04"	106°11'31"	—	Jurassic	sandstone	12.20	2.60	35.00	2.00	Deng et al., 2009
DT8	Apatite	28°36'01"	106°33'08"	—	Upper Jurassic	sandstone	12.00	2.70	52.00	4.00	Deng et al., 2009
DT2	Apatite	28°27'49"	106°32'27"	—	Upper Triassic	sandstone	12.00	2.40	52.00	5.00	Deng et al., 2009
DT4	Apatite	28°28'20"	106°32.258	—	Middle Jurassic	sandstone	11.80	2.50	37.00	2.00	Deng et al., 2009
DT6	Apatite	28°29'45"	106°31'04"	—	Upper Jurassic	sandstone	11.70	2.80	55.00	5.00	Deng et al., 2009
ADX4	Apatite	28°27'24"	106°26'45"	—	Middle Jurassic	sandstone	11.80	2.90	48.00	3.00	Deng et al., 2009
ACL14	Apatite	28°22'48"	106°24'00"	—	Permian	sandstone	11.90	2.80	38.00	3.00	Deng et al., 2009
B6-067	Apatite	30°01'17"	102°54'04"	620	Upper Cretaceous	sandstone	11.40	2.10	9.10	0.80	Li ZW et al., 2010
B5-066	Apatite	30°13'26"	102°50'31"	796	Upper Triassic	sandstone	11.70	2.00	43.00	3.00	Li ZW et al., 2010
A3-061	Apatite	31°38'14"	104°25'16"	597	Upper Jurassic	sandstone	11.80	1.90	108.00	10.00	Li ZW et al., 2010
QJ-56-1	Apatite	32°09'57"	105°20'32"	769	Upper Jurassic	sandstone	12.20	1.60	75.00	6.00	Li ZW et al., 2010
QJ-44-1	Apatite	32°15'30"	105°18'41"	586	Upper Triassic	sandstone	11.90	2.00	43.00	5.00	Li ZW et al., 2010
NJ14T	Apatite	32°27'19"	106°53'22"	575	Silurian	sandstone	—	—	60.80	5.70	Tian et al., 2010
NJ15T	Apatite	32°25'30"	106°51'51"	506	Upper Triassic	sandstone	—	—	73.80	3.70	Tian et al., 2010
NJ17T	Apatite	32°22'19"	106°51'10"	557	Jurassic	sandstone	—	—	68.40	3.40	Tian et al., 2010
FT-01	Apatite	28°56'15"	103°41'45"	610	Triassic-Jurassic	siltstone	11.54	0.22	20.20	3.70	An et al., 2008
FT-02	Apatite	28°56'18"	103°41'57"	498	Triassic-Jurassic	sandstone	12.19	0.19	8.50	1.50	An et al., 2008
FT-05	Apatite	28°56'43"	103°42'34"	437	Triassic-Jurassic	siltstone	12.26	0.20	23.50	2.90	An et al., 2008
FT-07	Apatite	28°14'05"	103°25'29"	914	Carboniferous	sandstone	12.35	0.20	20.70	3.30	An et al., 2008
FT-09	Apatite	28°12'28"	103°26'45"	653	Carboniferous	siltstone	11.52	0.20	11.00	3.20	An et al., 2008
FT-10	Apatite	28°29'30"	103°48'41"	379	Carboniferous	sandstone	11.80	0.23	10.40	4.20	An et al., 2008

FT-11	Apatite	28°29'46"	103°48'27"	455	Carboniferous	sandstone	11.20	0.16	15.80	2.60	An et al., 2008
FT-12	Apatite	28°29'42"	103°48'26"	510	Carboniferous	siltstone	12.13	0.24	30.70	3.50	An et al., 2008
FT-13	Apatite	28°14'22"	103°50'50"	517	Carboniferous	siltstone	12.13	0.24	22.30	3.30	An et al., 2008
FT-15	Apatite	28°14'52"	103°50'56"	504	Carboniferous	siltstone	12.52	0.18	27.20	6.70	An et al., 2008
2495-1	Apatite	30°55'05"	108°03'24"	440	Upper Triassic	sandstone	13.47	1.60	54.10	8.80	Richardson et al., 2008
2495-2	Apatite	30°54'27"	108°03'55"	275	Lower Jurassic	sandstone	—	—	66.50	8.20	Richardson et al., 2008
2495-3	Apatite	30°53'56"	108°04'17"	240	Middle Jurassic	sandstone	13.53	1.51	72.10	13.20	Richardson et al., 2008
2591-1	Apatite	30°14'56"	106°50'28"	290	Lower Jurassic	sandstone	—	—	80.90	14.60	Richardson et al., 2008
2592-2	Apatite	30°15'17"	106°49'50"	360	Upper Triassic	sandstone	—	—	84.90	12.00	Richardson et al., 2008
1835-3	Apatite	29°55'01"	106°02'06"	329	Jurassic	sandstone	—	—	62.10	11.60	Richardson et al., 2008
1835-4	Apatite	30°05'12"	105°29'32"	329	Jurassic	sandstone	—	—	119.90	17.70	Richardson et al., 2008
1835-5	Apatite	30°14'26"	105°02'30"	467	Jurassic	sandstone	—	—	98.20	20.00	Richardson et al., 2008
0735-2	Apatite	30°47'50"	104°28'17"	431	Jurassic	sandstone	—	—	40.30	6.00	Richardson et al., 2008
0835-1	Apatite	30°11'19"	104°05'53"	569	Jurassic	sandstone	—	—	32.30	6.00	Richardson et al., 2008
X-6	Apatite	30°11'30"	103°33'26"	809	Lower Jurassic	sandstone	14.09	1.83	32.90	6.00	Richardson et al., 2008
X-7	Apatite	30°11'40"	103°32'57"	622	Upper Triassic	sandstone	14.25	1.49	27.50	3.80	Richardson et al., 2008
1024-4	Apatite	30°47'51"	104°27'37"	461	Jurassic	sandstone	—	—	78.70	16.60	Richardson et al., 2008
1324-9	Apatite	30°13'55"	103°37'39"	731	Jurassic	sandstone	13.20	1.79	37.20	4.80	Richardson et al., 2008
1424-1	Apatite	30°00'22"	102°51'35"	618	Cretaceous-Paleogene	sandstone	—	—	72.60	12.20	Richardson et al., 2008
1424-2	Apatite	30°01'32"	102°53'44"	612	Cretaceous	sandstone	—	—	17.90	5.80	Richardson et al., 2008
1735-3	Apatite	29°34'58"	104°34'17"	377	Lower Jurassic	sandstone	—	—	30.80	4.40	Richardson et al., 2008
1435-1	Apatite	29°37'24"	103°24'38"	687	Triassic	sandstone	—	—	16.90	3.00	Richardson et al., 2008
0935-5	Apatite	31°04'30"	103°47'59"	670	Jurassic	sandstone	—	—	145.50	19.40	Richardson et al., 2008
1624-8	Apatite	30°04'14"	102°44'45"	761	Upper Cretaceous	sandstone	—	—	55.90	12.20	Richardson et al., 2008
FT-19	Apatite	30°37'52"	107°54'04"	410	Upper Triassic	sandstone	12.10	1.88	66.40	4.60	Wang et al., 2012
FT-28	Apatite	30°15'47"	108°15'14"	1120	Middle Jurassic	sandstone	12.47	1.83	97.40	6.20	Wang et al., 2012
FT-09	Apatite	30°43'23"	108°36'43"	370	Middle Jurassic	sandstone	11.91	1.96	84.40	5.60	Wang et al., 2012
FT-33	Apatite	29°44'46"	107°24'43"	230	Middle Jurassic	sandstone	12.38	1.99	49.10	3.40	Wang et al., 2012
FT-41	Apatite	29°43'52"	106°41'56"	220	Middle Jurassic	sandstone	12.78	1.95	52.40	3.40	Wang et al., 2012
FT-44	Apatite	29°51'11"	106°24'50"	260	Upper Triassic	sandstone	12.76	2.09	58.00	4.80	Wang et al., 2012

FT-46	Apatite	29°55'41"	106°22'37"	230	Upper Triassic	sandstone	12.09	2.22	67.10	4.50	Wang et al., 2012
F6	Apatite	40°46'03"	88°34'20"	1002	Ordovician	sandstone	—	—	130.00	16.00	Zhang et al., 2016
F12	Apatite	41°13'24"	88°28'24"	1360	Proterozoic	granite	11.02	0.20	184.00	6.00	Zhang et al., 2016
F13	Apatite	40°48'01"	88°31'06"	970	Ordovician	sandstone	11.89	0.24	214.00	9.00	Zhang et al., 2016
F14	Apatite	40°48'00"	88°31'08"	968	Ordovician	sandstone	11.82	0.57	95.00	12.00	Zhang et al., 2016
F15	Apatite	40°46'16"	88°33'05"	1065	Ordovician	sandstone	11.90	0.20	138.00	9.00	Zhang et al., 2016
F16	Apatite	40°46'09"	88°33'16"	1041	Ordovician	sandstone	11.38	0.27	129.00	7.00	Zhang et al., 2016
F17	Apatite	40°46'35"	88°34'20"	1024	Ordovician	sandstone	12.22	0.20	137.00	5.00	Zhang et al., 2016
F18	Apatite	40°46'15"	88°24'56"	853	Ordovician	sandstone	12.45	0.32	131.00	7.00	Zhang et al., 2016
F19	Apatite	40°46'23"	88°22'23"	833	Ordovician	sandstone	11.68	0.25	185.00	11.00	Zhang et al., 2016
K3	Apatite	41°47'12"	86°13'27"	1131	Proterozoic	granitic gneiss	12.18	0.17	85.00	5.00	Zhang et al., 2016
K9	Apatite	41°48'27"	86°14'31"	1229	Proterozoic	granitic gneiss	13.02	0.20	72.00	4.00	Zhang et al., 2016
K10	Apatite	41°48'43"	86°14'48"	1264	Proterozoic	hornblende schist	—	—	108.00	13.00	Zhang et al., 2016
K11	Apatite	41°48'56"	86°15'101	1209	Proterozoic	granitic gneiss	12.32	0.16	72.00	3.00	Zhang et al., 2016
T1	Apatite	41°47'19"	86°10'33"	1005	Archaeozoic	quartz schist	—	—	84.00	11.00	Zhang et al., 2016
T3	Apatite	41°48'33"	86°11'32"	992	Proterozoic	hornblende schist	—	—	100.00	16.00	Zhang et al., 2016
T6	Apatite	41°49'20"	86°11'48"	1017	Proterozoic	mica schist	13.53	0.23	96.00	5.00	Zhang et al., 2016
T7	Apatite	41°49'16"	86°11'58"	1028	Proterozoic	hornblende gneiss	11.65	0.17	77.00	3.00	Zhang et al., 2016
T8	Apatite	41°49'14"	86°12'11"	1039	Proterozoic	amphibolite	13.01	0.14	85.00	6.00	Zhang et al., 2016
T9	Apatite	41°49'14"	86°12'11"	1039	Proterozoic	amphibolite	12.96	0.15	75.00	4.00	Zhang et al., 2016
A29	Apatite	40°59'34"	79°59'18"	1392	Proterozoic	psammitic schist	12.31	0.15	126.00	7.00	Zhang et al., 2016
A33	Apatite	40°59'22"	79°59'31"	1294	Sinian	sandstone	12.13	0.22	53.00	6.00	Zhang et al., 2016
A34	Apatite	40°59'14"	79°59'30"	1272	Sinian	sandstone	13.29	0.23	42.00	5.00	Zhang et al., 2016
A35	Apatite	41°01'33"	80°02'49"	1219	Proterozoic	psammitic schist	12.69	0.15	121.00	5.00	Zhang et al., 2016
A37	Apatite	41°01'38"	80°02'31"	1250	Proterozoic	pelitic schist	12.53	0.17	119.00	5.00	Zhang et al., 2016
A1	Apatite	41°07'57.2"	79°59'21.5"	1147	Proterozoic	mica schist	12.12	0.62	62.50	5.60	Zhang et al., 2009
A2	Apatite	41°08'27.8"	80°00'13.7"	1148	Proterozoic	mica quartz schist	10.46	0.98	107.50	9.70	Zhang et al., 2009
A3	Apatite	41°08'27.8"	80°00'13.7"	1148	Proterozoic	mica schist	12.09	0.30	81.40	7.40	Zhang et al., 2009
A4	Apatite	41°08'58.2"	80°01'01.6"	1157	Proterozoic	mafic blueschist	10.88	0.42	74.80	7.80	Zhang et al., 2009
A5	Apatite	41°08'58.2"	80°01'01.6"	1157	Proterozoic	quartz schist	11.38	0.29	69.00	6.00	Zhang et al., 2009

A6	Apatite	41°09'23.0"	80°01'48.9"	1144	Proterozoic	mica quartz schist	11.50	0.33	90.00	8.20	Zhang et al., 2009
F1	Apatite	41°13'41"	87°34'50"	1334	Proterozoic	diorite	13.89	0.27	113.70	23.90	Zhu et al., 2007
F2	Apatite	41°15'25"	87°41'18"	1558	Proterozoic	granite	12.16	0.18	109.50	11.20	Zhu et al., 2007
F3	Apatite	41°15'39"	87°41'26"	1575	Upper Proterozoic	schist	11.79	0.14	109.40	10.60	Zhu et al., 2007
F4	Apatite	41°15'09"	87°57'38"	1444	Middle Proterozoic	schist	12.30	0.10	106.30	10.20	Zhu et al., 2007
F5	Apatite	41°14'53"	87°57'07"	1428	Middle Proterozoic	schist	12.48	0.10	98.50	9.20	Zhu et al., 2007
F7	Apatite	41°12'18"	87°56'56"	1386	Middle Proterozoic	schist	12.10	0.14	76.20	7.40	Zhu et al., 2007
F8	Apatite	41°14'00"	87°56'28"	1430	Middle Proterozoic	schist	11.97	0.13	110.70	10.40	Zhu et al., 2007
F9	Apatite	41°15'44"	87°58'23"	1478	Middle Proterozoic	schist	12.17	0.16	70.70	7.40	Zhu et al., 2007
F10	Apatite	41°15'52"	88°00'50"	1505	Proterozoic	schist	12.41	0.20	67.60	6.70	Zhu et al., 2007
F11	Apatite	41°16'57"	88°23'40"	1466	Proterozoic	granite	12.27	0.19	146.00	13.40	Zhu et al., 2007
XD-1	Apatite	41°13'28"	87°56'12"	—	Sinian	clasolite	12.60	1.70	92.00	6.00	Xiao et al., 2011
TK-Q-1	Apatite	40°53'43"	88°18'24"	—	Upper Cambrian	clasolite	12.50	2.00	88.00	6.00	Xiao et al., 2011
TK-Q-2	Apatite	40°53'43"	88°18'24"	—	Middle Ordovician	clasolite	12.60	2.30	100.00	16.00	Xiao et al., 2011
TY-y-1	Apatite	40°44'38"	88°32'25"	—	Upper Ordovician	clasolite	12.20	2.00	79.00	5.00	Xiao et al., 2011
TY-y-2	Apatite	40°44'38"	88°32'25"	—	Upper Ordovician	clasolite	12.80	1.60	84.00	5.00	Xiao et al., 2011
TK-n-1	Apatite	40°44'41"	88°59'31"	—	Sinian	clasolite	12.50	2.00	73.00	6.00	Xiao et al., 2011
TK-X-1	Apatite	40°47'32"	89°25'05"	—	Lower Silurian	clasolite	13.20	2.10	137.00	9.00	Xiao et al., 2011
TK-X-2	Apatite	40°47'32"	89°25'05"	—	Lower Silurian	clasolite	12.80	1.60	100.00	6.00	Xiao et al., 2011
Zk3-1-19	Apatite	37°24'25.92'	96°06'27.79"	3300	Upper Carboniferous	sandstone	12.30	2.60	64.00	3.60	Li ZX et al., 2015
Zk3-1-74	Apatite	37°24'25.92'	96°06'27.79"	3110	Upper Carboniferous	sandstone	12.10	3.20	60.00	5.30	Li ZX et al., 2015
Zk3-1-81	Apatite	37°24'25.92'	96°06'27.79"	3095	—	sandstone	11.80	2.10	61.00	4.20	Li ZX et al., 2015
C1203-10	Apatite	38°14'41.46'	94°34'11.64"	3808	—	phyllite	11.40	2.20	58.00	7.10	Li ZX et al., 2015
C1203-12	Apatite	38°14'42.09'	94°34'12.29"	3787	—	phyllite	10.30	3.00	39.00	5.70	Li ZX et al., 2015
C1203-18	Apatite	38°14'43.68'	94°34'13.35"	3763	—	gabbro	10.80	2.20	75.00	8.80	Li ZX et al., 2015
C1203-39	Apatite	38°14'46.62'	94°34'15.46"	3712	—	andesite basalt	10.90	2.20	41.00	5.80	Li ZX et al., 2015
hc-13	Apatite	35°38'50"	110°09'43"	1186	Upper Triassic	sandstone	12.94	2.00	36.00	3.00	Huang et al., 2016
hc-18	Apatite	35°39'17"	110°08'39"	1362	Upper Triassic	sandstone	13.40	1.80	38.00	4.00	Huang et al., 2016
hc-19	Apatite	35°39'26"	110°08'35"	1390	Upper Triassic	sandstone	13.00	2.20	38.00	3.00	Huang et al., 2016
hc-22	Apatite	35°39'32"	110°08'17"	1448	Upper Triassic	sandstone	13.93	2.00	45.00	4.00	Huang et al., 2016

in-8	Apatite	33°55'19"	110°15'32"	1054	Upper Triassic	sandstone	13.02	2.00	59.00	4.00	Huang et al., 2016
in-12	Apatite	33°55'02"	110°15'10"	1043	Upper Triassic	sandstone	12.71	1.80	66.00	4.00	Huang et al., 2016
H001	Apatite	42°49'11.7"	89°50'37.1"	—	Middle Jurassic	sandstone	10.31	0.42	92.60	11.60	Zhu et al., 2004
H002	Apatite	42°49'30.3"	89°50'53.2"	—	Middle Jurassic	sandstone	11.54	0.19	126.80	11.30	Zhu et al., 2004
H003	Apatite	42°49'47.3"	89°51'30.9"	—	Upper Jurassic	sandstone	12.36	0.18	96.70	9.00	Zhu et al., 2004
H004	Apatite	42°49'52.1"	89°51'58.4"	—	Lower Cretaceous	sandstone	12.32	0.18	190.20	17.00	Zhu et al., 2004
H005	Apatite	42°50'14.3"	89°52'38.7"	—	Upper Cretaceous	sandstone	12.68	0.21	80.50	8.10	Zhu et al., 2004
KK001	Apatite	43°12'11.1"	90°08'26.6"	—	Lower Jurassic	sandstone	12.89	0.23	101.00	9.10	Zhu et al., 2004
DK001	Apatite	42°30'11.4"	89°44'28.8"	—	Lower-Middle Jurassic	sandstone	13.10	0.24	93.40	8.40	Zhu et al., 2004
D001	Apatite	42°22'41.2"	92°59'21.0"	—	Middle Jurassic	sandstone	13.59	0.18	91.50	8.30	Zhu et al., 2004
Q001	Apatite	43°25'40.8"	91°56'15.1"	—	Upper Jurassic	sandstone	13.36	0.27	79.00	7.60	Zhu et al., 2004
Sy28	Apatite	30°56'24"	112°01'48"	99	Upper Jurassic	sandstone	11.00	0.20	93.20	5.70	Shi XB et al., 2013
Sy23	Apatite	31°14'24"	111°51'00"	139	Upper Triassic	sandstone	—	—	55.60	6.00	Shi XB et al., 2013
Sy21	Apatite	31°10'12"	111°44'24"	415	Upper Triassic	sandstone	—	—	72.30	6.00	Shi XB et al., 2013
TS-2	Apatite	32°03'41.7"	119°00'20.5"	—	Lower Silurian	sandstone	13.20	1.60	76.00	4.00	Yuan et al., 2016
GD-3	Apatite	30°52'14.4"	119°24'20.2"	—	Lower Cretaceous	sandstone	12.60	2.20	105.00	6.00	Yuan et al., 2016
YQ-01	Apatite	40°35'42"	116°14'54"	834	Jurassic	conglomerate	13.20	—	89.90	10.80	Li JF et al., 2010
YQ-02	Apatite	40°40'07"	116°14'33"	832	Jurassic	sandstone	—	—	81.70	16.00	Li JF et al., 2010
YQ-03	Apatite	40°39'48"	116°17'40"	489	Jurassic	andesite	10.80	—	76.00	8.00	Li JF et al., 2010
YQ-04	Apatite	40°40'17"	116°18'48"	491	Jurassic	sandstone	9.80	—	85.70	8.80	Li JF et al., 2010
YQ-05	Apatite	40°41'04"	116°19'21"	468	Jurassic	sandstone	9.40	—	79.70	14.20	Li JF et al., 2010
YQ-06	Apatite	40°42'03"	116°23'14"	457	Jurassic	sandstone	7.20	—	82.60	9.60	Li JF et al., 2010
YQ-07	Apatite	40°42'07"	116°23'16"	486	Jurassic	sandstone	7.70	—	64.50	7.10	Li JF et al., 2010
YQ-08	Apatite	40°42'04"	116°23'18"	489	Jurassic	sandstone	—	—	62.40	6.90	Li JF et al., 2010
430	Apatite	29°36'37.7"	120°21'03.0"	—	Upper Proterozoic	diorite	12.90	—	62.00	4.00	Wang F et al., 2015
431	Apatite	29°36'35.4"	120°21'05.5"	—	Upper Proterozoic	diorite	12.20	—	41.00	3.00	Wang F et al., 2015
432	Apatite	29°36'35.4"	120°21'05.5"	—	Upper Proterozoic	diorite	12.10	—	45.00	3.00	Wang F et al., 2015
43	Apatite	29°42'0.1"	120°25'11.6"	—	Upper Proterozoic	diorite	13.00	—	47.00	5.00	Wang F et al., 2015
44	Apatite	29°41'28.5"	120°25'06.4"	—	Upper Proterozoic	diorite	13.10	—	24.00	2.00	Wang F et al., 2015
434	Apatite	29°35'10.8"	120°21'57.9"	—	Upper Proterozoic	gneiss	13.00	—	63.00	4.00	Wang F et al., 2015

435	Apatite	29°34'48.4"	120°22'57.5"	—	Upper Proterozoic	gneiss	12.90	—	56.00	3.00	Wang F et al., 2015
436	Apatite	29°34'47.7"	120°23'24.6"	—	Upper Proterozoic	gneiss	12.30	—	66.00	4.00	Wang F et al., 2015
437	Apatite	29°34'43.6"	120°23'33.2"	—	Upper Proterozoic	gneiss	13.00	—	46.00	3.00	Wang F et al., 2015
438	Apatite	29°34'44.4"	120°23'52.1"	—	Upper Proterozoic	gneiss	12.80	—	53.00	4.00	Wang F et al., 2015
433	Apatite	29°35'30.4"	120°21'07.9"	—	Upper Proterozoic	mylonite	12.50	—	57.00	4.00	Wang F et al., 2015
42	Apatite	29°41'26.0"	120°27'13.5"	—	Upper Proterozoic	mylonite	12.60	—	44.00	4.00	Wang F et al., 2015
45	Apatite	29°41'31.8"	120°26'41.5"	—	Upper Proterozoic	mylonite	13.00	—	41.00	4.00	Wang F et al., 2015
46	Apatite	29°41'24.4"	120°26'21.1"	—	Upper Proterozoic	mylonite	13.00	—	45.00	3.00	Wang F et al., 2015
47	Apatite	29°41'23.2"	120°25'46.4"	—	Upper Proterozoic	mylonite	12.80	—	41.00	3.00	Wang F et al., 2015
Nx1	Apatite	25°12'45"	114°14'59"	188	Jurassic	granite	—	—	39.90	3.00	Yan Yi et al., 2009
Nx2	Apatite	25°13'23"	114°14'16"	208	Jurassic	granite	13.26	0.16	45.90	1.40	Yan Yi et al., 2009
Nx3	Apatite	25°15'23"	114°13'11"	221	Jurassic	granodiorite	—	—	37.30	2.30	Yan Yi et al., 2009
Nx4	Apatite	25°15'23"	114°13'11"	342	Jurassic	granite	13.84	0.14	54.20	1.60	Yan Yi et al., 2009
Fg3	Apatite	23°57'39"	113°36'22"	131	Jurassic	granite	13.88	0.15	38.50	1.70	Yan Yi et al., 2009
Hy1	Apatite	23°53'23"	114°47'59"	375	Jurassic	granite	—	—	46.20	4.60	Yan Yi et al., 2009
Hy2	Apatite	23°54'35"	114°49'23"	480	Jurassic	granite	—	—	43.90	3.30	Yan Yi et al., 2009
Hy4	Apatite	23°46'36"	114°45'75"	260	Jurassic	granite	13.33	0.19	40.00	3.00	Yan Yi et al., 2009
Hy5	Apatite	23°46'36"	114°45'75"	260	Jurassic	granodiorite	13.86	0.18	41.50	3.00	Yan Yi et al., 2009
Hy9	Apatite	23°46'48"	114°39'11"	550	Jurassic	granodiorite	—	—	40.40	4.20	Yan Yi et al., 2009
Hz1	Apatite	22°49'52"	114°57'28"	109	Jurassic	granite	12.55	0.12	60.90	3.60	Yan Yi et al., 2009
Hz3	Apatite	22°50'22"	114°58'32"	149	Jurassic	granite	13.78	0.11	59.80	3.10	Yan Yi et al., 2009
Hz4	Apatite	22°50'16"	115°01'74"	143	Jurassic	granite	13.95	0.19	54.20	4.70	Yan Yi et al., 2009
Hz6	Apatite	23°16'38"	114°01'47"	1120	Jurassic	granite	13.26	0.16	51.40	3.10	Yan Yi et al., 2009
Hz7	Apatite	23°16'36"	114°01'53"	984	Jurassic	granite	13.59	0.12	56.10	2.90	Yan Yi et al., 2009
Hz8	Apatite	23°15'12"	114°02'23"	212	Jurassic	granite	13.45	0.12	56.30	3.00	Yan Yi et al., 2009
Hz9	Apatite	23°14'02"	114°01'08"	250	Jurassic	granite	13.64	0.14	53.30	3.00	Yan Yi et al., 2009
L1	Apatite	36°21'16.9"	117°42'06.0"	243	Archaeozoic	granite	8.50	1.76	31.90	3.3	Yang et al., 2008
L2	Apatite	36°21'21.2"	117°42'18.9"	258	Archaeozoic	granite	12.17	0.36	32.10	2.9	Yang et al., 2008
L3	Apatite	36°20'26.2"	117°43'46.0"	247	Archaeozoic	granite	9.58	0.53	35.20	3.8	Yang et al., 2008
L4	Apatite	36°19'10.5"	117°47'08.0"	283	Archaeozoic	granite	9.88	0.27	27.60	2.5	Yang et al., 2008

L5	Apatite	36°17'20.2"	117°46'35.0"	359	Archaeozoic	granite	13.71	0.20	24.60	2.2	Yang et al., 2008
L6	Apatite	36°15'50.3"	117°45'26.0"	264	Archaeozoic	granite	11.93	0.46	25.90	2.9	Yang et al., 2008
L7	Apatite	36°14'19.1"	117°44'44.3"	227	Archaeozoic	granite	12.07	0.42	30.60	3.1	Yang et al., 2008
L8	Apatite	36°11'22.4"	117°47'08.0"	230	Archaeozoic	granite	11.01	0.74	36.60	4.2	Yang et al., 2008
MS-1	Apatite	35°32'45"	117°56'14"	980	Archaeozoic	granite	—	—	53.00	6.00	Tang et al., 2011
MS-4	Apatite	35°33'23"	117°56'52"	751	Archaeozoic	granite	13. 6	1. 9	64.00	5.00	Tang et al., 2011
MS-5	Apatite	35°33'25"	117°56'59"	695	Archaeozoic	granite	13. 5	2. 1	56.00	4.00	Tang et al., 2011
MS-7	Apatite	35°33'26"	117°57'05"	660	Archaeozoic	granite	13. 4	1. 9	63.00	4.00	Tang et al., 2011
MS-8	Apatite	35°33'31"	117°57'28"	590	Archaeozoic	granite	14. 0	1. 8	47.00	3.00	Tang et al., 2011
DLJ-1	Apatite	27°46'36"	98°26'57"	3326	—	granite	13.6	0.2	6.80	0.50	Lei et al., 2008a
DLJ-5	Apatite	27°48'02"	98°27'16"	3193	—	granite	13.8	0.2	6.30	0.90	Lei et al., 2008a
DLJ-7	Apatite	27°49'54"	98°27'04"	3008	—	granite	13.8	0.2	6.40	0.80	Lei et al., 2008a
DLJ-9	Apatite	27°50'56"	98°27'12"	2777	—	granite	13.9	0.3	6.40	1.10	Lei et al., 2008a
DLJ-11	Apatite	27°52'42"	98°25'14"	2442	—	granite	13.7	0.4	5.00	1.00	Lei et al., 2008a
DLJ-13	Apatite	27°54'23"	98°24'17"	2158	—	granite	13.1	0.2	5.50	1.10	Lei et al., 2008a
DLJ-14	Apatite	27°53'47"	98°23'05"	2049	—	granite	14.1	0.3	4.90	0.60	Lei et al., 2008a
DLJ-21	Apatite	27°54'14"	98°21'00"	1821	—	granite	13.7	0.4	4.80	0.80	Lei et al., 2008a
DLJ-19	Apatite	27°53'57"	98°21'20"	1587	Triassic-Jurassic	granite	13.1	0.6	4.00	1.00	Lei et al., 2008a
S42	Apatite	24°29'31"	100°17'42"	1034	Triassic-Jurassic	granite	13.24	0.13	15.40	0.70	Shi et al., 2006
S51	Apatite	23°49'59"	100°03'36"	1650	Triassic-Jurassic	granite	13.34	0.14	14.70	1.10	Shi et al., 2006
S53	Apatite	23°35'42"	100°01'55"	1981	Triassic-Jurassic	granite	12.26	0.31	15.60	2.40	Shi et al., 2006
S57	Apatite	23°57'50"	100°05'56"	1446	Triassic-Jurassic	granite	13.13	0.17	15.50	1.30	Shi et al., 2006
S61	Apatite	23°48'14"	100°14'56"	1462	Triassic-Jurassic	granite	14.56	0.17	20.60	0.90	Shi et al., 2006
S62	Apatite	22°01'52"	100°31'30"	1487	Triassic-Jurassic	granite	13.58	0.12	25.90	0.80	Shi et al., 2006
DXL-01	Apatite	29°29'18"	94°56'54"	4210	—	gly metamorphosed granito	11.10	2.40	3.60	0.40	Xu et al., 2011
DXL-02	Apatite	29°29'25"	94°57'20"	4160	—	gly metamorphosed granito	12.00	2.70	3.90	0.60	Xu et al., 2011
DXL-03	Apatite	29°29'08"	94°57'23"	4014	—	gly metamorphosed granito	12.20	2.40	4.60	0.60	Xu et al., 2011
DXL-05	Apatite	29°28'31"	94°58'46"	3575	—	ghtly metamorphosed granit	12.00	2.60	3.20	0.40	Xu et al., 2011
DXL-06	Apatite	29°28'14"	95°00'00"	3276	—	ghtly metamorphosed granit	11.20	2.10	2.80	0.40	Xu et al., 2011
DXL-07	Apatite	29°27'02"	95°01'12"	3042	—	ghtly metamorphosed granit	11.00	2.30	2.70	0.40	Xu et al., 2011

DXL-10	Apatite	29°22'04"	95°07'37"	2190	—	ghtly metamorphosed granit	11.80	2.40	1.70	0.30	Xu et al., 2011
DXL-12	Apatite	29°21'09"	95°09'12"	1784	—	ghtly metamorphosed granit	—	—	2.40	0.50	Xu et al., 2011
DXL-15	Apatite	29°18'30"	95°10'36"	944	—	ghtly metamorphosed granit	11.40	2.00	2.20	0.40	Xu et al., 2011
DXL-19	Apatite	29°14'51"	95°09'52"	710	—	ghtly metamorphosed granit	12.40	2.20	1.70	0.30	Xu et al., 2011
NW01	Apatite	38°51'18"	112°11'03"	1759	Triassic	sandstone	12.60	2.00	70.00	5.00	Zhao JF et al., 2016
NW02	Apatite	38°45'25"	112°12'21"	1575	Jurassic	sandstone	12.80	2.00	75.00	5.00	Zhao JF et al., 2016
NW03	Apatite	38°43'55"	112°12'27"	1610	Jurassic	sandstone	12.70	2.50	70.00	5.00	Zhao JF et al., 2016
NW05	Apatite	38°40'30"	112°20'31"	1725	Cretaceous	granite	13.20	1.80	94.00	7.00	Zhao JF et al., 2016
NW07	Apatite	38°40'29"	112°21'14"	1745	Cretaceous	granite	12.90	2.00	111.00	8.00	Zhao JF et al., 2016
NW09	Apatite	38°51'34"	112°04'47"	1713	Cambrian-Ordovician	monzonite	13.10	2.10	49.00	4.00	Zhao JF et al., 2016
NW10	Apatite	38°54'09"	111°55'23"	2108	Cambrian-Ordovician	mudstone	12.60	2.00	126.00	9.00	Zhao JF et al., 2016
NW11	Apatite	38°53'34"	111°53'19"	1554	Cambrian-Ordovician	monzonite	12.90	1.80	120.00	8.00	Zhao JF et al., 2016
LF01	Apatite	38°01'16"	111°40'18"	1300	Archaeozoic	diabase	12.30	2.00	120.00	14.00	Zhao JF et al., 2016
LF03	Apatite	38°00'38"	111°21'43"	1323	Archaeozoic	granite	12.80	2.10	111.00	7.00	Zhao JF et al., 2016
LF05	Apatite	37°56'44"	111°47'00"	1629	Archaeozoic	diabase	13.40	1.70	110.00	7.00	Zhao JF et al., 2016
LF06	Apatite	37°57'13"	112°04'16"	1042	Jurassic	sandstone	13.50	2.00	42.00	3.00	Zhao JF et al., 2016
NX-70	Apatite	37°00'17"	105°14'54"	1773	Permian	sandstone	12.50	1.80	67.00	5.00	Zhao XC et al., 2016
NX-80	Apatite	37°09'21"	105°24'21"	1738	Carboniferous	sandstone	13.00	1.50	68.00	5.00	Zhao XC et al., 2016
NX-96	Apatite	37°13'35"	105°13'36"	1383	Devonian	sandstone	12.50	2.30	71.00	5.00	Zhao XC et al., 2016
NX-94	Apatite	37°19'02"	105°27'28"	1553	Devonian	sandstone	12.60	2.10	95.00	8.00	Zhao XC et al., 2016
AH1	Apatite	36°21'04"	119°12'06"	119	—	fault gouge	12.24	2.10	69.80	6.60	Wang et al., 2007
AH3	Apatite	36°21'04"	119°12'06"	119	—	cataclasite	12.15	1.96	63.30	5.40	Wang et al., 2007
AH4	Apatite	36°21'55"	119°10'50"	97	Cretaceous	glutenite	12.61	2.40	121.00	9.00	Wang et al., 2007
WL1	Apatite	36°08'22"	119°12'41"	121	Cretaceous	glutenite	12.94	1.66	67.00	4.60	Wang et al., 2007
WL3	Apatite	36°17'49"	118°47'02"	198	—	cataclasite	12.37	1.88	63.30	4.30	Wang et al., 2007
WL4	Apatite	36°18'02"	118°47'32"	196	Cretaceous	volcanics	12.24	1.72	95.70	8.90	Wang et al., 2007
WL5	Apatite	36°18'04"	118°48'29"	171	Cretaceous	glutenite	13.31	1.68	70.50	5.70	Wang et al., 2007
WL7	Apatite	36°26'40"	118°50'21"	150	—	fault gouge	12.95	1.77	72.40	6.70	Wang et al., 2007
WL9	Apatite	36°25'58"	118°51'54"	146	—	cataclasite	13.82	1.25	70.00	4.20	Wang et al., 2007
WL10	Apatite	36°27'14"	118°52'00"	159	Cretaceous	glutenite	12.57	2.04	107.00	11.00	Wang et al., 2007

WL11	Apatite	36°24'24"	118°47'25"	178	Proterozoic	syenite	12.39	1.44	116.00	8.00	Wang et al., 2007
LM6	Apatite	36°03'36"	118°56'31"	186	Cretaceous	volcanics	12.51	1.95	137.00	15.00	Wang et al., 2007
LM7	Apatite	36°08'42"	118°57'52"	193	Carboniferous	intrusives	12.78	1.66	90.90	8.50	Wang et al., 2007
LM10	Apatite	36°01'49"	119°07'31"	151	Cretaceous	glutenite	11.98	2.07	104.00	7.00	Wang et al., 2007
BS02	Apatite	36°17'49"	118°47'02"	198	—	fault gouge	12.38	1.86	65.20	3.50	Wang et al., 2007
AL-1	Apatite	24°06'16"	101°30'44"	944	—	granitegneiss	10.60	2.70	7.90	1.10	Li et at., 2012
AL-4	Apatite	24°01'33"	101°32'28"	1695	—	granitegneiss	11.50	2.10	8.40	1.20	Li et at., 2012
DCS-5	Apatite	25°41'46"	100°06'33"	3051	Carboniferous-Permian	mylonite	13.60	2.00	6.60	1.30	Li et at., 2012
09YSD-L1	Apatite	40°46'30.0"	104°50'15.4"		Carboniferous-Permian	sandstone	13.20	2.10	149.00	9.00	Han et al., 2015
09YSH-L1	Apatite	40°49'29.7"	104°44'50.9"		Carboniferous-Permian	sandstone	12.90	2.30	143.00	12.00	Han et al., 2015
09YCH-L2	Apatite	40°43'14.1"	104°48'00.2"		Carboniferous-Permian	sandstone	12.50	2.00	151.00	11.00	Han et al., 2015
91-SI6	Apatite	40°48.3'	79°50.2'	1370	—	—	—	—	226.00	1.00	Dumitru et al., 2001
91-SU1	Apatite	40°48.3'	79°50.0'	1370	—	—	—	—	240.00	21.00	Dumitru et al., 2001
91-SU2	Apatite	40°50.4'	79°52.7'	1370	—	—	—	—	211.00	16.00	Dumitru et al., 2001
91-SH5	Apatite	40°50.4'	79°52.7'	1370	—	—	—	—	185.00	12.00	Dumitru et al., 2001
91-SH6	Apatite	40°50.4'	79°52.7'	1370	—	—	—	—	238.00	17.00	Dumitru et al., 2001
91-SI4	Apatite	40°54.9'	79°54.1'	1370	—	—	—	—	222.00	12.00	Dumitru et al., 2001
91-SH2	Apatite	40°55.1'	79°50.8'	1370	—	—	—	—	219.00	23.00	Dumitru et al., 2001
91-SU8	Apatite	40°55.1'	79°50.8'	1370	—	—	—	—	168.00	11.00	Dumitru et al., 2001
K-1	Apatite	42°06'30"	83°06'00"	1980	—	—	—	—	103.30	16.00	Dumitru et al., 2001
K-2	Apatite	42°09'30"	83°06'50"	1980	—	—	—	—	197.10	15.00	Dumitru et al., 2001
K-3	Apatite	42°09'30"	83°06'30"	1980	—	—	—	—	50.00	4.40	Dumitru et al., 2001
K-4	Apatite	42°14'20"	83°14'00"	1980	—	—	—	—	96.70	12.00	Dumitru et al., 2001
K-5	Apatite	42°14'30"	83°14'30"	1980	—	—	—	—	96.70	9.30	Dumitru et al., 2001
K-6	Apatite	42°15'30"	83°15'20"	1980	—	—	—	—	67.10	5.50	Dumitru et al., 2001
K-7	Apatite	42°17'20"	83°16'30"	1980	—	—	—	—	86.30	5.40	Dumitru et al., 2001
DK21	Apatite	44°05.0'	84°42.1'	1220	Carboniferous	volcanics	—	—	227.20	28.00	Dumitru et al., 2001
DK23	Apatite	43°46.0'	84°28.1'	2800	—	intrusives	—	—	38.70	3.50	Dumitru et al., 2001
DK24	Apatite	43°46.0'	84°28.1'	2800	—	intrusives	—	—	15.30	2.10	Dumitru et al., 2001
DK25	Apatite	43°45.7'	84°27.0'	3360	—	—	—	—	32.00	3.50	Dumitru et al., 2001

DK26	Apatite	43°44.6'	84°26.2'	3200	—	—	—	—	28.60	2.20	Dumitru et al., 2001
DK27	Apatite	43°44.4'	84°25.5'	3440	—	—	—	—	12.00	0.50	Dumitru et al., 2001
DK28	Apatite	43°44.4'	84°25.5'	3440	—	—	—	—	10.70	1.40	Dumitru et al., 2001
DK29	Apatite	43°43.8'	84°25.8'	3760	—	—	—	—	23.40	1.10	Dumitru et al., 2001
DK30	Apatite	43°43.7'	84°25.3'	3480	—	—	—	—	22.40	1.50	Dumitru et al., 2001
DK32	Apatite	43°43.2'	84°25.9'	2920	—	intrusives	—	—	106.10	4.50	Dumitru et al., 2001
DK33	Apatite	43°42.2'	84°27.0'	3080	—	intrusives	—	—	113.00	3.50	Dumitru et al., 2001
DK34	Apatite	43°39.4'	84°19.4'	2560	Carboniferous	volcanics	—	—	173.50	21.00	Dumitru et al., 2001
DK36	Apatite	43°30.6'	84°27.4'	3010	Jurassic	siliciclastics	—	—	22.70	6.80	Dumitru et al., 2001
DK37	Apatite	43°29.5'	84°27.1'	3280	—	intrusives	—	—	9.10	6.50	Dumitru et al., 2001
DK41	Apatite	43°28.6'	84°27.0'	3360	—	intrusives	—	—	35.80	3.00	Dumitru et al., 2001
DK43	Apatite	43°23.2'	84°23.2'	2400	Carboniferous	volcanics	—	—	244.50	7.30	Dumitru et al., 2001
DK44	Apatite	43°23.0'	84°23.2'	2280	Carboniferous	volcanics	—	—	208.30	11.00	Dumitru et al., 2001
DK45	Apatite	43°17.4'	84°18.9'	1760	Carboniferous	volcanics	—	—	275.40	7.90	Dumitru et al., 2001
DK52	Apatite	43°11.3'	84°17.3'	2560	—	intrusives	—	—	125.30	5.40	Dumitru et al., 2001
J4	Apatite	43°11.3'	84°17.3'	2560	—	intrusives	—	—	135.70	9.70	Dumitru et al., 2001
J5	Apatite	43°11.3'	84°17.3'	2560	—	intrusives	—	—	110.30	6.60	Dumitru et al., 2001
J9	Apatite	43°11.1'	84°17.4'	2600	—	intrusives	—	—	106.10	4.80	Dumitru et al., 2001
DK53	Apatite	43°10.9'	84°17.6'	2640	—	intrusives	—	—	70.80	10.00	Dumitru et al., 2001
DK55	Apatite	43°00.1'	84°09.8'	2710	—	intrusives	—	—	204.90	8.40	Dumitru et al., 2001
DK61	Apatite	42°59.9'	84°08.9'	2660	Carboniferous	volcanics	—	—	196.60	7.80	Dumitru et al., 2001
DK62	Apatite	42°32.7'	83°33.6'	2960	Carboniferous	carbonates/siliciclastics	—	—	159.00	48.00	Dumitru et al., 2001
DK68	Apatite	42°19.7'	83°11.7'	2000	Silurian	carbonates/siliciclastics	—	—	52.20	6.10	Dumitru et al., 2001
NJ11-001	Apatite	42°03.563'	81°30.611'	1824	Lower Triassic	sandstone	—	—	30.10	5.30	Yang et al., 2017
NJ11-007	Apatite	42°02.549'	81°31.245'	1775	Middle-Upper Triassic	sandstone	—	—	13.70	1.70	Yang et al., 2017
NJ11-008	Apatite	42°01.896'	81°31.786'	1756	Middle-Upper Triassic	sandstone	—	—	14.20	1.80	Yang et al., 2017
NJ11-009	Apatite	42°01.358'	81°31.878'	1744	Lower Jurassic	sandstone	—	—	11.90	2.00	Yang et al., 2017
NJ11-010	Apatite	42°00.754'	81°32.234'	1741	Middle Jurassic	sandstone	—	—	24.90	2.30	Yang et al., 2017
NJ11-011	Apatite	41°59.996'	81°32.278'	1715	Upper Jurassic	sandstone	—	—	74.20	10.10	Yang et al., 2017
NJ11-012	Apatite	41°59.545'	81°32.359'	1711	Lower Cretaceous	sandstone	—	—	76.70	7.90	Yang et al., 2017

NJ11-013	Apatite	41°58.341'	81°32.120'	1667	Upper Cretaceous	sandstone	—	—	98.40	23.10	Yang et al., 2017
NJ11-014	Apatite	41°58.139'	81°32.425'	1669	Palaeocene-Eocene	sandstone	—	—	104.40	13.00	Yang et al., 2017
NJ11-015	Apatite	41°58.204'	81°32.813'	1679	Oligocene	sandstone	—	—	67.70	4.90	Yang et al., 2017
NJ11-016	Apatite	41°57.641'	81°32.779'	1670	Palaeocene	sandstone	—	—	59.60	9.70	Yang et al., 2017
NJ11-018	Apatite	41°01.905'	81°32.966'	1636	Pleistocene	sandstone	—	—	46.20	4.60	Yang et al., 2017
T101-2	Apatite	28°18.08'	85°20.90'	2000	Pre-Sinian	plagiogranite	—	—	1.10	0.70	Li et al., 2013
T101-3	Apatite	28°21.83'	85°21.06'	2500	Pre-Sinian	plagiogranite	—	—	1.30	0.90	Li et al., 2013
T101-4	Apatite	28°23.70'	85°21.03'	2802	Pre-Sinian	granite	—	—	1.50	0.70	Li et al., 2013
T101-5	Apatite	28°25.10'	85°13.87'	3100	Pre-Sinian	granite	—	—	1.90	0.50	Li et al., 2013
T101-7	Apatite	28°31'36	85°13.00'	3371	Neogene	granite	—	—	1.50	0.70	Li et al., 2013
14YC03	Apatite	47.484810°	129.372217°	227	Jurassic	granite	—	—	132	8.4	Chen, 2016
14YC04	Apatite	47.365684°	129.549314°	220	Jurassic	granite	—	—	135.1	6.5	Chen, 2016
14YC05	Apatite	47.383196°	129.983080°	214	Upper Permian	diorite	12.78	0.12	143.1	9.1	Chen, 2016
10HC02	Apatite	47.167167°	130.868361°	81	Pre-Jurassic	diorite	—	—	43.7	2.4	Chen, 2016
10HC04	Apatite	47.114611°	130.827972°	79	Ordovician	granodiorite	14.68	0.09	50.4	2.3	Chen, 2016
14FJ01	Apatite	47.034824°	131.720510°	86	Pre-Jurassic	granite	—	—	46.8	3.2	Chen, 2016
14FJ02	Apatite	46.961312°	131.679236°	76	Ordovician	granodiorite	—	—	49.1	3.4	Chen, 2016
14FJ03	Apatite	47.354564°	132.487776°	49	Pre-Jurassic	diorite	—	—	61	2.2	Chen, 2016
14RH03	Apatite	46.869870°	133.850260°	111	Lower Cretaceous	granite	—	—	93.3	3.5	Chen, 2016
14RH04	Apatite	47.070630°	133.911230°	124	Cretaceous	granodiorite	14.8	0.07	75.2	3.4	Chen, 2016
14RH05	Apatite	46.957878°	133.812256°	185	Cretaceous	granodiorite	—	—	74.4	5.1	Chen, 2016
JX11	Apatite	45.337861°	130.903944°	201	Cretaceous	sandstone	—	—	84.6	6	Chen, 2016
JX12	Apatite	45.335917°	130.929583°	216	Cretaceous	sandstone	—	—	70.2	5.3	Chen, 2016
JX14	Apatite	45.337139°	130.942583°	205	Cretaceous	sandstone	—	—	67.4	4.9	Chen, 2016
10JX12	Apatite	45.129347°	130.723767°	395	Cretaceous	sandstone	13.9	0.11	93	5.9	Chen, 2016
14SFH01	Apatite	44.436015°	130.869396°	320	Jurassic	granodiorite	13.92	0.09	174.5	9.7	Chen, 2016
14SFH02	Apatite	44.432270°	130.765582°	408	Permian	granite	—	—	168	16.1	Chen, 2016
14SFH03	Apatite	44.388930°	130.490642°	542	Jurassic	granodiorite	—	—	123.9	6	Chen, 2016
14SZ01	Apatite	44.629387°	129.325004°	296	Jurassic	granite	—	—	167.7	11	Chen, 2016
14SZ03	Apatite	44.930132°	128.912692°	396	Jurassic	granite	13.87	0.12	140	7.1	Chen, 2016

14SZ04	Apatite	45.078113°	128.157053°	344	Jurassic	granite	—	—	150.7	12.9	Chen, 2016
14SZ05	Apatite	45.388816°	127.686335°	378	Jurassic	granite	—	—	115.9	10.4	Chen, 2016
HS03	Apatite	30°08'41.6"	118°09'59.4"	1599	Jurassic-Lower Cretaceous	granite	11.1	2.6	30	3	Zheng et al., 2011
HS04	Apatite	30°08'03.5"	118°09'51.1"	1817	Jurassic-Lower Cretaceous	granite	11.2	1.4	15	3	Zheng et al., 2011
HS08	Apatite	30°07'33.1"	118°09'33.1"	1689	Jurassic-Lower Cretaceous	granite	12.1	2.3	41	4	Zheng et al., 2011
HS12	Apatite	30°07'23.3"	118°10'15.4"	1591	Jurassic-Lower Cretaceous	granite	11.9	1.9	45	6	Zheng et al., 2011
HS14	Apatite	30°06'49.6"	118°10'15.0"	1132	Jurassic-Lower Cretaceous	granite	12.5	2.2	42	3	Zheng et al., 2011
HS15	Apatite	30°06'33.4"	118°10'07.3"	914	Jurassic-Lower Cretaceous	granite	12.6	2.4	32	3	Zheng et al., 2011
HS19	Apatite	30°06'25.5"	118°09'45.5"	1099	Jurassic-Lower Cretaceous	granite	—	—	46	5	Zheng et al., 2011
HS21	Apatite	30°06'17.4"	118°11'17.3"	827	Jurassic-Lower Cretaceous	granite	11.9	2.3	56	6	Zheng et al., 2011
HS22	Apatite	30°11'40.9"	118°08'40.5"	289	Jurassic-Lower Cretaceous	granite	11.7	2	39	2	Zheng et al., 2011
FJS-16	Apatite	27°54.756'	108°39.830'	2009	Proterozoic	pyroclastic rocks	12	2.1	35	3	Tang et al., 2014
FJS-39	Apatite	27°54'29"	108°39'10"	1880	Proterozoic	pyroclastic rocks	12	1.4	64	5	Tang et al., 2014
BXCS020-1	Apatite	27°57'51"	109°35'10"	375	Lower Cretaceous	sandstones	13.2	2.3	67	5	Tang et al., 2014
HH53-2	Apatite	27°54'22"	109°50'25"	181	Upper Cretaceous	siltstones	12.1	2.2	62	6	Tang et al., 2014
HH49-2	Apatite	27°45'16"	109°52'41"	219	Upper Cretaceous	sandstones	12.5	1.9	52	4	Tang et al., 2014
HH04-1	Apatite	27°32'50"	109°49'02"	223	Lower Cretaceous	sandstones	11.7	2	58	3	Tang et al., 2014
HH03-1	Apatite	27°32'59"	109°50'14"	238	Upper Cretaceous	siltstones	12.3	1.9	37	3	Tang et al., 2014
HH02-1	Apatite	27°32'59"	109°54'54"	263	Middle Jurassic	sandstones	11.8	2.4	46	5	Tang et al., 2014
HH14-1	Apatite	27°19'33"	109°45'48"	309	Upper Cretaceous	sandstones	12.4	2.1	35	2	Tang et al., 2014
HH38-1	Apatite	27°43'06"	110°09'13"	198	Middle Jurassic	siltstones	12.2	1.8	71	5	Tang et al., 2014
HH43-1	Apatite	27°45'35"	110°05'20"	177	Lower Cretaceous	siltstones	12.1	1.9	60	4	Tang et al., 2014
HH27-5	Apatite	27°27'09"	110°25'08"	657	Upper Triassic	diorite	12.3	2	49	3	Tang et al., 2014
HH28-3	Apatite	27°27'22"	110°25'33"	723	Upper Triassic	diorite	12.5	1.7	44	3	Tang et al., 2014
HH29-5	Apatite	27°24'59"	110°27'55"	549	Upper Triassic	diorite	12	2.1	43	3	Tang et al., 2014
I-03	Apatite	25°44'12.3"	113°09'47.9"	212	Jurassic-Lower Cretaceous	granite	12.8	2.2	28	3	Wan, 2013
I-08	Apatite	25°44'56.7"	113°09'55.6"	389	Jurassic-Lower Cretaceous	granite	12.7	2.3	36	4	Wan, 2013
H01	Apatite	19°20.060'	110°36.239'	42	Middle Jurassic	syenogranite	13.09	0.4	23.5	4.3	Shi et al., 2011
H02	Apatite	18°48.648'	110°18.325'	77	Lower Triassic	syenogranite	13.48	0.13	28.8	3.2	Shi et al., 2011
H04	Apatite	18°38.621'	109°57.281'	73	Permian-Triassic	monzonitic granite	13.66	0.13	28.7	2.3	Shi et al., 2011

H05	Apatite	18°40.404'	109°56.227'	124	Middle Jurassic	monzonitic granite	13.35	0.16	23.9	2.4	Shi et al., 2011
H06	Apatite	18°39.540'	109°57.162'	85	Permian-Triassic	monzonitic granite	13.51	0.16	29.1	2.8	Shi et al., 2011
H08	Apatite	18°31.073'	109°57.196'	41	Upper Cretaceous	granodiorite	13.68	0.12	23.9	2.2	Shi et al., 2011
H11	Apatite	18°26.735'	108°58.512'	68	Upper Cretaceous	monzonitic granite	13.43	0.28	26.3	2.6	Shi et al., 2011
H12	Apatite	18°42.726'	109°12.173'	216	Lower Permian	monzonitic granite	13.18	0.25	36.6	4	Shi et al., 2011
H13	Apatite	18°35.522'	109°06.129'	197	Upper Cretaceous	monzonitic granite	12.96	0.36	30.8	6.5	Shi et al., 2011
H14	Apatite	18°40.808'	108°44.367'	75	Permian-Jurassic	monzonitic granite	13.28	0.13	32.3	2.3	Shi et al., 2011
H15	Apatite	19°02.873'	108°47.519'	107	Lower Triassic	monzonitic granite	13.85	0.12	26.1	2.5	Shi et al., 2011
SY6-Ap	Apatite	18°53'13"	109°36'05"	481	Permian	granite	13.33	0.21	28.7	1.7	Yan et al., 2011
SY10-Ap	Apatite	18°54'17"	109°39'07"	632	Lower Cretaceous	granodiorite	13.65	0.23	22.9	1.7	Yan et al., 2011
SY11-Ap	Apatite	18°52'36"	109°38'31"	614	Permian	granite	—	—	23.2	1.7	Yan et al., 2011
SY15-Ap	Apatite	18°53'44"	109°30'41"	329	Permian	diorite	12.8	0.16	34.6	1.5	Yan et al., 2011
SY16-Ap	Apatite	19°03'39"	109°50'18"	240	Permian	granodiorite	13.02	0.23	35.2	1.6	Yan et al., 2011
SY17-Ap	Apatite	19°02'30"	109°48'08"	250	Permian	granodiorite	14.68	0.33	32.7	2	Yan et al., 2011
DF2-Ap	Apatite	18°14'38"	109°18'31"	164	Cretaceous	granite	12.68	0.23	45.5	3.9	Yan et al., 2011
DF4-Ap	Apatite	18°14'47"	109°18'52"	150	Cretaceous	granodiorite	12.209	0.15	32.3	2.1	Yan et al., 2011
DF11-Ap	Apatite	18°08'10"	109°28'32"	93	Cretaceous	granite	12.32	0.39	46.8	6.5	Yan et al., 2011
DF17-Ap	Apatite	18°21'47"	109°38'52"	364	Cretaceous	granite	12.52	0.15	43.7	2.9	Yan et al., 2011
DF18-Ap	Apatite	18°14'39"	109°40'40"	345	Cretaceous	granite	12.65	0.14	48.9	4	Yan et al., 2011
DF20-Ap	Apatite	18°21'45"	109°43'38"	320	Cretaceous	granite	12.02	0.18	39.9	3.2	Yan et al., 2011
Q1	Apatite	34°07.727'	109°25.628'	—	—	granite	13.5	1.1	27.9	106	Enkelmann et al., 2006
Q2	Apatite	34°03.750'	109°29.678'	—	—	syenite	—	—	43.6	2.1	Enkelmann et al., 2006
Q3	Apatite	34°02.519'	109°35.062'	—	Cretaceous	gneiss	12.3	1.7	56.5	3.5	Enkelmann et al., 2006
Q4	Apatite	33°31.080'	110°52.611'	—	Cretaceous	metavolcanic	12.1	1.1	170	9	Enkelmann et al., 2006
Q8	Apatite	33°26.081'	110°53.764'	—	Cretaceous	metavolcanic	12.2	1.4	143	7	Enkelmann et al., 2006
Q12	Apatite	33°52.771'	110°58.968'	—	Cretaceous	gneiss	—	—	42.3	2.6	Enkelmann et al., 2006
Q13	Apatite	33°51.222'	110°28.842'	—	Cretaceous	sandstone	—	—	42	2.6	Enkelmann et al., 2006
Q20	Apatite	33°15.906'	111°08.903'	—	Cretaceous	granite	—	—	90.8	11	Enkelmann et al., 2006
Q31	Apatite	33°46.315'	109°50.027'	—	Cretaceous	diorite	13.2	1.4	68.2	2.9	Enkelmann et al., 2006
Q32	Apatite	33°49.485'	109°50.631'	—	Cretaceous	gneiss	12.8	1.3	75.2	3.6	Enkelmann et al., 2006

Q33	Apatite	33°51.464'	109°50.228'	—	Cretaceous	gneiss	—	—	51.4	4.4	Enkelmann et al., 2006
Q36	Apatite	34°14.836'	106°56.149'	—	—	syenite	—	—	46	4.7	Enkelmann et al., 2006
Q37	Apatite	34°09.079'	106°45.767'	—	Cretaceous	diorite	12.9	1.3	84.6	6.7	Enkelmann et al., 2006
Q39	Apatite	34°02.006'	106°40.689'	—	Cretaceous	phyllite	—	—	69	6	Enkelmann et al., 2006
Q43	Apatite	33°56.876'	106°18.007'	—	Cretaceous	granodiorite	12.8	1.1	76.1	5.9	Enkelmann et al., 2006
Q44	Apatite	33°56.479'	106°17.849'	—	Cretaceous	metavolcanic	13.2	1	80.5	6.6	Enkelmann et al., 2006
Q55	Apatite	33°42.945'	106°47.103'	—	Cretaceous	granodiorite	11.7	1.3	66.5	3.4	Enkelmann et al., 2006
Q57	Apatite	33°41.587'	106°48.197'	—	Cretaceous	tonalite	—	—	73.2	4.6	Enkelmann et al., 2006
Q58	Apatite	33°40.840'	106°49.862'	—	Cretaceous	tonalite	—	—	69.3	9.2	Enkelmann et al., 2006
Q60	Apatite	33°38.064'	106°54.381'	—	Cretaceous	granodiorite	—	—	52.5	3.1	Enkelmann et al., 2006
Q62	Apatite	33°32.338'	106°58.743'	—	Cretaceous	granodiorite	—	—	54.9	5	Enkelmann et al., 2006
Q65	Apatite	33°23.738'	106°59.816'	—	Cretaceous	migmatite	11.9	1.6	57.9	5.1	Enkelmann et al., 2006
Q67	Apatite	33°15.184'	106°57.379'	—	Cretaceous	diorite	12.2	1.3	71.4	3.7	Enkelmann et al., 2006
Q69	Apatite	33°12.838'	106°44.709'	—	Cretaceous	granite	11.8	1.3	68.3	6.1	Enkelmann et al., 2006
Q76	Apatite	33°03.296'	106°18.851'	—	Cretaceous	granite	12.7	1.3	71.9	6.5	Enkelmann et al., 2006
Q77	Apatite	33°01.083'	106°15.693'	—	Cretaceous	metavolcanic	—	—	58.5	7	Enkelmann et al., 2006
Q96	Apatite	33°21.698'	106°21.673'	—	Cretaceous	granite	—	—	49	4.8	Enkelmann et al., 2006
Q98	Apatite	33°21.191'	106°21.787'	—	Cretaceous	gabbro	—	—	41	6.2	Enkelmann et al., 2006
Q104	Apatite	33°20.177'	105°50.887'	—	Cretaceous	dike	—	—	45.4	4.2	Enkelmann et al., 2006
Q105	Apatite	33°20.571'	105°48.372'	—	Cretaceous	dike	—	—	96.9	11	Enkelmann et al., 2006
Q106	Apatite	33°20.812'	105°45.634'	—	Cretaceous	dike	—	—	71.6	8.2	Enkelmann et al., 2006
Q113	Apatite	33°28.262'	105°21.860'	—	Cretaceous	conglomerate	12.1	1.1	44	2.2	Enkelmann et al., 2006
Q119	Apatite	33°57.529'	105°47.412'	—	—	granodiorite	—	—	55.9	4.6	Enkelmann et al., 2006
Q120	Apatite	34°03.158'	105°45.494'	—	—	granodiorite	—	—	79.7	6.9	Enkelmann et al., 2006
Q122	Apatite	34°13.118'	105°48.056'	—	Cretaceous	metabolcanic	13.1	1	95.2	8.8	Enkelmann et al., 2006
Q123	Apatite	34°32.555'	106°06.239'	—	Cretaceous	granodiorite	13.5	1	83	7.8	Enkelmann et al., 2006
Q124	Apatite	34°32.781'	106°11.538'	—	Cretaceous	gneiss	12.9	0.8	82.7	7.3	Enkelmann et al., 2006
Q125	Apatite	34°30.770'	106°21.671'	—	—	syenite	—	—	75.5	6.3	Enkelmann et al., 2006
Q127	Apatite	34°22.191'	106°42.751'	—	—	diorite	13	1.1	78	6.5	Enkelmann et al., 2006
D415	Apatite	33°17.164'	112°36.846'	—	—	granite	13.1	1.5	83.3	5.6	Enkelmann et al., 2006

D427	Apatite	33°48.579'	110°15.976'	—	Cretaceous	gneiss	13.4	1.3	73.6	6.1	Enkelmann et al., 2006
D436	Apatite	33°32.743'	110°38.927'	—	Cretaceous	gneiss	12.7	1.4	96.8	4.3	Enkelmann et al., 2006
HC119	Apatite	33°08.585'	111°45.352'	—	Cretaceous	sandstone	—	—	79	4.2	Enkelmann et al., 2006
HC128	Apatite	33°40.048'	110°21.232'	—	Cretaceous	granite	12.5	1.6	85.9	4.8	Enkelmann et al., 2006
HC130	Apatite	34°02.451'	109°32.221'	—	—	granite	13	2	49.5	2.4	Enkelmann et al., 2006
HC131	Apatite	34°04.461'	109°25.507'	—	—	granite	—	—	49.4	3.4	Enkelmann et al., 2006
DS428	Apatite	33°39.752'	110°20.153'	—	Cretaceous	mylonite	—	—	68.6	2.2	Enkelmann et al., 2006
DS430	Apatite	33°42.325'	110°20.549'	—	Cretaceous	granite	11.8	2	105	5	Enkelmann et al., 2006
Mc-8a	Apatite	32°35.369'	106°50.674'	1269	Proterozoic	diorite	—	—	99	—	Sun, 2011
Mc-17	Apatite	32°28.313'	106°26.733'	625	Proterozoic	vein	—	—	81	—	Sun, 2011
YWS-01	Apatite	33°12.413'	106°44.767'	605	—	sandstone	14.18	0.1	65.8	3.6	Wang, 2013
YWS-02	Apatite	33°13.182'	106°45.658'	706	Triassic	granite	14.06	0.09	55.3	2.6	Wang, 2013
YWS-03	Apatite	33°13.278'	106°45.895'	802	Triassic	granite	14.2	0.2	62.5	2.85	Wang, 2013
YWS-04	Apatite	33°13.512'	106°46.097'	896	Triassic	granite	14.4	0.12	59.3	2.8	Wang, 2013
YWS-05	Apatite	33°14.265'	106°48.652'	1021	Triassic	granite	14.38	0.1	59.9	2.7	Wang, 2013
YWS-06	Apatite	33°15.518'	106°49.095'	1122	Triassic	granite	14.28	0.1	58.9	2.6	Wang, 2013
YWS-07	Apatite	33°15.730'	106°49.215'	1212	Triassic	granite	14.6	0.09	62.2	2.9	Wang, 2013
YWS-08	Apatite	33°19.375'	106°51.140'	1361	Triassic	granite	14.41	0.09	59	2.7	Wang, 2013
YWS-09	Apatite	33°20.707'	106°50.685'	1480	Triassic	granite	14.27	0.11	58	3	Wang, 2013
YWS-10	Apatite	33°21.290'	106°51.057'	1616	Triassic	granite	14.57	0.11	62.7	3.1	Wang, 2013
YWS-11	Apatite	33°21.690'	106°51.105'	1745	Triassic	granite	14.67	0.09	61.9	3.4	Wang, 2013
YWS-12	Apatite	33°21.863'	106°51.348'	1869	Triassic	granite	14.8	0.09	65.5	3.3	Wang, 2013
YWS-13	Apatite	33°19.890'	106°48.143'	1965	Triassic	granite	14.21	0.1	62.2	3	Wang, 2013
YWS-14	Apatite	33°19.898'	106°47.952'	2080	Triassic	granite	14.59	0.09	62.5	3.1	Wang, 2013
YWS-15	Apatite	33°20.147'	106°48.060'	2205	Triassic	granite	14.42	0.1	60.2	3.1	Wang, 2013
YWS-16	Apatite	33°20.445'	106°48.248'	2323	Triassic	granite	14.6	0.1	59.8	3.1	Wang, 2013
YWS-17	Apatite	33°20.345'	106°47.945'	2401	Triassic	granite	14.07	0.09	60.8	3.3	Wang, 2013
D1058-1	Apatite	21°37'50.1"	107°41'41.1"	215	Upper Permian-Triassic	adamellite	—	—	63	6.5	Tan et al., 2010
D1060-1	Apatite	21°38'02.3"	107°40'23.4"	545	Upper Permian-Triassic	adamellite	—	—	61.5	6	Tan et al., 2010
D1061A	Apatite	21°38'04.2"	107°39'57.9"	781	Upper Permian-Triassic	adamellite	12.4	1.7	58.2	3.7	Tan et al., 2010

D1062-1	Apatite	21°38'28.9"	107°40'05.7"	970	Upper Permian-Triassic	adamellite	—	—	63.1	4.9	Tan et al., 2010
D5115-1	Apatite	22°30'11.1"	109°28'40.5"	630	Upper Permian-Triassic	adamellite	—	—	66.2	6.1	Tan et al., 2010
D5116-1	Apatite	22°30'09.6"	109°28'47.9"	544	Upper Permian-Triassic	adamellite	—	—	53.2	5.7	Tan et al., 2010
D5117-1	Apatite	22°30'08.6"	109°28'54.7"	438	Upper Permian-Triassic	adamellite	12.8	1.7	44.5	2.3	Tan et al., 2010
D5118-1	Apatite	22°30'29.4"	109°29'02.2"	366	Upper Permian-Triassic	adamellite	—	—	50.3	5.9	Tan et al., 2010
PD048	Apatite	28°45'19"	106°48'36"	368.4	Lower Permian	sandstone	10.7	2.1	60	5	Li SJ et al., 2011
LC019	Apatite	28°21'35"	106°24'19"	967.7	Lower Ordovician	sandstone	10.4	1.9	29	5	Li SJ et al., 2011
LC059	Apatite	28°26'28"	106°24'13"	—	—	sandstone	12.3	1.9	41	3	Li SJ et al., 2011
BS007	Apatite	27°32'35"	106°20'04"	993.6	Lower Cambrian	sandstone	11.4	1.6	43	4	Li SJ et al., 2011
BS022	Apatite	27°31'53"	106°20'49"	975.2	Lower Ordovician	sandstone	11.1	2.2	43	4	Li SJ et al., 2011
BS040	Apatite	27°30'50"	103°22'17"	967.4	Upper Triassic	sandstone	11.9	1.9	60	4	Li SJ et al., 2011
XW022	Apatite	28°17'41"	105°04'56"	329.9	Middle Silurian	sandstone	11.8	2.4	27	3	Li SJ et al., 2011
XW029	Apatite	28°17'56"	105°31'58"	422.8	Lower Triassic	sandstone	12.6	2.1	30	4	Li SJ et al., 2011
XW041	Apatite	28°19'31"	105°14'50"	316.3	Middle Jurassic	sandstone	11.6	2.3	28	2	Li SJ et al., 2011
SC012	Apatite	30°16'50"	109°16'50"	747.3	—	sandstone	12.2	1.9	93	7	Li SJ et al., 2011
2011NW01	Apatite	38°35'46"	111°58'28"	1519	Jurassic	sandstone	12.8	1.9	23	2	Li JX et al., 2015
2011NW02	Apatite	38°36'23"	111°57'14"	1551	Jurassic	sandstone	12.8	1.8	29	2	Li JX et al., 2015
2011NW03	Apatite	38°36'13"	111°56'43"	1590	Jurassic	sandstone	12.8	2.2	30	3	Li JX et al., 2015
2011NW04	Apatite	38°36'11"	111°56'30"	1594	Triassic	sandstone	12.4	1.9	31	3	Li JX et al., 2015
2011NW05	Apatite	38°36'22"	111°55'46"	1572	Permian	sandstone	12.9	1.9	18	1	Li JX et al., 2015
2011NW07	Apatite	38°36'08"	111°55'09"	1566	Permian	sandstone	13	2.1	28	2	Li JX et al., 2015
2011NW08	Apatite	38°55'19"	111°09'02"	943	Permian	sandstone	12.3	2	32	3	Li JX et al., 2015
2011NW09	Apatite	38°45'26"	111°07'34"	1160	Permian	sandstone	12.7	2.1	40	3	Li JX et al., 2015
2011NW11	Apatite	39°03'36"	111°01'51"	874	Permian	sandstone	11.6	2.1	61	5	Li JX et al., 2015
2011NW12	Apatite	39°03'35"	111°02'49"	967	Permian	sandstone	11.5	2.3	65	6	Li JX et al., 2015
2011NW13	Apatite	39°04'01"	111°01'00"	1038	Triassic	sandstone	12.2	2.1	48	4	Li JX et al., 2015
2011NW14	Apatite	39°04'43"	111°39'41"	1029	Jurassic	—	12.3	1.7	49	4	Li JX et al., 2015
2011NW16	Apatite	37°25'43"	110°53'42"	902	Permian	—	13.8	1.7	21	2	Li JX et al., 2015
2011NW17	Apatite	37°25'36"	111°53'05"	1188	Permian	—	13.9	1.8	20	2	Li JX et al., 2015
2011NW18	Apatite	37°26'44"	110°41'10"	787	Permian	—	12.3	2.2	17	2	Li JX et al., 2015

2011NW19	Apatite	37°26'57"	110°42'56"	791	Permian	—	13.7	1.8	24	2	Li JX et al., 2015
2011NW21	Apatite	37°46'43"	111°21'02"	1935	Proterozoic	instrusives	12.7	2	53	4	Li JX et al., 2015
2011NW22	Apatite	37°46'41"	111°20'48"	1837	Proterozoic	instrusives	12.2	1.9	67	6	Li JX et al., 2015
2011NW23	Apatite	37°46'36"	111°20'34"	1726	Proterozoic	instrusives	12.3	2	69	5	Li JX et al., 2015
2011NW24	Apatite	37°46'17"	111°20'20"	1636	Proterozoic	instrusives	—	—	15	2	Li JX et al., 2015
2011NW26	Apatite	37°31'09"	111°58'30"	927	Permian	—	13.4	1.9	41	4	Li JX et al., 2015
2011NW27	Apatite	37°30'56"	111°59'49"	856	Triassic	—	13	2	37	4	Li JX et al., 2015
2011NW28	Apatite	37°16'00"	111°23'32"	1544	Permian	—	13.5	2	15	1	Li JX et al., 2015
2011NW29	Apatite	36°15'53"	111°22'44"	1175	Permian	sandstone	13.5	1.5	15	2	Li JX et al., 2015
Tl09-18A	Apatite	28.35°	98.90°	2824	—	granite	13.99	0.15	15.3	1.1	Xiao et al., 2015
Tl09-19	Apatite	28.35°	98.91°	3021	—	granite	15.09	0.12	21	1.2	Xiao et al., 2015
TL09-20	Apatite	28.44°	98.92°	3517	—	granite	14.6	0.13	42	2.8	Xiao et al., 2015
YA06	Apatite	29.14°	99.58°	3339	Triassic	sandstone	13.1	0.43	41.1	4.5	Wilson et al., 2011
YA33	Apatite	30.14°	100.04°	4100	Cretaceous	microgranite	—	—	18	7	Wilson et al., 2011
YA34	Apatite	30.02°	100.32°	4190	Cretaceous	sandstone	11.99	0.21	74	4.8	Wilson et al., 2011
YA35	Apatite	30.14°	100.64°	4445	Cretaceous	sandstone	12.75	0.22	83.3	4.7	Wilson et al., 2011
YA36	Apatite	29.98°	100.9°	3729	Cretaceous	sandstone	11.97	0.37	29.3	3	Wilson et al., 2011
YA37	Apatite	30.01°	100.95°	3203	Cretaceous	sandstone	14.3	—	28.8	3.8	Wilson et al., 2011
YA38	Apatite	30.04°	100.99°	2711	Cretaceous	sandstone	12.24	0.67	16.6	1.4	Wilson et al., 2011
YA39	Apatite	30.05°	101.38°	4290	Cretaceous	sandstone	12.4	0.42	82.6	14.4	Wilson et al., 2011
068	Apatite	30.11°	101.62°	3748	Proterozoic	granite	12.21	—	13.4	3.4	Wilson et al., 2011
350	Apatite	30.04°	101.03°	3513	Triassic	sandstone	11.27	1.07	20.6	2.3	Wilson et al., 2011
353	Apatite	30.00°	100.87°	4163	Triassic	metasandstone	12.1	0.27	97.8	6.5	Wilson et al., 2011
365	Apatite	30.22°	99.92°	4255	—	granite	11.3	0.67	22.6	3.8	Wilson et al., 2011
393	Apatite	29.88°	100.32°	4057	Triassic	sandstone	12.5	0.28	91.5	5.1	Wilson et al., 2011
LM12	Apatite	30.29°	101.53°	3753	Upper Permian-Triassic	granite	12.21	0.23	85.7	7.5	Wilson et al., 2011
YA40	Apatite	30.05°	101.38°	4290	Triassic	sandstone	12.4	0.42	82.6	14.42	Wilson et al., 2011
YA41	Apatite	29.83°	102.22°	1300	Proterozoic	granite	—	—	6.9	4.7	Wilson et al., 2011
YA43	Apatite	29.8°	102.22°	2150	Proterozoic	granite	—	—	25.7	12.5	Wilson et al., 2011
YA44	Apatite	29.84°	102.25°	2251	Proterozoic	granite	12.3	0.07	40.2	19.7	Wilson et al., 2011

YA47	Apatite	30.04°	102.76°	750	Jurassic	sandstone	141	—	34.4	6.3	Wilson et al., 2011
HKT15	Apatite	31.02°	103.17°	2081	Triassic	sandstone	13.8	0.75	4.9	0.9	Wilson et al., 2011
KD10	Apatite	30.32°	102.16°	2265	Proterozoic	granite	10.5	1.59	9	2.45	Wilson et al., 2011
KD11	Apatite	30.38°	102.13°	1832	Proterozoic	granite	10.64	1.71	3.8	1.2	Wilson et al., 2011
005	Apatite	30.79°	102.73°	2667	Triassic	sandstone	8.8	1.55	37.7	4.8	Wilson et al., 2011
008	Apatite	30.85°	102.66°	3951	Triassic	sandstone	12.3	0.24	59.4	6.8	Wilson et al., 2011
009	Apatite	30.94°	102.95°	3443	Triassic	sandstone	14.4	1.34	42.1	7.6	Wilson et al., 2011
LM01	Apatite	31.08°	103.72°	799	Jurassic	sandstone	11.01	0.22	153.5	12.9	Wilson et al., 2011
LM03	Apatite	31.14°	103.49°	987	—	pegmatite	15.41	—	7.7	4.52	Wilson et al., 2011
LM04	Apatite	31.2°	103.49°	995	Proterozoic	granite	12.91	1.07	8.9	2.5	Wilson et al., 2011
LM07	Apatite	31.07°	103.45°	1014	Proterozoic	granite	13.23	0.66	4.9	0.6	Wilson et al., 2011
LM09	Apatite	30.87°	101.84°	2939	—	acid dike	10	1.43	3.8	0.6	Wilson et al., 2011
LM14	Apatite	29.99°	101.92°	3280	Palaeocene	granite	12.96	2.27	1.2	0.2	Wilson et al., 2011
LM16	Apatite	30.08°	102.09°	2032	Proterozoic	granite	13.29	0.44	5.5	0.7	Wilson et al., 2011
LM17	Apatite	30.07°	102.15°	1475	Proterozoic	granite	10.71	0.93	4.6	0.7	Wilson et al., 2011
YA07	Apatite	28.26°	99.21°	2764	Triassic	sandstone	10.7	1.14	22.4	3.8	Wilson et al., 2011
YA08	Apatite	28.34°	99.06°	4321	—	granite	14.2	0.67	130.7	32.7	Wilson et al., 2011
YA09	Apatite	28.36°	99.06°	4537	Eocene	sandstone	12.33	0.7	13.2	1.2	Wilson et al., 2011
YA10	Apatite	28.37°	99.02°	4236	Triassic	sandstone	11.6	1.79	39.6	5.4	Wilson et al., 2011
YA11	Apatite	28.43°	98.96°	3608	Palaeozoic	metaquartzite	13.2	0.35	17.2	2.5	Wilson et al., 2011
YA14	Apatite	29.28°	98.67°	4142	Triassic	sandstone	10.2	0.83	59.4	10	Wilson et al., 2011
YA15	Apatite	29.74°	98.67°	3718	Triassic	sandstone	13.1	0.18	70.9	5.9	Wilson et al., 2011
YA16	Apatite	29.74°	98.67°	3682	Devonian	quartzite	12.1	0.44	39	4.1	Wilson et al., 2011
YA17	Apatite	29.72°	98.82°	3361	Eocene	pebble conglomerate	—	—	23	7	Wilson et al., 2011
YA19	Apatite	29.76°	98.98°	2516	Triassic	metasandstone	10	0.45	23	8.5	Wilson et al., 2011
YA21	Apatite	29.44°	98.64°	3682	Jurassic	sandstone	13.5	0.89	43.7	6.8	Wilson et al., 2011
YA22T	Apatite	29.22°	98.66°	3790	Triassic	sandstone	12.2	0.61	17	1.7	Wilson et al., 2011
YA23	Apatite	29.2°	98.63°	3780	Triassic	sandstone	12.4	0.7	10.9	1.4	Wilson et al., 2011
YA24	Apatite	29.06°	98.61°	2707	Triassic	sandstone	9.9	1.68	4.2	0.7	Wilson et al., 2011
YA25	Apatite	28.99°	98.61°	2430	Triassic	sandstone	10.1	3.34	3	1.9	Wilson et al., 2011

YA26	Apatite	28.97°	98.62°	2707	Permian	acid dike	6.51	1.31	42.5	8.1	Wilson et al., 2011
YA28	Apatite	28.49°	98.82°	2726	Devonian	sandstone	11.4	3.99	11.2	3.8	Wilson et al., 2011
YA30	Apatite	29.3°	99.15°	2778	—	microgranite	11.93	2.3	11.9	2.8	Wilson et al., 2011
YA31	Apatite	29.33°	99.07°	2898	—	acid dike	12.66	0.58	16.9	2.9	Wilson et al., 2011
406	Apatite	31.62°	99.72°	3550	Jurassic	granodiorite	12.2	0.71	7.1	1	Wilson et al., 2011
413	Apatite	31.96°	98.65°	3300	Triassic	sandstone	12	0.42	47	4.2	Wilson et al., 2011
421	Apatite	31.56°	98.3°	3250	Cretaceous	granite	12.8	1.08	16.8	7.9	Wilson et al., 2011
426	Apatite	31.94°	98.84°	4900	Cretaceous	granite	12.5	—	6	1.4	Wilson et al., 2011
KD29	Apatite	28.44°	102.04°	2096	Proterozoic	quartzite	12.7	1.78	34.8	12.9	Wilson et al., 2011
KD31	Apatite	28.3°	102.22°	1671	Proterozoic	granite	13.44	—	5.6	0.9	Wilson et al., 2011
KD49	Apatite	26.8°	101.53°	1968	Proterozoic	metasandstone	13.42	0.3	24.8	3	Wilson et al., 2011
KD58	Apatite	27.06°	101.35°	1499	Permian	sandstone	12.14	0.22	100	7.3	Wilson et al., 2011
KD59	Apatite	26.98°	101.41°	1255	Proterozoic	quartzite	14.6	—	35.3	6.8	Wilson et al., 2011
KD60	Apatite	26.77°	101.55°	2198	Proterozoic	metasandstone	13.6	0.18	27.6	1.5	Wilson et al., 2011
KD61	Apatite	26.47°	101.76°	1162	Proterozoic	granodiorite	13.6	0.20	25.5	2	Wilson et al., 2011
KD63	Apatite	26.3°	101.78°	1643	Proterozoic	granodiorite	11.4	—	54.8	19	Wilson et al., 2011
QL-12	Apatite	34°30.20'	109°57.46'	1600	Cretaceous	granite	13.1	0.17	30	4.1	Hu et al., 2006b
QL-13	Apatite	34°26.53'	109°56.86'	1700	Upper Archaeozoic	granite	13.1	0.16	34	2	Hu et al., 2006b
QL-14	Apatite	34°20.33'	110°02.69'	1200	Jurassic	—	12.8	0.19	65	5	Hu et al., 2006b
QL-15	Apatite	34°01.24'	110°14.24'	1400	Cretaceous	granite	12.9	0.15	62	4.5	Hu et al., 2006b
QL-16	Apatite	33°46.69'	110°16.52'	900	Palaeozoic	granite	13	0.14	69	2.8	Hu et al., 2006b
QL-1	Apatite	33°39.58'	111°10.47'	863	Archaeozoic	granite	12.6	0.21	57	3	Hu et al., 2006b
QL-2	Apatite	33°37.42'	111°11.22'	804	Archaeozoic	granite	12.2	0.18	73	2.5	Hu et al., 2006b
QL-4	Apatite	33°35.88'	111°10.40'	763	Archaeozoic	granite	—	—	63	10	Hu et al., 2006b
QL-5	Apatite	33°33.88'	111°10.47'	651	Archaeozoic	granite	12.2	0.17	62	3	Hu et al., 2006b
QL-11	Apatite	33°38.45'	111°10.48'	875	Middle Proterozoic	—	12.6	0.17	73	4	Hu et al., 2006b
QL-3	Apatite	33°32.68'	111°10.63'	746	Middle-Upper Devonian	granite	11.6	0.23	88	5	Hu et al., 2006b
QL-17	Apatite	33°43.39'	110°16.36'	950	Archaeozoic	granite	12.2	0.26	80	4	Hu et al., 2006b
QL-19	Apatite	33°26.04'	110°53.72'	600	Middle-Upper Devonian	granite	12.4	0.15	116	7	Hu et al., 2006b
QL-6	Apatite	33°23.32'	111°05.22'	553	Middle Proterozoic	granite	12.9	0.10	98	8	Hu et al., 2006b

QL-7	Apatite	33°20.37'	111°02.68'	752	Middle Proterozoic	granite	12.7	0.23	99	9	Hu et al., 2006b
QL-8	Apatite	33°18.80'	111°03.10'	603	Middle Proterozoic	granite	11.9	0.23	129	5	Hu et al., 2006b
QL-21	Apatite	32°54.34'	110°56.42'	900	Middle Proterozoic	granite	12.9	0.23	80	5.1	Hu et al., 2006b
QL-22	Apatite	32°44.07'	110°46.71'	800	Middle Proterozoic	granite	—	—	102	16	Hu et al., 2006b
QL-23	Apatite	32°31.26'	110°40.11'	1100	Middle Proterozoic	granite	12.4	0.21	82	77	Hu et al., 2006b
QL-29	Apatite	31°11.29'	110°57.58'	500	Upper Archaeozoic-Lower Proterozoic	granite	12.6	0.22	87	3.3	Hu et al., 2006b
QL-30	Apatite	31°07.79'	111°10.98'	500	Upper Archaeozoic-Lower Proterozoic	granite	12.6	0.14	95	5	Hu et al., 2006b
QL-31	Apatite	31°04.40'	111°14.19'	400	Upper Archaeozoic-Lower Proterozoic	granite	11.8	0.24	93	7	Hu et al., 2006b
QL-32	Apatite	30°52.83'	110°59.93'	320	Upper Archaeozoic-Lower Proterozoic	granite	12.4	0.12	88	3	Hu et al., 2006b
QL-33	Apatite	30°52.57'	111°00.87'	200	Upper Archaeozoic-Lower Proterozoic	granite	12.4	0.14	96	4	Hu et al., 2006b
QL-34	Apatite	30°51.78'	111°04.08'	160	Upper Archaeozoic-Lower Proterozoic	granite	12.6	0.16	92	5.6	Hu et al., 2006b
QL-35	Apatite	30°57.53'	110°59.38'	1100	Upper Archaeozoic-Lower Proterozoic	granite	12.4	0.12	102	5	Hu et al., 2006b
04-Y-33	Apatite	39.38°	97.63°	3930	Pre-Cambrian	sandstone	13.06	0.15	33.1	3.7	Zheng et al., 2017
04-Y-34-1	Apatite	39.50°	97.69°	3288	Pre-Cambrian	sandstone	13.31	0.24	17.6	4.5	Zheng et al., 2017
04-Y-37	Apatite	39.70°	97.69°	2564	Jurassic	sandstone	13.24	0.12	98.2	12	Zheng et al., 2017
04-Y-39	Apatite	39.69°	97.68°	2590	Devonian	sandstone	13.73	0.15	71.1	9.6	Zheng et al., 2017
04-Y-40	Apatite	39.70°	97.69°	2491	Upper Permian	sandstone	13.15	0.16	75.2	8.6	Zheng et al., 2017
04-Y-41	Apatite	39.70°	97.691°	2478	Upper Permian	sandstone	13.13	0.11	51.3	6.1	Zheng et al., 2017
04-Y-44	Apatite	39.71°	97.70°	2451	Upper Permian	sandstone	13.49	0.13	58	7.1	Zheng et al., 2017
KLY-11	Apatite	37.063517°	77.858915°	2640	Upper Devonian	metapelite	12.7	1.60	6.8	0.6	Wang, 2015
KLY-12	Apatite	37.065117°	77.858458°	2631	Upper Devonian	metapelite	—	—	8.6	0.7	Wang, 2015
KLY-13	Apatite	37.075864°	77.856958°	2798	Upper Devonian	metasandstone	—	—	11.3	1	Wang, 2015
KLY-16	Apatite	37.106784°	77.839807°	2576	Middle Proterozoic	metamorphic schist	13.1	1.90	11.5	0.8	Wang, 2015
KLY-18	Apatite	37.117773°	77.839909°	2530	Middle Proterozoic	metamorphic schist	11.8	2.20	16.4	1.2	Wang, 2015
KLY-19	Apatite	37.120637°	77.838863°	2514	Middle Proterozoic	metamorphic schist	12.8	2.50	12.6	0.8	Wang, 2015
KLY-20	Apatite	37.135917°	77.833131°	2504	Middle Proterozoic	metamorphic schist	13.5	1.20	14.3	1.1	Wang, 2015
KLY-21	Apatite	37.152007°	77.808368°	2430	Middle Proterozoic	metamorphic schist	—	—	12.4	1	Wang, 2015
KLY-22	Apatite	37.168512°	77.76556°	2400	Middle Proterozoic	metamorphic schist	13.3	2.50	15	1.2	Wang, 2015
BY-1	Apatite	36.156679°	80.076316°	3864	Middle Proterozoic	quartz schist	12.6	1.60	9.3	1.4	Wang, 2015
BY-3	Apatite	36.157815°	80.074233°	4187	Middle Proterozoic	quartz schist	12.4	1.60	4.8	1.6	Wang, 2015

2254-1	Apatite	31°56'20"	99°55'49"	4583	Triassic	sandstone	14.03	0.12	33.4	2.3	Wang et al, 2015(b)
DD001-1	Apatite	31°52'25"	100°16'12"	3589	Triassic	sandstone	13.83	0.11	34.7	2.4	Wang et al, 2015(b)
DD002-1	Apatite	31°50'41"	100°16'08"	4106	Triassic	sandstone	14.06	0.12	44	3.4	Wang et al, 2015(b)
DD009-1	Apatite	31°53'35"	100°06'24"	3812	Triassic	sandstone	14.01	0.13	37.9	3.1	Wang et al, 2015(b)
DD007-1	Apatite	31°53'21"	100°12'37"	4653	Triassic	sandstone	13.79	0.12	34.2	2.6	Wang et al, 2015(b)
DD0010-1	Apatite	31°54'18"	100°07'37"	3653	Triassic	sandstone	14.16	0.12	30.1	2.4	Wang et al, 2015(b)
DD005-1	Apatite	31°52'16"	100°10'37"	3922	Triassic	sandstone	13.82	0.13	39.2	2.9	Wang et al, 2015(b)
SP17-(01)B	Apatite	34°31'	92°54'	5050	Eocene	sandstone	12.8	1.16	47.2	5.9	Jin, 2007
P03-11B2	Apatite	34°38'	91°07'	5080	Eocene	sandstone	12.4	0.55	46.7	2.7	Jin, 2007
P14-51	Apatite	34°58'40"	90°01'20"	4930	Eocene	sandstone	12	0.4	40	5.5	Jin, 2007
SQ16	Apatite	34°34'25"	92°54'42"	5350	Eocene	sandstone	13.9	0.54	39.4	4.2	Jin, 2007
P14-52	Apatite	34°58'41"	90°01'20"	4930	Eocene	sandstone	11.7	0.64	39.1	4.6	Jin, 2007
SP19-1	Apatite	34°33'36"	92°54'18"	8100	Eocene	sandstone	12.9	0.61	37.4	4.5	Jin, 2007
SQ22	Apatite	34°34'25"	92°54'52"	5350	Eocene	sandstone	14.1	0.47	36.2	4.3	Jin, 2007
KP01-(21)C	Apatite	35°25'46"	90°47'05"	5200	Eocene	sandstone	13.8	0.31	28.6	2.8	Jin, 2007
P0126HX	Apatite	33°36.25'	92°04'	—	Jurassic	sandstone	13.1	0.44	38.2	4	Jin, 2007
P0158HX	Apatite	33°36'	92°04'	—	Jurassic	sandstone	13.4	0.44	47.3	4.3	Jin, 2007
P0171HX	Apatite	33°35'	92°04'	—	Jurassic	sandstone	14	0.63	39.4	5.5	Jin, 2007
P0196B1	Apatite	33°40'	92°04'	—	Jurassic	sandstone	12.7	0.81	82	18.7	Jin, 2007
AR01	Apatite	29.26°	88.61°	3961	Cretaceous	sandstone	—	—	27.1	4.9	Ge, 2016
AR17	Apatite	29.26°	88.611°	3992	Cretaceous	sandstone	—	—	27.1	9.3	Ge, 2016
DJG11-01	Apatite	29.17°	88.67°	3984	Cretaceous	sandstone	—	—	26.1	6.8	Ge, 2016
DJG11-02	Apatite	29.31°	88.85°	3912	Cretaceous	sandstone	—	—	22.2	4.9	Ge, 2016
DJG11-04	Apatite	29.22°	88.89°	3862	Cretaceous	sandstone	—	—	18.7	2.9	Ge, 2016
L04-03U1	Apatite	29.44°	89.63°	3950	Palaeocene	granite	—	—	14	1.3	Ge, 2016
L05-08U1	Apatite	29.61°	89.98°	4294	Eocene	granite	—	—	17.7	1.6	Ge, 2016
L05-11U1	Apatite	29.56°	90.01°	4229	Upper Cretaceous	granite	—	—	14.9	1.3	Ge, 2016
L05-12U1	Apatite	29.53°	90.02°	4139	Upper Cretaceous	granite	—	—	14.2	1.4	Ge, 2016
L06-01U1	Apatite	29.27°	90.25°	4170	Lower Cretaceous	granite	—	—	23.3	3.9	Ge, 2016
L06-02U1	Apatite	29.29°	90.25°	4017	Lower Cretaceous	granite	—	—	8.5	0.9	Ge, 2016

L06-06U1	Apatite	29.36°	90.18°	3751	Lower-Middle Proterozoic	—	—	—	10.4	1.7	Ge, 2016
L06-07U1	Apatite	29.39°	90.17°	3776	Lower Cretaceous	granite	14.55	0.25	7.9	0.6	Ge, 2016
L07-14U1	Apatite	29.52°	90.92°	3646	Lower Cretaceous	granite	14.52	0.13	15.9	0.9	Ge, 2016
L10-04U1	Apatite	30.00°	91.93°	3946	Upper Triassic	granite	15.29	0.18	22.5	2.4	Ge, 2016
L13-04U1	Apatite	29.50°	91.80°	4219	Lower Cretaceous	granite	—	—	15	1.9	Ge, 2016
L13-08U1	Apatite	29.32°	91.84°	3699	Lower Cretaceous	granite	—	—	25.3	1.7	Ge, 2016
N12-44	Apatite	29.91°	89.10°	4251	Carboniferous	sandstone	—	—	17.3	5.7	Ge, 2016
N12-46	Apatite	29.97°	89.10°	4300	Carboniferous	sandstone	—	—	23	6.3	Ge, 2016
N12-47	Apatite	29.99°	89.08°	4299	Carboniferous	sandstone	—	—	18.2	3.3	Ge, 2016
N12-48	Apatite	30.01°	89.08°	4317	Carboniferous	sandstone	—	—	13.2	2.1	Ge, 2016
N12-50	Apatite	30.04°	89.084°	4391	Jurassic	granodiorite	—	—	15.6	1.8	Ge, 2016
N12-52	Apatite	30.09°	89.11°	4431	Jurassic	two-micagranite	—	—	16.6	1.2	Ge, 2016
N12-53	Apatite	30.09°	89.13°	4478	Jurassic	two-micagranite	12.66	0.18	22.1	0.9	Ge, 2016
N12-55	Apatite	30.11°	89.15°	4530	Triassic	two-micagranite	12.5	0.19	24.1	1	Ge, 2016
N12-56	Apatite	30.12°	89.16°	4681	Upper Triassic-Lower Jurassic	grant porphyry granodiorite	13.5	0.24	12.8	0.7	Ge, 2016
N12-59	Apatite	30.20°	89.27°	4805	Jurassic	grant porphyry granodiorite	13.44	0.2	36.9	3.1	Ge, 2016
QW12-40U1	Apatite	29.27°	87.42°	4205	Oligocene-Miocene	sandstone	—	—	18.3	3.3	Ge, 2016
QW12-6U1	Apatite	29.39°	87.42°	4417	Oligocene-Miocene	sandstone	—	—	15.8	1.8	Ge, 2016
QW13-U1	Apatite	29.39°	87.421°	4366	Oligocene-Miocene	sandstone	—	—	14.4	1.7	Ge, 2016
QW13-U2	Apatite	29.39°	87.4205°	4361	Oligocene-Miocene	sandstone	—	—	16.9	2.4	Ge, 2016
QW13-U3	Apatite	29.38°	87.42°	4258	Oligocene-Miocene	sandstone	—	—	13.1	2.2	Ge, 2016
X12-14	Apatite	29.35°	88.81°	3865	Cretaceous	epidotized granodiorite	—	—	16.6	1.4	Ge, 2016
X12-20	Apatite	29.37°	88.25°	3921	Cretaceous	tonalite	14.52	0.19	23.7	2.1	Ge, 2016
X12-21	Apatite	29.43°	88.26°	4024	Eocene	granodiorite	—	—	9.4	0.6	Ge, 2016
X12-23	Apatite	29.46°	88.23°	3982	Eocene	granite	—	—	25.4	3.9	Ge, 2016
X12-25	Apatite	29.49°	88.22°	4154	Eocene	monzogranite	—	—	25.4	6	Ge, 2016
X12-28	Apatite	29.54°	88.21°	4335	Eocene	monzogranite	13.44	0.66	23	2.4	Ge, 2016
X12-33	Apatite	29.60°	88.23°	4540	Eocene	monzogranite			25.4	3.8	Ge, 2016
X12-37	Apatite	29.65°	88.21°	4890	Eocene	monzogranite	12.69	0.5	28.6	2.8	Ge, 2016
X12-38	Apatite	29.70°	88.20°	5415	Eocene	monzogranite	—	—	9.4	1.3	Ge, 2016

SQM-05	Apatite	29.008°	103.8943°	360	Lower Cretaceous	sandstone	11.5	2.5	86	6	Deng et al., 2016
SQM-07	Apatite	28.9032°	103.8825°	476	Middle Jurassic	sandstone	12.2	1.8	42	3	Deng et al., 2016
SQM-08	Apatite	28.8934°	103.8778°	494	Middle Jurassic	sandstone	12	2.1	29	2	Deng et al., 2016
SQM-10	Apatite	28.8973°	103.8962°	583	Upper Triassic-Lower Jurassic	sandstone	12.2	1.9	25	2	Deng et al., 2016
SYX-01	Apatite	28.2646°	102.4768°	1909	Middle Jurassic	sandstone	11.9	1.7	14	2	Deng et al., 2016
SYX-02	Apatite	28.26239°	102.48689°	1914	Upper Triassic-Lower Jurassic	sandstone	11.9	2.2	17	2	Deng et al., 2016
SYX-03	Apatite	28.2648°	102.5032°	1952	Middle Jurassic	sandstone	11.3	2.1	22	2	Deng et al., 2016
SYX-06	Apatite	28.3164°	102.535°	2032	Upper Jurassic	sandstone	11.6	2.2	21	2	Deng et al., 2016
GAOJP213	Apatite	38.898449.9°	102°07'39.7"	1664	Middle Proterozoic	migmatitic granite	12.7	2.7	80	0.00	Tian et al., 2016
1	Apatite	30.04°	101.54167°	3480	—	sandstone	12.61	0	47.5	21.1	Xu et al., 2000
2	Apatite	30.0783333°	101.8044°	4290	—	granite	15.06	0.44	2.7	0.4	Xu et al., 2000
3	Apatite	29.9933333°	101.89667°	3200	—	granite	14.02	0.49	1.9	0.2	Xu et al., 2000
4	Apatite	29.9938889°	101.94°	3112	—	granite	13.69	0.49	1.6	0.4	Xu et al., 2000
5	Apatite	29.995°	101.94167°	3062	—	granite	14.59	0.51	2.3	0.8	Xu et al., 2000
6	Apatite	29.9958333°	101.94333°	3004	—	granite	13.95	0.07	2.6	1.5	Xu et al., 2000
7	Apatite	29.9966667°	101.945°	2983	—	granite	14.09	0.53	2.9	0.5	Xu et al., 2000
8	Apatite	30°	101.94667°	2983	—	granite	14.91	0.25	1.8	0.3	Xu et al., 2000
9	Apatite	30.005°	101.94833°	2870	—	granite	14.19	1.75	1.9	0.3	Xu et al., 2000
10	Apatite	30.0016667°	101.94833°	2868	—	granite	13.7	0.45	2.6	0.5	Xu et al., 2000
11	Apatite	30.01°	101.95°	2806	—	granite	14.72	1.39	2	0.5	Xu et al., 2000
12	Apatite	30.0133333°	101.95°	2795	—	granite	14.56	0.24	2.1	0.5	Xu et al., 2000
13	Apatite	30.0583333°	101.97833°	2503	Proterozoic	—	10.9	0.85	14.3	2.6	Xu et al., 2000
14	Apatite	30.0583333°	101.98167	2485	Proterozoic	—	13.3	0	9.8	4.1	Xu et al., 2000
15	Apatite	30.06°	102°	2450	Proterozoic	—	12.55	0.67	6.8	1.1	Xu et al., 2000
16	Apatite	30.06°	102.025°	2330	Proterozoic	—	13.03	0.62	7.6	1.1	Xu et al., 2000
17	Apatite	30.06°	102.03333°	2260	Proterozoic	—	14.5	0.51	4.5	1.1	Xu et al., 2000
18	Apatite	30.0616667°	102.03333°	2257	Proterozoic	—	11.94	1.58	5.8	1.2	Xu et al., 2000
19	Apatite	30.06167°	102.05°	2182	Proterozoic	—	10.9	1.98	6.5	1.3	Xu et al., 2000
20	Apatite	30.06167°	102.075°	2030	Proterozoic	—	12.52	2.75	5.4	1	Xu et al., 2000
21	Apatite	30.06333°	102.075°	2020	Proterozoic	—	—	—	7.7	1	Xu et al., 2000

22	Apatite	29.7°	102.01333°	2345	Proterozoic	—	—	—	3.4	0.6	Xu et al., 2000
23	Apatite	29.7°	102.015°	2340	—	granite	15.16	1.66	1.8	0.6	Xu et al., 2000
24	Apatite	29.70833°	102.01583°	2320	—	granite	13.6	0	1.2	0.3	Xu et al., 2000
25	Apatite	29.71667°	102.01667°	2288	—	granite	13.95	1.16	2.1	0.3	Xu et al., 2000
26	Apatite	29.725°	102.025°	2280	—	granite	15.11	0.4	1.7	0.7	Xu et al., 2000
27	Apatite	29.73333°	102.03333°	2280	—	granite	14.86	0	2	0.3	Xu et al., 2000
28	Apatite	29.7°	102.03833°	2110	—	granite	11.39	0	1.5	0.4	Xu et al., 2000
29	Apatite	29.7°	102.04°	2110	Permian-Triassic	—	—	—	2.5	1.5	Xu et al., 2000
30	Apatite	29.7°	102.04167°	2110	—	granite	—	—	1.6	0.3	Xu et al., 2000
31	Apatite	29.695°	102.03528°	2120	Permian-Triassic	—	14.62	0.88	3.5	0.9	Xu et al., 2000
32	Apatite	29.695°	102.04667°	2120	Permian-Triassic	—	12.75	0.95	6.4	1.1	Xu et al., 2000
33	Apatite	29.69167°	102.05667°	2120	Permian-Triassic	—	—	—	6	1.4	Xu et al., 2000
34	Apatite	29.63333°	102.14167°	1380	Proterozoic	—	—	—	12.6	3.5	Xu et al., 2000
35	Apatite	29.625°	102.14333°	1380	Proterozoic	—	12.72	1.01	7.8	0.9	Xu et al., 2000
36	Apatite	29.62°	102.14333°	1365	Proterozoic	—	10.6	1.71	13.6	1.4	Xu et al., 2000
37	Apatite	29.61667°	102.14333°	1382	Proterozoic	—	13.82	1.9	21.2	5.3	Xu et al., 2000
38	Apatite	29.53333°	102.14333°	1210	Proterozoic	—	—	—	5.6	2.8	Xu et al., 2000
39	Apatite	29.53333°	102.145°	1154	Proterozoic	—	13.07	1.12	21.9	3.7	Xu et al., 2000
40	Apatite	29.53333°	102.15°	1125	Proterozoic	—	—	—	15.6	5.7	Xu et al., 2000
41	Apatite	29.53333°	102.15833°	1084	Proterozoic	—	11.91	2.36	5.6	1.2	Xu et al., 2000
42	Apatite	29.53333°	102.16167°	1070	Proterozoic	—	—	—	12.1	1.9	Xu et al., 2000
43	Apatite	29.53333°	102.16667°	1082	Proterozoic	—	12.11	0.79	10.4	1.3	Xu et al., 2000
44	Apatite	29.5°	102.18°	1007	Proterozoic	—	—	—	23.1	5.8	Xu et al., 2000
45	Apatite	29.49167°	102.18°	1010	Proterozoic	—	—	—	22.9	9.1	Xu et al., 2000
47	Apatite	29.48333°	102.18°	1002	Proterozoic	—	—	—	9.1	5.4	Xu et al., 2000
48	Apatite	30.25667°	101.505°	3830	—	granite	13.62	0.23	84.4	3.9	Xu et al., 2000
49	Apatite	30.25667°	101.505°	3825	—	granite	12.97	0.27	77.6	3.4	Xu et al., 2000
50	Apatite	30.25667°	101.505°	3805	—	granite	12.94	0.51	78.9	6.9	Xu et al., 2000
51	Apatite	30.255°	101.50667°	3785	—	granite	12.58	0.3	73.1	3.8	Xu et al., 2000
52	Apatite	30.25°	101.50667°	3730	—	granite	13.52	0.12	71.4	3.7	Xu et al., 2000

53	Apatite	30.24583°	101.50833°	3585	—	granite	12.05	0.18	80.2	3.7	Xu et al., 2000
54	Apatite	30.245°	101.50833°	3590	—	granite	13.6	0.19	71.6	4.4	Xu et al., 2000
55	Apatite	30.28333°	101.53333°	3760	—	granite	12.45	0.25	69.3	5.6	Xu et al., 2000
56	Apatite	30.25333°	101.51667°	3560	—	granite	11.57	0.25	83.8	4	Xu et al., 2000
57	Apatite	30.54833°	101.61667°	3480	—	mylonite	—	—	3.1	0.6	Xu et al., 2000
58	Apatite	30.56667°	101.64333°	3280	—	granite	—	—	2.4	2.4	Xu et al., 2000
59	Apatite	30.57833°	101.65°	3000	—	granite	—	—	2.9	2.1	Xu et al., 2000
60	Apatite	31.54667°	100.37667°	3430	—	sandstone	10.27	1.29	10.6	1.7	Xu et al., 2000
61	Apatite	31.55167°	100.38167°	3420	—	sandstone	—	—	15.2	3.6	Xu et al., 2000
62	Apatite	31.56167°	100.38333°	3400	—	sandstone	12.86	1	11.2	3.8	Xu et al., 2000
63	Apatite	31.56417°	100.39°	3360	—	sandstone	—	—	18.4	5.8	Xu et al., 2000
64	Apatite	31.565°	100.39333°	3360	—	sandstone	12.65	0.31	18.8	2.7	Xu et al., 2000
65	Apatite	31.56667°	100.39667°	3350	—	sandstone	14.19	0.73	13.8	2.8	Xu et al., 2000
66	Apatite	31.43333°	100.59333°	3240	—	sandstone	14.9	0	14.8	3.3	Xu et al., 2000
67	Apatite	31.41667°	100.61667°	3235	—	sandstone	—	—	12.7	2.2	Xu et al., 2000
68	Apatite	31.44167°	100.68°	3200	—	sandstone	14.79	0	14.4	3.2	Xu et al., 2000
69	Apatite	31.45°	100.68333°	3200	—	sandstone	—	—	7.2	2.4	Xu et al., 2000
70	Apatite	31.48333°	100.69667°	3210	—	sandstone	12.94	1.66	13.2	3	Xu et al., 2000
71	Apatite	31.345°	100.655°	3660	—	granite	12.64	0.34	17.7	1.7	Xu et al., 2000
72	Apatite	31.34667°	100.65833°	3645	—	granite	12.74	0.48	11.6	1.4	Xu et al., 2000
73	Apatite	31.34833°	100.66°	3630	—	granite	12.29	0.25	20.6	1.5	Xu et al., 2000
74	Apatite	31.36667°	100.66667°	3520	—	granite	11.7	0.7	19.8	2.2	Xu et al., 2000
75	Apatite	31.37167°	100.66333°	3500	—	granite	12.49	0.24	18.8	1.7	Xu et al., 2000
76	Apatite	31.375°	100.665°	3400	—	granite	13.33	0.19	16.8	1.5	Xu et al., 2000
77	Apatite	31.37667°	100.66667°	3440	—	granite	13.19	0.23	22.1	1.4	Xu et al., 2000
78	Apatite	31.56167°	100.41667°	3420	—	mylonite	13.43	0.24	18.3	4	Xu et al., 2000
79	Apatite	30.76667°	101.03°	2830	—	sandstone	12.54	0	25.3	6.9	Xu et al., 2000
81	Apatite	30.95°	101.125°	2950	—	sandstone	—	—	20.9	15.4	Xu et al., 2000
82	Apatite	30.95833°	101.12833°	2930	—	sandstone	10.05	0.82	24.8	5.3	Xu et al., 2000
83	Apatite	30.83333°	101.26333°	3525	—	sandstone	—	—	21	12.7	Xu et al., 2000

87	Apatite	30.96667°	101.13°	2997	—	mylonite	16.15	0	30	18.3	Xu et al., 2000
88	Apatite	30.99667°	101.11°	2980	—	sandstone	—	—	7.4	3.1	Xu et al., 2000
89	Apatite	30.99333°	101.11333°	2990	—	sandstone	14.11	0.43	5.4	0.6	Xu et al., 2000
90	Apatite	30.995°	101.11667°	3000	—	sandstone	15.06	0	5.9	3	Xu et al., 2000
91	Apatite	31°	101.11667°	3100	—	sandstone	14.86	0.3	11.8	3.5	Xu et al., 2000
92	Apatite	31.01°	101.16°	3640	—	sandstone	13.24	0.77	15.4	2.6	Xu et al., 2000
93	Apatite	31.02333°	101.235°	4540	—	sandstone	13.04	0.45	18.2	4.3	Xu et al., 2000
94	Apatite	31.02667°	101.23833°	4560	—	sandstone	—	—	18.1	5.7	Xu et al., 2000
95	Apatite	31.00833°	101.00833°	3920	—	granite	13.44	0.27	24.7	4.1	Xu et al., 2000
96	Apatite	31.00333°	101.00583°	4040	—	granite	—	—	17.3	5.4	Xu et al., 2000
97	Apatite	30.825°	101.25°	3730	—	granite	14.08	0.11	71	4.2	Xu et al., 2000
98	Apatite	30.82583°	101.25083°	3730	—	granite	13.68	0.1	69.6	4.3	Xu et al., 2000
99	Apatite	30.82833°	101.25417°	3720	—	granite	14.63	0.18	84	7	Xu et al., 2000
100	Apatite	30.83111°	101.25722°	3665	—	granite	14.16	0.18	69.3	7	Xu et al., 2000
101	Apatite	30.83333°	101.2575°	3660	—	granite	14.17	0.25	85.4	6.1	Xu et al., 2000
102	Apatite	30.075°	101.94167°	2915	—	sandstone	—	—	3.2	1.4	Xu et al., 2000
104	Apatite	30.07167°	101.93667°	2982	—	sandstone	—	—	4	0.7	Xu et al., 2000
105	Apatite	29.92833°	102.225°	1368	Proterozoic	—	—	—	24.3	4	Xu et al., 2000
107	Apatite	29.91667°	102.225°	1346	—	mylonite	—	—	12	8.7	Xu et al., 2000
108	Apatite	29.91333°	102.225°	1351	—	mylonite	—	—	10	5.9	Xu et al., 2000
109	Apatite	29.92833°	102.22167°	1392	Proterozoic	—	14.56	0.37	19.1	7	Xu et al., 2000
110	Apatite	29.93°	102.22833°	1356	Proterozoic	—	—	—	14.2	5.5	Xu et al., 2000
111	Apatite	29.92917°	102.22833°	1340	Proterozoic	—	—	—	13.4	4.4	Xu et al., 2000
112	Apatite	29.92611°	102.23333°	1394	Proterozoic	—	—	—	11.4	5.8	Xu et al., 2000
113	Apatite	29.84°	102.24167°	2608	Proterozoic	—	—	—	10.5	10.8	Xu et al., 2000
114	Apatite	31.15333°	101.90333°	2240	—	granite	13.28	0.24	7.1	1.5	Xu et al., 2000
115	Apatite	30.59667°	102.67111°	2880	—	sandstone	10.1	1.25	7.7	1.7	Xu et al., 2000
116	Apatite	31.02833°	102.24167°	2800	—	sandstone	13.18	0	12	6.6	Xu et al., 2000
117	Apatite	30.98°	102.69333°	2720	—	sandstone	13.39	0.29	12	1.9	Xu et al., 2000
118	Apatite	30.96167°	102.8825°	3800	—	sandstone	—	—	14.5	3.6	Xu et al., 2000

119	Apatite	30.91333°	102.9°	4487	—	sandstone	11.51	0.89	17.1	2.3	Xu et al., 2000
QMG-23	Apatite	37°48.516'	90°50.937'	3346	Mesozoic	granodiorite	11.76	—	150.9	—	Liu et al., 2017
QMG-21	Apatite	37°48.599'	90°50.879'	3443	Mesozoic	granodiorite	11.42	—	115.82	—	Liu et al., 2017
QMG-28	Apatite	37°48.732'	90°50.825'	3538	Mesozoic	granodiorite	11.95	—	125.99	—	Liu et al., 2017
QMG-17	Apatite	37°48.702'	90°50.720'	3626	Mesozoic	granodiorite	11.5	—	38.72	—	Liu et al., 2017
QMG-13	Apatite	37°48.964'	90°50.931'	3743	Mesozoic	granodiorite	12.61	—	169.95	—	Liu et al., 2017
QMG-11	Apatite	37°51.268'	90°12.280'	4319	Lower Palaeozoic	monzonitic granite	12.79	—	29.21	—	Liu et al., 2017
QMG-8	Apatite	37°51.191'	90°12.209'	4424	Lower Palaeozoic	monzonitic granite	12.78	—	50.43	—	Liu et al., 2017
QMG-6	Apatite	37°51.060'	90°12.171'	4500	Lower Palaeozoic	monzonitic granite	13.32	—	25.5	—	Liu et al., 2017
SY01	Apatite	37°14'28"	120°14'13"	101	Mesozoic	granodiorite	—	—	66.8	8.7	Liu ZJ et al., 2010
JJ02	Apatite	37°23'35"	120°36'38"	134	Mesozoic	granite	—	—	64.2	6.2	Liu ZJ et al., 2010
WTK01	Apatite	37°20'57"	120°30'52"	173	Mesozoic	granite	—	—	79.9	5.1	Liu ZJ et al., 2010
WZ01	Apatite	37°27'26"	120°49'08"	138	Mesozoic	granite	—	—	91.8	23.6	Liu ZJ et al., 2010
CZ02	Apatite	37°24'13"	120°14'07"	108	Mesozoic	granite	—	—	39.9	5.1	Liu ZJ et al., 2010
XLU01	Apatite	37°15'27"	120°28'58"	132	Mesozoic	granodiorite	—	—	82	5.5	Liu ZJ et al., 2010
XL02	Apatite	37°22'13"	119°'"5532	1	Mesozoic	granodiorite	—	—	71.6	5.6	Liu ZJ et al., 2010
SSD01	Apatite	37°24'38"	119°56'51"	5	Mesozoic	granodiorite	—	—	135	25.1	Liu ZJ et al., 2010
BG01	Apatite	37°13'46"	120°33'28"	143	Mesozoic	granite	—	—	76.3	6.1	Liu ZJ et al., 2010
DIH01	Apatite	37°35'03"	120°01'45"	148	Mesozoic	granodiorite	—	—	77.9	4.5	Liu ZJ et al., 2010
XD01	Apatite	37°08'45"	120°19'51"	124	Mesozoic	granite	—	—	88.5	7.8	Liu ZJ et al., 2010
ZJG01	Apatite	37°34'34"	120°46'07"	113	Mesozoic	granite	—	—	66.3	4.5	Liu ZJ et al., 2010
BJS01	Apatite	37°06'43"	119°56'45"	136	Mesozoic	granite	—	—	84.4	6.1	Liu ZJ et al., 2010
DJJ01	Apatite	37°27'47"	120°30'18"	293	Mesozoic	granite	—	—	81.7	5.6	Liu ZJ et al., 2010
NS01	Apatite	37°09'23"	120°06'17"	122	Mesozoic	granite	—	—	88.8	4.7	Liu ZJ et al., 2010
JJ01	Apatite	37°19'38"	120°36'03"	201	Mesozoic	granite	—	—	102.7	12.2	Liu ZJ et al., 2010
WLZ01	Apatite	37°10'42"	120°00'04"	141	Mesozoic	granite	—	—	90.8	8.3	Liu ZJ et al., 2010
XL01	Apatite	37°23'13"	119°56'19"	0	Mesozoic	granite	—	—	92.4	5.9	Liu ZJ et al., 2010
SY02	Apatite	37°14'50"	120°18'52"	156	Mesozoic	granodiorite	—	—	79.3	5.6	Liu ZJ et al., 2010
YD01	Apatite	37°13'50"	120°08'51"	67	Mesozoic	granodiorite	—	—	97.6	8.2	Liu ZJ et al., 2010
CZ01	Apatite	37°23'46"	120°16'36"	130	Mesozoic	granodiorite	—	—	90.8	5.9	Liu ZJ et al., 2010

CG01	Apatite	37°13'27"	120°03'35"	65	Mesozoic	granodiorite	—	—	91.1	5.7	Liu ZJ et al., 2010
CZ03	Apatite	37°23'45"	120°10'46"	108	Mesozoic	granodiorite	—	—	101.4	6.7	Liu ZJ et al., 2010
LFSH01	Apatite	36°52'52"	119°56'38"	246	Mesozoic	granite	—	—	115	14.2	Liu ZJ et al., 2010
GJD01	Apatite	37°06'24"	120°05'32"	179	Mesozoic	granite	—	—	91.7	9.1	Liu ZJ et al., 2010
QTS01	Apatite	37°00'22"	119°59'31"	179	Mesozoic	granite	—	—	74.5	4.5	Liu ZJ et al., 2010
DHF01	Apatite	36°54'30"	120°11'40"	137	Mesozoic	granite	—	—	96.9	5.8	Liu ZJ et al., 2010
CZD01	Apatite	36°49'54"	120°04'59"	96	Mesozoic	granite	—	—	76.8	4.8	Liu ZJ et al., 2010
SZ01	Apatite	37°26'30"	120°11'16"	81	Mesozoic	granite	—	—	88.3	7.5	Liu ZJ et al., 2010
XDJ01	Apatite	37°32'52"	120°30'46"	143	Mesozoic	granite	—	—	72.7	4.4	Liu ZJ et al., 2010
YJK01	Apatite	37°33'51"	120°55'42"	100	Mesozoic	granite	—	—	92.7	5	Liu ZJ et al., 2010
GJL01	Apatite	37°30'34"	120°52'59"	139	Mesozoic	granite	—	—	99	6.2	Liu ZJ et al., 2010
ZX01	Apatite	37°27'31"	120°10'44"	36	Mesozoic	granite	—	—	89.2	5.4	Liu ZJ et al., 2010
DQJ01	Apatite	37°21'23"	120°27'08"	83	Mesozoic	granite	—	—	104	7.3	Liu ZJ et al., 2010
WZ03	Apatite	37°27'01"	120°46'49"	220	Mesozoic	granodiorite	—	—	81.5	4	Liu ZJ et al., 2010
NS02	Apatite	37°09'02"	120°06'55"	139	Mesozoic	granite	—	—	77.9	4.2	Liu ZJ et al., 2010
LJ01	Apatite	37°27'18"	120°33'35"	203	Mesozoic	granite	—	—	87.8	6.6	Liu ZJ et al., 2010
JQ01	Apatite	37°26'52"	120°31'53"	230	Mesozoic	granite	—	—	88.8	6.3	Liu ZJ et al., 2010
TJ01	Apatite	37°29'18"	120°36'02"	82	Mesozoic	granite	—	—	71.9	4	Liu ZJ et al., 2010
WZ02	Apatite	37°27'54"	120°47'36"	163	Mesozoic	granite	—	—	76.7	3.8	Liu ZJ et al., 2010
LJH01	Apatite	37°24'21"	120°33'51"	154	Mesozoic	granite	—	—	73.8	5	Liu ZJ et al., 2010
SJD01	Apatite	37°24'21"	120°33'51"	154	Mesozoic	granite	—	—	119.3	10.2	Liu ZJ et al., 2010
YJK02	Apatite	37°36'20"	120°57'09"	47	Mesozoic	granite	—	—	75.8	4.1	Liu ZJ et al., 2010
GJL02	Apatite	37°32'48"	120°52'38"	124	Mesozoic	granite	—	—	84.5	4.5	Liu ZJ et al., 2010
GJZ01	Apatite	37°30'34"	120°52'59"	139	Mesozoic	granite	—	—	76.9	4.3	Liu ZJ et al., 2010
FHS01	Apatite	36°50'08"	119°59'41"	134	Mesozoic	granite	—	—	69.8	5.1	Liu ZJ et al., 2010
DZS01	Apatite	36°58'17"	119°54'24"	104	Mesozoic	granite	—	—	77.1	4.9	Liu ZJ et al., 2010
JL01	Apatite	37°21'57"	120°20'05"	159	Mesozoic	granite	—	—	84.9	5.3	Liu ZJ et al., 2010
XJG01	Apatite	37°41'07"	120°45'39"	162	Mesozoic	granite	—	—	86	5.2	Liu ZJ et al., 2010
XM01	Apatite	36°58'50"	120°05'37"	97	Mesozoic	granite	—	—	72.9	4.2	Liu ZJ et al., 2010
MS01	Apatite	37°12'41"	120°10'21"	163	Mesozoic	granite	—	—	76.6	5.2	Liu ZJ et al., 2010

JD01	Apatite	37°02'00"	120°11'25"	192	Mesozoic	granite	—	—	67.6	4.6	Liu ZJ et al., 2010
AL01	Apatite	36°56'16"	120°13'55"	145	Mesozoic	granite	—	—	82.4	5.5	Liu et al., 2010(b)
ZF98-b1	Apatite	25°25'	105°27'20"	—	Upper Triassic	claystone	11.8	2.1	67.2	3.8	Hu, 2011
ZF115-b1	Apatite	25°21'	105°30'10"	—	Upper Triassic	claystone	12.1	1.7	96.7	5.3	Hu, 2011
ZF96-b1	Apatite	25°26'	105°27'40"	—	Upper Triassic	claystone and sandstone	11.4	2.2	72.3	9.1	Hu, 2011
YB353-b1	Apatite	25°18'20"	105°29'55"	—	Upper Triassic	claystone and sandstone	—	—	78.8	4.4	Hu, 2011
YB352-b1	Apatite	25°18'30"	105°29'50"	—	Upper Triassic	claystone and sandstone	—	—	68.5	4	Hu, 2011
ZF95-b1	Apatite	25°25'30"	105°27'30"	—	Upper Triassic	claystone and sandstone	—	—	67.2	4.7	Hu, 2011
YB19-b1	Apatite	25°58'	105°50'	—	Middle Triassic	claystone and sandstone	—	—	54.1	9.1	Hu, 2011
YB42-b1	Apatite	24°56'	105°48'	—	Middle Triassic	—	11.6	0.1	47.2	6.2	Hu, 2011
YB851-b1	Apatite	24°12'	105°50'	—	Middle Triassic	—	—	—	53.3	3.6	Hu, 2011
YB233-b1	Apatite	24°55'	105°49'	—	Middle Triassic	—	11.6	2.1	38.6	4	Hu, 2011
YB1-b1	Apatite	24°59'	105°48'	—	Middle Triassic	—	—	—	73.3	10.2	Hu, 2011
99711-1	Apatite	38°20.456'	89°17.701'	4196	Middle Proterozoic	gneiss	—	—	36.4	3.2	Chen et al., 2001
99713-1	Apatite	38°20.434'	89°18.085'	3993	Middle Proterozoic	gneiss	10.01	3.14	31.6	3	Chen et al., 2001
99722-2	Apatite	38°22.592'	89°17.491'	3691	Middle Proterozoic	gneiss	11.97	2.47	27.1	2.6	Chen et al., 2001
99725-2	Apatite	38°28.729'	89°15.898'	3652	—	granite	12.11	2.14	33.9	3.2	Chen et al., 2001
99728	Apatite	38°29.284'	89°16.927'	3481	—	granite	11.52	2.38	26.7	2.3	Chen et al., 2001
99732-1	Apatite	38°42.692'	89°12.593'	3298	—	granite	10.64	1.96	22.4	2.1	Chen et al., 2001
99731-2	Apatite	38°42.371'	89°12.515	3100	—	granite	—	—	13.8	2.2	Chen et al., 2001
99738	Apatite	38°50.916'	89°09.488'	2914	Middle Proterozoic	gneiss	—	—	19.7	2.8	Chen et al., 2001
997310	Apatite	38°52.096'	89°06.660'	2627	—	granite	—	—	18.8	2.3	Chen et al., 2001
997314	Apatite	38°54.3 90'	89°05.293'	2271	—	granite	10.12	1.99	28.4	2.7	Chen et al., 2001
99725-3	Apatite	38°28.630'	89°16.029'	3600	—	granite	12.2	0.7	66.1	6.9	Chen et al., 2006a
99724	Apatite	38°28.069'	89°17.515'	3500	—	granite	10.7	0.8	17.3	1.9	Chen et al., 2006a
99734-1	Apatite	38°43.921'	89°07.206'	3194	—	granite	11	0.8	16.7	0.8	Chen et al., 2006a
99734-2	Apatite	38°44.033'	89°07.090'	3115	—	granite	11.2	0.8	42	4.1	Chen et al., 2006a
997311-1	Apatite	38°52.543'	89°06.590'	2636	—	granite	10.6	0.8	11.1	1.2	Chen et al., 2006a
997312	Apatite	38°52.942'	89°06.090'	2438	—	granite	10.9	0.8	10.1	1.2	Chen et al., 2006a
03806	Apatite	37°15'33'	85°24'45"	3100	—	diorite	12.3	0.2	5.5	0.9	Chen et al., 2006a

03821	Apatite	37°16'59"	85°27'14"	3100	—	diorite	12.5	0.3	4.6	0.9	Chen et al., 2006a
03831	Apatite	37°16'20"	85°26'51"	3220	—	diorite	12	0.3	10.20	1.8	Chen et al., 2006a
03853	Apatite	37°17'47"	85°28'49"	2956	—	granite	12.7	0.3	1.8	0.4	Chen et al., 2006a
03856	Apatite	37°17'25"	85°29'13"	3048	—	granite	12.1	0.3	2.1	0.6	Chen et al., 2006a
03864	Apatite	37°16'46"	85°30'01"	3145	—	diorite	12.3	0.3	7.3	1.1	Chen et al., 2006a
03882	Apatite	37°15'41"	85°30'39"	3364	—	granite	11.9	0.2	2.1	0.4	Chen et al., 2006a
03891	Apatite	37°15'12"	85°31'40"	3564	—	granite	11.8	0.2	4.5	0.7	Chen et al., 2006a
03893	Apatite	37°15'58"	85°31'52"	3613	—	granite	12.5	0.2	2	0.6	Chen et al., 2006a
03901	Apatite	37°14'49"	85°32'01"	3674	—	granite	12.3	0.2	8.1	10	Chen et al., 2006a
Y04-1	Apatite	43º26'43.3"	81º00'54.0"	1880	Upper Palaeozoic	granite	11.85	0.19	106.7	10.6	Chen et al., 2006b
Y04-2	Apatite	43º31'42.6"	81º30'28.7"	1800	Upper Palaeozoic	granite	10.48	0.29	116.8	11.3	Chen et al., 2006b
Y04-3	Apatite	43º27'18."	81º00'41.2"	1750	Upper Palaeozoic	granite	9.66	0.31	24.1	3.1	Chen et al., 2006b
Y09-1	Apatite	43º30'03.5"	81º07'02.2"	1702	Carboniferous	tuff	11.05	1.17	45.3	5.3	Chen et al., 2006b
Y09-2	Apatite	43º28'37.0"	81º06'47.0"	2072	Carboniferous	volcanic breccia	12.08	0.73	47	8.5	Chen et al., 2006b
Y09-5	Apatite	43º26'28.9"	81º04'82.8"	2450	Carboniferous	rhyolite	13.76	0.19	86.5	9.5	Chen et al., 2006b
Y10-1	Apatite	43º14'58.4"	81º05'46.5"	2166	Upper Palaeozoic	granite	11.33	0.16	122.8	8.8	Chen et al., 2006b
Y10-3	Apatite	43º13'30.5"	81º07'17.8"	2100	Upper Palaeozoic	granite	11.33	0.21	156.7	10.2	Chen et al., 2006b
Y10-4	Apatite	43º12'15.2"	81º07'41.3"	1999	Upper Palaeozoic	granite	11.99	0.24	158.3	12.3	Chen et al., 2006b
Y12-2	Apatite	43º25'12.6"	81º53'56.5"	1304	—	—	12.85	0.22	85.3	9.3	Chen et al., 2006b
Y12-3	Apatite	43º24'38.8"	81º53'26.4"	1342	Upper Palaeozoic	orthophyre	12.05	0.33	89.3	10.2	Chen et al., 2006b
Y12-6	Apatite	43º22'37.1"	81º51'48.2"	1515	Upper Palaeozoic	orthophyre	12.25	0.18	111.6	12.9	Chen et al., 2006b
