# Supplementary material for: Uplift and denudation in the continental area of China linked to climatic effects: evidence from apatite and zircon fission track data
Source: Sci Rep. 2018 Jun 22;8:9546. doi: 10.1038/s41598-018-27801-7 (PMC6015080; doi:10.1038/s41598-018-27801-7)
Supplement: Supplementary file 2 — Dataset 2 [file 41598_2018_27801_MOESM2_ESM.pdf]

Uplift and denudation in continental area of China linked to climatic effects: evidence from apatite and zircon fission track data

Nansheng Qiu[1,2,*], Shuai Liu[1,2]

1. State Key Laboratory of Petroleum Resources and Prospecting, China University of Petroleum, Beijing, 102249, China
2. College of Geosciences, China University of Petroleum, Beijing 102249, China

Supplement 2. ZFT Dataset

Zircon Fission Track Dafa of Continental China

Sample	Mineral	Latitude	Longtitude	Altitude(m)	Strata	Lithology	Fission Track Age(Ma)	1σ	Reference
BO-1	Zircon	30°24'49"	102°45'07"	1604	Silurian	schist	13.2	1.00	Tan et al., 2014
BO-2	Zircon	30°24'24"	102°46'12"	2286	Silurian	schist	80.6	8.50	Tan et al., 2014
BO-3	Zircon	30°33'17"	102°53'58"	1157	Proterozoic	schist	13.2	1.10	Tan et al., 2014
BX-5	Zircon	30°49'53"	102°43'57"	2798	Triassic	sandstone	108	9.10	Tan et al., 2014
BX-9	Zircon	30°38'32"	102°47'23"	1955	Proterozoic	metaquartz	14.1	0.90	Tan et al., 2014
BX-11	Zircon	30°36'11"	102°52'04"	2154	Proterozoic	granite	25.4	2.40	Tan et al., 2014
BX-13	Zircon	30°31'44"	102°54'47"	1416	Proterozoic	granite	27.7	2.00	Tan et al., 2014
BX-14	Zircon	30°26'52"	102°51'31"	1254	Proterozoic	granite	43.6	5.50	Tan et al., 2014
BX-16	Zircon	30°13'54"	102°50'19"	804	Triassic	sandstone	107.6	7.30	Tan et al., 2014
BX110906	Zircon	30°12'23"	102°51'39"	1204	Eocene	sandstone	199.3	11.40	Tan et al., 2014
BX110907	Zircon	30°13'29"	102°50'23"	770	Triassic	sandstone	129.2	8.80	Tan et al., 2014
BX110913	Zircon	30°25'09"	102°43'36"	1378	Silurian	sandstone	28.5	1.40	Tan et al., 2014
GD-1	Zircon	31°03'41"	103°24'20"	1219	Proterozoic	granite	76.4	5.70	Tan et al., 2014
GD-2	Zircon	31°03'46"	103°20'18"	1451	Proterozoic	granite	64.7	8.40	Tan et al., 2014
GD-3	Zircon	30°52'57"	102°59'10"	2690	Triassic	sandstone	71.3	9.30	Tan et al., 2014
GD-6	Zircon	30°53'17"	102°58'21"	3460	Triassic	sandstone	75.7	7.80	Tan et al., 2014
GD-10	Zircon	30°54'38"	102°53'39"	4389	Triassic	sandstone	81.9	7.80	Tan et al., 2014
GD-11	Zircon	30°58'39"	102°51'49"	3608	Triassic	sandstone	63.3	5.90	Tan et al., 2014
DB-1	Zircon	30°02'25"	102°10'33"	1430	Proterozoic	granodiorite	135.5	9.70	Tan et al., 2014
DB-2	Zircon	30°12'55"	102°12'07"	1679	Proterozoic	granodiorite	82.2	6.10	Tan et al., 2014
WCH-1	Zircon	31°19'39"	103°23'55"	1766	Proterozoic	granite	10.6	0.90	Tan et al., 2014
Gong-41	Zircon	30°02'25"	102°10'33"	1488	Proterozoic	granodiorite	90.2	5.20	Tan et al., 2014
Gong-78	Zircon	31°19'20"	102°43'45"	2444	Triassic	sandstone	195	11.60	Tan et al., 2014
Gong-83	Zircon	30°18'51"	102°47'08"	956	Proterozoic	granodiorite	33	2.50	Tan et al., 2014
Gong-85	Zircon	30°41'19"	102°45'05"	2133	Devonian	sandstone	31.6	2.20	Tan et al., 2014
Gong-90	Zircon	30°33'26"	102°53'12"	1533	Proterozoic	gneiss	9.6	0.70	Tan et al., 2014

Gong-91	Zircon	30°31'32"	102°54'55"	1462	Proterozoic	granite	25.1	1.40	Tan et al., 2014
Gong-92	Zircon	30°30'43"	102°54'54"	1366	Proterozoic	granite	25.6	1.70	Tan et al., 2014
Gong-95	Zircon	30°24'33"	102°50'10"	1066	Proterozoic	granite	56.3	3.60	Tan et al., 2014
Kc0510	Zircon	30°30'21"	102°50'22"	2045	Proterozoic	granite	13.6	1.30	Tan et al., 2014
Kc0755	Zircon	30°22'19"	102°49'16"	1233	Proterozoic	granite	14.2	0.80	Tan et al., 2014
Kc0513	Zircon	30°33'29"	102°53'34"	2352	Proterozoic	granite	11.9	1.30	Tan et al., 2014
Kc0756	Zircon	30°25'08"	102°45'43"	1180	Proterozoic	granite	10.9	0.70	Tan et al., 2014
Kc0757	Zircon	30°34'43"	102°52'44"	1575	Proterozoic	granite	13.4	0.70	Tan et al., 2014
WCH-8	Zicron	31°07'01"	103°45'00"	822	Jurassic-Cretaceous	sandstone	58.70	3.40	Tan et al., 2015
LK-4	Zicron	31°28'12"	104°09'14"	659	Jurassic-Cretaceous	sandstone	89.20	9.90	Tan et al., 2015
JH-5	Zicron	31°24'36"	104°01'19"	1090	Jurassic-Cretaceous	sandstone	86.20	7.90	Tan et al., 2015
WCH-6	Zicron	31°03'07"	103°28'48"	884	Palaeozoic	sandstone	171.20	10.20	Tan et al., 2015
JH-1	Zicron	31°18'36"	104°03'29"	672	Jurassic-Cretaceous	conglomerate	240.60	55.90	Tan et al., 2015
JH-2	Zicron	31°17'13"	104°01'55"	725	Jurassic-Cretaceous	conglomerate	144.80	19.5	Tan et al., 2015
LJ2-1	Zicron	29°41'42"	91°45'00"	4710	—	altered sandstone	20.90	2.00	Yuan et al., 2002b
LJ1-2	Zicron	29°41'42"	91°44'54"	4850	—	altered sandstone	341.60	79.10	Yuan et al., 2002b
NGL3	Zicron	29°15'12"	91°48'24"	3690	—	volcanic breccia	42.90	5.50	Yuan et al., 2002b
LDG1	Zicron	29°22'00"	88°24'12"	4180	—	ore	44.10	4.20	Yuan et al., 2002b
NGL3	Zicron	29°15'12"	91°48.4'	3690	Upper Jurassic	sandstone	42.90	2.20	Yuan et al., 2002a
LL12	Zicron	29°14'06"	90°27'54"	4060	Upper Triassic	sandstone	93.70	7.10	Yuan et al., 2002a
LL5-2	Zicron	29°05'30"	90°23'30"	4470	Upper Triassic	slate	53.30	2.70	Yuan et al., 2002a
LL1-2	Zicron	28°59'06"	90°23'36"	4510	Upper Triassic	slate	60.40	6.30	Yuan et al., 2002a
Q1	Zicron	34°26'00"	87°50'00"	5052	Lower Triassic	sandstone	142.00	14.00	Song et al., 2014
Q2	Zicron	34°55'00"	87°48'00"	5080	Middle Jurassic	sandstone	159.00	15.00	Song et al., 2014
Q3	Zicron	34°48'00"	87°32'00"	4991	Lower Triassic	sandstone	199.00	18.00	Song et al., 2014
Q4	Zicron	34°47'00"	87°33'00"	5040	Lower Triassic	tuff	164.00	14.00	Song et al., 2014
Q5	Zicron	33°44'00"	87°40'00"	5200	Lower Triassic	sandstone	142.00	11.00	Song et al., 2014
Q6	Zicron	33°43'00"	87°44'00"	5150	Middle Jurassic	sandstone	181.00	15.00	Song et al., 2014
Q7	Zicron	33°26'00"	87°31'00"	4928	Lower Triassic	sandstone	168.00	13.00	Song et al., 2014
Q8	Zicron	33°01'00"	87°41'00"	5050	Lower Triassic	sandstone	92.00	10.00	Song et al., 2014

Q9	Zicron	33°01'00"	87°52'00"	5054	Lower Triassic	sandstone	108.00	8.00	Song et al., 2014
Q10	Zicron	33°41'00"	91°51'00"	5010	Lower Triassic	sandstone	104.00	7.00	Song et al., 2014
Q11	Zicron	33°44'00"	91°45'00"	5230	Lower Triassic	sandstone	102.00	7.00	Song et al., 2014
Q12	Zicron	33°44'00"	91°19'00"	5237	Lower Triassic	tuff	68.00	7.00	Song et al., 2014
Q13	Zicron	33°47'00"	91°08'00"	4950	Lower Triassic	sandstone	54.00	8.00	Song et al., 2014
Q14	Zicron	33°48'00"	91°15'00"	5220	Middle Jurassic	sandstone	128.00	12.00	Song et al., 2014
15-1	Zicron	47°54'26"	88°11'44"	—	—	copper ore	247.00	24.00	Yuan et al., 2009
15-2	Zicron	47°54'26"	88°11'44"	—	—	copper ore	312.00	39.00	Yuan et al., 2009
17-2	Zicron	47°52'23"	88°13'56"	—	—	gold ore	141.00	13.00	Yuan et al., 2009
19-2	Zicron	47°44'2"	88°27'3"	—	—	iron ore	158.00	19.00	Yuan et al., 2009
39	Zicron	47°52'33"	88°15'6"	—	—	iron ore	134.00	9.00	Yuan et al., 2009
20-1	Zicron	47°50'50"	88°16'40"	—	—	lead-zinc ore	134.00	9.00	Yuan et al., 2009
57	Zicron	46°50'58"	89°23'47"	—	—	iron-copper ore	207.00	16.00	Yuan et al., 2009
57-3	Zicron	46°50'58"	89°23'47"	—	—	gold ore	202.00	24.00	Yuan et al., 2009
60	Zicron	46°29'4"	90°4'48"	—	—	gold ore	234.00	28.00	Yuan et al., 2009
62-1	Zicron	46°24'23"	90°20'8"	—	—	gold ore	174.00	16.00	Yuan et al., 2009
62-3	Zicron	46°24'23"	90°20'8"	—	—	gold ore	255.00	24.00	Yuan et al., 2009
62-4	Zicron	46°24'23"	90°20'8"	—	—	gold ore	195.00	14.00	Yuan et al., 2009
82-1	Zicron	46°57'14"	89°9'7"	—	—	gold ore	218.00	21.00	Yuan et al., 2009
82-3	Zicron	46°57'14"	89°9'7"	—	—	gold ore	211.00	51.00	Yuan et al., 2009
38-2	Zicron	46° 57'6"	89°9'19"	—	—	gold ore	254.00	29.00	Yuan et al., 2009
38-3	Zicron	46°57'6"	89° 9'19"	—	—	gold ore	339.00	49.00	Yuan et al., 2009
FT01	Zicron	34°27'42.0"	110°26'18.3"	1843	Cretaceous	granite	50.00	4.00	Yu et al., 2013
FT02	Zicron	34°27'50.8"	110°26'09.7"	1685	Cretaceous	granite	62.00	6.00	Yu et al., 2013
FT03	Zicron	34°27'58.4"	110°26'08.3"	1470	Cretaceous	granite	51.00	4.00	Yu et al., 2013
FT04	Zicron	34°28'11.4"	110°26'10.2"	1285	Cretaceous	granite	45.00	3.00	Yu et al., 2013
FT05	Zicron	34°28'37.7"	110°25'58.0"	1087	Cretaceous	granite	45.00	3.00	Yu et al., 2013
FT06	Zicron	34°29'29.9"	110°25'47.4"	886	Cretaceous	granite	47.00	4.00	Yu et al., 2013
FT07	Zicron	34°30'11.4"	110°25'51.2"	686	Cretaceous	granite	48.00	4.00	Yu et al., 2013
FT08	Zicron	34°30'16.2"	110°25'43.9"	632	Cretaceous	granite	43.00	3.00	Yu et al., 2013

2	Zicron	36°15'19"	117°06'04"	1460	Upper Archaeozoic	diorite	123.00	46.00	Li et al., 2006
5	Zicron	36°15'12"	117°06'01"	1290	Upper Archaeozoic	diorite	1012.00	389.00	Li et al., 2006
15	Zicron	36°15'04"	117°06'07"	1180	Upper Archaeozoic	diorite	797.00	92.00	Li et al., 2006
8	Zicron	36°14'40"	117°06'27"	940	Upper Archaeozoic	diorite	252.00	58.00	Li et al., 2006
9	Zicron	36°14'29"	117°06'32"	900	Upper Archaeozoic	diorite	327.00	83.00	Li et al., 2006
16	Zicron	36°14'08"	117°06'31"	755	Upper Archaeozoic	diorite	15.00	1.00	Li et al., 2006
13	Zicron	36°13'14"	117°06'59"	440	Upper Archaeozoic	diorite	567.00	73.00	Li et al., 2006
14	Zicron	36°12'49"	117°07'11"	350	Upper Archaeozoic	diorite	200.00	25.00	Li et al., 2006
YK4900	Zicron	35°38'43"	94°04'13"	4900	Triassic	metasandstone	76.30	6.10	Wang et al., 2010b
YK4815	Zicron	35°38'30"	94°04'15"	4815	Triassic	metasandstone	74.90	5.10	Wang et al., 2010b
YK4665	Zicron	35°41'02"	94°02'55"	4665	Triassic	metasandstone	64.80	5.60	Wang et al., 2010b
YK4515	Zicron	35°41'55"	94°16'41"	4515	Permian	metasandstone	55.50	5.40	Wang et al., 2010b
YK4405	Zicron	35°42'17"	94°16'58"	4405	Permian	metasandstone	50.40	4.10	Wang et al., 2010b
YK-3	Zicron	35°44'12"	94°10'46"	4352	Devonian	monzogranite	67.80	7.70	Wang et al., 2010b
YK4203	Zicron	35°44'23"	94°16'23"	4203	Ordovician-Silurian	metasandstone	62.40	6.40	Wang et al., 2010b
YK4000	Zicron	35°47'46"	94°20'24"	4000	Ordovician-Silurian	metasandstone	62.30	5.70	Wang et al., 2010b
YK3693	Zicron	35°52'15"	94°27'08"	3693	Jurassic	granodiorite	63.10	4.00	Wang et al., 2010b
YK3501	Zicron	35°54'07"	94°37'47"	3501	Lower-Middle Proterozoic	metasandstone	65.60	5.20	Wang et al., 2010b
YK3418	Zicron	35°54'43"	94°46'19"	3418	Middle Triassic	metasandstone	71.20	6.20	Wang et al., 2010b
YK3335	Zicron	35°58'16"	94°49'02"	3335	Permian	metasandstone	64.10	7.50	Wang et al., 2010b
YK-4	Zicron	36°01'53"	94°48'47"	3260	Lower-Middle Proterozoic	diorite	207.60	11.00	Wang et al., 2010b
YK3225	Zicron	36°08'42"	94°46'58"	3225	Permian	monzogranite	76.20	8.20	Wang et al., 2010b
YK3137	Zicron	36°07'59"	94°47'23"	3137	Permian	monzogranite	76.20	8.20	Wang et al., 2010b
YK-5	Zicron	36°08'01"	94°47'19"	3140	Permian	monzogranite	76.20	8.20	Wang et al., 2010b
WK17	Zicron	38°28'27"	75°59'12"	2270	Carboniferous	sandstone	101.00	9.00	Cao et al., 2013
WK18	Zicron	38°30'09"	76°02'01"	2170	Jurassic	sandstone	48.00	4.00	Cao et al., 2013
WK20	Zicron	38°33'06"	76°03'45"	2074	Jurassic	sandstone	157.00	14.00	Cao et al., 2013
WK21	Zicron	38°33'28"	76°04'44"	2021	Jurassic	sandstone	159.00	18.00	Cao et al., 2013
WK22	Zicron	38°33'56"	76°05'35"	2009	Jurassic	sandstone	42.00	12.00	Cao et al., 2013
WK23	Zicron	38°34'24"	76°06'55"	1956	Jurassic	sandstone	181.00	13.00	Cao et al., 2013

WK24	Zircon	38°35'46"	76°08'10"	1897	Jurassic	sandstone	176.00	14.00	Cao et al., 2013
WK75	Zircon	39°14'23"	75°10'27"	2920	Triassic	granite	1.90	0.30	Cao et al., 2013
WK76	Zircon	38°45'05"	75°11'32"	2853	Triassic	granite	1.60	0.10	Cao et al., 2013
WK77	Zircon	38°46'27"	75°14'19"	2603	Devonian	sandstone	3.40	0.50	Cao et al., 2013
WK78	Zircon	38°47'03"	75°18'13"	2394	Devonian	sandstone	170.00	15.00	Cao et al., 2013
WK79	Zircon	38°49'20"	75°21'34"	2350	Devonian	sandstone	154.00	18.00	Cao et al., 2013
WK81	Zircon	38°49'04"	75°28'02"	2120	Carboniferous	diorite	46.00	6.00	Cao et al., 2013
WK82	Zircon	38°53'32"	75°29'39"	1973	Carboniferous	diorite	190.00	14.00	Cao et al., 2013
WK93	Zircon	38°44'55"	75°05'15"	3230	Triassic	gneiss	0.70	0.10	Cao et al., 2013
WK108	Zircon	38°43'49"	75°03'15"	3567	Triassic	gneiss	1.30	0.10	Cao et al., 2013
WK97	Zircon	37°51'50"	75°18'37"	3005	Ordovician	schist	9.50	0.90	Cao et al., 2013
WK100	Zircon	37°53'00"	75°23'38"	3011	Ordovician	schist	7.40	0.40	Cao et al., 2013
WK102	Zircon	37°57'25"	75°16'14"	3520	Triassic	schist	8.10	0.50	Cao et al., 2013
WK103	Zircon	38°00'55"	75°14'43"	3061	Ordovician	gneiss	8.60	0.70	Cao et al., 2013
WK105	Zircon	37°54'50"	75°12'04"	3278	Ordovician	schist	7.90	0.70	Cao et al., 2013
WK68	Zircon	38°06'30"	74°59'06"	3544	Neogene	granitoid	10.80	0.50	Cao et al., 2013
WK69	Zircon	38°07'38"	74°58'44"	3595	Neogene	granitoid	11.00	0.50	Cao et al., 2013
WK72	Zircon	38°25'10"	75°02'32"	3638	Ordovician	metasandstone	13.00	1.00	Cao et al., 2013
WK73	Zircon	38°36'33"	74°58'59"	3308	Jurassic	granitoid	44.00	4.00	Cao et al., 2013
1	Zircon	43°51'18.1"	85°38'25.6"	2175	Carboniferous	tuff	134.10	16.20	Shen et al., 2008
3	Zircon	43°54'2.6"	85°39'47.5"	1548	Lower Permian	andesite	127.10	20.60	Shen et al., 2008
4	Zircon	43°42'31.9"	86°39'52.0"	1977	Jurassic	sandstone	118.60	14.20	Shen et al., 2008
5	Zircon	43°26'51.5"	87°40'14.5"	1425	Carboniferous	tuff	131.70	12.20	Shen et al., 2008
DB01	Zircon	32°16'00"	107°55'19"	—	Middle Jurassic	feldspar-rich sandstone	209.20	13.30	Xu et al., 2010
DB02	Zircon	32°16'26"	107°56'18"	—	Lower Jurassic	quartz-feldspar sandstone	209.40	12.90	Xu et al., 2010
DB03	Zircon	32°16'28"	107°56'31"	—	Upper Triassic	quartz-feldspar sandstone	178.70	10.50	Xu et al., 2010
DH1	Zircon	43°12'29"	117°40'23"	1030	Jurassic-Lower Cretaceous	granite	88.00	7.00	Li XM et al., 2011
DH4	Zircon	43°36'33"	117°25'42"	1540	Upper Jurassic	moyite	89.00	7.00	Li XM et al., 2011
DH5	Zircon	43°57'15"	117°31'19"	1750	Jurassic-Lower Cretaceous	monzogranite	90.00	5.00	Li XM et al., 2011
DH6	Zircon	43°58'20"	117°33'12"	1728	Jurassic-Lower Cretaceous	granite	87.00	6.00	Li XM et al., 2011

DH8	Zircon	43°51'41"	117°55'58"	1090	Jurassic-Lower Cretaceous	granodiorite	89.00	6.00	Li XM et al., 2011
DH9	Zircon	43°46'50"	117°53'23"	1110	Jurassic-Lower Cretaceous	diorite	87.00	6.00	Li XM et al., 2011
DH10	Zircon	43°44'47"	117°55'07"	1035	Jurassic-Lower Cretaceous	granite porphyry	88.00	7.00	Li XM et al., 2011
DH11	Zircon	43°25'04"	118°05'59"	960	Jurassic-Lower Cretaceous	granite	88.00	8.00	Li XM et al., 2011
DH12	Zircon	43°49'22"	119°18'31"	659	Jurassic-Lower Cretaceous	granite	88.00	6.00	Li XM et al., 2011
DH13	Zircon	44°15'00"	119°09'26"	633	Jurassic-Lower Cretaceous	monzonite	87.00	7.00	Li XM et al., 2011
DH14	Zircon	44°28'33"	118°53'22"	1050	Lower Triassic-Jurassic	diorite	85.00	7.00	Li XM et al., 2011
DH15	Zircon	44°12'57"	119°15'32"	619	Jurassic-Lower Cretaceous	granite	90.00	9.00	Li XM et al., 2011
DH17	Zircon	44°56'20"	120°24'31"	635	Jurassic-Lower Cretaceous	granodiorite	89.00	8.00	Li XM et al., 2011
DH19	Zircon	45°26'58"	120°28'41"	836	Upper Jurassic-Lower Cretaceous	riebeckite granite	87.00	7.00	Li XM et al., 2011
DH20	Zircon	45°58'09"	121°40'39"	448	Triassic-Jurassic	diorite	87.00	9.00	Li XM et al., 2011
DH22A	Zircon	45°27'47"	121°01'13"	478	Upper Permian	quartz diorite	86.00	6.00	Li XM et al., 2011
DH22B	Zircon	45°27'47"	121°01'13"	—	Upper Permian	granodiorite	84.00	5.00	Li XM et al., 2011
DH23	Zircon	41°50'35"	121°09'41"	268	Jurassic-Lower Cretaceous	diorite	97.00	8.00	Li XM et al., 2011
DH24	Zircon	46°13'33"	121°28'56"	389	Triassic	granite	84.00	5.00	Li XM et al., 2011
DH25	Zircon	46°18'12"	121°17'43"	435	Jurassic-Lower Cretaceous	granite	90.00	7.00	Li XM et al., 2011
DH26	Zircon	47°18'56"	119°45'47"	867	Upper Jurassic	granite	87.00	5.00	Li XM et al., 2011
DH28	Zircon	49°30'18"	117°37'53"	652	Jurassic-Lower Cretaceous	quartz monzonite	85.00	8.00	Li XM et al., 2011
DH30	Zircon	50°32'14"	119°43'16"	730	Upper Permian	monzogranite	85.00	5.00	Li XM et al., 2011
DH31	Zircon	50°46'16"	121°29'35"	723	Jurassic-Lower Cretaceous	granite porphyry	88.00	6.00	Li XM et al., 2011
DH33	Zircon	49°05'03"	121°01'33"	698	Triassic	monzogranite porphyry	87.00	6.00	Li XM et al., 2011
DH35	Zircon	48°00'11"	122°46'19"	354	Lower Cretaceous	hornblende monzogranite	84.00	5.00	Li XM et al., 2011
DH36	Zircon	47°33'24"	122°51'05"	353	Lower Cretaceous	alkaline granite	94.00	6.00	Li XM et al., 2011
DH37	Zircon	47°33'18"	122°35'49"	349	Triassic	porphyritic granite	87.00	8.00	Li XM et al., 2011
DH38	Zircon	47°45'09"	122°21'39"	414	Jurassic-Lower Cretaceous	granodiorite	87.00	7.00	Li XM et al., 2011
DH40	Zircon	50°25'19"	124°07'11"	403	Triassic	moyite	88.00	8.00	Li XM et al., 2011
DH41	Zircon	50°33'47"	125°41'26"	463	Lower Cretaceous	biotite monzogranite	86.00	7.00	Li XM et al., 2011
DH42	Zircon	50°35'25"	124°16'26"	459	Lower Jurassic-Cretaceous	plagiogranite	87.00	7.00	Li XM et al., 2011
DH43	Zircon	50°42'36"	124°18'32"	426	Permian	monzogranite	93.00	6.00	Li XM et al., 2011
DH45	Zircon	52°19'42"	124°41'03"	378	Carboniferous	monzogranite	93.00	6.00	Li XM et al., 2011

DH46	Zircon	52°59'10"	122°28'43"	507	Triassic	diorite porphyry	95.00	6.00	Li XM et al., 2011
ZAY06	Zircon	28°07'20"	86°36'08"	5793	—	granite	15.20	1.00	Gao et al., 2014
ZAY07	Zircon	28°06'07"	86°37'16"	6022	—	monzonite	17.10	1.30	Gao et al., 2014
ZAY09	Zircon	28°06'15"	86°37'27"	6227	—	granulite	11.20	0.80	Gao et al., 2014
ZAY10	Zircon	28°06'19"	86°37'31"	6328	—	granite	13.80	2.00	Gao et al., 2014
ZAY11	Zircon	28°06'24"	86°37'31"	6435	—	pegmatite	14.30	0.90	Gao et al., 2014
T1	Zircon	28°28'01"	86°09'45"	4551	Triassic	sandstone	188.70	24.10	Wang et al., 2010a
T2	Zircon	28°21'54"	86°05'34"	4365	—	granite	13.40	0.50	Wang et al., 2010a
T5	Zircon	28°13'10"	85°59'14"	3975	Lower-Middle Proterozoic	graniteic gneiss	14.90	0.50	Wang et al., 2010a
T6	Zircon	28°11'31"	85°59'06"	3848	Lower-Middle Proterozoic	graniteic gneiss	16.10	0.90	Wang et al., 2010a
T7	Zircon	28°09'45"	85°59'01"	3753	Lower-Middle Proterozoic	granite	12.60	0.50	Wang et al., 2010a
T8	Zircon	28°08'15"	85°58'19"	3770	Lower-Middle Proterozoic	biotitic plagiogneiss	7.40	0.30	Wang et al., 2010a
T9	Zircon	28°06'02"	85°59'40"	3447	Lower-Middle Proterozoic	granitic mylonite	4.70	0.30	Wang et al., 2010a
T10	Zircon	28°04'12"	85°59'51"	3140	Lower-Middle Proterozoic	granitic mylonite	3.90	0.20	Wang et al., 2010a
T11	Zircon	28°02'26"	85°59'13"	2917	Lower-Middle Proterozoic	biotitic plagiogneiss	3.00	0.40	Wang et al., 2010a
T12	Zircon	28°01'28"	85°59'05"	2660	Lower-Middle Proterozoic	biotitic plagiogneiss	3.50	0.20	Wang et al., 2010a
FY03	Apatite	37°31'05"	111°06'45"	930	Archaeozoic	sandstone	130.00	11.00	Zhao JF et al., 2016
NW03	Zircon	38°43'55"	112°12'27"	1610	Triassic	sandstone	184.00	15.00	Zhao JF et al., 2016
LF06	Zircon	37°57'13"	112°04'16"	1042	Carboniferous-Permian	sandstone	128.00	7.00	Zhao JF et al., 2016
BG2	Zircon	43°48'4.8"	87°43'11.3"	938	Upper Triassic	sandstone	152.30	14.70	Shen et al., 2006
BG3	Zircon	43°41'38.6"	87°55'7.9"	1666	Carboniferous	tuff	123.90	13.10	Shen et al., 2006
BG4	Zircon	44°03'17.5"	88°19'49.0"	1087	Upper Permian	truff-sandstone	113.10	10.60	Shen et al., 2006
BG5	Zircon	44°04'11.7"	88°20'58.7"	998	Upper Permian	truff-sandstone	106.30	9.40	Shen et al., 2006
D311/1	Zircon	46°34'45.1"	86°10'43.4"	1250	Hercynian	granite	130.00	14.00	Li W et al., 2010
D407/1	Zircon	45°38'06.9"	90°22'07.4"	1580	Permian	granite	153.00	9.00	Li W et al., 2010
D407/5	Zircon	45°38'23.4"	90°22'02.5"	1328	Permian	granite	126.00	8.00	Li W et al., 2010
BJ13A3	Zircon	53°24'41.1"	122°17'7.5"	681	Upper Jurassic	sandstone	95.40	9.40	Sun et al., 2016
BJ14A1	Zircon	53°11'7.9"	122°14'22"	708		granite	88.90	11.30	Sun et al., 2016
BJ14A3	Zircon	53°17'21.2"	122°12'29.5"	721	Upper Jurassic	sandstone	136.40	15.70	Sun et al., 2016
BJ14A8	Zircon	53°10'02.1"	122°17'41.5"	646		granite	58.00	12.10	Sun et al., 2016

BJ14A9	Zircon	53°09'03.4"	122°19'14.2"	654	Upper Jurassic	sandstone	133.70	15.30	Sun et al., 2016
BJ14B8B	Zircon	53°00'47.2"	122°26'21.8"	709	—	—	143.90	18.70	Sun et al., 2016
BJ15A4A	Zircon	52°49'36.2"	122°16'27.1"	853	—	—	96.40	15.80	Sun et al., 2016
BJ15A6A	Zircon	52°48'07.1"	122°10'29.2"	765	—	—	110.90	15.70	Sun et al., 2016
BJ15B3A	Zircon	52°39'28.8"	121°52'22.1"	521	—	—	140.00	16.50	Sun et al., 2016
BJ15B3B	Zircon	52°39'28.8"	121°52'22.1"	521	—	—	84.90	10.90	Sun et al., 2016
BJ17A1B	Zircon	53°08'30.2"	124°19'45.8"	642	—	—	132.90	15.10	Sun et al., 2016
BJ17A3	Zircon	53°06'52.1"	124°24'21.3"	768	—	—	98.50	8.10	Sun et al., 2016
BJ17A5	Zircon	52°59'32.1"	124°35'01.4"	582	—	—	132.30	17.90	Sun et al., 2016
BJ17A8	Zircon	53°02'40.6"	124°41'10.4"	451	—	—	109.60	14.20	Sun et al., 2016
BJ17B2	Zircon	53°06'37.4"	124°45'09"	714	—	—	102.40	14.40	Sun et al., 2016
BJ18A1	Zircon	53°08'41.8"	124°17'26.2"	686	—	—	135.70	15.20	Sun et al., 2016
BJ18A6	Zircon	53°14'32.3"	124°08'18"	407	—	—	86.20	9.50	Sun et al., 2016
BJ18A6F	Zircon	53°14'32.3"	124°08'18"	407	—	—	99.90	15.30	Sun et al., 2016
BJ18B1	Zircon	53°16'12.8"	123°46'56.2"	706	—	—	138.30	15.10	Sun et al., 2016
BJ18B2	Zircon	53°17'22.1"	123°38'57"	872	—	—	78.60	10.90	Sun et al., 2016
BJ18B6	Zircon	53°20'53"	123°47'24.4"	705	—	—	116.70	15.70	Sun et al., 2016
BJ18B8	Zircon	53°24'07.9"	123°58'49.2"	398	—	—	122.20	11.90	Sun et al., 2016
BJ18C1	Zircon	53°05'29.3"	124°11'40.3"	742	—	—	93.40	8.30	Sun et al., 2016
BJ18C3	Zircon	53°01'25.8"	124°08'44.3"	772	—	—	84.00	8.50	Sun et al., 2016
BJ19A9A	Zircon	52°44'39"	124°30'00.9"	852	—	—	83.90	14.80	Sun et al., 2016
BJ20A1	Zircon	52°32'57"	125°16'55.6"	575	—	—	99.80	12.30	Sun et al., 2016
BJ20A4	Zircon	52°57'45.9"	125°17'49.5"	594	—	—	106.50	16.50	Sun et al., 2016
BJ21A3	Zircon	52°21'40.2"	124°48'03.5"	785	—	—	135.00	15.50	Sun et al., 2016
BJ15A9	Zircon	52°44'52.9"	121°58'58.9"	648	—	—	64.30	9.60	Sun et al., 2016
BJ15B2	Zircon	52°41'42.3"	121°54'26.3"	865	—	—	93.70	10.10	Sun et al., 2016
BJ15B5	Zircon	52°38'14.2"	121°45'28.3"	743	—	—	108.20	17.70	Sun et al., 2016
BJ15B6	Zircon	52°35'55.1"	121°39'54.1"	665	—	—	73.20	14.10	Sun et al., 2016
Zk3-1-19	Zircon	37°24'25.92"	96°06'27.79"	3300	—	sandstone	214.00	16.00	Li ZX et al., 2015
Zk3-1-81	Zircon	37°24'25.92"	96°06'27.79"	3095	Upper Carboniferous	sandstone	155.00	8.10	Li ZX et al., 2015

DMY15801	Zircon	38°23'09.94"	90°07'19.92"	3237	Upper Ordovician	andesite basalt	177.00	9.80	Li ZX et al., 2015
DMY15201	Zircon	38°22'59.86"	90°07'28.08"	3213	Upper Ordovician	altered basalt	98.00	4.40	Li ZX et al., 2015
C120401	Zircon	38°15'10.22"	94°34'09.33"	3711	Upper Ordovician	andesite basalt	162.00	12.00	Li ZX et al., 2015
C1203-10	Zircon	38°14'41.46"	94°34'11.94"	3808	Upper Ordovician	phyllite	156.00	11.00	Li ZX et al., 2015
C1203-18	Zircon	38°14'43.68"	94°34'13.35"	3763	—	gabbro	145.00	8.00	Li ZX et al., 2015
C1203-58	Zircon	38°13'33.18"	94°34'08.22"	3706	—	andesite basalt	184.00	11.00	Li ZX et al., 2015
DXS17001	Zircon	38°53'13.00"	93°26'48.07"	2825	Upper Ordovician	diorite	143.00	6.20	Li ZX et al., 2015
Zk3-1-74	Zircon	37°24'25.92"	96°06'27.79"	3110	—	sandstone	195.00	9.80	Li ZX et al., 2015
Zk3-2-82	Zircon	37°24'08.85"	96°06'24.77"	3505	—	sandstone	188.00	15.00	Li ZX et al., 2015
C1203-02	Zircon	38°15'03.33"	94°34'15.25"	3813	Upper Ordovician	phyllite	193.00	14.00	Li ZX et al., 2015
ln-8	Zircon	33°55.309'	110°15.525"	1054	Upper Triassic	sandstone	89.00	4.80	Huang et al., 2016
ln-12	Zircon	33°55.025'	110°15.16"	1043	Upper Triassic	sandstone	106.90	5.90	Huang et al., 2016
TS-1	Zircon	32°03'48.6"	119°00'21.5"	—	Middle Silurian	sandstone	243.00	16.00	Yuan et al., 2016
LS-4	Zircon	32°04'05.0"	119°15'12.5"	—	Upper Devonian	sandstone	203.00	9.00	Yuan et al., 2016
MS-1	Zircon	31°47'06.1"	119°18'46.5"	—	Middle Silurian	sandstone	227.00	11.00	Yuan et al., 2016
MS-2	Zircon	31°47'25.0"	119°18'46.2"	—	Middle Silurian	sandstone	253.00	12.00	Yuan et al., 2016
FS-1	Zircon	31°29'58.2"	119°10'14.7"	—	Upper Devonian	sandstone	217.00	11.00	Yuan et al., 2016
FS-2	Zircon	31°31'18.1"	119°11'21.6"	—	Middle Silurian	sandstone	227.00	10.00	Yuan et al., 2016
GD-3	Zircon	30°52'14.4"	119°24'20.2"	—	Lower Cretaceous	sandstone	153.00	7.00	Yuan et al., 2016
GD-6	Zircon	30°48'04.0"	119°25'22.2"	—	Lower Silurian	sandstone	111.00	5.00	Yuan et al., 2016
WX-1	Zircon	31°31'01.3"	120°14'10.0"	—	Upper Devonian	sandstone	196.00	10.00	Yuan et al., 2016
WX-2	Zircon	31°35'28.2"	120°14'40.1"	—	Middle Silurian	sandstone	97.00	4.00	Yuan et al., 2016
430	Zircon	29°36'37.7"	120°21'03.0"	—	Upper Proterozoic	diorite	113.00	3.00	Wang F et al., 2015
431	Zircon	29°36'35.4"	120°21'05.5"	—	Upper Proterozoic	diorite	105.00	5.00	Wang F et al., 2015
432	Zircon	29°36'35.4"	120°21'05.5"	—	Upper Proterozoic	diorite	149.00	8.00	Wang F et al., 2015
43	Zircon	29°42'0.1"	120°25'11.6"	—	Upper Proterozoic	diorite	208.00	11.00	Wang F et al., 2015
44	Zircon	29°41'28.5"	120°25'06.4"	—	Upper Proterozoic	diorite	121.00	6.00	Wang F et al., 2015
434	Zircon	29°35'10.8"	120°21'57.9"	—	Upper Proterozoic	gneiss	131.00	7.00	Wang F et al., 2015
435	Zircon	29°34'48.4"	120°22'57.5"	—	Upper Proterozoic	gneiss	134.00	6.00	Wang F et al., 2015
436	Zircon	29°34'47.7"	120°23'24.6"	—	Upper Proterozoic	gneiss	133.00	7.00	Wang F et al., 2015

437	Zircon	29°34'43.6"	120°23'33.2"	—	Upper Proterozoic	gneiss	103.00	5.00	Wang F et al., 2015
438	Zircon	29°34'44.4"	120°23'52.1"	—	Upper Proterozoic	gneiss	123.00	6.00	Wang F et al., 2015
433	Zircon	29°35'30.4"	120°21'07.9"	—	Upper Proterozoic	mylonite	299.00	16.00	Wang F et al., 2015
42	Zircon	29°41'26.0"	120°27'13.5"	—	Upper Proterozoic	mylonite	199.00	6.00	Wang F et al., 2015
45	Zircon	29°41'31.8"	120°26'41.5"	—	Upper Proterozoic	mylonite	111.00	8.00	Wang F et al., 2015
46	Zircon	29°41'24.4"	120°26'21.1"	—	Upper Proterozoic	mylonite	130.00	7.00	Wang F et al., 2015
47	Zircon	29°41'23.2"	120°25'46.4"	—	Upper Proterozoic	mylonite	168.00	8.00	Wang F et al., 2015
Nx1	Zircon	25°12'45"	114°14'59"	188	Jurassic	granite	87.30	4.00	Yan Yi et al., 2009
Nx2	Zircon	25°13'23"	114°14'16"	208	Jurassic	granite	92.80	4.90	Yan Yi et al., 2009
Fg1	Zircon	23°58'30"	113°33'16"	263	Jurassic	granite	109.90	5.40	Yan Yi et al., 2009
Fg2	Zircon	23°57'39"	113°36'20"	131	Jurassic	granite	116.60	4.70	Yan Yi et al., 2009
Fg3	Zircon	23°57'39"	113°36'22"	131	Jurassic	granite	97.40	3.60	Yan Yi et al., 2009
Hy4	Zircon	23°46'36"	114°45'75"	260	Jurassic	granite	108.10	4.60	Yan Yi et al., 2009
Hy5	Zircon	23°46'36"	114°45'75"	260	Jurassic	granodiorite	105.50	5.40	Yan Yi et al., 2009
Hy7	Zircon	23°46'32"	114°38'53"	445	Jurassic	granite	95.60	4.20	Yan Yi et al., 2009
Hy8	Zircon	23°46'48"	114°39'11"	550	Jurassic	granite	96.10	4.00	Yan Yi et al., 2009
Hy9	Zircon	23°46'48"	114°39'11"	550	Jurassic	granodiorite	111.90	4.20	Yan Yi et al., 2009
Hz1	Zircon	22°49'52"	114°57'28"	109	Jurassic	granite	97.40	4.30	Yan Yi et al., 2009
Hz4	Zircon	22°50'16"	115°01'74"	143	Jurassic	granite	98.80	5.60	Yan Yi et al., 2009
Hz6	Zircon	23°16'38"	114°01'47"	1120	Jurassic	granite	112.70	4.80	Yan Yi et al., 2009
Hz7	Zircon	23°16'36"	114°01'53"	984	Jurassic	granite	106.50	4.50	Yan Yi et al., 2009
Hz8	Zircon	23°15'12"	114°02'23"	212	Jurassic	granite	98.20	3.90	Yan Yi et al., 2009
Hz9	Zircon	23°14'02"	114°01'08"	250	Jurassic	granite	94.80	4.10	Yan Yi et al., 2009
MS-3	Zicron	35°32'46"	117°56'15"	883	Archaeozoic	granite	297.00	28.00	Tang et al., 2011
S42	Zicron	24°29'31"	100°17'42"	1034	Triassic-Jurassic	granite	50.30	4.40	Shi et al., 2006
S51	Zicron	23°49'59"	100°03'36"	1650	Triassic-Jurassic	granite	56.90	5.70	Shi et al., 2006
S53	Zicron	23°35'42"	100°01'55"	1981	Triassic-Jurassic	granite	60.70	5.00	Shi et al., 2006
S57	Zicron	23°57'50"	100°05'56"	1446	Triassic-Jurassic	granite	52.90	5.10	Shi et al., 2006
S61	Zicron	23°48'14"	100°14'56"	1462	Triassic-Jurassic	granite	69.80	6.70	Shi et al., 2006
S62	Zicron	22°01'52"	100°31'30"	1487	Triassic-Jurassic	granite	62.00	5.40	Shi et al., 2006

T101-2	Zircon	28°18.08'	85°20.90'	2000	Pre-Sinian	plagiogranite	2.7	0.4	Li et al., 2013
T101-3	Zircon	28°21.83'	85°21.06'	2500	Pre-Sinian	plagiogranite	5.8	1	Li et al., 2013
T101-5	Zircon	28°25.10'	85°13.87'	3100	Pre-Sinian	granite	8.7	0.8	Li et al., 2013
T101-8	Zircon	28°32.76'	85°13.87'	3539	—	metasandstone	13.1	2.2	Li et al., 2013
HS01	Zircon	30°08'36.4"	118°10'12.3"	1656	Jurassic-Lower Createceous	granite	75	5	Zheng et al., 2011
HS05	Zircon	30°08'07.8"	118°09'38.5"	1780	Jurassic-Lower Createceous	granite	81	9	Zheng et al., 2011
HS09	Zircon	30°07'37.9"	118°09'49.7"	1666	Jurassic-Lower Createceous	granite	85	6	Zheng et al., 2011
HS16	Zircon	30°07'05.8"	118°10'14.6"	760	Jurassic-Lower Createceous	granite	77	6	Zheng et al., 2011
HS18	Zircon	30°06'54.3"	118°10'03.3"	693	Jurassic-Lower Createceous	granite	88	10	Zheng et al., 2011
HS19	Zircon	30°06'25.5"	118°09'45.5"	1099	Jurassic-Lower Createceous	granite	86	10	Zheng et al., 2011
HS20	Zircon	30°06'15.2"	118°10'01.07"	897	Jurassic-Lower Createceous	granite	69	6	Zheng et al., 2011
HS23	Zircon	30°11'57.9"	118°49'18.6"	936	Jurassic-Lower Createceous	granite	83	8	Zheng et al., 2011
HS24	Zircon	30°12'14.6"	118°48'46.8"	733	Jurassic-Lower Createceous	granite	67	11	Zheng et al., 2011
HS25	Zircon	30°13'12.6"	118°48'11.9"	415	Jurassic-Lower Createceous	granite	61	6	Zheng et al., 2011
I-03	Zircon	25°44'12.3"	113°09'47.9"	212	Jurassic-Lower Createceous	granite	159	8	Wan, 2013
I-05	Zircon	25°44'53.5"	113°09'57.0"	308	Jurassic-Lower Createceous	granite	151	7	Wan, 2013
I-08	Zircon	25°44'56.7"	113°09'55.6"	389	Jurassic-Lower Createceous	granite	165	10	Wan, 2013
SY11-Zr	Zircon	18°52'36"	109°38'31"	614	Permian	granite	100.9	4.1	Yan et al., 2011
SY12-Zr	Zircon	18°55'54"	109°27'15"	239	Permian	granodiorite	79.8	3.9	Yan et al., 2011
SY13-Zr	Zircon	18°55'36"	109°28'30"	203	Permian	diorite	102.3	4.3	Yan et al., 2011
SY14-Zr	Zircon	18°52'29"	109°30'39"	431	Permian	diorite	107	5.5	Yan et al., 2011
SY15-Zr	Zircon	18°53'44"	109°30'41"	329	Permian	diorite	107.4	4.4	Yan et al., 2011
SY17-Zr	Zircon	19°02'30"	109°48'08"	250	Permian	granodiorite	100.4	5.1	Yan et al., 2011
DF2-Zr	Zircon	18°14'38"	109°18'31"	164	Cretaceous	granite	56.2	3.2	Yan et al., 2011
Mc-8a	Zircon	32°35.369'	106°50.674'	1269	Proterozoic	diorite	169	—	Sun, 2011
Mc-17	Zircon	32°28.313'	106°26.733'	625	Proterozoic	vein	210	—	Sun, 2011
L04-03U1	Zircon	29.44°	89.63°	3950	Palaeocene	granite	53.9	2.8	Ge, 2016
L05-08U1	Zircon	29.61°	89.98°	4294	Eocene	granite	17.5	0.8	Ge, 2016
L05-11U1	Zircon	29.56°	90.01°	4229	Upper Cretaceous	granite	18.6	0.9	Ge, 2016
L06-01U1	Zircon	29.27°	90.25°	4170	Lower Cretaceous	granite	54.6	2.2	Ge, 2016

L06-07U1	Zircon	29.39°	90.17°	3776	Lower Cretaceous	granite	25.5	1.1	Ge, 2016
L07-14U1	Zircon	29.52°	90.92°	3646	Lower Cretaceous	granite	18.1	0.8	Ge, 2016
L10-14U1	Zircon	30.00°	91.93°	3946	Lower Cretaceous	granite	126.7	5.8	Ge, 2016
L13-04U1	Zircon	29.50°	91.80°	4219	Lower Cretaceous	granite	24.1	1.6	Ge, 2016
L13-08U1	Zircon	29.32°	91.84°	3699	Lower Cretaceous	granite	25.9	1.8	Ge, 2016
QW13-U1	Zircon	29.39°	87.421°	4366	Oligocene-Miocene	sandstone	72.6	4.6	Ge, 2016
QW13-U2	Zircon	29.39°	87.4205°	4361	Oligocene-Miocene	sandstone	55.8	2.9	Ge, 2016
QW13-U3	Zircon	29.38°	87.42°	4258	Oligocene-Miocene	sandstone	70.9	4.8	Ge, 2016
X12-21	Zircon	29.43°	88.26°	4024	Eocene	granodiorite	36.1	1.8	Ge, 2016
X12-33	Zircon	29.46°	88.23°	3982	Eocene	granite	66.3	2.6	Ge, 2016
GAOJP213z	Zircon	38.898449.9°	102°07'39.7"	1664	Middle Proterozoic	migmatitic granite	92	2	Tian et al., 2016
1	Zircon	30.04°	101.54167°	3480	—	sandstone	162.6	10.6	Xu et al., 2000
2	Zircon	30.0783333°	101.8044°	4290	—	granite	7.1	0.6	Xu et al., 2000
3	Zircon	29.9933333°	101.89667°	3200	—	granite	5.4	0.6	Xu et al., 2000
4	Zircon	29.9938889°	101.94°	3112	—	granite	4.7	0.4	Xu et al., 2000
5	Zircon	29.995°	101.94167°	3062	—	granite	5.4	0.8	Xu et al., 2000
6	Zircon	29.9958333°	101.94333°	3004	—	granite	3.7	0.3	Xu et al., 2000
7	Zircon	29.9966667°	101.945°	2983	—	granite	3	0.2	Xu et al., 2000
8	Zircon	30°	101.94667°	2983	—	granite	4	0.2	Xu et al., 2000
9	Zircon	30.005°	101.94833°	2870	—	granite	7.3	0.4	Xu et al., 2000
10	Zircon	30.0016667°	101.94833°	2868	—	granite	2.8	0.2	Xu et al., 2000
11	Zircon	30.01°	101.95°	2806	—	granite	5.3	0.3	Xu et al., 2000
12	Zircon	30.0133333°	101.95°	2795	—	granite	3.2	0.3	Xu et al., 2000
13	Zircon	30.0583333°	101.97833°	2503	Proterozoic	—	25.1	1.2	Xu et al., 2000
14	Zircon	30.0583333°	101.98167	2485	Proterozoic	—	19.9	1.4	Xu et al., 2000
15	Zircon	30.06°	102°	2450	Proterozoic	—	21	1.4	Xu et al., 2000
16	Zircon	30.06°	102.025°	2330	Proterozoic	—	22.4	2.1	Xu et al., 2000
17	Zircon	30.06°	102.03333°	2260	Proterozoic	—	18.6	1.4	Xu et al., 2000
18	Zircon	30.0616667°	102.03333°	2257	Proterozoic	—	16.8	1.5	Xu et al., 2000
19	Zircon	30.06167°	102.05°	2182	Proterozoic	—	18.2	0.8	Xu et al., 2000

20	Zircon	30.06167°	102.075°	2030	Proterozoic	—	20.5	1.5	Xu et al., 2000
21	Zircon	30.06333°	102.075°	2020	Proterozoic	—	17.4	1.6	Xu et al., 2000
22	Zircon	29.7°	102.01333°	2345	Proterozoic	—	4.2	0.3	Xu et al., 2000
23	Zircon	29.7°	102.015°	2340	—	granite	2	0.1	Xu et al., 2000
24	Zircon	29.70833°	102.01583°	2320	—	granite	1.9	0.1	Xu et al., 2000
25	Zircon	29.71667°	102.01667°	2288	—	granite	2.4	0.3	Xu et al., 2000
26	Zircon	29.725°	102.025°	2280	—	granite	2.3	0.2	Xu et al., 2000
27	Zircon	29.73333°	102.03333°	2280	—	granite	3	0.1	Xu et al., 2000
28	Zircon	29.7°	102.03833°	2110	—	granite	2.2	0.2	Xu et al., 2000
30	Zircon	29.7°	102.04167°	2110	—	granite	2	0.2	Xu et al., 2000
31	Zircon	29.695°	102.03528°	2120	Permian-Triassic	—	14	3	Xu et al., 2000
32	Zircon	29.695°	102.04667°	2120	Permian-Triassic	—	19.4	5.1	Xu et al., 2000
34	Zircon	29.63333°	102.14167°	1380	Proterozoic	—	10.7	2.8	Xu et al., 2000
35	Zircon	29.625°	102.14333°	1380	Proterozoic	—	23.7	7.8	Xu et al., 2000
36	Zircon	29.62°	102.14333°	1365	Proterozoic	—	35.1	8.7	Xu et al., 2000
37	Zircon	29.61667°	102.14333°	1382	Proterozoic	—	29.8	1.5	Xu et al., 2000
38	Zircon	29.53333°	102.14333°	1210	Proterozoic	—	30.4	26.2	Xu et al., 2000
39	Zircon	29.53333°	102.145°	1154	Proterozoic	—	26.9	2.2	Xu et al., 2000
40	Zircon	29.53333°	102.15°	1125	Proterozoic	—	32.8	4.7	Xu et al., 2000
41	Zircon	29.53333°	102.15833°	1084	Proterozoic	—	44.2	2.4	Xu et al., 2000
42	Zircon	29.53333°	102.16167°	1070	Proterozoic	—	53.4	3.6	Xu et al., 2000
43	Zircon	29.53333°	102.16667°	1082	Proterozoic	—	37.2	1.6	Xu et al., 2000
47	Zircon	29.48333°	102.18°	1002	Proterozoic	—	58.1	11.1	Xu et al., 2000
48	Zircon	30.25667°	101.505°	3830	—	granite	108.5	13	Xu et al., 2000
49	Zircon	30.25667°	101.505°	3825	—	granite	130.1	4.3	Xu et al., 2000
50	Zircon	30.25667°	101.505°	3805	—	granite	126.1	5.2	Xu et al., 2000
51	Zircon	30.255°	101.50667°	3785	—	granite	132	4.7	Xu et al., 2000
52	Zircon	30.25°	101.50667°	3730	—	granite	131.6	3.4	Xu et al., 2000
53	Zircon	30.24583°	101.50833°	3585	—	granite	129.4	4.5	Xu et al., 2000
54	Zircon	30.245°	101.50833°	3590	—	granite	120	4.5	Xu et al., 2000

55	Zircon	30.28333°	101.53333°	3760	—	granite	122.3	4.4	Xu et al., 2000
56	Zircon	30.25333°	101.51667°	3560	—	granite	114.3	3.2	Xu et al., 2000
57	Zircon	30.54833°	101.61667°	3480	—	mylonite	8.7	0.6	Xu et al., 2000
58	Zircon	30.56667°	101.64333°	3280	—	granite	7.9	0.5	Xu et al., 2000
59	Zircon	30.57833°	101.65°	3000	—	granite	7.8	0.5	Xu et al., 2000
60	Zircon	31.54667°	100.37667°	3430	—	sandstone	181	13.1	Xu et al., 2000
61	Zircon	31.55167°	100.38167°	3420	—	sandstone	210.9	17	Xu et al., 2000
62	Zircon	31.56167°	100.38333°	3400	—	sandstone	230.8	24.5	Xu et al., 2000
64	Zircon	31.565°	100.39333°	3360	—	sandstone	239.6	18.9	Xu et al., 2000
65	Zircon	31.56667°	100.39667°	3350	—	sandstone	274.2	14.2	Xu et al., 2000
66	Zircon	31.43333°	100.59333°	3240	—	sandstone	275.1	20.9	Xu et al., 2000
67	Zircon	31.41667°	100.61667°	3235	—	sandstone	262.9	22.8	Xu et al., 2000
68	Zircon	31.44167°	100.68°	3200	—	sandstone	171	17	Xu et al., 2000
69	Zircon	31.45°	100.68333°	3200	—	sandstone	150.5	4.5	Xu et al., 2000
70	Zircon	31.48333°	100.69667°	3210	—	sandstone	142.8	5.2	Xu et al., 2000
71	Zircon	31.345°	100.655°	3660	—	granite	126	5	Xu et al., 2000
72	Zircon	31.34667°	100.65833°	3645	—	granite	134.8	2.3	Xu et al., 2000
73	Zircon	31.34833°	100.66°	3630	—	granite	130.1	3.8	Xu et al., 2000
74	Zircon	31.36667°	100.66667°	3520	—	granite	131.8	5.4	Xu et al., 2000
75	Zircon	31.37167°	100.66333°	3500	—	granite	134.7	4.6	Xu et al., 2000
76	Zircon	31.375°	100.665°	3400	—	granite	133.3	2.5	Xu et al., 2000
77	Zircon	31.37667°	100.66667°	3440	—	granite	121.8	3.6	Xu et al., 2000
79	Zircon	30.76667°	101.03°	2830	—	sandstone	163.7	17	Xu et al., 2000
82	Zircon	30.95833°	101.12833°	2930	—	sandstone	135.7	13	Xu et al., 2000
83	Zircon	30.83333°	101.26333°	3525	—	sandstone	265.7	17.1	Xu et al., 2000
88	Zircon	30.99667°	101.11°	2980	—	sandstone	140.2	17.5	Xu et al., 2000
89	Zircon	30.99333°	101.11333°	2990	—	sandstone	135.2	16.9	Xu et al., 2000
90	Zircon	30.995°	101.11667°	3000	—	sandstone	130.7	8.3	Xu et al., 2000
91	Zircon	31°	101.11667°	3100	—	sandstone	134.1	9	Xu et al., 2000
92	Zircon	31.01°	101.16°	3640	—	sandstone	151.3	6.5	Xu et al., 2000

93	Zircon	31.02333°	101.235°	4540	—	sandstone	150.2	8.1	Xu et al., 2000
94	Zircon	31.02667°	101.23833°	4560	—	sandstone	167.6	14.7	Xu et al., 2000
95	Zircon	31.00833°	101.00833°	3920	—	granite	138.3	4.3	Xu et al., 2000
96	Zircon	31.00333°	101.00583°	4040	—	granite	132.1	14.2	Xu et al., 2000
97	Zircon	30.825°	101.25°	3730	—	granite	103.9	3	Xu et al., 2000
98	Zircon	30.82583°	101.25083°	3730	—	granite	105.1	2.9	Xu et al., 2000
99	Zircon	30.82833°	101.25417°	3720	—	granite	107.6	3.8	Xu et al., 2000
100	Zircon	30.83111°	101.25722°	3665	—	granite	107.5	3.7	Xu et al., 2000
101	Zircon	30.83333°	101.2575°	3660	—	granite	98.8	11.9	Xu et al., 2000
102	Zircon	30.075°	101.94167°	2915	—	sandstone	5.7	0.4	Xu et al., 2000
105	Zircon	29.92833°	102.225°	1368	Proterozoic	—	74	12.3	Xu et al., 2000
107	Zircon	29.91667°	102.225°	1346	—	mylonite	12.9	1.1	Xu et al., 2000
108	Zircon	29.91333°	102.225°	1351	—	mylonite	10.8	0.9	Xu et al., 2000
109	Zircon	29.92833°	102.22167°	1392	Proterozoic	—	80.6	7.8	Xu et al., 2000
113	Zircon	29.84°	102.24167°	2608	Proterozoic	—	263.4	18.8	Xu et al., 2000
114	Zircon	31.15333°	101.90333°	2240	—	granite	23.7	1.3	Xu et al., 2000
115	Zircon	30.59667°	102.67111°	2880	—	sandstone	11.9	1.3	Xu et al., 2000
116	Zircon	31.02833°	102.24167°	2800	—	sandstone	202.1	33.9	Xu et al., 2000
117	Zircon	30.98°	102.69333°	2720	—	sandstone	211.3	16.5	Xu et al., 2000
118	Zircon	30.96167°	102.8825°	3800	—	sandstone	196.2	9	Xu et al., 2000
119	Zircon	30.91333°	102.9°	4487	—	sandstone	213.6	14.5	Xu et al., 2000
