# Supplementary material for: Uplift and denudation in the continental area of China linked to climatic effects: evidence from apatite and zircon fission track data
Source: Sci Rep. 2018 Jun 22;8:9546. doi: 10.1038/s41598-018-27801-7 (PMC6015080; doi:10.1038/s41598-018-27801-7)
Supplement: Supplementary file 3 — Dataset 3 [file 41598_2018_27801_MOESM3_ESM.pdf]

Uplift and denudation in continental area of China linked to climatic effects: evidence from apatite and zircon fission track data

Nansheng Qiu[1,2,*], Shuai Liu[1,2]

1. State Key Laboratory of Petroleum Resources and Prospecting, China University of Petroleum, Beijing, 102249, China
2. College of Geosciences, China University of Petroleum, Beijing 102249, China

Supplement 3. AFT and ZFT Dataset References

Supplement 3. References

An, Y.F., Han, Z.J., Wan, J.L., 2008. Fission track dating of the Cenozoic uplift in Mabian area, southern Sichuan Province, China. Science China: Earth Sciences, 51:1238-1247 **(in Chinese)**.

Cao, K., Wang, G.C., Beek, P.V.D., Bernet, M., Zhang, K.X., 2013. Cenozoic thermo-tectonic evolution of the northeastern Pamir revealed by zircon and apatite fission-track thermochronology. Tectonophysics, 589: 17-32.

Chen, D.X., 2016. Mesozoic-Cenozoic tectonic evolution and low temperature thermochronological study of Eastern Heilongjiang, NE China. Ph.D. Dissertation, Zhejiang University, Hangzhou, China, 187pp **(in Chinese with English abstract)**.

Chen, H., Hu, J.M., Wu, G.L., Shi, W., Geng, Y.Y., Qu, H.J., 2015. Apatite fission-track thermochronological constraints on the pattern of late Mesozoic-Cenozoic uplift and exhumation of the Qinling Orogen, central China. Journal of Asian Earth Sciences, 114: 649-673.

Chen, Z.L., Gong, H.L., Li, L., Wang, X.F., Chen, B.L., Chen, X.H., 2006a. Cenozoic uplifting and exhumation process of the Altyn Tagh Mountains. Earth Science Frontiers, 2016, 13: 91-102 **(in Chinese with English abstract)**.

Chen, Z.L., Wan, J.L., Liu, J., Li, S.X., Zheng, E.J., Han, X.Z., Li, X.G., Gong, H.L., 2006b. Multi-stage uplift and exhumation of the west Tianshan Mountains: Evidence from the apatite fission-track dating. Acta Geoscientica Sinica, 27: 97-106 **(in Chinese with English abstract)**.

Chen, Z.L., Zhang, Y.Q., Wang, X.F., Chen, X.H., 2001. Fission track dating of apatite: Constrains on the Cenozoic uplift of the Altyn Tagh Mountain. Acta Geoscientia Sinica, 22: 413-418 **(in Chinese with English abstract)**.

Deng, B., Liu, S.G., Li, Z.W., Jansa, L.F., Liu, S., Wang, G.Z., Sun, W., 2013a. Differential exhumation at eastern margin of the Tibetan Plateau, from apatite fission-track thermochronology. Tectonophysics, 591: 98-115.

Deng, B., Liu, S.G., Liu, S., Li, X.H., Li, J.C., Li, Z.W., 2013b. Tectno-thermal events and chronological framework in Zoige and its periphery. Earth Science-Journal of China University of Geosciences, 38: 317-328 **(in Chinese with English abstract)**.

Deng, B., Liu, S.G., Liu, S., Li, Z.W., Zhao, J.C., 2009. Restoration of exhumation thickness and its significance in Sichuan Basin, China. Journal of Chengdu University of Technology (Science & Technology Edition), 36, 675-686 **(in Chinese with English abstract)**.

Deng, B., Yong, Z.Q., Liu, S.G., Li, Z.W., Zhao, G.P., MISE, Z.H., Tang, C., 2016. Cenozoic mountain-building processes in the Daliangshan, southeastern margin of the Tibetan Plateau: Evidence form low-temperature thermochronology and thermal modeling. Chinese Journal of Geophysics, 59: 2162-2175 **(in Chinese with English abstract)**.

Du, Z.L., Wang, Q.C., 2007. Mesozoic and Cenozoic uplifting history of the Tianshan region: Insight from apatite fission track. Acta Geologica Sinica, 81: 1081-1101 **(in Chinese with English abstract)**.

Dumitru, T.A., Zhou, D., Chang, E.Z., Graham, S.A., Hendrix, M.S., Sobel, E.R., Carroll, A.R., 2001. Uplift, exhumation, and deformation in the Chinese Tian Shan. In: Hendrix, M.S., Davis, G.A. (Eds.), Paleozoic and Mesozoic tectonic evolution of central Asia: From continental assembly to intercontinental deformation. Geological Society of America Memoir 194, Colorado, pp.71-99.

Enkelmann, E., Ratschbacher, L., Jonckheere, R., Nestler, M., Gloaguen, R., Hacker, B.R., Zhang, Y.Q., Ma, Y.S., 2006. Cenozoic exhumation and deformation of northeastern Tibet and the Qinling: Is Tibetan lower crustal flow diverging around the Sichuan Basin? Geological Society of America Bulletin, 118: 651-671.

Gao, C., Liu, J., Li, D.W., Wang, F., Liu, D.M., 2014. The constraints of the fission track thermochronology on active time of the Southern Tibetan Detachment System in Cho Oyu, Tibet. Earth Science Frontiers, 21: 372-380 **(in Chinese with English abstract)**.

Ge, Y.K., 2016. The exhumation history of the Gangdese Batholith from low temperature thermochronology and sedimentary response. Ph.D. Dissertation, China University of Geosciences, Beijing, China, 100pp **(in Chinese with English abstract)**.

Gillespie, J., Glorie, S., Xiao, W.J., Zhang, Z.Y., Collins, A.S., Evans, N., McInnes, B., Grave, D.J., 2017. Mesozoic reactivation of the Beishan, southern Central Asian Orogenic Belt: Insights from low-temperature thermochronology. Gondwana Research, 43: 107-122.

Grimmer, J.C., Jonckheere, R., Enkelmann, E., Ratschbacher, L., Hacker, B.R., Blythe, A.E., Wagner, G.A., Wu, Q., Liu, S., Dong, S., 2002. Cretaceous-Cenozoic history of the southern Tan-Lu fault zone: apatite fission-track and structural constraints from the Dabie Shan (eastern China). Tectonophysics, 359: 225-253.

Guo, Z.J., Zhang, Z.C., Wu, C.D., Fang, S.H., Zhang, S., 2006. The Mesozoic and Cenozoic exhumation history of Tianshan and comparative studies to the Junggar and Altai Mountains. Acta Geologica Sinica, 80: 1-15 **(in Chinese with English abstract)**.

Han, W., Lu, J.C., Wei, J.S., Zhang, Y.P., Li, Y.H., Li, Y., 2015. Apatite fission track constraints on the Mesozoic tectonic activities in Shangdan Depression, Yin'e Basin, Inner Mongolia. Acta Geologica Sinica, 89: 2277-2285 **(in Chinese with English abstract)**.

Han, W., Lu, J.C., Zhang, Y.P., Li, Y.H., Wei, J.S., Liu, X., 2014. Apatite fission track and its petroleum geological significance of the Ejina area and its vicinity, western Inner Mongolia. Geotectonica et Metallogenia, 2014,

38: 647-655**(in Chinese with English abstract)**.
Hendrix, M.S., Dumitru, T.A., Gramham, S.A., 1994. Late Oligocene-early Miocene uproofing in the Chinese Tian Shan: An early effect of the India-Asia collision. Geology, 22: 487-490.
Hu, S.B., Kohn, B.P., Raza, A., Wang, J.Y., Gleadow, A.J.W., 2006a. Cretaceous and Cenozoic cooling history across the ultrahigh pressure Tongbai-Dabie belt, central China, from apatite fission-track thermochronology. Tectonophysics, 420: 409-429.
Hu, S.B., Raza, A., Min, K., Kohn, B.P., Reiners, P.W., Ketcham, R.A., Wang, J.Y. Gleadow, A.J.W., 2006b. Late Mesozoic and Cenozoic thermotectonic evolution along a transect from the north China craton through the Qinling orogen into the Yangtze craton, central China. Tectonics, 25, TC6009.
Hu, Y.Z., 2011. Sedimentary basin analysis and study of antimony and gold deposit mineralization in Southwestern Guizhou Depression. Ph.D. Dissertation, Kunming University of Science and Technology, Kunming, China, 171pp **(in Chinese with English abstract)**.
Huang, Z.G., Ren, Z.L., Gao, L.G., 2016. Evidence from detrital zircon and apatite fission track for tectonic evolution since Cretaceous in southeastern margin of Ordos Basin. Chinese Journal of Geophysics, 59: 3753-3764 **(in Chinese with English abstract)**.
Jia, Y.Y., Fu, B.H., Jolivet, M., Zheng, S., 2015. Cenozoic tectono-geomorphological growth of the SW Chinese Tian Shan: Insight from AFT and detrital zircon U-Pb data. Journal of Asian Earth Sciences, 111: 395-413.
Jin, W., 2007. Research on sedimentary response to uplifting of Tibetan Plateau hinterland and oil-gas reservation during the late Cretaceous-Paleogene period. Ph.D. Dissertation, Chengdu University of Technology, Chengdu, China, 169pp **(in Chinese with English abstract).**
Jolivet, M., Brunel, M., Seward, D., Xu, Z., Yang, J., Malavieille, J., Roger, F., Leyreloup, A., Arnaud, N., Wu, C., 2003. Neogene extension and volcanism in the Kunlun Fault Zone, northern Tibet: New constraints on the age of the Kunlun Fault. Tectonics, 22: 1052.
Jolivet, M., Roger, F., Xu, Z.Q., Paquette, J.L., Cao, H., 2015. Mesozoic-Cenozoic evolution of the Danba dome (Songpan Garzê, East Tibet) as inferred from LA-ICPMS U-Pb and fission-track data. Journal of Asian Earth Sciences, 102: 180-204.
Kang, W.J., Xu, X.W., Yu, G.H., Tan, X.B., Li, K., 2016. Thermochronological evidence for division of Quaternary uplifting stages of Mt. Namjagbarwa. Chinese Journal of Geophysics, 59: 1753-1761 **(in Chinese with English abstract)**.
Lai, Q.C., Ding, L., Wang, H.W., Qiu, Y.H., Cai, F.L., 2006. Extension process of eastern margin of the Tibetan Plateau: Constraints from apatite fission track thermal history. Science China: Earth Sciences, 36: 785-796 **(in Chinese)**.
Lei, Y.L., Gong, D.H., Wang, X.M., Zhong, D.L., Wang X.S., Zhang, J., 2008a. Discussion about using different modes of apatite fission track thermochronology to constrain cooling histories of rocks: An example from the batholith in Dulong River region, west Yunnan. Progress in Geophysics, 23: 422-432 **(in Chinese with English abstract)**.
Lei, Y.L., Zhong, D.L., Ji, J.Q., Jia, C.Z., Zhang, J., 2008b. Fission track evidence for two Pleistocene uplift-exhumation events in the eastern Himalayan syntaxis. Quaternary Science, 28: 584-590 **(in Chinese with English abstract)**.
Lei, Y.L., Zhong, D.L., Jia, C.Z., Ji, J.Q., Zhang, J., 2008c. Late Cenozoic differential uplift-exhumation of batholith and propagation of uplift recorded by fission track thermochronology in Chayu area, the southeast margin of the Tibetan Plateau. Acta Petrologica Sinica, 24: 384-394 **(in Chinese with English abstract)**.
Li, B.L., Ji, J.Q., Luo, Q.H., Gong, J.F., Qing, J.C., 2012. The structural style and timing of uplift of the Ailaoshan-Diancang Range, west Yunnan, China. Seismology and Geology, 34: 696-712 **(in Chinese with English abstract)**.
Li, J.F., Tang, W.H., Liu, Z., Zhang, Z.C., 2010. Apatite fission track analysis of Upper Jurassic Houcheng Formation at Qianjiandian area, Beijing and its geological significance. Chinese Journal of Geophysics, 53: 2907-2917 **(in Chinese with English abstract)**.
Li, J.H., Shi, W., Zhang, Y.Q., Dong, S.W., Ma, Z.L., 2016. Thermal evolution of the Hengshan extensional dome in central South China and its tectonic implications: New insights into low-angle detachment formation. Gondwana Research, 35: 425-441.
Li, J.X., Liu, C.Y., Yue, L.P., Wang, J.Q., 2015. Apatite fission track evidence for the Cenozoic uplift to the Lüliang Mountains and a discussion on the uplift mechanism. Geology in China, 42: 960-972 **(in Chinese with English abstract)**.
Li, K., Jolivet, M., Zhang, Z.C., Li, J.F., Tang, W.H., 2016. Long-term exhumation history of the Inner Mongolian Plateau constrained by apatite fission track analysis. Tectonophysics, 666: 121-133.
Li, L., Zhong, D.L., 2006. Fission track evidence of Cenozoic uplifting events of the Taishan, China. Acta Petrologica Sinica, 22: 457-464 **(in Chinese with English abstract)**.
Li, M., Wang, A., Liu, C., Wang, G.C., Li, T., Garver, J.I., 2013. Neogene exhumation of the Greater Himalaya Slab in Gyirong area, Tibet, constrained by fission track geochronology. Geological Bulletin of China, 32: 86-92 **(in Chinese with English abstract)**.
Li, S.J., Li, J.M., Zhou, Y., Wo, Y.J., Wang, X.W., 2011. Fission track evidence for Mesozoic-Cenozoic uplifting in the southeastern margin of Sichuan Basin. Acta Petrologica et Mineralogica, 30: 225-233 **(in Chinese with**

English abstract).
Li, W., Hu, J.M., Qu, H.J., 2010. Fission track analysis of Junggar Basin peripheral orogen and its geological significance. Acta Geologica Sinica, 84: 171-182 **(in Chinese with English abstract)**.
Li, X.M., Yang, X.Y., Xia, B., Gong, G.L., Shan, Y.H., Zeng, Q.S., Li, W., Sun, W.D., 2011. Exhumation of the Dahinggan Mountains, NE China from the Late Mesozoic to the Cenozoic: New evidence from fission-track thermochronology. Journal of Asian Earth Sciences, 42: 123-133.
Li, Y., Ji, J.Q., Tu, J.Y., Gong, J.F., Wang, Z.L., Wang, J.D., 2009. Structure of Liujiang terrain and implications for displacement of Tanlu fault system. Acta Petrologica Sinica, 25: 675-681 **(in Chinese with English abstract)**.
Li, Z.W., Chen, H.D., Liu, S.G., Hou, M.C., Deng, B., 2010. Differential uplift driven by thrusting and its lateral variation along the Longmen Shan belt, western Sichuan, China: Evidence from fission track thermochronology. Chinese Journal of Geology, 45, 944-968 **(in Chinese with English abstract)**.
Li, Z.X., Gao, J., Zheng, C., Liu, C.L., Ma, Y.S., Zhao, W.Y., 2015. Present-day heat flow tectonic-thermal evolution since the late Paleozoic time of the Qaidam Basin. Chinese Journal of Geophysics, 58: 3687-3705 **(in Chinese with English abstract)**.
Liu, D.L., Li, H.B., Sun, Z.M., Pan, J.W., Wang, M., Wang, H., Chevalier, M.L., 2017. AFT dating constrains the Cenozoic uplift of the Qimen Tagh Mountains, Northeast Tibetan Plateau, comparison with LA-ICPMS Zircon U-Pb ages. Gondwana Research, 41: 438-450.
Liu, J.H., Zhang, P.Z., Lease, R.O., Zheng, D.W., Wan, J.L., Wang, W.T., Zhang, H.P., 2013. Eocene onset and late Miocene acceleration of Cenozoic intracontinental extension in the North Qinling range-Weihe graben: Insights from apatite fission track thermochronology. Tectonophysics, 584: 281-296.
Liu, J.H., Zhang, P.Z., Zheng, D.W., Wan, J.L., Wang, W.T., Du, P., Lei, Q.Y., 2010. Pattern and timing of late Cenozoic rapid exhumation and uplift of the Helan Mountain, China. Science China: Earth Sciences, 53: 345-355.
Liu, S.G., Ma, Y.S., Sun, W., Cai, X.Y., Liu, S., Hang, W.M., Xu, G.S., Yong, Z.Q., Wang, G.Z., Wang, H., Pan, C.L., 2008. Studying on the difference of Sinian natural gas pools between Weiyuan Gas Field and Ziyang Gas-Brone area, Sichuan basin. Acta Geologica Sinica, 82, 328-337 **(in Chinese with English abstract)**.
Liu, Z.J., Wang, J.P., Zheng, D.W., Liu, J.J., Liu, J., Fu, C., 2010. Exploration prospect and post-ore denudation in the northwestern Jiaodong Gold Province , China: Evidence from apatite fission track thermochronology. Acta Petrologica Sinica, 26: 3597-3611 **(in Chinese with English abstract)**.
Lü, H.H., Chang, Y., Wang, W., Zhou, Z.Y., 2013. Rapid exhumation of the Tianshan Mountains since the early Miocene: Evidence from combined apatite fission track and (U-Th)/He thermochronology. Science China: Earth Sciences, 43: 1964-1974 **(in Chinese)**.
Lu, H.J., Wang, E., Shi, X.H., Meng, K., 2012. Cenozoic tectonic evolution of the Elashan range and its surroundings, northern Tibetan Plateau as constrained by paleomagnetism and apatite fission track analyses. Tectonophysics, 580: 150-161.
Qi, B.S., Hu, D.G., Yang, X.X., Zhang, Y.L., Tan, C.X., Zhang, P., Feng, C.J., 2016. Apatite fission track evidence for the Cretaceous-Cenozoic cooling history of the Qilian Shan (NW China) and for stepwise northeastward growth of the northeastern Tibetan Plateau since early Eocene. Journal of Asian Earth Sciences, 124: 28-41.
Reid, A.J., Fowler, A.P., Phillips, D., Wilson, C.J.L., 2005. Thermochronology of the Yidun Arc, central eastern Tibetan Plateau: constraints from $^{40}Ar/^{39}Ar$ K-feldspar and apatite fission track data. Journal of Asian Earth Sciences, 25: 915-935.
Ren, X.M., Zhu, W.B., Zhu, X.Q., Wang, X., Luo, M., 2015. Mesozoic-Cenozoic uplift-exhumation history of Lüliangshan Area of Shanxi: Evidence from apatite fission track. Journal of Earth Sciences and Environment, 37: 63-73 **(in Chinese with English abstract)**.
Richardon, N.J., Densmore, A.L., Seward, D., Fowler, A., Wipf, M. Ellis, M.A., Li, Y., Zhang, Y., 2008. Extraordinary denudation in the Sichuan Basin: Insights from low-temperature thermochronology adjacent to eastern margin of the Tibetan Plateau. Journal of Geophysical Research, 113, B04409.
Shen, C.B., Mei, L.F., Liu, L., Tang, J.G., Zhou, F., 2006. Evidence from apatite and zircon fission track analysis for Mesozoic-Cenozoic uplift thermal history of Bogeda Mountain of Xinjiang, Northwest China. Marine Geology and Quaternary Geology, 26: 87-92 **(in Chinese with English abstract)**.
Shen, C.B., Mei, L.F., Xu, Z.P., Tang, J.G., Tian, P., 2007. Fission track thermochronology evidence for Mesozoic-Cenozoic uplifting of Daba Mountain, central China. Acta Petrologica Sinica, 23: 2901-2910 **(in Chinese with English abstract)**.
Shen, C.B., Mei, L.F., Zhang, S.W., Liu, L., Tang, J.G., Zhou, F., Yan, S.L., Luo, J.C., 2008. Fission track dating evidence on space-time difference of Mesozoic-Cenozoic uplift of the Yilianhabierga Mountain and Bogeda Mountain. Journal of Mineralogy and Petrology, 28: 63-70 **(in Chinese with English abstract)**.
Shi, H.C., Shi, X.B., Yang, X.Q., Jiang, H.Y., 2013. The exhumation process of Mufushan granite in Jiangnan uplift since Cenozoic: Evidence from low-temperature thermochronology. Chinese Journal of Geophysics, 56: 1945-1957 **(in Chinese with English abstract)**.
Shi, X.B., Kohn, B.P., Spencer, S., Guo, X.W., Li, Y.M., Yang, X.Q., Shi, H.C., Gleadow, A.J.W., 2011. Cenozoic denudation history of southern Hainan Island, South China Sea: Constraints from low temperature thermochronology. Tectonophysics, 504: 100-115.

Shi, X.B., Qiu, X.L., Liu, H.L., Chu, Z.Y., Xia, B., 2006. Cenozoic cooling history of Lincang granitoid batholith, western Yunnan: Evidence form fission track data. Chinese Journal of Geophysics, 49: 135-142 **(in Chinese with English abstract)**.

Shi, X.B., Shi, H.C., Yang, X.Q., Jiang, H.Y., Long, Y.K., Wu, Z.B., 2013. Erosion thickness of the main unconformities of Dangyang Subbasin, Jianghan Basin: Constrained by the meddle-low thermochronology. Acata Geologica Sinica, 87: 1076-1088 **(in Chinese with English abstract)**.

Song, C.H., Wang, J., Tan, F.W., Fu, X.G., Chen, W.B., He, L., 2014. Cretaceous rapid uplift of the Qiangtang Basin in the central Tibet: Evidence from the zircon fission track dating. Journal of Mineralogy and Petrology, 34: 77-84 **(in Chinese with English abstract)**.

Sun, D., 2011. The structural character and Meso-Cenozoic evolution of Micang Mountain structural zone, northern Sichuan Basin, China. Ph.D. Dissertation, Chengdu University of Technology, Chengdu, China, 195pp **(in Chinese with English abstract)**.

Sun, Q.S., Fang, S., Xie, R.X., Gong, F.H., Li, S.C., Zhu, H., Yuan, W., 2016. The application of zircon fission track to the study of the uplifting process in the Mohe Basin. Geological Bulletin of China, 35: 807-813 **(in Chinese with English abstract)**.

Tan, X.B., Lee, Y.H., Chen, W.Y., Cook, K.L., Xu, X.W., 2014. Exhumation history and faulting activity of the southern segment of the Longmen Shan, eastern Tibet. Journal of Asian Earth Sciences, 81: 91-105.

Tan, X.B., Xu, X.W., Lee, Y.X., Yuan, R.M., Yu, G.H., Xu, C., 2015. Differential Late-Cenozoic vertical motions of the Beichuan-Yingxiu Fault and the Jiangyou-Guanxian Fault in the central Longmenshan range and their tectonic implications. Chinese Journal of Geophysics, 59: 143-152 **(in Chinese with English abstract)**.

Tan, X.B., Xu, X.W., Li, Y.X., Chen, G.H., Wan, J.L., 2010. Apatite fission track evidence for rapid uplift of the Gongga Mountain and discussion of its mechanism. Chinese Journal of Geophysics, 53: 1859-1867 **(in Chinese with English abstract)**.

Tan, X.F., Pan, L.Z., Hu, G.A., Zhou, F.S., Xie, L.F., Li, G.N., 2010. Apatite fission track evidence for Mesozoic-Cenozoic differential uplift on both sides of Qinfang Tectonic Belt in southern Guangxi. Journal of Guilin University of Technology, 30: 325-331 **(in Chinese with English abstract)**.

Tang, S.L., Yan, D.P., Qiu, L., Gao, J.G., Wang, C.L., 2014. Partitioning of the Cretaceous Pan-Yangtze Basin in the central South China Block by exhumation of the Xuefeng Mountains during a transition from extensional to compressional tectonics? Gondwana Research, 25: 1644-1659.

Tang, W.H., Zhang, Z.C., Li, J.F., Li, K., Luo, Z.W., Chen, Y., 2015. Mesozoic and Cenozoic uplift and exhumation of the Bogda Mountain, NW China: Evidence from apatite fission track analysis. Geoscience Frontiers, 6: 617-625.

Tang, Z.B., Li, L., Shi, X.P., Hu, Q.Y., Gong, H.B., Wang, Q.H., 2011. Fission track thermochronology of Late Cretaceous-Cenozoic uplifting events of the Mengshan Mountain in the western Shandogn Rise, China. Acta Scientiarum Naturalium Universitatis Sunyatseni, 50: 127-133 **(in Chinese with English abstract)**.

Tian, X.S., Yang, J., Liu, D.X., Di, P.F., 2016. Mesozoic-Cenozoic lifting and cooling process of Jinchuan Copper-Nickel deposit: Evidence from fission-track thermochronology. Gansu Geology, 25: 50-55 **(in Chinese with English abstract)**.

Tian, Y.T., Kohn, B.P., Zhu, C.Q., Xu, M., Hu, S.B., Gleadow, A.J.W., 2012. Post-orogenic evolution of the Mesozoic Micang Shan Foreland Basin system, central China. Basin Research, 24: 70-90.

Tian, Y.T., Zhu, C.Q., Xu, M., Rao, S., Kohn, B.P., Hu, S.B., 2010. Exhumation history of the Micangshan-Hannan Dome since Cretaceous and its tectonic significance : Evidence from apatite fission track analysis. Chinese Journal of Geophysics, 53: 920-930 **(in Chinese with English abstract)**.

Tian, Z.H., Xiao, W.J., Zhang, Z.Y., Lin, X., 2016. Fisson-track constrains on superposed folding in the Beishan orogenic belt, southernmost Altaids. Geoscience Frontiers, 7: 181-196.

Wan, G.L., 2013. Hunan Qianlishan Granite constraints on mineralization. Ph.D. Dissertation, China University of Geosciences, Beijing, China, 112pp **(in Chinese with English abstract)**.

Wan, J.L., Zheng, W.J., Zheng, D.W., Wang, W.T., Wang, Z.C., 2010. Low closure temperature thermochronology study on the Late Cenozoic tectonic active of northern Qilianshan and its implications for dynamics of Tibetan Plateau growth. Geochimica, 39: 439-446 **(in Chinese with English abstract)**.

Wang, A., Garver, J.I., Wang, G.C., Smith, J.A., Zhang, K.X., 2010a. Episodic exhumation of the Greater Himalayan Sequence since the Miocene constrained by fission track thermochronology in Nyalam, central Himalaya. Tectonophysics, 495: 315-323.

Wang, A., Wang, G.C., Zhang, K.X., Garver, I.J., 2010b. An early Cenozoic tectonic event in eastern Kunlun Orogen, evidence from detrital fission track geochronology. Earth Science-Journal of China University of Geosciences, 35: 737-736 **(in Chinese with English abstract)**.

Wang, C., 2015. Cenozoic tectonic uplift and sedimentary evolution of the Tiklik area, Western Kunlun orogen. Ph.D. Dissertation, Zhejiang University, Hangzhou, China, 143pp **(in Chinese with English abstract)**.

Wang, F., Chen, H.L., Batt, G.E., Lin, X.B., Gong, J.F., Gong, G.H., Meng, L.F., Yang, S.F., Jourdan, F., 2015. Tectonothermal history of the NE Jiangshan-Shaoxing suture zone: Evidence from $^{40}Ar/^{39}Ar$ and fission-track thermochronology in the Chencai region. Precambrian Research, 264: 192-203.

Wang, M.M., 2013. A study on developmental mechanism and tectonic evolution of the Hanzhong Basin. Ph.D. Dissertation, Institute of Geology, China Earthquake Administration, Beijing, China, 130pp **(in Chinese with**

English abstract).
Wang, P., Liu, S.F., Gao, T.J., Wang, K., 2012. Cretaceous transportation of Eastern Sichuan arcuate fold belt in three dimensions: Insights from AFT analysis. Chinese Journal of Geophysics, 55: 1662-1673 **(in Chinese with English abstract)**.
Wang, X.M., Zhong, D.L., Zhang, J.J., Ji, J.Q., Wang, X.S., 2007. Low-temperature thermochronology constraints on sinistral strike slip movement of the Yi Shu Fault Zone between the Late Cretaceous and Early Paleogene. Acta Geologica Sinica, 81: 454-465 **(in Chinese with English abstract)**.
Wang, X.X., Song, C.H., Zattin, M., He, P.J., Song, A., Li, J.J., Wang, Q.Q., 2016. Cecozoic pulsed defromaiton history of northern Tibetan Plateau reconstructed from fission-track thermochronology. Tectonophysics, 672-673: 212-227.
Wang, Y.W., Mei, G., Xie, Q.X., Zhou, X.K., Wang, G., 2015. Apatite fission track evidence for the Cenozoic uplift process in Garze area on the eastern margin of the Tibetan Plateau. Geology in China, 42: 469-479 **(in Chinese with English abstract)**.
Weilson, C.J.L., Flowler, A.P., 2011. Denudational response to surface uplift in east Tibet: Evidence from apatite fission-track thermochronology. Geological Society of American Bulletin, 123, 1966-1987.
Wu, Q.H., Liu, S.S., Jonckheere, R., Wagner, G.A., 2002. Understanding the uplift pattern in Mesozoic and Cenozoic, eastern Dabie area, China using fission track dating of apatite. Nuclear Techniques, 25: 531-536 **(in Chinese with English abstract)**.
Xiao, H., Ren, Z.L., Zhao, J.Z., Wang, Q.Z., Shi, B.H., Song, L.J., 2011. The tectonic-thermal evolution history in the Kuruketage uplift, Xinjiang, China. Earth Science Frontiers, 18: 33-41 **(in Chinese with English abstract)**.
Xiao, P., Liu, J., Wang, W., Zeng, L.S., Xie, K.J., Pik, R., Zhong, N., 2015. The thermal history of the Baimaxueshan pluton in Deqin area and its implications for the tectonic-geomorphic evolution in the Three River Region of Tibetan Plateau, Acta Petrologica Sinica, 31: 1348-1360 **(in Chinese with English abstract)**.
Xu, C.H., Zhou, Z.Y., Chang, Y., Guillot, F., 2010. Genesis of Daba arcuate structural belt related to adjacent basement upheavals: Constraints from fission-track and (U-Th)/He thermochronology. Science China: Earth Sciences, 53: 1634-1646.
Xu, C.H., Zhou, Z.Y., Haute, P.V.D., Donelick, R.A., Grave, J.D., Ma, C.Q., Reiners, P.W., 2004. Apatite fission track (AFT) chronology study on Dabie Orogen. Science China: Earth Sciences, 34: 622-634 **(in Chinese)**.
Xu, G.Q., Kamp, P.J.J., 2000. Tectonics and denudation adjacent to the Xianshuihe Fault, eastern Tibetan Plateau: Constraints from fission track thermochronology. Journal of Geophysical Research, 105: 19231-19251.
Xu, Q.Q., Ji, J.Q., Sun, D.X., Zhao, L., 2015. Late Cenozoic uplift-exhumation history of Qinghe-Fuyun region, Altay, Xinjiang: Evidence from apatite fission track. Geological Bulletin of China, 34: 834-845 **(in Chinese with English abstract)**.
Xu, X.J., Ji, J.Q., Gong, J.F., Sun, D.X., Qing, J.C., Wang, L.N., Zhong, D.L., Zhang, Z.C., 2011. Evidence of rapid erosion driven by climate in the Yarlung Zangbo (Tsangpo) Great Canyon, the eastern Himalayan syntaxis. Chinese Science Bulletin, 56: 1123-1130.
Yan, Y., Carter, A., Palk, C., Brichau, S., Hu, X.Q., 2011. Understanding sedimentation in the Song Hong-Yinggehai Basin, South China Sea. Geochemistry Geophysics Geosystems, 12, Q06014.
Yan, Y., Carter, A., Bin, X., Lin, G., Stephanie, B., Hu, X.Q., 2009. A fission-track and (U-Th)/He thermochronometric study of the northern margin of the South China Sea: An example of a complex passive margin. Tectonophysics, 474: 584-594.
Yang, N., Zhang, Y.Q., 2010. Fission-track dating for activity of the Longmenshan Fault Zone and uplifting of the western Sichuan Plateau. Journal of Geomechanics, 16: 359-371 **(in Chinese with English abstract)**.
Yang, W., Jolivet, M., Guillaume, D.N., Guo, Z.J., 2014. Mesozoic-Cenozoic tectonic evolution of southwestern Tian Shan: Evidence from detrital zircon U/Pb and apatite fission track ages of the Ulugqat area, Northwest China. Gondwana Research, 26: 986-1008.
Yang, W., Li, J.F., Guo Z.J., Jolivet, M., Heilbronn, G., 2017. New apatite fission-track ages of the western Kuqa Depression: Implications for the Mesozoic-Cenozoic tectonic evolution of south Tianshan, Xinjiang. Acta Geologica Sinica (English Edition), 91: 396-413.
Yang, W., Zhu, W.B., Guo, S.P., Wan, J.L., Zhang, Z.Y., Su, J.B., 2008. Fission track evidence of Cenozoic basin-mountain coupling of southwest Shandong. Atomic Energy Science and Technology, 42: 557-560 **(in Chinese with English abstract)**.
Yu, X.Q., Liu, J.L., Zhang, D.H., Zheng, Y., Li, C.L., Chen, S.Q., Li, T., 2013, Uprising period and elevation of the Wenyu granitic pluton in the Xiaoqinling District, Central China. Chinese Science Bulletin, 58: 4459-4471.
Yuan, W.M., Du Y.S., Yang, L.Q., Li, S.R., Dong, J.Q., 2007. Apatite fission track studies on the tectonics in Nanmulin area of Gangdese terran, Tibet plateau. Acta Petrologica Sinica, 23: 2911-2917 **(in Chinese with English abstract)**.
Yuan, W.M., Hou, Z.Q., Li, S.R., Wang, S.C., 2002a. Fission track dating evidence for activity of the Yarlung Zangbo River thrust belt. Chinese Science Bulletin, 2002,47: 147-150 **(in Chinese)**.
Yuan, W.M., Wang, S.C. Li, S.R., Yang, Z.Q., Wang, L.F., 2002b. A fission track study on the metallogenic epoch of epithermal Au-Cu deposits in northern margin of Lhasa Terrane .Progress in Natural Science, 12: 541-544 **(in Chinese)**.

Yuan, W.M., Wang, S.C., Li, S.R., Yang, Z.Q., 2001. Fission track evidence for tectonic activity of Gangdese tectonic belt. Chinese Science Bulletin, 2001,46: 1739-1742 **(in Chinese)**.

Yuan, W.M., Zeng, Q.G., Bao, Z.K., Dong, J.Q., Carter, A., An, Y.C., Deng, J., 2009. Zircon fission track thermochronology constraints on mineralization epochs in Altai Mountains, northern Xinjiang, China. Radiation Measurements, 44: 950-954.

Yuan, Y.S., Yu, H., Li,T.Y., Li, S.J., 2016. Mesozoic tectonothermal events in central Lower Yangtze area and impacts on hydrocarbon generation of marine source rocks. Chinese Journal of Geophysics, 59: 2191-2202 **(in Chinese with English abstract)**.

Zhang, J., Wang, Y.N., Zhang, B.H., Zhao, H., 2015. Evolution of the NE Qinghai-Tibetan Plateau, constrained by the apatite fission track ages of the mountain ranges around the Xining Basin in NW China. Journal of Asian Earth Sciences, 97: 10-23.

Zhang, Z.C., Guo, Z.J., Wu, C.D., Fang, S.H., 2007. Thermal history of the Jurassic Strata in the Northern Tianshan and its geological significance, revealed by fission-track and vitrinite-reflectance analysis. Acta Petrologica Sinica, 23: 1683-1695 **(in Chinese with English abstract)**.

Zhang, Z.Y., Zhu, W.B., Zheng, D.W., Zheng, H.B., Yang, W., 2016. Apatite fission track thermochronology in the Kukuketage and Aksu areas, NW China: Implication for tectonic evolution of the northern Tarim. Geoscience Frontiers, 7: 171-180.

Zhang, Z.Y., Zhu, W.B., Shu, L.S., Wan, J.L., Yang, W., Su, J.B., Zheng, B.H. Apatite fission track thermochronology of the Precambrian Aksu bluseschist, NW China: Implications for thermo-tectonic evolution of the north Tarim basement. Gondwana Research, 2009, 16(2):182-188.

Zhao, J.F., Liu, C.Y., Mountney, N., Lu, J.J., Cao, J.L., Yang, Y., Xue, R., 2016. Timing of uplift and evolution of the Lüliang Mountains, North China Craton. Science China: Earth Sciences, 59: 58-69.

Zhao, W.J., Yuan, W.M., Liu, H.T., Song, G., 2013. Apatite fission track analysis on tectonic activities and paleotopography in southern Altai region, Xinjiang, China. Atomic Energy Science and Technology, 47: 1458-1467 **(in Chinese with English abstract)**.

Zhao, X.C., Liu, C.Y., Wang, J.Q., Zhao, Y., Zhang, D.D., Wang, L., Deng, Y., Guo, P., 2016. Mesozoic-Cenozoic tectonic uplift events of Xiangshan Mountains in northern Nouth-South Tectonic Belt, China. Acta Petrologica Sinica, 32: 2124-2136 **(in Chinese with English abstract)**.

Zheng, D.W., Wang, W.T., Wan, J.L., Yuan, D.Y., Liu, C.R., Zheng, W.J., Zhang, H.P., Pang, J.Z., Zhang, P.Z., 2017. Progressive northward growth of the northern Qilian Shan-Hexi Corridor (northeastern Tibet) during the Cenozoic. Lithosphere, 9: 408-416.

Zheng, Y., Xu, X.Q., Wang, D.E., Wang, C., 2009. Exhumation history of the Fuling Granite, Southern Anhui: New insights from apatite fission track analysis. Geological Review, 55: 385-394 **(in Chinese with English abstract)**.

Zheng, Y., Yu, X.Q., Yuan, W.M, Wang, D.E., Jiang, G.B., Zhu, D.L., Wang, C., Jia, Y.Y., 2011. Exhumation history of the Huangshan granite pluton, southern Anhui Province: New insights from fission-track analysis. Science China: Earth Sciences, 41: 40-51 **(in Chinese)**.

Zheng, Y., Zhang, J.J., Wang, J.M., Wang, X.X., Wang, M., 2014. Rapid denudation of the Himalayan orogen in the Nyalam area, southern Tibet, since the Pliocene and implications for tectonics-climate coupling. Chinese Sciences Bulletin, 59:874-885.

Zhou, Z.Y., Xu, C.H., Reiners P.W., Yang, F.L., Donelick R.A., 2003. The exhumation history of Tiantangzhai Region, Dabieshan since Late Cretaceous: Evidence from (U-Th)/He and fission track. Chinese Science Bulletin, 48: pp.598 **(in Chinese)**.

Zhu, W.B., Wan, J.L., Shu, L.S., Sun, Y., Wang, F., 2004. Mesozoic-Cenozoic thermal history of Turpan-Kumul Basin: Evidence form apatite fission track. Progress in Natural Science, 14: 1194-1198 **(in Chinese)**.

Zhu, W.B., Zhang, Z.Y., Shu, L.S., Wan, J.L., Lu, H.F., Wang, S.L., Yang, W., Su, J.B., 2007. Uplift and exhumation history of the Precambrian basement, Northern Tarim: Evidence from apatite fission track data. Acata Petrologica Sinica, 23: 1671-1682 **(in Chinese with English abstract)**.

Zou, B., Wang, G.Z., Deng, J.H., 2014. Evidence for apatite fission track of Pliocene rapid uplift of Zhongdian region on southeastern margin of Tibetan Plateau, China. Journal of Chengdu University of Technology (Science & Technology Edition), 42: 227-236 **(in Chinese with English abstract)**.
